# Sample Preservation and Storage Significantly Impact Taxonomic and Functional Profiles in Metaproteomics Studies of the Human Gut Microbiome

**DOI:** 10.3390/microorganisms7090367

**Published:** 2019-09-19

**Authors:** Oskar Hickl, Anna Heintz-Buschart, Anke Trautwein-Schult, Rajna Hercog, Peer Bork, Paul Wilmes, Dörte Becher

**Affiliations:** 1 Institute of Microbiology, University of Greifswald, D-17489 Greifswald, Germany; 2 German Centre for Integrative Biodiversity Research (iDiv) Halle-Jena-Leipzig, D-04103 Leipzig, Germany; 3 Helmholtz Centre for Environmental Research GmbH – UFZ, D-06120 Halle, Germany; 4 Luxembourg Centre for Systems Biomedicine, Université du Luxembourg, L-4362 Esch-sur-Alzette, Luxembourg; 5 European Molecular Biology Laboratory Heidelberg, D-69117 Heidelberg, Germany; 6 Max Delbrück Centre for Molecular Medicine, D-13125 Berlin, Germany; 7 Molecular Medicine Partnership Unit (MMPU), European Molecular Biology Laboratory and University Hospital Heidelberg, D-69120 Heidelberg, Germany; 8 Faculty of Biology, University of Würzburg, D-97074 Würzburg, Germany

**Keywords:** proteomics, metaproteomics, metagenomics, microbiome, microbiota, flash freezing, RNAlater, sample storage

## Abstract

With the technological advances of the last decade, it is now feasible to analyze microbiome samples, such as human stool specimens, using multi-omic techniques. Given the inherent sample complexity, there exists a need for sample methods which preserve as much information as possible about the biological system at the time of sampling. Here, we analyzed human stool samples preserved and stored using different methods, applying metagenomics as well as metaproteomics. Our results demonstrate that sample preservation and storage have a significant effect on the taxonomic composition of identified proteins. The overall identification rates, as well as the proportion of proteins from *Actinobacteria* were much higher when samples were flash frozen. Preservation in RNAlater overall led to fewer protein identifications and a considerable increase in the share of *Bacteroidetes*, as well as *Proteobacteria*. Additionally, a decrease in the share of metabolism-related proteins and an increase of the relative amount of proteins involved in the processing of genetic information was observed for RNAlater-stored samples. This suggests that great care should be taken in choosing methods for the preservation and storage of microbiome samples, as well as in comparing the results of analyses using different sampling and storage methods. Flash freezing and subsequent storage at −80 °C should be chosen wherever possible.

## 1. Introduction

Humans, as well as almost all multicellular organisms are not simply the sum of their respective cells, organs, and tissues, but an intimate and complex association of their own elements with many different microorganisms [1,2,3]. If microbial partners are lost or the composition of the microbiota is altered, phenotypical changes may lead to health defects [4,5]. Of the different habitats that parts of the host’s body provide, the digestive tract’s microbiome plays a central role. In mammals, the gut microbiota possesses the highest biomass [6] and arguably has the strongest impact on the host [7,8,9]. It profoundly influences the availability and composition of nutrients [10,11,12], susceptibility to disease [13,14,15], proper ontogenesis, host behavior [7,16,17], as well as the immune system [18,19,20,21].

In consequence, there has been great interest in understanding the nature of host–microbiota relations and elucidating the identity and role of the different members within the gut microbiota in recent years. This promises to generate profound new insights, not only in the context of human health, but also several technical applications, such as waste water treatment [22] and biogas production [23,24]. Furthermore, it could help to explain fundamental questions regarding interactions among uni- and multicellular organisms.

Analyzing samples as complex as human stool constitutes a considerable challenge as they contain vast amounts of highly diverse organisms, some of them adapted to very specific ecological niches [25]. Hence, preserving the information contained in a sample at the time of sampling is essential to permit the inference of biological meaning. In particular, the processing of samples after collection, as well as the subsequent storage conditions may have a profound effect on all downstream stages. Therefore, understanding the effects of different treatments is of immediate interest, especially as different institutions and laboratories handle sample processing in their own ways, possibly already introducing individual biases through, for example, different storage conditions.

So far, the most widely used approaches for microbiome research are metagenomics and metatranscriptomics, for the identification of taxa and the description of their activity [26,27]. However, as technology advances, “integrated multi-omics” approaches, which also include metabolomics and proteomics, will surely become a widely used approach in the near future. They will provide significantly more information, which will help our understanding of the complex network of microbial interactions, as well as its interactions with the host [28,29].

Proteomics provides the most immediate evidence for a member of a system to be active, since proteins are the central facilitators of all biological processes and constitute the enzymes which catalyze metabolic reactions [30]. It might be insufficient to rely on transcriptome data alone to infer activity of members of the microbiome, as transcript levels do not necessarily correlate with protein levels [31,32]. Additionally, vital information about the function and regulation of proteins is available through analysis of post-translational modifications [33].

This study investigated the influence of two popular sample storage methods on protein identification rates in a mass spectrometry-based metaproteomics study of human faecal samples and subsequent information gain. Flash freezing of samples and subsequent storage at −80 °C is proven to be the storage method that keeps samples close to their original state after specimen collection [34,35]. Furthermore, RNAlater is a popular RNA stabilizing agent [36,37,38,39,40] that is also often used when other biomolecules in addition to RNA are extracted from the same sample [41,42].

The results showed a strong bias introduced by the choice of storage and processing condition on the taxonomic origin of the identified proteins.

## 2. Materials and Methods

### 2.1. Ethics

Written informed consent was obtained from all subjects enrolled in the study. This study was approved by the Comité d’Ethique de Recherche (CNER; reference no. 201110/05, 17 October 2011) and the National Commission for Data Protection in Luxembourg.

### 2.2. Sample Collection and Processing

The stool samples were collected in Med Auxil stool collectors (Süsse GmbH & Co. KG, Gudensberg, Germany), homogenized, and aliquoted for the different storage conditions. Aliquots for the flash freezing processing were transferred to 50 mL falcon tubes, flash frozen in liquid nitrogen, and stored at −80 °C. After cryomilling, aliquots of 150 mg were created. To preserve RNA integrity prior to biomolecule extraction, 1.5 mL Ambion RNAlaterICE (Thermo Fisher Scientific Inc., Waltham, MA, USA) was added to frozen aliquots, and they were incubated for 16 h [43]. After homogenization and lysis, extraction was performed using the AllPrep DNA/RNA/Protein Kit (Qiagen, Venlo, Netherlands) with an in-house built automated sample preparation platform. For detailed information, see the parts concerning the processing of human faecal samples in chapter eleven of volume 531 of Methods in Enzymology [44].

For the RNAlater storage conditions, 200 mg aliquots of the same stool sample were stored in 1.5 mL Ambion RNAlater (Thermo Fisher Scientific Inc., Waltham, MA, USA) at 4 °C for 6 h, and after that stored at −80 °C. Samples were thawed on ice prior to homogenization, lysis and biomolecule extraction using the AllPrep DNA/RNA/Protein Kit (Qiagen, Venlo, Netherlands) with an in-house built automated sample preparation platform. For detailed information, see the parts concerning the processing of human faecal samples in chapter eleven of Volume 531 of Methods in Enzymology [44].

### 2.3. Metagenomics and Metatranscriptomics

DNA was treated with RNase, and RNA with DNase, before libraries were prepared for metagenomic and metatranscriptomic sequencing, respectively. Library preparation for metatranscriptomic sequencing, which was only successful for the flash frozen subsamples, included the depletion of ribosomal RNAs. Libraries were prepared using a dual barcoding system and sequenced at 150 bp paired-end on Illumina HiSeq 4000 (Illumina, Inc., San Diego, CA, USA) and Illumina NextSeq 500 (Illumina, Inc., San Diego, CA, USA) machines at the European Molecular Biology Laboratory (EMBL). Metagenomic and metatranscriptomic sequencing data, depleted of host sequencing data, is accessible as SAMN12288743 and SAMN12288744 in the NCBI short read archive under BioProject PRJNA289586. To avoid biases due to different search databases in the comparison of metaproteomics data from different storage conditions, metagenomic reads of all samples of the same donor were processed and de-novo assembled together with the metatranscriptomic reads of the flash frozen subsamples, using the Integrated Meta-omic Pipeline (IMP) [45]. On top of the published IMP workflow, metagenome-assembled genomes were generated and phylogenetically annotated with metagenomic operational taxonomic units (mOTUs; [46]) as described in [43]. In addition, further (incomplete) open reading frames were predicted using Prodigal [47]. Open reading frames were prepared as metaproteomics search database by removing nontryptic peptides from the beginning and/or ends of the predicted sequences if the start and/or stop codons, respectively, were missing. Entries were filtered to contain only predicted sequences with at least two tryptic peptides. In addition, human protein sequences (based on HG38) were added to the search database.

### 2.4. Prefractionation and Digestion

Of the protein solutions, 27 µL were separated on precast 12% Criterion XT Bis−Tris gels (Biorad, Hercules, CA, USA). In-gel digestion was done according to Bonn et al. [48]. Sample gel lanes were cut into ten pieces, proteins digested in-gel with trypsin, and after elution from gel, desalting was performed with ZipTip-tips (Merck Chemicals GmbH, Darmstadt, Germany), according to manufacturer’s instructions. After drying samples at 30 °C in a vacuum centrifuge, peptides were resuspended in 10 µL 0.1% acetic acid in H_2_O and transferred to glass vials.

### 2.5. High-Pressure Liquid Chromatography and Mass Spectrometry

Of the samples, 8 µL each were loaded onto in-house built columns (100 µm × 20 cm), filled with 3 µm ReproSil-Pur material (Dr. Maisch GmbH, Ammerbuch-Entringen, Germany), and separated using a nonlinear 80 min gradient from 1% to 99% buffer B (99.9% acetonitrile, 0.1% acetic acid in H_2_O) at a flow rate of 300 nL/min, operated on an EASY-nLC II (Thermo Fisher Scientific Inc., Waltham, MA, USA).

Measurement was done with an LTQ Orbitrap Velos Pro mass spectrometer (Thermo Fisher Scientific Inc., Bremen, Germany), performing one full scan in a range from 300 to 2000 *m*/*z*, followed by a data-dependent MS/MS scan of the 20 most intense ions, a dynamic exclusion repeat count of 1, and repeat exclusion duration of 30 s.

The mass spectrometry proteomics data have been deposited to the ProteomeXchange Consortium [49] via the PRIDE) partner repository [50] and is accessible using the dataset identifier PXD014482.

### 2.6. Database Searching

Tandem mass spectra were extracted, and charge state deconvoluted by msConvert (version 3.0.18188, ProteoWizard, Palo Alto, CA, USA) [51]. The 200 most intense peaks for each spectrum were selected, and data from all fractions merged into one mgf file for each sample. All MS/MS samples were analyzed using Mascot (version 2.6.2, Matrix Science, London, UK) [52], Sequest (version v.27, rev. 11, Thermo Fisher Scientific, Waltham, MA, USA) [53] and X! Tandem (version X! Tandem Vengeance (2015.12.15.2), The Global Proteome Machine Organization) [54]. Mascot, Sequest, and X! Tandem were set up to search a sample-specific database containing common contaminants (901940 entries), assuming trypsin digestion. Mascot and X! Tandem were searched with a fragment ion mass tolerance of 0.5 Da and a parent ion tolerance of 10 ppm. Sequest was searched with a fragment ion mass tolerance of 1.0 Da and a parent ion tolerance of 10 ppm. Formation of pyroglutamate from a glutamate or glutamine of the n-terminus, ammonia loss of the n-terminus, and oxidation of methionine were specified in X! Tandem as variable modifications. Oxidation of methionine was specified in Mascot and Sequest as a variable modification.

### 2.7. Criteria for Protein Identification

Scaffold (Proteome Software Inc., Portland, ME, USA; version 4.8.8) [55] was used to validate MS/MS-based peptide and protein identifications. Scaffold combines the scores of each search engine, as described by Searle et al. [56]. Peptide identifications were accepted if they could be established at greater than 99% probability by the Scaffold Local False Discovery Rate (FDR) algorithm. Proteins were filtered to a 1% FDR, requiring at least two identified proteins. Proteins that contained similar peptides and could not be differentiated on the basis of MS/MS analysis alone were grouped to satisfy the principles of parsimony. Proteins sharing significant peptide evidence were grouped into clusters.

### 2.8. Further Analyses

Significance testing was performed in Scaffold using a Benjamini–Hochberg-corrected t-test with a significance level of 0.05. Further, a fold change of at least 1.5 was required for a protein(-group) being significantly altered in abundance.

Functional and taxonomic annotation of identified bacterial proteins was conducted with donor-specific metagenome-based annotations (Table S1) as well as Prophane (https://gitlab.com/s.fuchs/prophane) [57]. Figures were created using the Matplotlib python library [58].

## 3. Results

To elucidate possible effects of initial sample storage on the human stool samples, metagenomics and metaproteomics analyses were performed. Of the same stool sample three aliquots were processed, each with the flash freezing or RNAlater refrigeration approach.

The results revealed significant differences in information content between flash frozen (FF) and RNAlater (RL)-treated samples. As of now, the term protein(s) will be used synonymously with protein groups as defined by the Scaffold cluster grouping method, unless explicitly stated otherwise.

### 3.1. Flash Frozen Samples Achieved a Higher Protein Identification Rate

In total, about 14,000 different proteins passed the filter criteria combined for all six samples (Tables S2 and S3).

**Figure 1:**
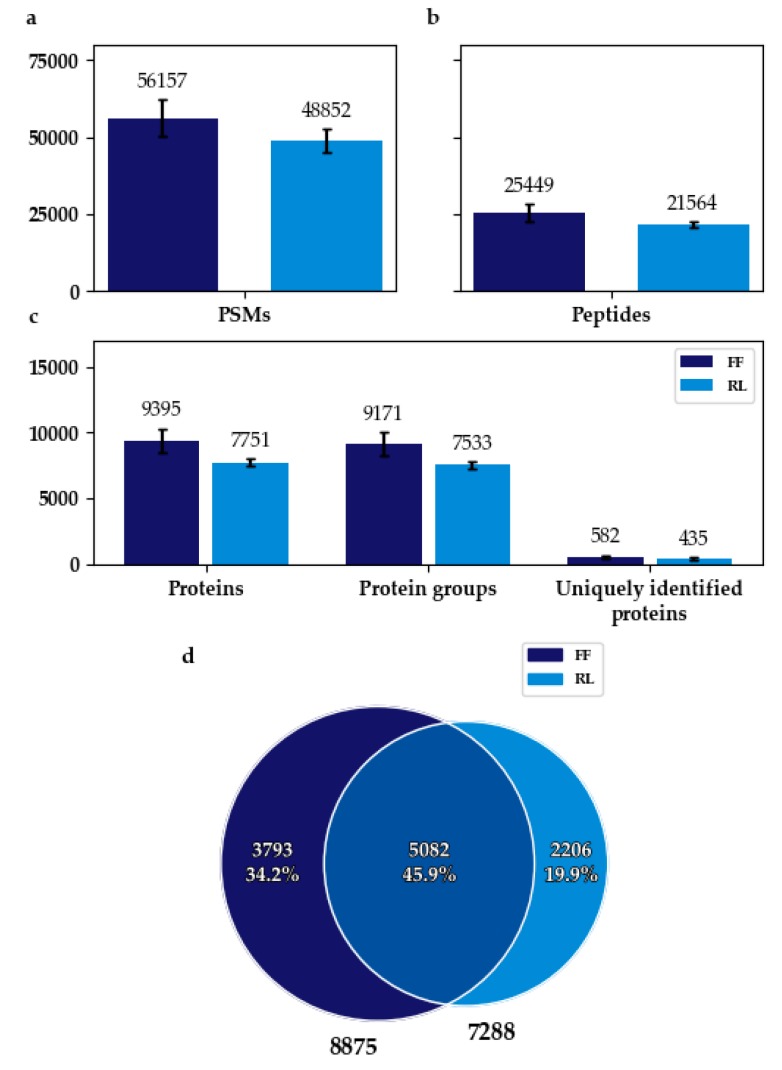
Identification rates and overlap of identified proteins of FF and RL stored samples. (**a**): Mean count of peptide-spectrum matches (PSMs); (**b**): Mean count of peptides identified, (**c**): Mean count of total number of proteins identified (Proteins), mean count of protein groups assembled by Scaffold (Protein groups), and mean number of proteins uniquely identified in one replicate (Uniquely identified proteins); (**d**): Overlap of bacterial proteins identified in at least two replicates in both storage conditions. Means are based on three replicates each. Error bars represent the standard deviation. FF: flash frozen; RL: RNAlater-treated.

The PSM (peptide spectrum match), peptide, and protein/protein group identification rates of FF samples were approx. 13%, 15%, and 17% higher, respectively, compared with RL samples. About 25% more unique proteins were identified in replicates of FF samples (Figure 1a–c).

When counting only bacterial proteins that were found in at least two replicates, 20% more could be found in FF. The overlap amounted to about 60% of the total number of proteins found in at least two replicates for FF, and 70% for RL (Figure 1d).

Of all bacterial proteins identified, about 2000 were significantly differentially abundant, around 1300 of these had a higher abundance in FF (Table S4).

### 3.2. Metaproteomics-Based Taxonomic Profiles Differed Significantly between Storage Conditions

**Figure 2:**
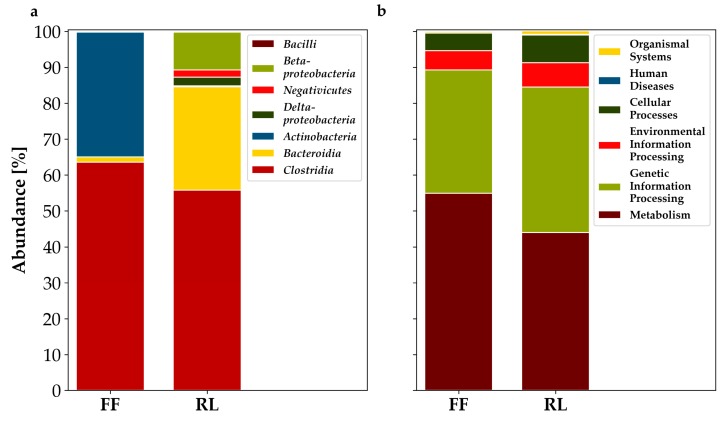
Analysis of proteins significantly different in abundance. (**a**): Proportions of taxonomy of proteins at class level; (**b**): Functional analysis of proteins which were mappable to the KEGG Orthology database and that had a functional annotation assigned. Contains only protein identifications that occurred in at least two of three replicates (normalized NSAFs, significance testing: Fold change ≥ 1.5, *t*-test, Benjamini–Hochberg-corrected, *p* < 0.05) and that could be annotated. FF: flash frozen; RL: RNAlater-treated.

At the class level, the taxonomic origin of proteins was vastly different between the tested conditions. About 35% of the assignable proteins that were significantly more abundant in FF belonged to *Actinobacteria*, whereas in RL, *Actinobacteria* only made up approx. 0.2%. The opposite was observed for the proportions of *Bacteroidia*. They made up about 30% of the significantly more abundant proteins of RL and only 1.5% of the significantly more abundant proteins in FF. Similarly, *Proteobacteria* and *Negativicutes* were almost nonexistent in FF, but made up approx. 5% and 10% in RL, respectively. The majority of significantly higher abundant proteins comprised clostridial proteins, with approx. 65% and 55%, respectively (Figure 2a).

Functional annotation using the KEGG Orthology (KO) database also revealed small differences between FF and RL samples. In both conditions, proteins assigned to metabolism and processing of genetic information possessed the largest share of proteins significantly more abundant. The share of metabolism-related proteins was 10% higher in FF, whereas the proportion of proteins related to processing of genetic information was about 6% higher in RL. Less than 10% of proteins were attributed to cellular processes and processing of environmental information, respectively. Both categories made up a slightly larger share in RL. The percentage of proteins assigned to organismal systems and human disease were below 1% for both conditions, respectively (Figure 2b).

Overall the functional differences were not as distinct as the taxonomic ones.

### 3.3. Integration of Metagenomics and Metaproteomics Data

**Figure 3:**
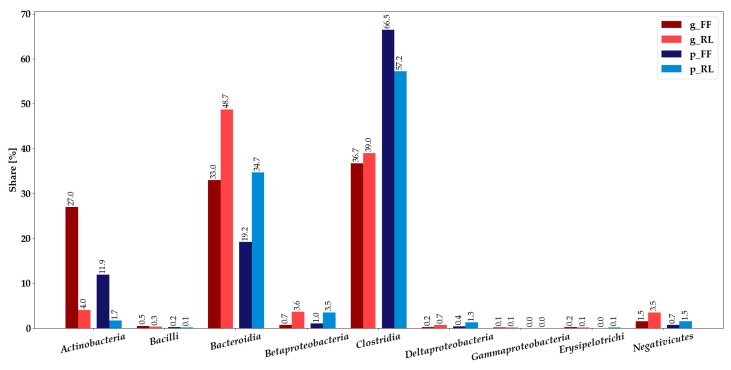
Metaproteomic and metagenomic proportions of identifications at class level (Tables S3, S5 and S6). For the metaproteomics data, proteins that could not be assigned using the bacterial annotations (Table S1) were excluded and made up about 52% and 49% for flash frozen and RNAlater-treated samples, respectively. (g_FF: metagenomic analysis for flash frozen, g_RL: metagenomic analysis for RNAlater, p_FF: metaproteomic analysis for flash frozen, p_RL: metaproteomic analysis for RNAlater).

The ratios of the shares of classes between FF and RL metagenomics and metaproteomics approaches were similar (Figure 3, Table S5). In the metagenomic analysis, *Actinobacteria* represented a much larger proportion for FF, whereas *Bacteroidia*, *Beta*-, *Deltaproteobacteria,* and *Negativicutes* were more abundant in RL. The ratios between FF and RL were similar for *Actinobacteria*, *Bacteroidia*, and *Clostridia*, however, *Actinobacteria* and *Bacteroidia* were more abundant in metagenomics analysis, and *Clostridia* were more abundant in metaproteomics analysis.

*Actinobacteria*, while having the same ratio between FF and RL on both omics levels (Table S5), made up a much larger share in the metagenomics analysis. *Bacteroidia* were much more abundant in the metagenomics analysis, whereas *Clostridia* made up a larger share in metaproteomics. *Gammaproteobacteria* were only detected in low amounts with metagenomics, *Erysipelotrichi* in low amounts on both omics levels. In conclusion, the taxonomic profiles of the metaproteomics and metagenomics analyses concur in most cases.

### 3.4. Annotation of Identified Proteins Using Prophane

Taxonomic annotation of proteins with Prophane using DIAMOND BLAST [59] against the NCBI RefSeq nonredundant database [60] produced similar results compared with processing with the metagenomics-derived annotation (Figure S1K). Flash frozen samples contained almost 20% *Actinobacteria*, while they made up less than 2% in RNAlater-treated samples. *Coriobacteriia* represented about 4% in FF and were almost not detected in RL. It has to be noted that *Coriobacteriia* form one class with *Actinobacteria* in the microbial annotations (Table S1) and are thus not detected separately during annotation with that resource (Figure 3, Tables S3 and S5; [46]). Proportions of *Bacteroidia* were 17% for FF and around 40% for RL. Analogous to the metagenomics-based annotation, *Negativicutes* and the different classes of *Proteobacteria* were more abundant in RL, whereas *Clostridia* made up about 50% in both storage conditions. Approximately 3% and 4% of bacterial proteins for FF and RL, respectively, could not be annotated by Prophane.

Functional annotation using the EggNOG [61] database showed both storage conditions to be similar. The share of proteins attributed to a metabolic function was 5% higher in FF, while ones assigned to cellular processes and signaling, as well as information storage and processing made up around 2% more in RL. To roughly 43% of proteins, no function or only a poor characterization could be assigned in both conditions (Figure S2K).

## 4. Discussion

The higher identification rates of FF and the large proportion of proteins identified exclusively in FF or RL indicate a strong impact of sampling and initial storage conditions on the information content of the sample (Figure 1). The alikeness of the functional profiles of flash frozen and RNAlater-treated samples (Figure 2b and Figure S2K), as well as the similarity of taxonomic profiles between metagenomics and metaproteomics, might hint to a difference in effect on overall cell preservation and/or proliferation (Figure 3). It may, for example, be possible that some bacteria overgrow others during refrigeration in the RNAlater-treated samples, or that immersion in RNAlater results in osmotic shock, thus distorting the original composition of the sample. Differences between the taxonomic composition after annotation with metagenomics-based data and Prophane are likely ascribable to the much larger amount of unannotated proteins using the metagenomics-based approach, shifting the proportions.

The small differences of functional profiles could be attributed to the differential preservation of taxonomic groups that have different physiological capabilities. A direct effect of the treatments on specific (functional) groups seems unlikely.

The higher identification rate of flash frozen samples could be attributable to superior conservational effect (Figure 1a–c). Results of Fouhy et al., for example, suggest that flash freezing keeps the taxonomic profile similar to that of samples that are directly analyzed [35], and it is therefore considered the gold-standard approach.

There are already various published metagenomics studies available discussing effects of different storage conditions [35,40,62,63,64,65,66], some of them showing, to an extent, similar results [62,64,65]. Several of these studies reported an increase in share of Gram negatives in RNAlater-stored samples compared with flash frozen ones, which is in agreement with this studies results (Figure 2a) [64,65]. Choo et al. observed significantly fewer *Actinobacteria* in RNAlater-treated samples compared with frozen ones as well, even though the methodology did differ [64]. Neither RNAlaterICE nor flash freezing was applied. Dominianni et al. observed similar microbial community compositions to the ones observed in this study in samples of one of the study subjects, although overall there were no significant differences [62].

Others observed no significant changes, but also detected almost no *Actinobacteria*, which this study found to be significantly less abundant in RNAlater-stored samples (Figure 2a) [35,63,66]. Hale et al. studied samples of a different species and their methodology differed in several key parameters, such as storage of RNAlater-immersed samples at room temperature for extended amounts of time and absence of RNAlaterICE from the samples stored at −80 °C [63]. These factors might well explain the distinctly different results. Fouhy et al. [35] and Guo et al. [66] serve as examples of studies using human faecal samples but detecting almost no *Actinobacteria*. The reason for this remains unclear, as both studies do not seem to share more characteristics of methodology and/or sample origin with each other than with other similar studies. Although, in the case of the Guo et al. study, the reason might be that faecal samples from infants were employed [66]. Voigt et al. [40] found no significant differences in taxonomic composition between RNAlater-stored and frozen samples as well. Again, the experimental setup differed significantly, with samples stored at −20 °C initially, requiring transport to the laboratory, and frozen samples not being flash frozen.

Taken together, this set of disagreeing and agreeing studies with varying degrees of similarity in methodology shows clearly why a standardized sample storage/processing approach might be crucial to achieve more reproducible results in the field of human microbiome omics studies.

The cause for the significantly lesser abundance of *Actinobacteria* and the increased share of Gram negatives in RNAlater-stored samples in this study could be a lesser tolerance of Gram negatives to the flash freezing process, differences in the ability of both methods to preserve oxygen-sensitive (anaerobic) bacteria and/or growth (in RNAlater-treated samples). No obvious difference could be detected in the amount of *Clostridia*, which made up a large portion of overall detected, but also significantly differentially abundant proteins on class level. As there is quite a lot of discussion about the phylogenetically most correct assignment of members of the class *Clostridia*, and as they are a phenotypically very diverse group [67,68], it is possible that the proteins significantly more abundant in one or the other condition belonging to *Clostridia* are ones of sub-groups, with properties that are, for example, preserved better in those conditions. In fact, significantly differentially abundant proteins for the clostridial genera *Ruminococcus*, *Blautia*, and *Clostridium* were only found in FF, whereas *Pseudoflavonibacter* proteins were only found in RL. Additionally, *Dorea* was much more abundant in FF, and *Oscillibacter* as well as *Faecalibacterium* were much more abundant in RL.

The central question regarding this study, as well as previous ones, is how relevant these observations are for a scientist planning or evaluating an “omics” experiment. Is initial sample storage something that introduces minor variations in information contained in a (stool) sample and that could probably be rendered irrelevant by often-observed large interindividual variability of the gut microbiome [40,66,69]? To resolve this, further investigations into the reaction of different members of a sample’s microbiota to RNAlater, RNAlaterICE, and the flash freezing process might be necessary and, once understood, will then allow to recognize conditions which modulate the microbial community structure in a certain way. This would need to be performed with sufficiently large sample sizes to take differences in the microbiota composition because of interindividual variation into account and enable researchers to separate effects caused by storage conditions or inherent variability.

Based on the distinct difference of results between sampling and storage conditions obtained in this study, it seems clear that proper and consistent storage of samples is essential for the ability to obtain high-quality (metaproteomics) data from environmental samples such as human stool. One has to keep in mind as well, that lacking other metaproteomics experiments to compare to, almost all of the studies cited here use metagenomics in one form or the other. To the authors best knowledge this is the first study combining metagenomics and metaproteomics to study the effects of storage conditions on human stool samples. It suggests that great care should be taken in the initial processing of microbiome samples for a multi-omics or metaproteomics experiment.

## 5. Conclusions

Depending on the processing method chosen for initial storage, the information content of samples for metaproteomics analysis might vary considerably. These findings could prove especially useful not only for future (meta) proteomics studies, but also the rapidly developing field of integrated multi-omics, as it indicates that initial storage conditions should be chosen that permit an analysis of all biomolecules of interest. Finally, flash freezing, storage at −80 °C, and handling without thawing is, as reported numerous times, the gold standard to maximize the preservation of a sample.

## Supplementary Materials

The following are available online at https://www.mdpi.com/2076-2607/7/9/367/s1, Table S1: Microbial Annotations; Table S2: Identified Proteins; Table S3: Identified Proteins—Taxonomy; Table S4: Significantly Differentially Abundant Proteins; Table S5: Metagenomics vs Metaproteomics; Table S6: Metagenomics—Taxonomy; Interactive Krona Plot K1: Taxonomical Annotation according to Prophane; Interactive Krona Plot K2: Functional Annotation according to Prophane.
