# Supplementary material for: Sample Preservation and Storage Significantly Impact Taxonomic and Functional Profiles in Metaproteomics Studies of the Human Gut Microbiome
Source: Microorganisms. 2019 Sep 19;7(9):367. doi: 10.3390/microorganisms7090367 (PMC6780314; doi:10.3390/microorganisms7090367)
Supplement: Supplementary file 1 [file microorganisms-07-00367-s001.zip › Supplement/Supplementary Figure K1 - Taxonomical Annotation according to Prophane.html]

Javascript must be enabled to view this page.

amountdescaff (mean)arl (mean)aff::aff\_01aff::aff\_02aff::aff\_03arl::arl\_01arl::arl\_02arl::arl\_030.9999999999995180.99999999999964651.00.99999999999999290.99999999999999360.99999999999999330.99999999999999130.9999999999999940.92792879135411080.94001613900865730.88926932560884690.94434576218430140.95017128627041910.93978589403065160.93731633285171360.94294619014455480.217489025126283460.012421747300952060.20569297282665920.2305046520336720.216269450518971620.01253993000344680.012469386408644830.0122559254907799770.178543705968959740.0117332796604068990.16787226931491390.19218207022318590.175576778369191780.0117809672127160170.0116760104341258830.011742861334393070.178443456487985120.01173327966040690.167687194656001040.192123267670231080.17551990713813550.0117809672127160170.0116760104341258830.011742861334393070.171939646764145140.01128009687047990.161213596711446360.184914977052020780.169690366529391460.0113602613005632010.0110770153172684150.0114030139936217020.171450947302370150.01128009687047990.16071751644048210.184352767277428050.169282558189622180.0113602613005632010.0110770153172684150.0114030139936217020.0272023964532000020.0006245423845490.024254171236241050.0298652846147880630.0274877335086868640.00065072997370113690.0005006355904367330.00072226158950821928.25856482374e-050.00.08.443443501209415e-050.000163322509700030560.00.00.00.0005532207700593.01880208622e-050.00051850276157820470.00055386473422935880.00058729481437002730.04.280773692275497e-054.775632566395048e-058.00501839308e-050.03.2911474287272356e-058.906673934339875e-050.000118172338161764670.00.00.00.000108803570787999980.00.000122157904561767680.000103831534947304960.000100421272856099880.00.00.02.63975731699e-050.03.315287874414722e-051.8786145687287452e-052.7253695078305834e-050.00.00.00.008337882329880.001797669977950.0071004260680339530.0093928590991616280.0085203618224413530.0018107892152229960.00164579814507796720.00193642257355220872.27950128569e-050.00.00.06.838503857071832e-050.00.00.00.07.16344884959e-050.00.00.00.00.00.00021490346548777720.0003926059912980.00.000303374505195039260.00043736048048220290.00043708298821635050.00.00.00.0005356373749397.84339833641e-050.00044451794291374550.0005676331057353240.00059476107616909148.204242277810006e-050.000119432129969012773.3827397345298245e-050.00054689727127900010.00.00050427166259515120.000486329919549268840.00065009023169243490.00.00.00.00059552005768800010.00.000444277347835379670.00064006915450179290.0007022136707268980.00.00.09.618708852299998e-050.08.193067919248468e-050.0001392785544308266.735203194578299e-050.00.00.04.36841265244e-050.00.06.662023340549625e-056.443214616778662e-050.00.00.00.001073790939523.46548259597e-050.00088114914224712750.00120341501825986870.0011368086580480024.983568656355665e-055.412879131554968e-050.00.0003306523526660.00.00019097925924445370.0004869845230627120.000313993275690903850.00.00.06.23972896009e-050.05.4785969318610964e-056.457515206121452e-056.783074742290442e-050.00.00.00.00.00.00.00.00.00.00.06.79030087021e-050.09.00965276169848e-057.658006896445748e-053.7032429525006925e-050.00.00.00.0002670527677810.00.000151687393456468860.000292145476843878570.000357325433041295340.00.00.00.000317901421220999970.00.000143487062501123960.0004268629770055870.000383354224157016170.00.00.00.0097752619485599980.0006511111959090.0079740989449271840.0113567428822348940.0099949440185195180.000420809168520287370.00083424073956885720.00069828367963703230.0003864183965060.00.00057293777773336110.00032465634870847470.00026166106307575320.00.00.00.000480081528310.00.00049032219639055950.00050324555864996770.000446676829888814040.00.00.01.73835601495e-050.00.05.215068044864639e-050.00.00.00.03.92017019699e-050.00.00.000117605105909702570.00.00.00.00.01324086323940.000230295588019000020.0117322476914437370.0144154234146114690.0135749186121085050.000192375336169338030.00026540947517501890.000233101952712991220.0005646255514460.00.000410752212561086640.0007119728724794050.00057115156929646350.00.00.09.34405907738e-050.00.000104909302950531648.917060254662168e-058.624186682419409e-050.00.00.00.00057917360732099990.00.00058357761852738840.00069971415257333730.000454229050861955760.00.00.06.94525686957e-050.00.00.00010445891582311950.00010389879026390460.00.00.00.00.00.00.00.00.00.00.09.64430134006e-060.01.5639593317596558e-051.3293310702596138e-050.00.00.00.02.43983764798e-050.02.7392984659305482e-052.3283435109395654e-052.251870967076179e-050.00.00.01.21028895625e-050.00.00.03.630866868738464e-050.00.00.00.0002083413278660.00.000304859957579263050.000190074403589730470.000130089622428379160.00.00.08.226481355569999e-060.00.02.467944406670418e-050.00.00.00.00.00076842628056500016.798775948700001e-050.000464680308235031170.00107242434044534310.00076817419301353256.184736486349081e-056.717521794032318e-057.494069565727614e-050.0005918014920950.00.00053686745173610690.00067158579852109520.00056695122602752330.00.00.00.0003809426256260.000229884443754000010.00030236142782712070.00059190685273364120.000248559596318503340.00017147255187271150.000310406812757417460.00020777396663273241.68690935016e-050.00.02.5726116917747437e-052.488116358711403e-050.00.00.04.77359539823e-050.05.359496998559768e-054.5554546953165415e-054.405834500801219e-050.00.00.00.1033921945550.007463694202130.101791394187045260.108323116526931230.100062072951373230.0079203595808715840.0072369806781047810.00723374234742421750.0004886994617750.00.00049608027096425170.00056220977459272440.00040780833976928360.00.00.00.0004886994617750.00.00049608027096425170.00056220977459272440.00040780833976928360.00.00.00.0065038097238399990.000453182789926999950.0064735979445546810.00720829061821028250.0058295406087440360.000420705912152815360.00059899511685746780.00033984734077136850.0065038097238399990.000453182789926999950.0064735979445546810.00720829061821028250.0058295406087440360.000420705912152815360.00059899511685746780.00033984734077136850.0065038097238399990.000453182789926999950.0064735979445546810.00720829061821028250.0058295406087440360.000420705912152815360.00059899511685746780.00033984734077136856.16183487628e-050.06.918126227732762e-055.8802552954851284e-055.687123105626064e-050.00.00.06.16183487628e-050.06.918126227732762e-055.8802552954851284e-055.687123105626064e-050.00.00.06.16183487628e-050.06.918126227732762e-055.8802552954851284e-055.687123105626064e-050.00.00.06.16183487628e-050.06.918126227732762e-055.8802552954851284e-055.687123105626064e-050.00.00.03.86311322118e-050.00.00011589339663552320.00.00.00.00.03.86311322118e-050.00.00011589339663552320.00.00.00.00.03.86311322118e-050.00.00011589339663552320.00.00.00.00.03.86311322118e-050.00.00011589339663552320.00.00.00.00.00.038574386040578740.000688467640545160.0374704733714663550.037964520382640410.04028816436767040.00075896279073078520.00079337597451894760.0005130641563869040.035088259906111020.00047676788217560.0342259645203340230.0351443446061045560.035894470591934480.00062345451940066480.000484893281472258660.000321955845653717370.0002576216383950.00.00040594042971923980.00024732404247104830.000119600442993852860.00.00.00.000220267568404999970.00.00029387821974935370.00024732404247104830.000119600442993852860.00.00.00.000220267568404999970.00.00029387821974935370.00024732404247104830.000119600442993852860.00.00.03.7354069990000004e-050.00.000112062209969886080.00.00.00.00.03.7354069990000004e-050.00.000112062209969886080.00.00.00.00.00.034830638267716030.00047676788217560.03382002409061480.0348970205636335160.0357748701489406240.00062345451940066480.000484893281472258660.000321955845653717370.0344650826576051240.00047676788217560.033646349838448720.034555154629484080.035193743504922240.00062345451940066480.000484893281472258660.000321955845653717370.008025310156640.0001285271078560.0080310759833953550.0086123318769486810.0074325226095762149.821039208779079e-050.000126921254805502810.000160449676675396580.00169796025783999990.00.00159103675243494390.00189147588887044760.00161136813221610330.00.00.00.0004917978682940.00.00060413043543597930.000447858611899009740.00042340455754728930.00.00.00.00.00.00.00.00.00.00.02.35163740233e-050.00.00.07.054912206979167e-050.00.00.00.001067660433730.00.00049602741992390320.00140893039087236540.00129802349039630960.00.00.06.848069149819999e-060.00.02.054420744946676e-050.00.00.00.00.0001702178587440.00.00013006740917368070.00019347027015361140.000187115896904531450.00.00.00.00276434990617999970.00.0024477345219621930.00269825505054134330.00314706014604562970.00.00.00.000463293057403000031.01517139296e-050.000273929846593054840.00055298158384814680.00056296774176904483.0455141788840172e-050.00.00.00.00.00.00.00.00.00.00.0004938809112010.00.000443121810665235830.00056496570486033590.00047355521807631410.00.00.00.0192602477644000030.000338089060390.0196292256588643760.0181643410440406740.0199871765903210130.00049478898552403380.000357972026666755850.000161506168978320760.00.00.00.00.00.00.00.00.00.00.00.00.00.00.00.01.89893024489e-050.02.1320011645214174e-051.8121541476652278e-051.7526354224885353e-050.00.00.01.89893024489e-050.02.1320011645214174e-051.8121541476652278e-051.7526354224885353e-050.00.00.00.000346566307661999960.00.000152354240520856320.00032374439267277670.00056360028979350430.00.00.00.000346566307661999960.00.000152354240520856320.00032374439267277670.00056360028979350430.00.00.00.00193661889397472020.000194687724513459970.0015712087349843460.00162142982727644220.0026172181196632990.000135508271330120320.00028435899599629660.000164195906215380330.00193661889397472020.000194687724513459970.0015712087349843460.00162142982727644220.0026172181196632990.000135508271330120320.00028435899599629660.000164195906215380331.35605368455e-050.00.00.04.0681610536595204e-050.00.00.01.35605368455e-050.00.00.04.0681610536595204e-050.00.00.00.001268446013868090.000166830077136459980.0011266948005312170.00119214588331619380.0014864973577568440.000135508271330120320.0002007860538653520.000164195906215380330.00073909966465199999.85640895287e-060.00041660647132024570.00076841314462061310.00103227937801410230.02.9569226858607262e-050.03.1541486380900003e-060.00.00.09.462445914284454e-060.00.00.00.000134341446032999980.00.000163861358384969360.00010445891582311950.000134704063891565980.00.00.00.0001180806023458.01172324059e-060.000124399334003268020.000148031381016616448.18110920148777e-050.02.403516972176701e-050.00.00027377015220.000148961944942999990.000421827636822733870.000171242441855844660.00022824037792201360.000135508271330120320.000147181657284977750.000164195906215380339.604416982630001e-060.00.02.881325094787713e-050.00.00.00.09.604416982630001e-060.00.02.881325094787713e-050.00.00.00.01.85779354784e-050.00.00.05.573380643513542e-050.00.00.01.85779354784e-050.00.00.05.573380643513542e-050.00.00.00.00062642999080012.7857647376999997e-050.000444513934453129140.00040047069301237140.00103430534493472430.08.357294213094453e-050.04.8308785429999995e-050.05.423810962542486e-054.61012015166034e-054.458704514810835e-050.00.00.04.04836087551e-050.02.7957788466713842e-054.7527011872787015e-054.5966025925884886e-050.00.00.00.000416346059528000040.00.000199688007805863350.000221763378113952490.00082758679266393160.00.00.00.000121291537087000012.7857647376999997e-050.00016263002855512718.507910150902852e-050.000116165481196799510.08.357294213094453e-050.00.00064602034249900021.70120338561e-050.00055914197483985750.00058444041937009470.00079447863328821810.02.4123697050392307e-052.691240451780635e-050.00064602034249900021.70120338561e-050.00055914197483985750.00058444041937009470.00079447863328821810.02.4123697050392307e-052.691240451780635e-050.00064602034249900021.70120338561e-050.00055914197483985750.00058444041937009470.00079447863328821810.02.4123697050392307e-052.691240451780635e-050.00064602034249900021.70120338561e-050.00055914197483985750.00058444041937009470.00079447863328821810.02.4123697050392307e-052.691240451780635e-050.0009034868979940.00.00111415814130811940.00061430552988930910.00098199702278440380.00.00.00.0009034868979940.00.00111415814130811940.00061430552988930910.00098199702278440380.00.00.00.0009034868979940.00.00111415814130811940.00061430552988930910.00098199702278440380.00.00.00.0009034868979940.00.00111415814130811940.00061430552988930910.00098199702278440380.00.00.00.0003709331167450.00.000350230140278971150.000358061427845668030.00040450778210942960.00.00.00.0003709331167450.00.000350230140278971150.000358061427845668030.00040450778210942960.00.00.00.0003709331167450.00.000350230140278971150.000358061427845668030.00040450778210942960.00.00.00.0003709331167450.00.000350230140278971150.000358061427845668030.00040450778210942960.00.00.00.0003709331167450.00.000350230140278971150.000358061427845668030.00040450778210942960.00.00.00.162168990700662260.3651276373692160.147296236433981460.16346815710126030.17574257856638430.355182980143163460.36533463098456440.3748653009809380.162071341820943350.36490637866096740.147122857717471240.163413361695337240.175677806049660960.35499691727092220.3651256198979460.3745965988150510.162071341820943350.364622981837670450.147122857717471240.163413361695337240.175677806049660960.35472891202318040.364543434675796530.3745965988150510.144846658691165970.31623340942559220.130550093507309480.14710618718319880.156883695382749020.306026399993574840.31522707347060250.3274467548135550.14475675977124650.31592329134762290.130550093507309480.147029495422964780.156690690383224280.30579153024664560.31497197082439630.327006372972782464.69396308683e-050.0002420492055740.06.354937047173063e-057.726952213311122e-050.000292368939071897540.000238320246452190130.000195458431197005484.52346058801e-058.92664582691e-050.09.147063792976864e-054.423317971042494e-054.785807995389171e-050.000103961646812404920.000115979648041022611.66348748589e-054.99263520286e-050.03.264957614490326e-051.7255048431930473e-053.4164974924591246e-057.421624644964884e-054.139783471152648e-050.000358118995560999950.0009581064924550.000212170066717732360.00030881702670280.00055336989326382010.00075485371243506860.00110367549718550950.00101579026774330348.15779746782e-050.000232110336032000024.4312181066523576e-057.532876064804478e-050.000125092982319995240.00021131557540010870.000128423210768264920.000356592221927338350.000115754484886000010.000424402848431999947.099229008563463e-050.000143506150592091350.000132765013981288780.00052830213251481010.000377544190008516250.000367362222771872331.07934856677e-050.000116673865435999991.777133342903829e-050.01.4609123574085306e-056.991441245438092e-050.00019539161484934588.471556900387737e-056.78704243668e-050.000183912642639000040.00.000117778548920989248.583272417935006e-050.000185733397568411180.000100866718315268420.00026513781203287490.00.00.00.00.00.00.00.00.05.16832911029e-050.00.00.03.536726143220149e-057.68279618672318e-054.2854650009292797e-054.98934558588e-050.0001521996751342.89733491588808e-054.925342042372159e-057.145359799376339e-050.000187385246053143280.000175537909777163849.36758695715952e-050.000336287703475000040.0003789443452150.00039752294330820170.000337885767839040240.000273454399276325940.00040393135425198550.000313377212879498150.00041952446851202430.00.00.00.00.00.00.00.01.87480028292e-053.95157001482e-053.0402527816942186e-052.5841480670741815e-050.02.704088822506885e-055.874066142763687e-053.27655507918584e-053.93612181704e-050.0001376901201050.06.0027606141410684e-055.805604836993274e-058.255225064059695e-050.000177922907053366640.000152595202620964950.003991776248070.00701902135917000050.00388233392975519080.0041245378175472860.0039684569969005860.0060540844761962110.0070051143393474530.0079978652619721851.68023883598e-050.0001028790620335.040716507939113e-050.00.08.269574684267476e-050.000122303101393522820.000103638337862226060.00.0001310387940430.00.00.00.000135508271330120327.359082864248887e-050.000184017282156438160.05.2826264338099995e-060.00.00.01.5847879301420114e-050.00.00.00.00.00.00.00.00.00.00.001428214530260.003685992634230.00134699476794358180.00147949764217790070.00145815118066247040.0033691733781002610.00406009872957797950.00362870579502445070.01.57906570664e-050.00.00.00.02.239173931344106e-052.4980231885758714e-055.99929025523e-050.000102076956075.9961424569131667e-056.900235728899169e-055.101492579875097e-050.000110957943986742757.386748589302455e-050.00012140543832966870.0005648387695210.001051288280450.00056284244883135480.00057099140084877530.00056068245888176480.00087233589535385390.00097573437170166640.0013057945743053744.86758134805e-052.32254742879e-050.00.00.000146027440441402880.06.967642286363308e-050.05.34389219359e-055.86620048671e-055.999790887768237e-055.0996904332525894e-054.932195259746498e-055.33638767627465e-055.796091813434965e-056.466121970428693e-050.000165611704341000020.0002084406629740.000172603544757101340.00014728818375911630.000176943384506829820.000229974965435193840.000208593852200307280.000186753171286502848.574472984299998e-050.0005178332184325.116802794851402e-050.000104753349967172640.000101312811613375920.000315009578367877130.0006513699484891790.00058712012843761470.02.41688099166e-050.00.00.07.250642974978381e-050.00.00.000134469544995999980.000106458632752000010.00011589339663552320.000144608042364046580.000142907195987526750.000100676753934308520.000218699144320495620.00.0002826348066230.0006190132086060.000210287479717340780.00028749894204330670.000350117998108877660.00094382622512506960.00036651046306223750.00054670293763213540.00.00.00.00.00.00.00.09.84258732066e-050.000320129455738.55490688098184e-050.000139401981175683127.03265696342403e-050.000348911862668991940.000412547669606473650.000198928834914806581.07013002592e-051.21626439168e-050.03.2103900777578966e-050.00.03.648793175031482e-050.05.9581636986999996e-050.000210037063715000010.00.000103598205655288577.514670530580058e-050.000216813234128192530.000117745325827982210.00029555263118768450.00.00.00.00.00.00.00.00.00.00.00.00.00.00.00.00.06.84334992564e-050.00.00.06.432125945803044e-056.304091482760113e-057.793832348356719e-050.0009582409352940.00230403331457000030.00107769165196538380.00067485864165565950.00112217251226079610.0027108010345818540.001879640704785330.0023216582043485360.07.0412541653e-050.00.00.00.000114885301970975410.09.635232298792646e-050.000223138939235999960.0003161201486170.00020506217760254210.000256183250545790650.000208171389560480250.000305425917114227130.000287210088649136250.00035572444008668930.000444040235036999940.00061040901607000010.000305811951305780150.000486958738950726840.00053935001485322030.00053861848675577830.00079631452061401770.00049629404083982780.01.28188032045e-050.00.00.00.00.03.8456409613602225e-059.97387169051e-050.000163641708761000020.00010519004948778188.940923093500472e-050.000104616870292367160.000126075663552173180.000185318550356174440.00017953091237371150.000195801315197000020.0004218267784140.00028341452578762810.000103598205655288570.000200391214148801620.00048656346458245930.00051515376652096170.000263763104139592650.000202996017972999980.000420801499180.000382728596147151164.6472985399801814e-050.000179786472371404580.00048629984469277050.00042255379026977490.000353550862576665641.34472489123e-054.9300721078e-050.02.0507651920197244e-051.9834094816774173e-050.05.197151343214651e-059.593064980198368e-050.0003774459291150.0008222009900270.000326591667178055360.00045323969601911240.000352506424146938860.00076628971241934810.0008423549141998210.00085795834346286030.06.84149609231e-060.00.00.00.00.02.0524488276922542e-054.31900925897e-056.24968537116e-053.674668673809272e-050.09.282359103088671e-055.685237671437429e-056.174993558161906e-056.888824883887321e-053.84924646285e-050.00.05.870271839974967e-055.6774675485706726e-050.00.00.00.07.03291542493e-050.00.00.08.473233827902137e-059.203162176835844e-053.422350270062962e-050.08.6634705677e-060.00.00.00.02.599041170310123e-050.00.0001223156893250.0005234436456080.000129308126219790280.000145282373534783029.235656822023894e-050.00042614329805523560.00058107195222373040.00056311568654356420.02.30416314835e-050.00.00.00.06.912489445046447e-050.00.000226764427875999970.000413166841030.000216925943729946160.000253370904542788160.00020999643535611680.00036698885215846420.00050067582376937260.00037183584716283740.00222244339123999980.002801568043130.0020579134662999280.0024928617875821710.00211655491982381880.0023736381210920.00292398268735589780.0031070833209509040.000100564679084999990.000166805834707000040.000112067012031653889.626830815466629e-059.335871706950746e-050.00012797455090029510.000120515886296247880.000251927066925866531.72384198889e-057.57510131877e-050.01.773123135253977e-053.398402831410697e-059.277104729523625e-050.00.000134481992267904690.03.71843146391e-050.00.00.00.00.00.000111552943917319430.02.66230378551e-050.00.00.07.986911356543515e-050.00.00.00.00.00.00.00.00.00.04.48131067489e-050.0001678530696832.337849552820037e-053.974241510052017e-057.131840961794362e-050.000178975889854093050.00018099184977383770.000143591469420903962.28302388611e-055.01233009451e-052.5632376949633675e-052.178695723847042e-052.107138239513627e-054.55963559485093e-054.952426275373543e-055.5249284132963515e-050.01.13708051201e-050.00.00.00.03.4112415360320365e-050.00.00056014787791699990.00126412596749999990.0005577727094723360.000418177550571218160.00070449337370720370.0015925845510337690.00115780205866398130.00104199129281391320.000226963117517000040.0003314403274990.000193707534376517310.000288132509478771260.000199049308696912240.000301505903709517740.000327479187459075540.00036533589132922120.000137026343497999980.0001270771199839.487184843016904e-050.000179176667894935420.000137030514168238080.000109933115779989519.165085155427621e-050.00017964739261517510.00.00.00.00.00.00.00.02.99894995584e-053.52826346581e-050.04.573531896488433e-054.423317971042494e-054.785807995389171e-050.05.798982402051129e-050.00.00.00.00.00.00.00.00.0002283807751450.000395184155948000040.000210717279652952180.000202993196770182480.000271431849012196470.00038693800625674960.00046122919453584830.000337385267050311661.41241426215e-050.000118680618027999990.04.237242786452519e-050.00.000128799623313243520.000163974443520452056.326778725076642e-050.000232860409265999990.000405850114506999940.00026076014242992720.0002770504898834340.00016077059548596760.000347891427357135870.000377860600914317860.00049179831525087470.07.60558031372e-050.00.00.05.176067016472408e-055.096952333993393e-050.000125437215907028720.04.47926199843e-050.00.00.03.182120355773274e-056.39987786822278e-053.855787771284656e-051.79509593556e-059.96017563212e-060.02.7376010401783496e-052.647686766514748e-051.4323320841307254e-051.555720605506297e-050.06.6947515237400004e-065.89171286091e-050.00.02.0084254571219973e-054.5611810961675316e-054.954104914196701e-058.159852572369724e-051.68498543555e-050.0001008767698920.05.05495630664511e-050.05.289577258061714e-055.745248902790798e-050.000192282048068011160.000109252095390.0006024553003021.6928248946761817e-050.000106666196594115960.00020416184062937590.00061646751334637190.00056469446850023130.00062620391905959230.00.00.00.00.00.00.00.00.0002532074554070.0005326571616380.00019907555981189240.00022994901595688740.000330597790453223570.000420557856615186230.00060572190833412930.00057169171996493110.0005248541644290.001265085935250.00052284194284948770.00051546287958949870.00053625767084767360.00104256160476224380.00136614392696407950.0013865522740156556.78026842277e-068.28882209511e-060.00.02.0340805268297602e-052.486646628532105e-050.00.04.64507552212e-053.2255645701e-055.215202848598544e-054.432807838134943e-054.287215879625803e-054.638552364761812e-055.038141345524239e-050.00.002452860747020.006234214582870.00196289917604333980.0030861408906835140.0023095421743403160.0058341546138036590.00607188583350981150.0067966033012938127.01275909716e-055.84035819382e-055.9733600909058216e-050.000101544496732606644.9104675273247085e-055.312879360520137e-055.770558369322918e-056.437636851616232e-050.0054304965454300010.00999011356170.0047521046181300990.0059428226715506490.005596562346600450.0091333219680360230.0097448755939513360.0110921431231150430.00131859540859999990.003002041196940.00150820704316884380.00112857058932472790.0013190085933007660.00323634890191151470.00265930537628985370.00311046931263268430.00.000109292152097999990.00.00.00.000198315279629160238.316931744992394e-054.639185921640904e-050.000269880878455999960.00041659154835800010.00025620071602408350.0002515178262833070.00030192409305922580.000281833120558540.000315169117039617060.00065277240747659730.0007024835766930.001288441197250.00066199862024814630.00063407766565399310.00081137444417632540.00108355708265946040.0013791216264524740.00140264488263341530.1185591566790.262439962815999960.106551475856850720.119639489205907750.129486504974024450.25549281066507510.26197545247519910.269851625308628444.22631879237e-057.76980995373e-050.07.669176023401759e-055.009780353720039e-058.025133477045139e-058.716460135537992e-056.567836248615213e-054.22631879237e-057.76980995373e-050.07.669176023401759e-055.009780353720039e-058.025133477045139e-058.716460135537992e-056.567836248615213e-054.76357319958e-050.000232419978432000040.00.00.000142907195987526750.000154618412158727050.000167938044850807950.00037470347828638074.76357319958e-050.000232419978432000040.00.00.000142907195987526750.000154618412158727050.000167938044850807950.00037470347828638077.27541949248e-050.0001890745266128.168390003829045e-056.942952035633043e-056.714916437968124e-050.000145304049980490470.000157821295161000260.00026409823469582257.27541949248e-050.0001890745266128.168390003829045e-056.942952035633043e-056.714916437968124e-050.000145304049980490470.000157821295161000260.00026409823469582257.27541949248e-050.0001890745266128.168390003829045e-056.942952035633043e-056.714916437968124e-050.000145304049980490470.000157821295161000260.00026409823469582250.00025756668123660.00037826635561470.000208608113943941760.000286755083314379130.00027733684645036970.000231927618238090570.00042687101305451950.00047600043555193630.00025756668123660.00037826635561470.000208608113943941760.000286755083314379130.00027733684645036970.000231927618238090570.00042687101305451950.00047600043555193630.01.6858645429e-050.00.00.00.05.057593628711591e-050.00.0002148697666110.000309218250390.000208608113943941760.000221640391906747180.00021436079398129010.000231927618238090570.00030228848073145430.000393438652200699834.26969146256e-055.21894597957e-050.06.511469140763194e-056.297605246907958e-050.07.400659603594927e-058.256178335123643e-050.001377498959074080.00466572443812980.00140530051568721250.00117682698062771380.00155036938090912760.0043263771384238220.0049209022119714410.004749893963987070.001377498959074080.00466572443812980.00140530051568721250.00117682698062771380.00155036938090912760.0043263771384238220.0049209022119714410.004749893963987070.00.00.00.00.00.00.00.05.91142717517e-050.0001903273150223.4327917484446115e-055.8355951286839746e-058.465894648375002e-050.000183193460481732310.000165812246814721780.000221976237769653366.89722704086e-050.000251102493620000030.00.000130652901380994787.626390984477572e-050.00021802199335601990.000236803539678694340.00029848194782439090.00.00.00.00.00.00.00.06.39454648043e-050.000282391728017000048.143860304117845e-055.234174300164143e-055.805604836993274e-050.000260875040559473840.000346308473135621050.000239991670355156535.42032940917e-050.000185962516395999984.70000949960354e-057.697283307786645e-053.8636954201133744e-050.000280417251976019460.00.000277470297212066740.0001777969146090.0006526356568950.00014982903211443330.0002784662767463570.00010509543496682520.00063638896853671910.0007643910608616860.00055712694128652840.00.00.00.00.00.00.00.00.01.96580866314e-050.00.00.05.8974259894282203e-050.00.08.20014256788e-066.2486810494e-050.02.4600427703630415e-050.03.8613349461197574e-058.646315180437638e-056.238393021630244e-052.07332223339e-051.84407280556e-056.219966700163401e-050.00.05.532218416688398e-050.00.00.0004261850982390.00090433195855699990.00074649525880737670.00019009675989394310.0003419632760159490.0007690103173842730.0008781919023278260.001065793655959790.05.39680548418e-050.00.00.04.995212508356497e-057.87396856847225e-053.3212353757201924e-050.0004983482802680.00204441908960.00028400994224210850.000365340087536440850.00084569481102676120.00177560818752365620.0023641921516637930.00199345692960597920.00.00.00.00.00.00.00.00.00.00.00.00.00.00.00.00.00448106423214190.01370615495470050.0042037514321277760.0047995936496311130.0044398476146761520.0140348101228050980.0140777109043743220.013005943836916990.00436955125078790.0135537318491915010.0041082618025055490.0046913748667282880.0043090170831382760.0138649476418419880.0138932155874959690.0129030323182327020.0001241192434310.00071420305681300010.000109351027470614640.000148008148423174450.000114998554399727270.00065778868073289230.00079525739375278930.00068956309595191961.36728492318e-050.000153383745596999980.04.1018547695490996e-050.00.000128730644994107950.000214826476542801630.000116594115254056520.0008916639368840.00176396230535999990.00072930188067586330.00099314732082256060.00095254260915437170.00159310205780435340.00195608853423620860.0017426963240345099.48987180758e-050.000240568093912999978.609223750067438e-050.000109764765515722418.883915121095395e-050.000181522882083937480.000308552052348228860.00023162934730737232.47535790802e-050.000112813489219999992.1253177752909425e-051.8064734136600078e-053.4942825351182085e-050.000121257057382985160.000102658052495007980.000114525357783454910.000162041751960999980.0005872840363750.000159134637092416730.00013526094518290630.000191729673606588360.00075280680293639420.0006099788473912480.000399066458798314230.00.00.00.00.00.00.00.00.000297473177864000030.001543644343490.0003104756103923290.000288999950318587840.00029294397288041490.0015578469526703550.00163761189226697860.00143547418553617750.01.48013192072e-050.00.00.00.04.440395762156956e-050.04.28110288951e-050.0001735599205514.165753427451986e-053.540798887603948e-055.136756353468703e-050.000143731237390228050.000225236491493065620.00015171203277099321.68890322531e-053.81203085557e-050.00.05.066709675921402e-055.481925521991232e-055.9541670447104624e-050.00.02.94262030956e-050.00.00.04.231661806449372e-054.596199122232639e-050.00.000149217523471000020.0007079588068530.000153917519695265770.00017646640507106790.00011726864564787670.00078131457497697450.00052469970449463920.0008178621410861920.0001184739739360.000226427669297999960.000126527471910011320.000107545571189588030.000121348878707915410.00013129339508346030.00026976577942052680.0002782238333904410.000186046186884999980.000483639224736000060.000173840094953284820.000236869757282250670.00014742870842049740.00039017800652135140.00057305033712754120.000487689330559694930.00.00.00.00.00.00.00.00.0002486570401630.000400033643955999960.000284465609923556940.000201491265369770120.000260014245196255530.00029784254488039970.00048496148730716630.000417296899681733036.09468964026e-050.0001518453045633.71154947254276e-059.487318131149082e-055.0852013170744e-050.000176303684771186660.00017292477423068640.000106307454687050475.51087254443e-050.0001556057875583.182987654074229e-058.116408717711867e-055.2332212615150644e-050.000141552067469257130.0001537460973986270.00017151919780714610.001882777586810.006056454590050.00184329962959793290.002023292198355920.0017817409324826960.00671254117885969940.00571395004769945350.0057428725435836470.0001115129813540.0001524231055099.548962962222688e-050.00010821878290282490.00013083053153787660.000169862480963108570.000184495316878352430.000102911518684287660.0001115129813540.0001524231055099.548962962222688e-050.00010821878290282490.00013083053153787660.000169862480963108570.000184495316878352430.000102911518684287660.01.41679975212e-050.00.00.00.02.00907476968758e-052.2413244866823385e-050.01.41679975212e-050.00.00.00.02.00907476968758e-052.2413244866823385e-050.01.41679975212e-050.00.00.00.02.00907476968758e-052.2413244866823385e-050.0110357990623999990.02943618413950.0106734202483645520.0099745692782088980.0124594076604966060.029964093100158070.0297129650329359330.028631494285477320.0110357990623999990.02943618413950.0106734202483645520.0099745692782088980.0124594076604966060.029964093100158070.0297129650329359330.028631494285477320.0110357990623999990.02943618413950.0106734202483645520.0099745692782088980.0124594076604966060.029964093100158070.0297129650329359330.028631494285477320.00.000283396823296999960.00.00.00.000268005247741793530.00058218522214946760.00.00.000283396823296999960.00.00.00.000268005247741793530.00058218522214946760.00.00.000283396823296999960.00.00.00.000268005247741793530.00058218522214946760.00.00.000283396823296999960.00.00.00.000268005247741793530.00058218522214946760.06.13449241463e-059.52090238406e-056.446684979250182e-055.4795405923062686e-056.477251672334809e-058.919350558760165e-050.000103796889844397969.263667608981837e-056.13449241463e-059.52090238406e-056.446684979250182e-055.4795405923062686e-056.477251672334809e-058.919350558760165e-050.000103796889844397969.263667608981837e-056.13449241463e-059.52090238406e-056.446684979250182e-055.4795405923062686e-056.477251672334809e-058.919350558760165e-050.000103796889844397969.263667608981837e-056.13449241463e-059.52090238406e-056.446684979250182e-055.4795405923062686e-056.477251672334809e-058.919350558760165e-050.000103796889844397969.263667608981837e-056.13449241463e-059.52090238406e-056.446684979250182e-055.4795405923062686e-056.477251672334809e-058.919350558760165e-050.000103796889844397969.263667608981837e-053.63039555726e-050.000126049684407999990.000108911866717720580.00.09.68693666536603e-050.000105214196774000160.000176065489797215043.63039555726e-050.000126049684407999990.000108911866717720580.00.09.68693666536603e-050.000105214196774000160.000176065489797215043.63039555726e-050.000126049684407999990.000108911866717720580.00.09.68693666536603e-050.000105214196774000160.000176065489797215043.63039555726e-050.000126049684407999990.000108911866717720580.00.09.68693666536603e-050.000105214196774000160.000176065489797215043.63039555726e-050.000126049684407999990.000108911866717720580.00.09.68693666536603e-050.000105214196774000160.000176065489797215040.04.10937124201e-050.00.00.03.536726143220149e-058.791387582793975e-050.00.04.10937124201e-050.00.00.03.536726143220149e-058.791387582793975e-050.00.02.9304625276000003e-050.00.00.00.08.791387582793975e-050.00.02.9304625276000003e-050.00.00.00.08.791387582793975e-050.00.02.9304625276000003e-050.00.00.00.08.791387582793975e-050.00.02.9304625276000003e-050.00.00.00.08.791387582793975e-050.00.00.00.00.00.00.00.00.00.00.00.00.00.00.00.00.00.00.00.00.00.00.00.00.00.00.00.00.00.00.00.00.00.00.00.00.00.00.00.00.00.01.17890871441e-050.00.00.03.536726143220149e-050.00.00.01.17890871441e-050.00.00.03.536726143220149e-050.00.00.01.17890871441e-050.00.00.03.536726143220149e-050.00.00.01.17890871441e-050.00.00.03.536726143220149e-050.00.00.50987402247883880.48385205018143170.50065232547555240.51100365030503980.51796609165703920.491215451195964440.47934010363333140.48100059571470810.00369741466634999040.00088555389253610.00372791664442017950.00372533385401086240.00363899350061512840.00104658111485327550.00068979052173487320.00092029004101999450.00030824940470440.00043733457811160.000393774154477516340.00021343148850279350.00031754257113286810.00050287663501915920.000382457445207679370.00042666965410711960.00023976694305610.00043733457811160.00018832676953272520.00021343148850279350.00031754257113286810.00050287663501915920.000382457445207679370.00042666965410711965.42421473821e-050.000387083594160.00.00.000162726442146380810.000352123683164400240.000382457445207679370.00042666965410711960.00.00.00.00.00.00.00.05.42421473821e-050.000387083594160.00.00.000162726442146380810.000352123683164400240.000382457445207679370.00042666965410711960.0001855247956740.00.00018832676953272520.00021343148850279350.00015481612898648730.00.00.00.0001855247956740.00.00018832676953272520.00021343148850279350.00015481612898648730.00.00.00.05.02509839516e-050.00.00.00.00015075295185475890.00.00.05.02509839516e-050.00.00.00.00015075295185475890.00.06.84824616483e-050.00.000205447384944791170.00.00.00.00.06.84824616483e-050.00.000205447384944791170.00.00.00.00.06.84824616483e-050.00.000205447384944791170.00.00.00.00.00.003389165261645590.000448219314424499970.00333414248994266330.0035119023655080690.003321450929482260.00054370447983411630.00030733307652719390.0004936203869128757.35964707318e-050.09.825744497359576e-054.1758334707068304e-058.077363251468903e-050.00.00.07.35964707318e-050.09.825744497359576e-054.1758334707068304e-058.077363251468903e-050.00.00.07.35964707318e-050.09.825744497359576e-054.1758334707068304e-058.077363251468903e-050.00.00.03.38807333952e-050.0001570657442150.00.00.000101642200185657330.000183286263653202280.000199075493896094548.883547509525136e-053.38807333952e-050.0001570657442150.00.00.000101642200185657330.000183286263653202280.000199075493896094548.883547509525136e-053.38807333952e-050.0001570657442150.00.00.000101642200185657330.000183286263653202280.000199075493896094548.883547509525136e-050.000252911237386138.50072729911e-050.000346119032349526470.000252255835694247150.000160358844114664274.963060143366548e-050.000108257582631099369.71336349088058e-050.00012233741001381.23320132094e-050.000107189939971195350.000171705600054238888.811669001602439e-050.00.03.6996039628275565e-052.68655020493e-050.00.08.059650614790805e-050.00.00.00.00.01.23320132094e-050.00.00.00.00.03.6996039628275565e-059.54719079645e-050.00.000107189939971195359.110909390633083e-058.811669001602439e-050.00.00.00.00.00.00.00.00.00.00.00.000130573827372337.26752597817e-050.000238929092378331128.05502356400083e-057.224215409863989e-054.963060143366548e-050.000108257582631099366.0137595280530236e-058.75117720513e-060.00.02.6253531615377788e-050.00.00.00.03.08432946088e-050.03.267356001531618e-052.0398761733010354e-053.9457562077971994e-050.00.00.03.55211657574e-050.03.9880962959871216e-053.389794229162015e-053.27845920206679e-050.00.00.00.03.65893989047e-050.00.00.04.963060143366548e-050.06.0137595280530236e-055.545818980100001e-053.6085860876999994e-050.000166374569403143720.00.00.00.000108257582631099360.00.003028776820132460.00020614629721840.00288976601261954150.00321788819510675360.0029786762526672490.000310787614747248630.00.00030765127690881780.003028776820132460.00020614629721840.00288976601261954150.00321788819510675360.0029786762526672490.000310787614747248630.00.00030765127690881780.01.89626354534e-050.00.00.05.688790636028636e-050.00.00.00.00.00.00.00.00.00.08.27524965629e-060.00.00.02.4825748968879924e-050.00.00.00.0001052066109390.00.00010607153925835560.000106523485281908840.000103024808277236790.00.00.00.00.00.00.00.00.00.00.07.91566106617e-060.02.3746983198522268e-050.00.00.00.00.00.00092909728143100020.00.0011080884949993660.00077263270356795550.00090657064572477760.00.00.00.001978282017040.0001871836617650.00165185899516329720.0023387320062568890.0019442550496963550.00025389970838696230.00.00030765127690881780.478369105456370070.45095662158621820.469943837537905250.479741320944696240.48542215788751630.456101839094943060.447998970898615350.44876905476485270.478186602395063440.449777528160409830.469711481683094840.47960295833071520.48524536717238830.454940790065810230.446961288160044770.44743050625512940.026537411854589720.061553840967528270.0267552113061586760.0259116830844625740.026945341173139930.059886150937466720.061955970393606060.062819401571414770.00.00.00.00.00.00.00.00.00.00.00.00.00.00.00.00.00065835476169670.00255320296280150.00058033362025070280.00074239794233150890.00065233272250827050.00202098884851519950.00307359383165441540.00256502620823079830.05.09815676732e-050.00.00.07.331450546128091e-057.963019755843781e-050.01.06715744251e-054.66430716553e-050.03.201472327541903e-050.06.700131193544838e-053.447149341674479e-053.8456409613602225e-050.0003399263478110.001256406221710.000251762201814562230.00038676664910675530.000381250192511500150.00105195952596818250.00140593717474886760.00131132196440838350.000242230649674000030.00096537290371900010.00032857141843614060.000223686219841090220.000174434310745090420.00066008726948243610.00122510868154125720.00101092276013477836.55261897866e-050.000233799198044000040.09.993035010824436e-059.664821925167995e-050.00016862623566785160.00032844628438910760.00020432507407403430.0238673447696509180.0554384098976845740.023892211956055320.0232921123005820350.0244177100523074180.0544669583536769660.05533312262210420.056515148717174124.75192929868e-050.04.0476201213003626e-056.880776345761701e-053.3273914289633096e-050.00.00.00.00.00.00.00.00.00.00.00.0001124924675640.08.833568342903072e-050.00012665072959621860.000122490989665718220.00.00.00.00.00.00.00.00.00.00.04.96242789804e-050.000150668291014999980.09.051283020275535e-055.83600067383617e-050.000157856494088752740.00010287304318086140.000191275335774461326.85532674479e-060.0006276370993240.00.02.056598023436732e-050.00065867395665928440.0006611439540057550.00056309338730671093.47631520571e-050.04.655631727504279e-059.892961698841933e-064.7840177197541135e-050.00.00.00.00.0001842872942330.00.00.00.000327178825798747650.000132330798853712469.33522580458157e-051.92299081138e-050.00.02.9326464069086134e-052.8363260272333558e-050.00.00.00.00.00.00.00.00.00.00.02.04894228963e-052.23639143527e-050.06.146826868880454e-050.03.216062972901522e-053.493111332896805e-050.02.98579149316e-051.09710477877e-050.05.191576747365248e-053.7657977321037455e-050.00.03.291314336299289e-058.00773080965e-068.66396275027e-060.00.02.402319242893769e-052.599188825082049e-050.00.00.00.00.00.00.00.00.00.00.0001318471601850.000217966559249000020.000107786386378030290.000128262484346670820.000159492609830276320.000210910330098867670.000187428469769900130.00025556087787735660.00.00.00.00.00.00.00.00.00.0001179959095170.00.00.07.960551913122581e-050.000129694727706564540.000144687481714543040.03.93368393344e-050.00.00.00.00.000118010518003270440.00.00.000292301785434000030.00.00.00.000222298290303894240.000184495316878352430.000470111749120108238.86104037745e-050.000260550837272999970.000109351027470614646.658694766708077e-058.989323618570229e-050.000240804140896593260.000313066183427427730.000227782187494855872.79897024697e-050.0002370496656511.920612947075951e-054.8974364217354886e-051.5788613721001538e-050.00022031619343895290.00023006513439636450.00026076766911746580.000104877620640000010.07.999721183690982e-050.00013599174488673579.864390519492996e-050.00.00.00.00.00.00.00.00.00.00.00.00359985978142000030.0062094243744300010.00359706920833315240.0036987293585185530.003503780777418280.0059524439197549980.0066091718107416530.006066657392779311.61459844352e-050.01.8127710436305102e-051.5408155587100067e-051.490208728212177e-050.00.00.00.02.91092611075e-050.00.00.00.08.732778332242014e-050.00.02.5259158903e-050.00.00.03.4262034512445194e-050.04.151544219650241e-050.000176877368340.00.000150661415626180160.000256117786203352250.000123852903189189840.00.00.00.000169812874143000020.00264957786717999940.000143222529378859220.00018895070321371320.000177265389835747140.00280578563643255150.0029729442860288860.00217000367908093970.000369220810363000030.001621412200830.00028126062291753370.000419046576515680150.000407355231655011670.00169517933135912570.00130127129479977740.00186778597633015596.95197470046e-050.09.224168303643685e-057.840340393980171e-053.7914154037507105e-050.00.00.02.71194199864e-058.38257775645e-050.04.9464808494209664e-053.1893451465027425e-053.450711344314938e-050.000112439205994532340.000104531013255857270.00069960618921300010.000119339815276000010.00097958563011698860.00052586921882057110.00059336371870106169.171282242114608e-059.961344105219026e-050.000166693182355538182.24844112296e-051.25582258953e-051.9151874867734765e-053.255734570381596e-051.5744013117269895e-051.7034231847995348e-050.02.0640445837809107e-050.00.000127868896107999980.00.00.06.04523115207053e-056.565998746046626e-050.000257494389342985460.000284364329390999940.0002537509647710.000192333722075974630.00024031477386314540.000420444492235254960.000316832114739286340.000203764827752313660.00024065595182229990.00.0001266905837330.00.00.06.23268018004171e-050.000101543934095837370.000216201015301390434.92668267115e-055.62153224209e-050.02.9252031419164593e-050.000118548448715273986.121947283441984e-053.324661801614979e-057.41798764120246e-050.05.58284808724e-050.00.00.06.522014855206407e-057.083855072492209e-053.1426743340148055e-050.0009353548588030.001012308642220.00087121107794061130.00110076387535343670.00083408962311362760.00112985125402252440.00089609433694495210.00101098033568065240.000209126033770.0002979735981980.00020117993184504770.000168646679810213270.000257551489655074960.00028025041457835470.00022787359136873250.000385796788646572030.000251065609380.0003129092939880.000226224043635298440.000277158522613806570.000249814261891121750.000221631794890746890.00025899225782984440.00045810382924410910.000266582899420.00.000334803145835955830.000189716878669149820.00027522867375375520.00.00.00.00.00.00.00.00.00.00.00.0004620428602923.35440016421e-050.00056878677229899260.00035396815201294090.000463373656564810170.08.396902242540398e-051.6662982500762653e-050.00498259294071000040.01301391614490.0053442723900751810.00430440006852504750.0052991063635387350.0130767526383293310.0130875436200614420.0128774521763354130.00.00.00.00.00.00.00.00.00.0002313064068540.00.00.00.00021191867537882120.000258687972480040570.000223312572704382030.000102827006852999997.17612356022e-055.116802794851402e-050.000130804581204199180.000126508411405081580.04.94308207485397e-050.00016585288605797690.0006130970905210.00088772015817899990.00065123786126828360.0006188182654184270.000569235144876090.00070977518786447540.00116240338330676350.00079098190336709610.01.08488757633e-050.00.00.00.00.03.254662728990834e-050.00.00.00.00.00.00.00.00.00.000106916234676999990.00.00.09.725996893855409e-050.000105638447567443730.00011785028752555520.00.000370549284685999950.00.00.00.000280931365465435960.00034209135403871980.00048862513455450860.00.00.00.00.00.00.00.00.05.59850568718e-050.00.00.00.07.938889392947285e-058.85662766858718e-050.01.85257083692e-050.00.00.05.55771251077452e-050.00.00.0001041137617030.0001946066403850.000116892477641001839.935603775130043e-059.609276971575076e-050.000155951329504922950.000112923857744508790.000314944733904500970.04.70211214803e-050.00.00.02.0722055237767545e-054.501432130021656e-057.53269879029322e-058.59455658401e-060.00.02.578366975201533e-050.00.00.00.03.55893107778e-050.000135849914827999980.00010676793233351350.00.00.000217202055175354630.000159122399451815413.1225289857198404e-052.74514318815e-050.04.5198424687854053e-050.03.715587095675696e-050.00.00.05.8563518186699995e-060.00.01.7569055456022638e-050.00.00.00.01.01365878445e-050.00.03.0409763533379554e-050.00.00.00.04.71937214865e-050.000470710457279000040.05.777092921880125e-058.381023524080516e-050.000362713869124231860.000426789918493030670.00062262758422022650.000148897798347000020.00.0001602310990827710.00010345871076437030.00018300358519484250.00.00.00.000174922513314000020.000181687721531999980.00033898818515890540.00.00018577935478378480.000301505903709517740.00.000243557260886147470.0001585595709540.000305410497517000030.00033961710572506294.989307523441927e-058.616853190342521e-050.00028019760205387690.00039818581367432370.00023784807682241610.00.00.00.00.00.00.00.00.00.00.00.00.00.00.00.00.0074733018087399990.0122485740197999980.0070682948974015550.0077613421147054720.00759026841410607650.011346298051429540.011940992197592920.0134584318102325930.001683595932060.01188963074790.00150197522931421820.00164974743197510720.00189906513487609520.0123456468552513060.0119461194136057090.0113771259748860230.00103097680143599990.00197345664551000020.00116688147638151150.00093461378197846720.0009914351459460890.00207366875940501050.001887720449599080.00195898072752850520.0004443427195720.00083744450756300010.0004769683007260980.00048263401922742270.000373425838761376360.0007232670462449280.00085889804291035710.00093016843353503560.0004452601602250.00094190785743600010.00051304977470294110.00030164975780559870.00052108094816708580.00119309428513772550.00085796370018833580.00077466558698183760.0001413739216390.000194104280510999960.000176863400952472370.000150330004945445869.692835901762684e-050.000157307428022357060.000170858706500387210.000254146707011632140.000159224038281000030.00.000251494771255490360.00013084919991202729.532814367577266e-050.00.00.00.000159224038281000030.00.000251494771255490360.00013084919991202729.532814367577266e-050.00.00.00.0002917012764858.62426868317e-050.00032032225716240160.000326673019184212030.00022810855310896566.626503378231159e-050.000128926349959999186.353667675290805e-050.0002917012764858.62426868317e-050.00032032225716240160.000326673019184212030.00022810855310896566.626503378231159e-050.000128926349959999186.353667675290805e-056.539900411100001e-054.81201718586e-050.04.0298253073954025e-050.000155898759259120064.216865786147101e-050.00.000102191857714467466.539900411100001e-054.81201718586e-050.04.0298253073954025e-050.000155898759259120064.216865786147101e-050.00.000102191857714467461.30371477041e-050.000353134173971900050.00.03.9111443112375736e-050.000362997637886116970.000334479017298969050.00036192586673177921.30371477041e-050.0003176733893890.00.03.9111443112375736e-050.000362997637886116970.00028419430521312250.000305828225068942930.03.54607845829e-050.00.00.00.05.028471208584653e-055.6097641662836256e-050.0004513740552250.00110127442886999980.00054396722505324260.000444738587400367840.000365416353221920160.00085310364633964850.00119812812298939480.00125259151728219840.0004513740552250.00110127442886999980.00054396722505324260.000444738587400367840.000365416353221920160.00085310364633964850.00119812812298939480.00125259151728219840.00.00.00.00.00.00.00.00.00.00.00.00.00.00.00.00.00.00.00.00.00.00.00.06.87876948139e-050.08.113467888055126e-058.984186179271928e-053.538654376833996e-050.00.00.06.87876948139e-050.08.113467888055126e-058.984186179271928e-053.538654376833996e-050.00.00.06.87876948139e-050.08.113467888055126e-058.984186179271928e-053.538654376833996e-050.00.00.00.0472599439326359460.039273663723921180.046775689635235480.045222635773444290.049781506389177680.040333566255878130.038085264627740820.039402160288231610.045987423582503940.03895449338956920.0456760873337792860.044054259947325240.0482319234663642770.040049157957901780.037784371729716570.039029950481177030.00.00.00.00.00.00.00.01.08642897534e-050.00.00.03.259286926031312e-050.00.00.02.21948719199e-050.00.03.384816557753553e-053.273645018216472e-050.00.00.04.3132844134e-054.73486182142e-054.842688359412934e-054.116178706839589e-053.980986173938245e-054.3072271958502536e-054.678274106558221e-055.2190841618460165e-053.6771357038e-050.00.07.435677663968291e-053.595729447428093e-050.00.00.04.04257994549e-050.000282267833084999960.03.0170943400918458e-059.110645496377578e-050.000291859117959649530.00024422822473678580.00031071615655806940.0001941716340680.0003212539963570.000195469150228371420.000265131799136002970.000121913952838626760.000264677694665785240.00033277729550357140.00036630699890308530.0003232991496287.25150332708e-050.0003796667673779740.00032270841061622380.000267522270888650074.824094459352284e-055.239666999345208e-050.000116907485225350780.0008223297720337.38640925759e-050.000475597425984623030.00085440527370289990.00113698661641241053.1737463548370287e-050.000127941846535086.191296764437084e-054.42656916782e-052.63570836978e-052.6957310947030246e-058.367920486876794e-052.216055921874172e-052.3976612621035205e-052.6042082501715745e-052.9052555970514607e-050.000185598677250999970.0001045437986680.000121422661742179830.00023573965347842320.000199633716532193850.00010799682986659180.000111958696567205339.367586957159516e-059.07598889314e-060.02.7227966679430146e-050.00.00.00.00.02.61553155091e-050.00.02.6740836146521696e-055.172511038063613e-050.00.00.03.58373779155e-052.38580339236e-054.023598636900954e-053.419970438917167e-053.307644298821094e-057.157410177080541e-050.00.00.0004947725383960.0009532598749460.00045792260796355240.00041953661516253910.00060685839206183820.00090329051347782030.00078038581481676970.00117610329654406620.00328581124034000050.006111229689620.00352901450312521470.0029902702679887770.0033381489499051520.006465781555252620.0060371975966048020.0058307099170127620.008914996096340.006153901233610.0088107457694542380.008811450522721850.0091227919968294490.0064846181468342580.0060783429933275180.0058987425606783130.004136373917670.01432662654110.0046738194211082720.00352497776699709380.004210324564907490.0148767976519911160.0134335456090246430.0146695363623497250.00.00.00.00.00.00.00.00.03.89856175547e-050.00.00.00.00.000116956852663955520.05.63379801126e-050.0001560020986180.000102841857600941721.4570544095007394e-055.1601538641885634e-050.000147514610508713340.000160222284848780860.000160269400496701550.000249676987547999970.00.000124780927052357820.00031733634451980320.000306913691070379540.00.00.00.0001285262889342.24476738144e-052.9223119410250457e-050.000150885493254260530.000205470254138748180.00.06.734302144317442e-053.40879550062e-050.00.00.00.000102263865018597120.00.00.00.0002625834364710.0004885231991970.000211956183852716540.00035102437702997520.000224769748529627480.00052522706546471370.00049526327365458990.00044507925847214760.03.35941049498e-050.00.00.00.00.00.000100782314849440310.003141130353190.004609154120570.00302553513882763970.00336379504739290660.00303406087333653970.0041734332378544590.0049740847782953110.0046799443455454680.001761319436230.0001311136582180.0018190134932309640.00168004575894552730.0017848990565200520.000118179468232123140.00012836006112930920.00014680144529390510.002486530145790.000300657062339000030.0017198284742070490.00309494599438239670.0026448159687950440.00017228656156400860.00036969416483006550.00035999046062341980.0192411544372000020.0046769900252399990.019856401685023340.0173332786598105570.0205337829667300820.0052988941097376870.0042681907436174310.0044638852223764570.000123063303075000020.0002313925848356.0734252859849106e-050.00015680285920254960.000151652797164052330.000179432781719810560.000226040512319313080.000288704460465043140.000123063303075000020.0002313925848356.0734252859849106e-050.00015680285920254960.000151652797164052330.000179432781719810560.000226040512319313080.000288704460465043140.0001171855542474.58913095448e-064.281666943198724e-050.000173178637216771580.000135561356092328961.3767392863448298e-050.00.00.0001171855542474.58913095448e-064.281666943198724e-050.000173178637216771580.000135561356092328961.3767392863448298e-050.00.00.00103227149280999988.31886185625e-050.00099605137916435460.00083839432969972890.00126236876955702889.120812339308779e-057.485238570493154e-058.350534658953627e-050.00103227149280999988.31886185625e-050.00099605137916435460.00083839432969972890.00126236876955702889.120812339308779e-057.485238570493154e-058.350534658953627e-050.149930973105886020.078350083687528620.148324345195455680.15155501811461740.149913556007762520.08078738227174360.074527066560015770.079735802230671350.000201757131924000020.0001898960575970.000137811413153565640.000261198125625758030.000206261856993438359.44072111980475e-050.000186508960891086450.000288772000703276160.000201757131924000020.0001898960575970.000137811413153565640.000261198125625758030.000206261856993438359.44072111980475e-050.000186508960891086450.000288772000703276167.16124309953e-054.01631929732e-050.000117908933968314910.09.692835901762684e-050.05.695290216679574e-056.353667675290805e-057.16124309953e-054.01631929732e-050.000117908933968314910.09.692835901762684e-050.05.695290216679574e-056.353667675290805e-050.0001264151144567.19519274737e-050.000128729690566672920.000109417408662824540.000141098244139583340.000122232123125480144.425394425122642e-054.936971504448935e-050.0001264151144567.19519274737e-050.000128729690566672920.000109417408662824540.000141098244139583340.000122232123125480144.425394425122642e-054.936971504448935e-050.0179005698664611060.0022079694212530.018676512455172280.0181052442272315940.01691995291694680.0024436588569528880.00204531049090666980.0021349389158981160.00061402767805699990.00.000373238592601790850.00070193677420347690.00076690766736512430.00.00.00.0138227585834000010.001826751870030.0149144356058853280.014021203027899210.01253263711637950.0020411052521862680.00173011881445178760.00170903154345062560.001823457338940.000181644865992000020.0017732726631850660.00177380812888887550.00192329122475734270.00023688800623620040.000135254783249197180.000172791808489613980.001408847593560.000199572685230999970.00148687741005166050.00125279626760209290.00148686910302048870.00016566559853041930.000179936893205685260.000253115563957876059.32108478961e-050.07.02566186857835e-050.000171728990451522433.764693455087319e-050.00.00.00.000138267824607999960.05.843156476264942e-050.000183771038186416350.000172600870873472780.00.00.00.00020520163055150.00018427148317380.000254263132538609330.00016052591914949930.000200815839965710020.00013316992914155190.00021036908882544540.000209275431554304580.0001220382351049.44064727382e-057.082919010664948e-059.44696752389414e-050.000200815839965710026.483997929236941e-059.18996767909128e-050.000126479762131251625.94761170854e-050.00.000145021683490440823.340666776565464e-050.00.00.00.02.36872783621e-058.98650104356e-053.841225894151902e-053.264957614490326e-050.06.832994984918249e-050.00011846941203453268.279566942305296e-054.32874421416e-050.000114305116377000014.86004566536065e-054.130932035537939e-053.9952549415867686e-058.64533057231592e-054.695042114108609e-050.00020951162226765374.32874421416e-050.000114305116377000014.86004566536065e-054.130932035537939e-053.9952549415867686e-058.64533057231592e-054.695042114108609e-050.00020951162226765370.0521090300893046960.00923306530123080.048243608987381440.054649648312779150.053433832967673120.0089439249625055920.009595192745150730.0091600781960183790.00.00.00.00.00.00.00.00.000184101237950.00.000149321156220316440.000239666105129754540.000163316452498642960.00.00.00.004696657789170.0009996333083350.0044283902190522080.0044794260420856680.0051821571063709460.00092834783718149310.00110126853680917880.00096928355101488990.001064609116720.0008247830390220.00112399746366829440.00087807506339728780.00119175482309089830.00052051225331207530.0010517931143024220.00090204374945177930.00141018981333999980.0005064620510680.00118455732727054260.00156837733232405860.00147763478041991580.00039372911620404370.0006009376239612310.00052471941304017410.0006288290120920.00.00052082793592137560.00080868740559744520.00055697169475572230.00.00.00.000103036733504999990.01.490057956742441e-050.000196537756831274169.76718641160139e-050.00.00.00.0001059695049840.00.000103114276854419898.764486980342852e-050.000127149368293084650.00.00.07.41185182353e-050.05.3807648437921476e-050.000128595356852114663.9952549415867686e-050.00.00.00.006571103496510.001215498302640.0068697829766831790.0069789862352892610.0058645412775629640.00169320061200466330.000884511669193280.00106878262671330830.000526847152656.60772434045e-050.00060825552757581790.00055094076502376370.000421345165350388170.00.000198231730213356140.00.00155128004690.00052124857812900010.00165092784416595260.00148081563352230440.00152209666301809540.00058279554931123870.00061127725420384650.000369672930873382370.003354891022770.0003170075028750.00294979304593770850.0038773825143758110.00323749750800877330.000183837760553767520.0003420358541387470.000425148893932318173.365055106800001e-050.00.07.130601148307617e-052.964564172081672e-050.00.00.00.0009803227078823.16541631191e-050.00090889908466133590.00110808823437949660.00092398080460455989.496248935732841e-050.00.00.0003276511299681.1282659344000001e-050.00020660580175484970.00050984478758739070.00026650280056105470.03.384797803194579e-050.00.00167630658835999984.75969785993e-050.0020712802986185920.00136373897605777150.00159390049039516494.922545366686005e-059.356548213116442e-050.08.45804531127e-050.02.5392373420142725e-050.00020747490111072572.0874084807166827e-050.00.00.00.000128593779102999988.52020863382e-056.487812156151297e-058.707101772211672e-050.000233832198023895030.000119645199884729257.797123510930369e-055.798982402051129e-053.75999236913e-050.02.7785916816303718e-054.168282234774268e-054.333103190980968e-050.00.00.00.000383162125126999953.86473174297e-050.00041761678110206160.000412887576422927370.000318982017856683245.55771251077452e-056.03648271813964e-050.00.00517064957600000060.00195762489743999970.0052587873229739420.00520412374858798650.0050490376564379220.0019879955026229410.00194795249193607650.00193692669775331330.0002912571516570.0002227538500730.000320670323913827840.000299004740501634630.000254096390556984640.000231984178285892820.000251968499700151360.00018430887223364237.83863724254e-050.07.964557117622878e-058.896137745113445e-056.655216864895604e-050.00.00.00.000277618792044000035.2607098387e-050.00022515268307521010.000262107921882000240.000345595771173771240.00.000157821295161000260.00.0141420335401000010.001545914046060.0128716464684688280.0143425536553461620.0152119004963911370.00151924779417529110.00133795671557285350.00178053762842549650.008225583953940.00078907217896700010.0061075722384834450.0093756674616668220.0091935121616838910.00058286409083752130.00084368843750477560.00094066400855956320.00021937253340397.89288600898e-050.000204416543895755220.000173749571077386150.00027995148523847737.055719983017099e-055.900525900163522e-050.000107224121437512260.0001319792307651.37992782372e-050.000125087891202548080.000106321959217258780.000164527841874938530.00.04.139783471152648e-050.00.00.00.00.00.00.00.00.00.00.00.00.00.00.00.01.62546384354e-055.4106039248e-061.8249700412323305e-051.55118443864749e-051.5002370507438879e-051.623181177440203e-050.00.07.11386642035e-055.97189779278e-056.107895228088384e-055.191576747365248e-050.000100421272856099885.4325388055768955e-055.900525900163522e-056.582628672598578e-053.01570191764e-050.00.01.6631025078139754e-057.38400324511286e-050.00.00.01.92517466097e-050.00.00.05.7755239829155896e-050.00.00.01.09052725667e-050.00.01.6631025078139754e-051.6084792621972712e-050.00.00.00.001166096551563030.000190427101602930.00148292679211540260.00105294115917790170.00096242170339569880.000146518764482561780.000179672261975214520.000245090278352240670.0002575441527730.0001421117209250.0003552714604285470.000225177196012413190.000192183801878368050.000146518764482561780.000159140651476212930.000120675746817325520.000228836890199999986.84387016633e-060.000219409828581815730.000201860600815418810.00026524024120281010.02.0531610499001603e-050.09.33564596903e-060.00.00.02.800693790710323e-050.00.00.00.00.00.00.00.00.00.00.00.00.00.00.00.00.00.00.00.00.00.00.00.00.00.00.02.6665737279e-050.07.999721183690982e-050.00.00.00.00.00.00.00.00.00.00.00.00.00.0006437141253424.14715105116e-050.00082824829126812990.00062590336235006970.000476990722407417360.00.00.000124414531534915140.00895601956705870.00233061714559061040.0081551858145719380.0102154335442538060.008497439342364490.00294538284008241630.00183142310206951470.0022150454946067910.0007367224407220.000104679734406000010.00052550734273874830.00099151272772700690.0006931472516989210.000200926537294290375.346598978923682e-055.9646676135383045e-056.64676291231e-051.01261428618e-050.08.709294996839434e-050.000112309937400776683.0378428585341834e-050.00.00.001369664636170.0003906501697540.00134752232764010960.00139702539027913460.00136444619059118940.00044945425288414730.000352032204319198670.00037046405205780355.2990760272e-050.08.6366416600995e-054.89397043700673e-052.3666159845068124e-050.00.00.00.0009800606020150.00078254461831100010.00086550174934188580.001098268864438230.00097641119226457250.00076662869342843320.0007277954366644130.0008532097248395911.37106534896e-050.00.00.04.113196046873463e-050.00.00.05.46221615308e-051.59055884326e-052.50638214535235e-050.000118198668153738822.060399498526264e-052.1574662161682846e-050.02.6142103136259117e-050.000162529178910.00.0001835497627298890.000101693826874860440.000202343947125722940.00.00.00.000169465221642000058.27176690561e-060.000109645774175387180.000248523997394088440.000150225893356160192.4815300716832737e-050.00.00.000144268453271.68741808496e-050.00.000208614521258457650.000224190838552684872.426606870901551e-052.635647383976463e-050.04.6810838594999994e-050.05.2556307776574466e-054.467170689593353e-054.3204501112508085e-050.00.00.01.99993029592e-050.05.999790887768237e-050.00.00.00.00.00.0051387076883599990.001001564944070.00489947440323714250.0058708911868938940.004645757474962890.00142733889630267250.00067177299745690160.00090558293843775430.00013890168752110.02.0575914121936592e-050.00026378390238266690.000132345246058706340.00.00.06.82386528566e-050.00.00.000157880827111655484.6835131458097e-050.00.00.07.06630346645e-050.02.0575914121936592e-050.000105903075271011438.551011460060933e-050.00.00.00.00725603381470000040.0006023846803520.00661227893419094260.0079627032974243960.00719311921249059850.00065428569378489280.00081870299996845680.00033416534730128310.00725603381470000040.0006023846803520.00661227893419094260.0079627032974243960.00719311921249059850.00065428569378489280.00081870299996845680.00033416534730128310.00.000246660820065999970.00.00.00.000354712827893550240.000385269632304794660.00.00.000246660820065999970.00.00.00.000354712827893550240.000385269632304794660.01.64178068079e-050.00.04.925342042372159e-050.00.00.00.01.64178068079e-050.00.04.925342042372159e-050.00.00.00.00.00.00.00.00.00.00.00.00.00.00.00.00.00.00.00.00.00579151537102670.007237939952425550.005497347425648670.0056493033192482110.00622789536818252240.0074880403546999470.0062972158136412630.0079285636889447670.000178208540975999960.000441580423269999950.000121883392416685080.000195609875861042160.000217132354650423620.00048649304223121220.000475434680951656340.00036281354662814320.00.00.00.00.00.00.00.00.00.00.00.00.00.00.00.04.09305213714e-050.00.02.9476471557930572e-059.331509255629944e-050.00.00.07.82640946479e-050.03.053947614044192e-050.000103831534947304960.000100421272856099880.00.00.00.0001411300969440.06.191565025733431e-050.000157880827111655480.000203593813461681950.00.00.00.000139839698150999980.000317981848866999970.000237414181472251269.257269380844057e-058.953221917290832e-050.00026976331715529450.000179096325380567140.00050508590406593775.78262156003e-050.03.820689969763663e-050.000122503406568171751.2768340534968026e-050.00.00.06.70955353195e-050.07.533070781309008e-056.402944655083806e-056.192645159459492e-050.00.00.00.0001342157908934.0607907521e-059.294623173177979e-050.00014861114019420590.000161090000753435263.0532243413622055e-055.0628006027428384e-054.066347312186114e-059.98382430899e-050.00.00.00013559176916648060.00016392296010333950.00.00.08.67862625507e-052.50994899446e-055.003515648101924e-050.000128059710233562858.226392093746926e-050.04.8336411433073875e-052.696205840068053e-050.00.00.00.00.00.00.00.00.00142983704739000010.00205879812660.00131663686554263550.00140943104739689610.00156344322923417730.00172918397047838860.0022287219109497070.002218488498380610.08.80320396395e-060.00.00.00.02.640961189186093e-050.00.00.00.00.00.00.00.00.00.000164802840393999970.000326998104536000030.00023670899105307090.000139418956199405450.00011828057392855570.0003564257814638120.000347752176374877860.000276816355768162260.000114027554204999990.0002981744291850.00.000222865964357334020.000119216698256974160.000320154375260002450.000258824061072625850.00031554485122322580.0001160935051920.000131544887643999989.4491480183667e-057.325402783358593e-050.00018053500755726560.000195329815371960655.550494702696195e-050.000143799900532053345.69679073468e-056.09023709905e-056.396003493564252e-055.436462442995684e-055.257906267465607e-050.000113775812720572730.06.893130025079644e-050.0006240653856980.0009438262066530.00083402354808270270.00044158428174176640.00059658832727000050.00116393127665794520.00067453117223995530.00099301617106136690.0001530406795270.000213594641889000020.00022412441993977210.00014287562453492799.212199410766188e-050.00024626973515843170.000213355897286582960.000181158293221101430.00.00.00.00.00.00.00.00.000231247780140.0006132652276638.535549072312903e-050.00036682741426634450.00024156043542920690.00067974752215458930.00047571142308303360.00068433673775043041.29628711642e-050.00.01.9768954338166124e-051.911965915442039e-050.00.00.00.01.68204130382e-050.00.00.05.046123911456363e-050.00.00.00.00.00.00.00.00.00.00.00092758933143800010.00.00118575250457655910.00060512011330694250.0009918953764300450.00.00.00.00.00.00.00.00.00.00.00.000261505145092000030.0005173950612550.00025354277256340750.000234270103422929580.000296702559288388860.00049452271903804780.000478141723846292650.00057952074088103310.01.23633127353e-050.00.00.00.00.03.70899382060123e-050.0006752403238960.001210184296670.0004944796220378440.00076135533142032240.000769886018229950.00135144950448150450.00078476746607663870.00149433591945335160.00284836317685700040.0042520992887340.00244016490279216470.00298939592139377740.00311552870638178140.00369341883964176360.00409548737293901660.00496739165361887150.0005593359175060.001233757024430.0004423915433591490.000566961988450570.00066865422070749370.0011349679933559850.00112698724647598470.00143931583345510180.000306579554107999960.0002641411937040.00027609492410492080.0003076851025041720.000335958635716013330.000228411431214947650.000330737486865217470.000233274663031185730.00117514236820000010.001268406578350.00102482976332860380.00135018623061773930.00115041111064952430.0009320681978905180.00120901598508096440.00166413555207028310.00080730533704300010.00148579449224999980.0006968486719994910.00076456259982129640.000960504739308750.00139797121718031260.00142874665451684980.00163066560506230059.39001546287e-050.000106983595056000017.223088683816766e-050.000126189460227967878.328011681995047e-050.000172941698015199543.898561755465185e-050.000109023469599100289.39001546287e-050.000106983595056000017.223088683816766e-050.000126189460227967878.328011681995047e-050.000172941698015199543.898561755465185e-050.000109023469599100280.0002006591946032.16928491142e-050.00013945134285816250.000222362112492571770.00024016412845705813.5575917841831e-052.950262950081761e-050.00.0002006591946032.16928491142e-050.00013945134285816250.000222362112492571770.00024016412845705813.5575917841831e-052.950262950081761e-050.00.001441326068320.00130985673970000010.001396982783836090.001637127812404660.00128986760873161880.0014995871469230690.00127177980610189360.0011582032660848880.001441326068320.00130985673970000010.001396982783836090.001637127812404660.00128986760873161880.0014995871469230690.00127177980610189360.0011582032660848882.58276712502e-052.4950795235e-057.748301375060695e-050.00.00.07.485238570493154e-050.02.58276712502e-052.4950795235e-057.748301375060695e-050.00.00.07.485238570493154e-050.00.00018080350991580.0001800129790510.000176708331814063530.00018590403213199420.000179798165800501960.000130734267191118780.00021129063672156290.000198014033240770320.00.00.00.00.00.00.00.06.04588311098e-053.4931113329e-050.09.22024030332068e-058.91740902962167e-050.00.000104793339986904160.00.000120344678806000010.0001450818657220.000176708331814063539.370162909878742e-059.062407550428528e-050.000130734267191118780.000106497296734658720.000198014033240770320.00029623959353210.000138584979952999990.000294184207100488360.00032051271856275160.00027402185493284690.000142724688146517220.000273030251711708560.07.75472149344e-050.000138584979952999993.857618038792664e-057.447172812057582e-050.000119593736294696660.000142724688146517220.000273030251711708560.01.47316839936e-050.00.02.2466472473978264e-052.172857950687541e-050.00.00.01.45801369961e-050.04.3740410988245855e-050.00.00.00.00.00.00.00.00.00.00.00.00.00.000189380557607999980.00.000211867615724315850.00022357451796819750.000132699539131274820.00.00.00.0223726610530241980.036286866658430.0231217194455118350.0215365315466236260.0224597321670790550.037672800259283130.033429738513812490.03775806120205989.66175708601e-050.000106060904799999990.000108476219250849729.22024030332068e-058.91740902962167e-059.648188918704568e-050.000104793339986904160.000116907485225350780.0001987146719380.0001951503209580.000212552848599914320.00020885971874822160.000174731448465543420.000279290959672016849.948734808883306e-050.00020667265511217390.00177047639323000020.002329111721260.00140142247126942570.00192356455971355460.00198644214871187150.0025094577711537890.00227549556176778870.00220238183086745360.003851113260480.008147531506760.00335798709677966050.0040759396722224220.0041194130124303850.0087979642004578830.0066956552373301310.0089489750824869340.00090534534043100010.00137248229476999980.00091570384958797440.00093159141808043510.00086874075362508730.00112266783508582960.00141377679094304740.00158100225828609450.00084341147684800010.00084960690621800010.00092617387240997930.00077454984166792280.00082951071646659740.00087582217860754530.0008617664711774270.0008112320688681341.04966305821e-050.00.03.14898917463138e-050.00.00.00.00.04.38841911507e-050.00.00.00.00.00.000131652573451971570.000207652537140.0001793447987870.000218702054941229250.00023571072162531850.00016854483485283790.000143453062833539240.00026789880715955380.000126682526368933880.0007095084635720.00073178661267499990.00066785965165829140.00080481522008279370.00065585051897506780.00089290173460717030.00069683412212152940.00060562398129515953.41977940258e-050.00.08.392209013944795e-051.867129193806882e-050.00.00.01.31567355926e-050.00.03.947020677791387e-050.00.00.00.00.08.21752878097e-050.00.00.07.475352984533497e-058.119318697332451e-059.057914661055071e-050.01.62371507257e-050.00.00.00.00.04.871145217722949e-050.02.56598177329e-050.00.00.00.03.638657638434173e-054.059287681435791e-056.01606864956e-050.000330082624082999950.07.364409187955815e-050.000106837967607288350.000385311059053696770.00037149534908683840.00023344146410812850.0002525593185290.00.000103600596134023790.00037659994071355040.000277477418739446260.00.00.00.00.00.00.00.00.00.00.00.01341925017330.02187775252070.0152092407848804880.0118841717701929690.0131643379649706430.022494696038779280.0205249557227927750.022613605800387330.00.00.00.00.00.00.00.00.00.00.00.00.00.00.00.00.00106708382999650.00085863019591509990.00133215708906780760.00065869024686501050.00121040415405172320.00107903790155444430.00067604520085423420.00082080748533809835.80634440265e-054.99832078861e-056.51900356074818e-055.5410097976686795e-055.359019849532254e-058.697285683928397e-056.297676681905299e-050.00.00100902038597000010.00080864698802899990.0012669670534603260.00060328014888832370.00115681395555640070.00099206504471516030.00061306843403518120.00082080748533809830.002856466672566590.004511279291784940.0028627009764560580.00300449341152751130.0027022056297177430.0046193999596933150.00465121163203120050.0042632262836246977.66100432098e-060.00.00.02.298301296294245e-050.00.00.00.00.00.00.00.00.00.00.00.0002341198978340.00.000425211660826501530.000160631364169349840.000116516668505509620.00.00.00.000188837364725999970.000196552522157000020.00023309282232773379.115525055645124e-050.000242264021292673280.0003342418692227480.00012072965436279280.000134686042886348810.000393518770454999977.12450114434e-050.00033318845713069780.000486382480851793760.00036098537338399526.737562094067435e-050.000146359413389530950.03.67956624854e-050.04.741093498725949e-050.06.297605246907958e-050.00.00.00.000383990365939000040.00.000324161218770897340.000368895881470258060.00045891399757575060.00.00.00.000178365952169999980.00025428177270.00018775904844966850.000207713405717577830.000139625402342719250.000286215423979972860.00027738407353947170.000199245820580060930.00.00.00.00.00.00.00.00.000153007036312999980.0001067254391340.000133907670544372740.000165270811714013820.000159842626680853820.00013460084987032048.771763949796665e-059.785782803461281e-057.8169555712e-050.08.776393143272631e-057.459741345728706e-057.214732224613001e-050.00.00.00.01.7234205940000003e-050.00.00.00.02.443874533276683e-052.726387248725531e-050.00.00.00.00.00.00.00.00.09.03225768719e-050.00.00.09.229772562536256e-056.683248723654602e-050.000111837517753843180.00.00.00.00.00.00.00.00.00025820474980.000131263496038999980.00020780884913955880.000353265912004623740.000213539488257223830.00011940827869683870.000129694727706564540.000144687481714543045.56552827535e-056.10949912441e-056.248630141177978e-055.311198331405922e-055.136756353468703e-055.55771251077452e-056.03648271813964e-056.734302144317442e-055.50105872489e-050.08.920741714708035e-057.582434459967665e-050.00.00.00.01.000974025e-050.00.01.5265298515431592e-051.4763922234472961e-050.00.00.00.000341554665184000050.000110147994819999980.000241518299858762120.00052787635324355040.0002552693424510020.000122750495148913080.000133324860034229267.436862927821297e-050.02.49663091455e-050.00.00.00.03.540315540098114e-053.9495772035591476e-050.0002664797230820.00289078143080.000242692955456541550.000178296850806954450.00037844936298250710.002986984003460650.0029808722269802850.0027044880619506010.00.00.00.00.00.00.00.01.88326769533e-050.000222403178540999975.649803085981756e-050.00.00.00015075295185475890.000272899322882562870.000243557260886147479.75219710151e-060.00.00.02.9256591304533033e-050.00.00.00.06.69178416264e-060.00.00.00.02.0075352487912675e-050.00.0001865014402380.000292862785330999950.00018999337811266080.00024620606110648350.000123304881493662460.000237624316967580630.000260824131604573370.00038013990741941320.03.47057934554e-050.00.00.03.1571298817750546e-053.429101439362047e-053.8255067154892275e-050.02431525412410.007930545254350.0268303957214510970.022162668299517090.0239526983514564870.0082578175240329530.0080183148907893540.0075155033482233970.02431525412410.007930545254350.0268303957214510970.022162668299517090.0239526983514564870.0082578175240329530.0080183148907893540.0075155033482233970.00245803068686150.00662682153870840.00196679984221288450.0023297709800261220.0030775212383473890.00646017239340196650.0070583556386128890.0063619365840958380.00203480101172070.00604883055365340.001486083100422750.00197753074838948220.00264078918635182130.0060031218792212790.0065138914638643040.0056294783178612130.0002209906895240.00053244279584599990.00010847788814143650.000193007422617358780.00036148675781417310.00058865583950559160.00053291309434682970.000475759453684146261.43886396743e-052.21278835943e-050.04.3165919023036895e-050.03.182120355773274e-053.4562447225232235e-050.00.0001895381366630.00090859939701899990.000221480483951371920.000205523528563013960.00014161039747584320.00064738648918867580.00108497702370105370.0009934346781666090.0001982436898510.000243735667650.000144250291556981010.00021331564422983280.000237165133766533750.00025636533622213720.000209029268590899230.00026581239813657240.00.00.00.00.00.00.00.00.00.00.00.00.00.00.00.00.01.01266787401e-050.00.00.01.3736032059659124e-050.01.6644004160784563e-050.000117138742712000020.0001460321928130.000113468848588754930.00014466904241611959.327833712993376e-050.000151383717343690930.00016442469663217180.000122288164461663980.0002154208625120.00087327237647899990.00017246507293747720.0002020713505941780.000271726164004564560.00082596753732797870.0009220610684375260.00087178852367229518.02901931504e-050.0002545146622180.06.438715295615e-050.000176483426495053420.00024003059206160870.000219539120084296420.000303974274507776140.000157052320264999990.0006008174988945.480032622880976e-050.00023369307609023880.00018266355847713850.00048209045851134590.00079339547672848720.00052696656144227840.00084173773736899990.00245716140040.00067114018901791870.00067769761189955330.00117637541118858140.0027656846734428590.0025529892681178070.00205281025962908770.0004002939523280.00057799098505499990.000480716741790134640.00035224023163663970.00036792488355712880.000457050514180687040.00054446417474858510.00073245826623462440.0004002939523280.00057799098505499990.000480716741790134640.00035224023163663970.00036792488355712880.000457050514180687040.00054446417474858510.00073245826623462442.29357228128e-050.00.00.06.88071684384388e-050.00.00.02.29357228128e-050.00.00.06.88071684384388e-050.00.00.00.00087936350346340010.00255993699389209980.00072509451156722390.00106580819177440270.00084718780704995840.00299553413090073530.0025185681801980230.00216570867057340349.06588630448e-050.000329011117870000042.717340161594432e-050.000154909951332672728.989323618570229e-050.00029296120198495650.00047729760115492780.000216774550468733849.06588630448e-050.000329011117870000042.717340161594432e-050.000154909951332672728.989323618570229e-050.00029296120198495650.00047729760115492780.000216774550468733841.03210752658e-050.00.00.03.096322579729746e-050.00.00.01.03210752658e-050.00.00.03.096322579729746e-050.00.00.01.16065462026e-050.00.03.48196386077065e-050.00.00.00.01.16065462026e-050.00.03.48196386077065e-050.00.00.00.00.00.00.00.00.00.00.00.00.00.00.00.00.00.00.00.00.0006704747457110.00214332621304310.00055217363482171340.00079375502769723170.00066549557461590830.00249069183424074350.0020412705790430950.00189801622584275720.0001114481455490.0001330600614327.533070781309008e-050.000147546115964974680.000111467612870270846.700131193544838e-050.000250993118732806178.118575362871582e-050.00.00.00.00.00.00.00.00.000176694931169000030.00.000135257131235473430.000172448384725449130.000222379277546675050.00.00.00.000267074454920999970.001975610300610.000153259026240424650.00039372371881826040.00025424061970571890.0023197229693020130.0017902774603102890.00181683047221404150.0001152572140723.46558510011e-050.00018832676953272528.003680818854757e-057.740806449324365e-050.000103967553003281980.00.00.00.00.00.00.00.00.00.00.00.00.00.00.00.00.00.04.4946571443400004e-058.7599662979e-057.400394387900434e-050.06.0835770451050204e-050.000211881094675035420.05.091789426191236e-050.01.69726314206e-050.00.00.00.00.05.091789426191236e-050.04.23166180645e-050.00.00.00.000126949854193481150.00.01.88023506038e-050.03.0957825128667156e-050.02.5449226682710237e-050.00.00.02.61442208396e-052.83104134939e-054.304611875033719e-050.03.538654376833996e-058.493124048155428e-050.00.05.13557017958e-050.07.174353125056198e-058.232357413679178e-050.00.00.00.05.13557017958e-050.07.174353125056198e-058.232357413679178e-050.00.00.00.00.0834709229299770.152917843789945330.078759665393083880.086257971098861980.085395132298130550.15237340159370720.156399988582170880.149980141193828830.0001197087993160.0001621525494359.13099488643516e-050.000155222900729304370.000112593548353808960.000162427422873814260.000176419764287717430.000147610461143119680.0001197087993160.0001621525494359.13099488643516e-050.000155222900729304370.000112593548353808960.000162427422873814260.000176419764287717430.000147610461143119680.00.00.00.00.00.00.00.00.0001408508492480.00080092851590400010.00024309846601308560.00011764209677411846.18119849557879e-050.00078587888014094720.0009343799712458550.00068252669632502370.00.000214906074514000040.00.00.00.00030003036309673950.0002703192311677687.436862927821297e-050.0001408508492480.000586022441390.00024309846601308560.00011764209677411846.18119849557879e-050.000485848517044207730.0006640607400780870.00060815806704681070.000189376743452999980.0002575693796330.000183857998730253720.000195344074222895750.000188928157407238760.00036793940791669960.00035523166097255654.953707001074185e-050.000189376743452999980.0002575693796330.000183857998730253720.000195344074222895750.000188928157407238760.00036793940791669960.00035523166097255654.953707001074185e-054.07026917097e-050.03.75610177461391e-052.881325094787713e-055.573380643513542e-050.00.00.00.00.00.00.00.00.00.00.01.25203392487e-050.03.75610177461391e-050.00.00.00.00.00.00.00.00.00.00.00.00.00.00.00.00.00.00.00.00.00.00.00.00.00.00.00.00.02.8182352461e-050.00.02.881325094787713e-055.573380643513542e-050.00.00.00.000183370186317999988.729650927819999e-060.000205447384944791170.00020955091598456090.000135112258024570730.00.02.618895278345672e-050.000183370186317999988.729650927819999e-060.000205447384944791170.00020955091598456090.000135112258024570730.00.02.618895278345672e-050.000190835922876016.044171314637e-050.000103695570695464160.000204526198836445830.000264285999095793039.496248935732841e-053.944953402012464e-054.691311606153723e-050.06.14119432647e-060.00.00.00.01.8423582979413533e-050.00.00.00.00.00.00.00.00.09.41609508101e-060.00.02.8248285243016796e-050.00.00.00.00.0001814198277955.43005188199e-050.000103695570695464160.000176277913593429040.000264285999095793039.496248935732841e-052.1025951040711108e-054.691311606153723e-050.00.00.00.00.00.00.00.00.00.00.00.00.00.00.00.00.069258303241650.141752216668360020.065244947315359820.072079684670433870.07045027773930990.141493223167445780.145099169345239530.138664257492287030.02130288175580.04577586822020.0201708993231229030.022169059772288160.021568686172014440.04598721562138710.0469414137650595860.044398975274063080.003714500039150.00596938742456000050.003945262936321260.0039448165409528620.0032534206401642750.006741591922098690.006084457989040550.0050821123625404690.044240921446699990.090006961023600010.041128785055915650.045965808357192850.045628170927131190.088764415623960010.09207329759113940.089183169855683480.03.0975731542e-050.00.00.02.8178121842011e-053.0605531538231354e-053.41435412457216e-050.03.0975731542e-050.00.00.02.8178121842011e-053.0605531538231354e-053.41435412457216e-050.00079472644886999990.00133760799944550.00067628565617736730.00087096850036140210.00083692519007225970.00124084848061898770.00116739347267903880.00160458204502336070.0007438582822050.001093938129690.00067628565617736730.00080475912871876260.00075053006172002820.0010225636634537260.00094732134028061760.00131192938532189153.40555553723e-054.55118696825e-050.06.620937164263956e-053.595729447428093e-050.04.2255379026977475e-059.428023002044418e-051.68126112927e-050.000198158000072999960.00.05.043783387795062e-050.000218284817165261690.000177816753371443730.000198372429681025070.00.00.00.00.00.00.00.00.00.00.00.00.00.00.00.01.87403258198e-056.22090587725e-050.05.622097745927245e-050.05.883042023600346e-050.000127796756081590440.00.00.00.00.00.00.00.00.01.87403258198e-056.22090587725e-050.05.622097745927245e-050.05.883042023600346e-050.000127796756081590440.00.09.35368359762e-050.00.00.00.0001067277535254930.000173882754403048940.00.09.35368359762e-050.00.00.00.0001067277535254930.000173882754403048940.00.01.1939081415999998e-050.00.00.00.00.03.581724424796286e-050.01.1939081415999998e-050.00.00.00.00.03.581724424796286e-050.00012701517502451.6962357451999997e-059.970240739967804e-050.00015840089759543290.000122942220077504635.088707235603674e-050.00.00.00.00.00.00.00.00.00.00.0001024631610690.09.970240739967804e-058.474485572905038e-050.000122942220077504630.00.00.01.0453787192e-051.6962357451999997e-050.03.136136157592068e-050.05.088707235603674e-050.00.00.00.00.00.00.00.00.00.00.00.00.00.00.00.00.00.01.40982267635e-050.00.04.229468029046184e-050.00.00.00.00.0095609243591655980.00304158833620740.0092632743347011780.0090794596445747750.0103400390982155180.00318399076525173330.00285380683591154130.00308696740744992220.00.00.00.00.00.00.00.00.00.00.00.00.00.00.00.00.00061377872319.43858808161e-050.00071993581886300270.0005751384710318510.00054626187940459184.061023466730512e-050.000158014415434567558.453299234654143e-050.0002559477880271.33938317979e-050.000233282191937311230.000305000481957217830.000229560690185339750.04.01814953937516e-050.01.39700610656e-050.00.04.191018319691218e-050.00.00.00.00.000154835850737000030.0001699693987180.000173840094953284820.000147760261271164760.000142907195987526750.000154618412158727050.000167938044850807950.000187351739143190350.0003847942630180.0003991537614340.000381977932520953660.00043425494319414680.00033814991333778770.00030923682431745410.00041984511212701980.000468379347857975760.00.00.00.00.00.00.00.00.001598861052945.77729529851e-050.00127799347384209040.00129405873122158550.00222453095376188264.088215643518885e-054.440395762156956e-058.803274489860752e-051.2891834875999997e-050.00.03.8675504628022994e-050.00.00.00.03.0882664051e-050.00.06.024461631847185e-053.2403375834381074e-050.00.00.00.0001751578118740.00.000168057815719865970.000101060212670294720.000256355407233115930.00.00.00.000179146421084000030.00.000327000300288134240.000115707280636791249.473168232600924e-050.00.00.00.000164101117025000030.00.00.00032257442398692220.000169728927088972120.00.00.00.0003019833311710.00.00040146208583504770.000315559750269284070.000188928157407238760.00.00.00.00.00.00.00.00.00.00.00.000301229713904000070.000252074328010999950.000317364863084784140.000325430940411002960.00026089333821751260.00029131005189325390.000253124009630203340.000211788922509693440.0001498545889771.81554643082e-050.000321445765838669246.512854756270517e-056.29894535300391e-050.05.446639292458637e-050.00.00.00.00.00.00.00.00.00.004855736197710.001991765637510.00475815833163350.0046231056250656980.0051859446364208060.0022125818438984560.00171583340792903510.00204688166069391360.0003677529396064.49170806271e-050.000182755660184533830.0003138496711527040.00060665348748031420.00013475124188134870.00.00.07.01036722538e-050.00.00.00.000167503279838620944.280773692275497e-050.00.07.01036722538e-050.00.00.00.000167503279838620944.280773692275497e-050.00.001653918801220.00261627654640.0016781565953062870.00159173231710487040.00169186749126114170.0022897580420220210.002542438976281730.00301663262090264570.00.00.00.00.00.00.00.00.00.00.00.00.00.00.00.00.001015104171220.001610303103530.00106971626296979030.00092681114138462890.00104878510931727120.00151666598122838580.00170274875202769040.00161149457732871820.000638814630.001005973442870.00060844033233649680.00066492117572024150.00064308238194387040.00077309206079363530.00083969022425403960.00140513804357392750.00014299284347640.000263986018983700053.75610177461391e-050.000191193518194505570.000200223994488330850.00019069875455171940.00031598388287281160.00028527541952757283.31053093079e-057.08664683361e-053.75610177461391e-050.06.175491017743538e-050.00.000108857347631825660.000103742057376407029.38801725308e-050.000164690845962000020.00.000143171433281377020.000138469084310895480.000149816598116530520.00016272257761941640.000181533362151165821.60073616377e-050.00.04.802208491312855e-050.00.00.00.00.00.00.00.00.00.00.00.00.02.84287046856e-050.00.00.04.088215643518885e-054.440395762156956e-050.00.001049456541830.002330619674090.00089476767939933710.00131921113564265690.00093439081043355370.00215154753572995540.0025406223597143390.0022996891268207440.001049456541830.002330619674090.00089476767939933710.00131921113564265690.00093439081043355370.00215154753572995540.0025406223597143390.0022996891268207440.0095271050848359590.012792878008885940.0097209169031417070.0091721747005849580.0096882236508006260.0125555426585754820.0131376974022914170.0126853939657922290.000103904424569999990.0003047727149420.00031171327370933820.00.00.00027724680800875190.000301130287318690150.000335941049498134350.000103904424569999990.0003047727149420.00031171327370933820.00.00.00027724680800875190.000301130287318690150.000335941049498134350.00164922549399763040.0054196759791133990.00160434495424880430.00153029370100661650.0018130378267480420.0047885327867003140.0054149618913752550.0060555332592645960.00135081512688000020.00366576203121000020.00146327651654220950.00106751306631327560.00152165579779485880.0031571369192883160.00361808924907686780.00422205992526127152.59831265432e-057.20550996664e-050.00.07.794937962956003e-058.4337315722942e-052.963612556190729e-050.000102191857714467460.00.00.00.00.00.00.00.01.22485476316e-057.8053982467e-050.01.8679579220665885e-051.8066063674274044e-057.81862959376383e-058.492166935729672e-057.105398210616945e-057.78883847668e-050.000101787997663999990.00.00018816816945552414.549698484500852e-055.457120429131543e-050.000118544502247629160.000132248286454016719.33564596903e-060.0001210032843770.00.02.800693790710323e-050.000121208403501313670.000131649924606663520.000110151525023885770.03.51964931947e-050.00.00.02.346349445210255e-052.5484761669966964e-055.664122346189476e-051.7703994438e-050.000296296033996999970.05.311198331405922e-050.00.00026176936965070340.00039240945820953830.00023470927413219660.06.97350896773e-050.00.00.09.459008743828007e-050.00.000114615181593481156.07775737885e-050.0003121893814077.603473311975445e-056.462785259336925e-054.167013565243771e-050.0002732414324009490.00029202691112981250.00037129980069127889.44730939805e-050.0006675965854536.503370458684034e-050.000138193050109722448.019252724479919e-050.00064002826401675370.00072219928951557370.00064056220282593319.44744400011e-050.000204873816000999980.000107189939971195350.00.000176233380032048780.000286013505495194640.000155326096818533830.000173281845689749179.44744400011e-050.000204873816000999980.000107189939971195350.00.000176233380032048780.000286013505495194640.000155326096818533830.000173281845689749170.00.00.00.00.00.00.00.00.00.00.00.00.00.00.00.00.000249985075717130030.00028947733890670.00030758282014274020.00024545407587137770.000196918331136562640.000203702245503605260.000335655605603039260.000329074165614291436.28786751096e-058.32819955544e-050.00012326332428750960.06.537270104121194e-053.890398757542165e-059.970786805488549e-050.000111234131032892468.10007610893e-060.02.430022832680325e-050.00.00.00.00.00.0001138916330910.000187211042630000010.000160019267528427385.0110001648481956e-050.00013154563009535070.00016479825792818360.000178994835381358030.000217840034581398966.51146914076e-050.00.00.000195344074222895750.00.00.00.00.01.89843007223e-050.00.00.00.05.695290216679574e-050.00.0069568785054911010.0059786701157428410.0070767638601422840.0069059633342551450.0068879083220860560.006553156558637520.0059575992419269770.0054252545466649870.0007489154340730.000155484790129000020.00053992833807999130.00076151401660496350.00094530394753506940.000148891804300996420.00018764575019461250.00012991681589136290.0004591180721450.0001889692745420.000433792706999844960.00056629084802259280.00037727066141123780.000206647138152951360.000151517400122946620.00020874328534970890.000235186530010.000287120848102999970.00021437679870920770.000243481982045028420.00024770080927662710.00026864615307716650.000291788727322820050.00030092766390875136.78050297252e-050.08.829472284508295e-052.162345286895094e-059.349691346152704e-050.00.00.00.00086341119116699990.00071944931795000010.00087768052540585190.0010434554564391790.00066909759165678920.00066391991345941080.00071735928439657130.00077706875599459030.0002367550327440.0005605955374750.00033861454152687570.000151648689199353320.000220001867507113590.00065115320125707780.00054537928453134540.000485254126637477540.000123561820397000010.07.77050281166545e-050.00015899353744230190.000133986895631891760.00.00.09.03825908086e-050.000154721946557000029.13099488643516e-050.000104775457992280447.506236556920596e-050.000243641134310721380.000220524705359646820.03.14898917463e-050.000384980589140999970.09.44696752389414e-050.00.00049426923848245650.000335044520495375370.00032562800844480560.00.00.00.00.00.00.00.00.000464249383775999937.856685835039999e-060.0004242303595159740.00056188266492890920.00040663512688222090.00.02.3570057505111045e-050.002173077803670.001180669775780.0024280828963951940.00187694199953148560.00221420851509061480.00147884418447830350.00140421935177684740.00065894579108749510.000235902732924000020.00.000117908933968314910.000250550008242409840.000339249256561693950.00.00.01.56452006386e-050.00.01.6727576747679032e-053.0208025168095085e-050.00.00.00.07.54474845004e-050.00.00.00.07.004902405541721e-050.000156293429445656120.00.0002307821427770.00.00.00.000177356413946775120.000192634816152397360.000322355198231665764.19349828779e-054.99345126176e-050.02.9401276477425642e-059.640367215634426e-054.542461826132094e-054.933773069063284e-055.504118890082428e-050.00.000166258411340000030.00.00.00.000149816598116530520.00012204193321456230.000226916702688957230.02.74966160074e-050.00.00.03.954175786354331e-054.294809015856728e-050.00.05.37684548478e-050.00.00.02.955940232446252e-059.592871797096783e-053.581724424796286e-050.00.00.00.00.00.00.00.00.000118403885857999990.00.000132936543199570720.000112993140972067160.000109281973402226350.00.00.03.56478957015e-050.00.05.436462442995684e-055.257906267465607e-050.00.00.00.00.00.00.00.00.00.00.01.44403912741e-059.22100005526e-054.332117382222432e-050.00.00.000189756637546507114.1063220998003205e-054.5810143113381955e-056.05587982764e-050.0004146343770086.365975308148458e-055.410939145141245e-056.390725029638332e-050.00037485725884234240.000428202845275686660.00044084302690522420.000184014500049000010.00.000169677830343006780.000242880110030907430.00013948555977309190.00.00.04.18503932295e-050.00.000125551179688483470.00.00.00.00.00.00071452694439999990.001228289350580.00090969257958016950.00055985942558930010.00067402882803126730.00139083110421695340.00106191383921057630.00123212310831201070.000472637145059000060.00059540804417999990.000313322054927347170.00049046358945181830.00061412579079791750.000446890754230096750.00097302427924892170.00036630909906047170.000472637145059000060.00059540804417999990.000313322054927347170.00049046358945181830.00061412579079791750.000446890754230096750.00097302427924892170.00036630909906047170.1580540636020.095702459450.156602624217358740.157998054525150760.159561512064211260.099549039824136430.093278376775408930.09427996175052140.1580540636020.095702459450.156602624217358740.157998054525150760.159561512064211260.099549039824136430.093278376775408930.09427996175052140.1580540636020.095702459450.156602624217358740.157998054525150760.159561512064211260.099549039824136430.093278376775408930.09427996175052147.83760965419e-050.0001598696945296.729293998191669e-055.7197520492063784e-050.000110637829151633590.00017955686573271530.000227528963991417247.252325386188014e-057.83760965419e-050.0001598696945296.729293998191669e-055.7197520492063784e-050.000110637829151633590.00017955686573271530.000227528963991417247.252325386188014e-050.00.00.00.00.00.00.00.00.00.00.00.00.00.00.00.07.83760965419e-050.0001598696945296.729293998191669e-055.7197520492063784e-050.000110637829151633590.00017955686573271530.000227528963991417247.252325386188014e-057.83760965419e-050.0001598696945296.729293998191669e-055.7197520492063784e-050.000110637829151633590.00017955686573271530.000227528963991417247.252325386188014e-057.30627553572e-050.00083644401306130027.187028660612834e-058.116509348902961e-056.615288597642188e-050.00077427161102244110.00072012513197872210.00101493529618491867.30627553572e-050.00083644401306130027.187028660612834e-058.116509348902961e-056.615288597642188e-050.00077427161102244110.00072012513197872210.00101493529618491867.30627553572e-050.00083644401306130027.187028660612834e-058.116509348902961e-056.615288597642188e-050.00077427161102244110.00072012513197872210.00101493529618491861.34798834844e-050.00.04.043965045316088e-050.00.00.00.00.00.000280779776840.00.00.00.000227551625441145450.00024715410374269850.00036763360133758112.20509619921e-050.000138643521786000020.00.06.615288597642188e-050.000193479724305572886.615741160789406e-050.000156293429445656123.75319098807e-050.00039594918709300017.187028660612834e-054.0725443035868734e-050.00.000322938160400394350.000373901135476463650.00049100826540168130.02.10715273423e-050.00.00.03.030210087532841e-053.291248115166588e-050.03.10642094075e-050.000182779718217999999.319262822237948e-050.00.00.000207220552377675439.002864260043312e-050.000251089959676440663.10642094075e-050.000182779718217999999.319262822237948e-050.00.00.000207220552377675439.002864260043312e-050.000251089959676440663.10642094075e-050.000182779718217999999.319262822237948e-050.00.00.000207220552377675439.002864260043312e-050.000251089959676440663.10642094075e-050.000182779718217999999.319262822237948e-050.00.00.000207220552377675439.002864260043312e-050.000251089959676440660.000333126533231670.000344128867755400060.000220204542755807960.00034103947537049350.00043813558156911890.000290188038020911870.000133664974473092040.00060853359077346450.000333126533231670.000344128867755400060.000220204542755807960.00034103947537049350.00043813558156911890.000290188038020911870.000133664974473092040.00060853359077346450.000333126533231670.000344128867755400060.000220204542755807960.00034103947537049350.00043813558156911890.000290188038020911870.000133664974473092040.00060853359077346450.00.00.00.00.00.00.00.00.00.00.00.00.00.00.00.00.00.00.00.00.00.00.00.04.2263661089699994e-060.00.01.2679098326898625e-050.00.00.00.04.2263661089699994e-060.00.01.2679098326898625e-050.00.00.00.00.00.00.00.00.00.00.00.00.00.00.00.00.00.00.00.05.44931460007e-050.00.00.000163479438002139720.00.00.00.05.44931460007e-050.00.00.000163479438002139720.00.00.00.00.01.74593018556e-050.00.00.00.00.05.2377905566913425e-050.01.74593018556e-050.00.00.00.00.05.2377905566913425e-050.00.00.00.00.00.00.00.03.79141540375e-054.4554991491e-050.00.00.000113742462112521290.00.000133664974473092040.03.79141540375e-054.4554991491e-050.00.00.000113742462112521290.00.000133664974473092040.01.12433892258e-050.00013277808330783.3730167677503024e-050.00.00.000124332331426605240.00.00027400191849691580.00.00.00.00.00.00.00.01.12433892258e-059.13339728323e-053.3730167677503024e-050.00.00.00.00.00027400191849691580.04.14441104755e-050.00.00.00.000124332331426605240.00.00.00.00.00.00.00.00.00.02.52761026917e-050.00.00.07.582830807501421e-050.00.00.00.00.00.00.00.00.00.00.00.00.00.00.00.00.00.00.02.52761026917e-050.00.00.07.582830807501421e-050.00.00.00.000199973375167000020.0001493364911010.000186474375078304930.000164880939041455170.00024856481138158340.000165855706594306660.00.00028215376670963530.000199973375167000020.0001493364911010.000186474375078304930.000164880939041455170.00024856481138158340.000165855706594306660.00.00028215376670963530.0057143032661330.0146978253454006680.005002514272923240.00587846892144539350.00626192660403284640.0151848574275528850.0145436090699705510.0143650095386639220.000241927861664000027.1333163812e-050.000224743548171650.00025470277080996360.000246337266011648270.000133262277882659770.08.073721355341904e-050.000241927861664000027.1333163812e-050.000224743548171650.00025470277080996360.000246337266011648270.000133262277882659770.08.073721355341904e-050.02.22103796471e-050.00.00.06.663113894132988e-050.00.00.02.22103796471e-050.00.00.06.663113894132988e-050.00.00.000241927861664000024.91227841649e-050.000224743548171650.00025470277080996360.000246337266011648276.663113894132988e-050.08.073721355341904e-050.000241927861664000024.91227841649e-050.000224743548171650.00025470277080996360.000246337266011648276.663113894132988e-050.08.073721355341904e-050.01.23167125118e-050.00.00.00.01.7465556664484026e-051.94845808708918e-050.01.23167125118e-050.00.00.00.01.7465556664484026e-051.94845808708918e-050.01.23167125118e-050.00.00.00.01.7465556664484026e-051.94845808708918e-050.01.23167125118e-050.00.00.00.01.7465556664484026e-051.94845808708918e-050.0054723754044690010.014614175469076870.004777770724751590.0056237661506354290.0060155893380211980.0150515951496702250.0145261435133060660.0142647877442396120.0054723754044690010.014614175469076870.004777770724751590.0056237661506354290.0060155893380211980.0150515951496702250.0145261435133060660.0142647877442396120.00494076118751690.014129342888687870.004502125532914970.0048612003114038070.0054589577182318150.0146787470612267730.0139147167062751450.0137945648985476920.002058293175490.0054990924524299990.0018770355196177680.00198241626713022540.00231542773972451570.0057240973594080750.0055373919470968250.0052357880507799820.0007154610749590.001693454637710.00076439172437180260.00075374330189280080.000628248198611050.00157372943881938520.00168254776308081250.00182408671121707630.000177100166420999970.00063260386880699994.4024439631026676e-050.00025958519803568140.000227690861596887220.00062158041408382150.0005200757168582830.00075615547547843390.05.52233748855e-050.00.00.03.890398757542165e-050.000126766137080932460.01.83816592969e-059.68693666537e-060.05.5144977890673926e-050.02.9060809996098097e-050.00.00.001971525111350.0062392816181899990.00181667384929437270.00181031056645442530.00228759091829936230.006691375051343970.0060479351421582930.0059785346610721990.000225100510047999950.00.00.00039939159729730680.00027590993284720510.00.00.00.000225100510047999950.00.00.00039939159729730680.00027590993284720510.00.00.05.53251050015e-050.00.000165975315004509030.00.00.00.00.00.00.00.00.00.00.00.00.00.00.00.00.00.00.00.00.00.00.00.00.00.00.00.00.05.53251050015e-050.00.000165975315004509030.00.00.00.00.00.0001672180031390.0003270969161940.000109669876832111360.00023511534883264010.000156868783752987780.00037284808844345210.000300591321703485150.00030785133843448880.0001672180031390.0003270969161940.000109669876832111360.00023511534883264010.000156868783752987780.00037284808844345210.000300591321703485150.00030785133843448888.39705987636e-050.0001577356641950.00.000128058893101676120.000123852903189189840.00.00031083548532743650.000162371507257431648.39705987636e-050.0001577356641950.00.000128058893101676120.000123852903189189840.00.00031083548532743650.000162371507257431642.07663069895e-050.00.06.229892096838298e-050.00.00.00.02.07663069895e-050.00.06.229892096838298e-050.00.00.00.02.07663069895e-050.00.06.229892096838298e-050.00.00.00.02.07663069895e-050.00.06.229892096838298e-050.00.00.00.02.07663069895e-050.00.06.229892096838298e-050.00.00.00.00.00.00.00.00.00.00.00.00.00.00.00.00.00.00.00.00.00.00.00.00.00.00.00.00.00602021497396460.00366452519562140.005026661584576370.0063928068751637550.0066411764621418840.0041185302797928020.0034538108679762720.00342123443909652170.00602021497396460.00366452519562140.005026661584576370.0063928068751637550.0066411764621418840.0041185302797928020.0034538108679762720.00342123443909652170.00602021497396460.00366452519562140.005026661584576370.0063928068751637550.0066411764621418840.0041185302797928020.0034538108679762720.00342123443909652170.00602021497396460.00366452519562140.005026661584576370.0063928068751637550.0066411764621418840.0041185302797928020.0034538108679762720.00342123443909652172.5979273074000003e-050.04.27745344049092e-050.03.516328481712015e-050.00.00.00.00284728272430.00152346955676999980.00215156364378526470.0029175730839759820.00347271144512831770.0017386396201757590.00165523592974743850.00117653312039132840.00.00.00.00.00.00.00.02.91556101659e-058.97625891644e-052.4834299279040687e-054.221721750604707e-052.041531371250382e-056.626503378231159e-059.596459705760456e-050.000107058136653251640.0002831600748220.00.000124304497939502940.00040511054968054580.000320065176847426860.00.00.03.14880262345e-057.1071761714e-050.00.09.446407870361935e-050.000102205391087972120.00011100989405392390.04.79395584566e-050.03.669696185752697e-054.67874169654297e-056.033429654683132e-050.00.00.01.20635944665e-050.00.00.03.619078339943859e-050.00.00.00.000988032437040.0001715128380330.00100499524705720290.00100193082919210520.00095717123486970780.000201990358254811730.00014773497178604910.000164813184058295250.0004427878468610.00067770204116599990.00046974807106038040.000458961431585956560.00039965403793602730.00071680154038742250.00049327941374451240.00082302516936549737.43732963873e-050.05.136184623619778e-058.731288166023372e-058.444516126535672e-050.00.00.00.01.42692456409e-050.00.00.00.04.280773692275497e-050.01.54287823014e-050.0001010417817140.04.6286346904220285e-050.00.000145304049980490470.000157821295161000260.00.00066986005107500010.0007819191215840.00069033642161878310.0006986669111867260.00062057682041890430.00077243792166896730.00066223939000502160.00091108005307704470.000286857629872999960.00.00027938464571138130.000312981368190277840.00026820687571645650.00.00.00.00.00.00.00.00.00.00.03.84870895829e-053.04551417888e-050.08.731288166023372e-052.814838708845224e-059.136542536652052e-050.00.00.00.00.00.00.00.00.00.00.000148419836539000020.00.000150661415626180160.000170745190802234830.000123852903189189840.00.00.04.23135593092e-050.0001672385608780.07.71783507532423e-054.976232717422806e-050.000283520939088547168.771763949796665e-050.00013047710404615040.00.00.00.00.00.00.00.00.00.00.00.00.00.00.00.02.60598409956e-050.00.03.974241510052017e-053.8437107886300294e-050.00.00.01.05257424807e-053.60825571683e-050.00.03.157722744200308e-050.00.00.000108247671504954420.01571909127580.01330339529390.0167311908929715270.0148623813133846370.015563701621163910.0144734552408015220.0125202573005612740.012916473340301540.01571909127580.01330339529390.0167311908929715270.0148623813133846370.015563701621163910.0144734552408015220.0125202573005612740.012916473340301540.01571909127580.01330339529390.0167311908929715270.0148623813133846370.015563701621163910.0144734552408015220.0125202573005612740.012916473340301540.01571909127580.01330339529390.0167311908929715270.0148623813133846370.015563701621163910.0144734552408015220.0125202573005612740.012916473340301540.01571909127580.01330339529390.0167311908929715270.0148623813133846370.015563701621163910.0144734552408015220.0125202573005612740.012916473340301540.00.00.00.00.00.00.00.00.00.00.00.00.00.00.00.00.00.00.00.00.00.00.00.00.00.00.00.00.00.00.00.00.00.00.00.00.00.00.00.00.00.00.00.00.00.00.00.00.008702148814823390.0389411637914555060.0075659351321836750.0094391341211819640.0091013771910866330.039342191563887570.041139058123290910.0363422416873202740.00.0004910227074060.00.00.00.000446675412902989150.000485154351791222950.00054123835752477190.00.0004910227074060.00.00.00.000446675412902989150.000485154351791222950.00054123835752477190.00.0004910227074060.00.00.00.000446675412902989150.000485154351791222950.00054123835752477190.00.0004910227074060.00.00.00.000446675412902989150.000485154351791222950.00054123835752477190.00.0004910227074060.00.00.00.000446675412902989150.000485154351791222950.00054123835752477190.00680985681857908950.0305074861557385020.0060260698566586140.0069181396738245670.007485360925239110.0305980309036580.031877233951346110.0290471936123183480.00680985681857908950.0305074861557385020.0060260698566586140.0069181396738245670.007485360925239110.0305980309036580.031877233951346110.0290471936123183480.00680985681857908950.0304863414140139030.0060260698566586140.0069181396738245670.007485360925239110.0305980309036580.031813799726172440.0290471936123183480.04.04244416409e-050.00.00.00.00.00.000121273324922563030.04.04244416409e-050.00.00.00.00.00.000121273324922563030.0067703641632238890.0300948785509040.0060260698566586140.0068461065464548750.00743891608654316350.0301929662214488470.03152187033181290.0285697990995570645.48823827579e-060.0005159388281080.01.6464714827358357e-050.00.0004444808256554330.00038620606483574720.0007171295938323170.0001069591379040.0002621557112130.000142357243111351339.075039668622716e-058.776977391359909e-050.000158270815595547360.000206286102336425520.000421910215708286850.000232662032633999980.001960362749910.000153042786789923780.000228589847117217430.000316353463994305770.00153950965055538220.00222725330116107550.002114325298020110.002750775491460.009904497205480.00256373210447928030.0028676560251246020.00282093834476595950.009229757605527660.0108356302058865240.0096481038050301485.86187979801e-050.000182303725792999980.00.00013298423514404834.287215879625803e-050.000231927618238090578.767849731166679e-050.000227305061827942960.003615860464970.0172696203304000020.0031669377222780590.0035096613275554210.00417098234507304150.0185890197058767370.017778816160281460.0154410251251382563.94926553552e-050.0003510384214690.07.203312736969282e-054.644483869594619e-050.00040506468220915180.000291929394359536850.000356121187838721563.94926553552e-050.0003510384214690.07.203312736969282e-054.644483869594619e-050.00040506468220915180.000291929394359536850.000356121187838721560.02.11447417246e-050.00.00.00.06.343422517367081e-050.00.02.11447417246e-050.00.00.00.06.343422517367081e-050.00.02.11447417246e-050.00.00.00.06.343422517367081e-050.00.0014631263428680.0072873532359215010.00090176986816712120.00189988416688902980.00158772499354542430.0076993349862796820.0080874193805656170.00607530534094474950.0014631263428680.0072873532359215010.00090176986816712120.00189988416688902980.00158772499354542430.0076993349862796820.0080874193805656170.00607530534094474950.0014631263428680.0072873532359215010.00090176986816712120.00189988416688902980.00158772499354542430.0076993349862796820.0080874193805656170.00607530534094474950.0014440901869770.0067956548103180.00090176986816712120.00187085318356623170.00155964750919523020.0073038942669679830.0075063864172769160.0055766837467348520.00.0001232244585680.00.00.00.00019384562404687130.00017582775165587950.00.00073435235453199990.002461501302670.00051400204275555710.00104936449040153830.000639690530437490.0025598404651073180.00273584229300065760.0020888211499155320.00070973783244500010.004210929049080.00038776782541156410.00082148869316469350.00091995697875774020.0045502081778137940.00459471637262037860.003487862596819320.00.000216725607116899960.00.00.00.000151132973715405020.00033148166724877880.00016756218038681760.00.00.00.00.00.00.00.00.06.18728952822e-050.00.00.04.116121552348365e-054.4707056308406204e-059.97504140148044e-050.06.32006519168e-050.00.00.00.000109971758191921367.963019755843781e-050.00.04.28655887881e-050.00.00.00.06.0784999992403797e-056.78117663720132e-050.04.87864711298e-050.00.00.00.00.000146359413389530950.00.00.00.00.00.00.00.00.01.9036155890999998e-050.000238756125417999980.02.9030983322798107e-052.807748435019417e-050.00024430774559629320.0002495512960399220.00022240933461736271.9036155890999998e-050.000238756125417999980.02.9030983322798107e-052.807748435019417e-050.00024430774559629320.0002495512960399220.00022240933461736270.03.62166930686e-050.00.00.00.00.00.000108650079205716320.03.62166930686e-050.00.00.00.00.00.000108650079205716320.00042916565337630.00065530169238950.00063809540735793950.00062111028046836752.82912723020992e-050.00059815026104689030.00068925043958796130.00067850437653240171.402104669e-051.59357268837e-050.04.2063140069893625e-050.00.04.780718065095993e-050.01.402104669e-051.59357268837e-050.04.2063140069893625e-050.00.04.780718065095993e-050.01.402104669e-051.59357268837e-050.04.2063140069893625e-050.00.04.780718065095993e-050.01.402104669e-051.59357268837e-050.04.2063140069893625e-050.00.04.780718065095993e-050.00.00.00.00.00.00.00.00.00.00.00.00.00.00.00.00.00.000395187376592500050.00063936596550580.00063809540735793950.00051917545011717082.82912723020992e-050.00059815026104689030.00064144325893700130.00067850437653240170.000336534448454300050.00042781937656280.00063809540735793950.0003432166657026542.82912723020992e-050.00059815026104689030.000341462323859985550.00034384554478044353.3441466557300003e-052.12854514778e-050.07.203312736969282e-052.82912723020992e-053.060973641720992e-053.324661801614979e-050.00.00.00.00.00.00.00.00.09.4304241007e-062.12854514778e-050.00.02.82912723020992e-053.060973641720992e-053.324661801614979e-050.02.40110424566e-050.00.07.203312736969282e-050.00.00.00.00.000303092981897000050.0004065339250850.00063809540735793950.00027118353833296120.00.00056754052462968040.00030821570584383580.00034384554478044350.000303092981897000050.0004065339250850.00063809540735793950.00027118353833296120.00.00056754052462968040.00030821570584383580.00034384554478044355.86529281382e-050.000211546588942999980.00.000175958784414516830.00.00.000299980935077015750.00033465883175195835.86529281382e-050.000211546588942999980.00.000175958784414516830.00.00.000299980935077015750.00033465883175195835.86529281382e-050.000211546588942999980.00.000175958784414516830.00.00.000299980935077015750.00033465883175195831.99572300938e-050.00.05.987169028130312e-050.00.00.00.01.99572300938e-050.00.05.987169028130312e-050.00.00.00.01.99572300938e-050.00.05.987169028130312e-050.00.00.00.01.99572300938e-050.00.05.987169028130312e-050.00.00.00.00.00.00.00.00.00.00.00.00.00.00.00.00.00.00.00.00.00.00.00.00.00.00.00.00.00.00.00.00.00.00.00.00.00.00.00.00.00.00.00.00.00.00.00.00.00.00.00.00.00.00.00.00.00.00.00.00.00011810542594580.000109868431382000010.00023922967039484725.8504062838329186e-055.65825446041984e-050.000122438945668839670.000132986472064599177.41798764120246e-050.00011810542594580.000109868431382000010.00023922967039484725.8504062838329186e-055.65825446041984e-050.000122438945668839670.000132986472064599177.41798764120246e-050.00.00.00.00.00.00.00.00.00.00.00.00.00.00.00.00.00.00.00.00.00.00.00.00.00.00.00.00.00.00.00.00.00011810542594580.000109868431382000010.00023922967039484725.8504062838329186e-055.65825446041984e-050.000122438945668839670.000132986472064599177.41798764120246e-050.00011810542594580.000109868431382000010.00023922967039484725.8504062838329186e-055.65825446041984e-050.000122438945668839670.000132986472064599177.41798764120246e-050.00.00.00.00.00.00.00.00.00.00.00.00.00.00.00.00.00011810542594580.000109868431382000010.00023922967039484725.8504062838329186e-055.65825446041984e-050.000122438945668839670.000132986472064599177.41798764120246e-053.38564978935e-050.00.000101569493680570910.00.00.00.00.08.42489280523e-050.000109868431382000010.000137660176714276285.8504062838329186e-055.65825446041984e-050.000122438945668839670.000132986472064599177.41798764120246e-050.00.00.00.00.00.00.00.00.00.00.00.00.00.00.00.00.00.00.00.00.00.00.00.00.00.00.00.00.00.00.00.00.00.00.00.00.00.00.00.00.00.00.00.00.00.00.00.00.0001276422155570.07.776441063996426e-050.000195174134732865430.000109988101299639430.00.00.00.0001276422155570.07.776441063996426e-050.000195174134732865430.000109988101299639430.00.00.00.0001276422155570.07.776441063996426e-050.000195174134732865430.000109988101299639430.00.00.00.0001276422155570.07.776441063996426e-050.000195174134732865430.000109988101299639430.00.00.00.0001276422155570.07.776441063996426e-050.000195174134732865430.000109988101299639430.00.00.00.00.00.00.00.00.00.00.00.0001276422155570.07.776441063996426e-050.000195174134732865430.000109988101299639430.00.00.00.0294488565920.0395225782218000050.0277448616594353420.0296764904255761750.0309252176910335930.0413475349170881750.038812253353989450.03840794639439640.0294488565920.0395225782218000050.0277448616594353420.0296764904255761750.0309252176910335930.0413475349170881750.038812253353989450.03840794639439640.0294488565920.0395225782218000050.0277448616594353420.0296764904255761750.0309252176910335930.0413475349170881750.038812253353989450.03840794639439640.0294488565920.0395225782218000050.0277448616594353420.0296764904255761750.0309252176910335930.0413475349170881750.038812253353989450.03840794639439640.0294488565920.0395225782218000050.0277448616594353420.0296764904255761750.0309252176910335930.0413475349170881750.038812253353989450.03840794639439640.0294488565920.0395225782218000050.0277448616594353420.0296764904255761750.0309252176910335930.0413475349170881750.038812253353989450.03840794639439640.033083848454577290.02423636894376920.049752322734448180.026258038256045240.023241184373303510.023669976424241360.0269100652583642470.02212906514886130.00024295768919840.00356717564045099960.000236083118829576380.000100220003296963910.00039256994546949050.0037961950026966940.0038736384840263630.0030316934346238580.00024295768919840.00356717564045099960.000236083118829576380.000100220003296963910.00039256994546949050.0037961950026966940.0038736384840263630.0030316934346238580.00024295768919840.00356717564045099960.000236083118829576380.000100220003296963910.00039256994546949050.0037961950026966940.0038736384840263630.0030316934346238580.00024295768919840.00356717564045099960.000236083118829576380.000100220003296963910.00039256994546949050.0037961950026966940.0038736384840263630.0030316934346238580.00.0001118748810490.00.00.00.000103078941439151360.000149278262089607078.326743961919572e-050.00.0001118748810490.00.00.00.000103078941439151360.000149278262089607078.326743961919572e-050.00024295768919840.00326868390316299980.000236083118829576380.000100220003296963910.00039256994546949050.0036931160612575420.00345973057550517940.0026532050727184230.00.000104325852432000020.00.00.00.000147076050590008660.000106497296734658725.940420997223109e-057.21759869744e-050.0008031402648910.000118174184861261450.09.835377606200372e-050.00083626372865145220.00090004424383588870.00067311282218448050.0001707817022240.002361217785840.000117908933968314910.000100220003296963910.00029421616940748680.00270977628201608160.0024531890349346320.00192068804056171140.00.000186616856239000020.00.00.00.00.00026462964643157620.000295220922286239360.00.000186616856239000020.00.00.00.00.00026462964643157620.000295220922286239360.02293123367034770.0169729566063231970.0392480920680515350.016826755117118960.0127188538259468370.0162516494745298950.019120385951791660.0155468343928089270.05.33906348399e-050.00.00.06.617413524488729e-055.3906039087913674e-054.009173018702016e-050.05.33906348399e-050.00.00.06.617413524488729e-055.3906039087913674e-054.009173018702016e-050.05.33906348399e-050.00.00.06.617413524488729e-055.3906039087913674e-054.009173018702016e-050.05.33906348399e-050.00.00.06.617413524488729e-055.3906039087913674e-054.009173018702016e-050.05.33906348399e-050.00.00.06.617413524488729e-055.3906039087913674e-054.009173018702016e-052.00881887502e-050.06.0264566250472073e-050.00.00.00.00.02.00881887502e-050.06.0264566250472073e-050.00.00.00.00.02.00881887502e-050.06.0264566250472073e-050.00.00.00.00.02.00881887502e-050.06.0264566250472073e-050.00.00.00.00.02.00881887502e-050.06.0264566250472073e-050.00.00.00.00.05.04697049864e-059.9753814296e-060.000151409114959312550.00.02.9926144288785883e-050.00.05.04697049864e-059.9753814296e-060.000151409114959312550.00.02.9926144288785883e-050.00.05.04697049864e-059.9753814296e-060.000151409114959312550.00.02.9926144288785883e-050.00.05.04697049864e-059.9753814296e-060.000151409114959312550.00.02.9926144288785883e-050.00.05.04697049864e-059.9753814296e-060.000151409114959312550.00.02.9926144288785883e-050.00.00.0227970426575053040.0168391961842130970.0388761171721240.0167961569745194440.0127188538259468370.0160915125428809230.0189969268109425260.0154291491989767641.20529132501e-050.03.615873975028324e-050.00.00.00.00.01.20529132501e-050.03.615873975028324e-050.00.00.00.00.01.20529132501e-050.03.615873975028324e-050.00.00.00.00.01.20529132501e-050.03.615873975028324e-050.00.00.00.00.00.0001535373547532.24414506069e-050.00040832251984602093.5245566908718203e-051.704397750309952e-054.729504371914004e-052.0029308101472507e-050.00.0001535373547532.24414506069e-050.00040832251984602093.5245566908718203e-051.704397750309952e-054.729504371914004e-052.0029308101472507e-050.00.0001535373547532.24414506069e-050.00040832251984602093.5245566908718203e-051.704397750309952e-054.729504371914004e-052.0029308101472507e-050.00.0001535373547532.24414506069e-050.00040832251984602093.5245566908718203e-051.704397750309952e-054.729504371914004e-052.0029308101472507e-050.00.0001117543467560.00.000335263040267049170.00.00.00.00.00.0001117543467560.00.000335263040267049170.00.00.00.00.00.0001117543467560.00.000335263040267049170.00.00.00.00.00.0001117543467560.00.000335263040267049170.00.00.00.00.00.02195567776132980.0163892535893339980.03674934746350050.0165509951582683830.0125666906622959980.0155617679389312460.0186282174017911860.0149777754274406630.00430965633717480.00249518908963660030.0086353699672099380.0029222332281465920.00137136581616654780.00215076167426488540.0029268750389527930.0024079305557037638.14616103613e-051.65707368733e-050.000178775910519534826.560892056442609e-050.00.04.971221061997351e-050.08.14616103613e-051.65707368733e-050.000178775910519534826.560892056442609e-050.00.04.971221061997351e-050.01.67401572918e-050.05.022047187539339e-050.00.00.00.00.01.67401572918e-050.05.022047187539339e-050.00.00.00.00.00.00097893394866710010.0005395308310240.00221980309651186860.00054696340782410820.000170035341666514870.0005314680336574550.00066605936432354330.000421065095091305741.98238704771e-050.05.94716114313869e-050.00.00.00.00.00.000959110078190.0005395308310240.00216033148508048160.00054696340782410820.000170035341666514870.0005314680336574550.00066605936432354330.000421065095091305742.40110424566e-050.00.07.203312736969282e-050.00.00.00.02.40110424566e-050.00.07.203312736969282e-050.00.00.00.00.0002916027399224.86299844693e-050.0008748082197649170.00.00.000145889953407831160.00.00.0002916027399224.86299844693e-050.0008748082197649170.00.00.000145889953407831160.00.00.00272499844869999960.001890457537270.00479271725337650.0022088145214404880.00117346357128246520.00147340368719959920.0022111034640092760.0019868654606124570.00272499844869999960.001890457537270.00479271725337650.0022088145214404880.00117346357128246520.00147340368719959920.0022111034640092760.0019868654606124570.000191908389776000020.00.00051904501516172442.881325094787713e-052.786690321756771e-050.00.00.00.000191908389776000020.00.00051904501516172442.881325094787713e-052.786690321756771e-050.00.00.00.01713177502889440.01361837442783970.0268784445841015670.0134538614284805230.0110630190741766060.0131956114510151590.0152178943073952560.0124416175252571820.0003868013341459.95414845304e-050.00110792237820847922.6678936062849192e-052.580268816441455e-050.000110893956832395340.000120075702068327696.765479469059649e-050.0003868013341459.95414845304e-050.00110792237820847922.6678936062849192e-052.580268816441455e-050.000110893956832395340.000120075702068327696.765479469059649e-050.01306591379890.01096109452750.0197194175101281160.0103107055746573320.0091676183119863520.0107440833718435770.0119040144637259560.01023518574706570.01306591379890.01096109452750.0197194175101281160.0103107055746573320.0091676183119863520.0107440833718435770.0119040144637259560.01023518574706570.00265578072101940.00172475297430730.0045973402268665530.0022711905468955050.00109881138928460490.00134253602713927380.0023925252786691930.00143919761712578050.002454060900880.001641793249210.004169199256084850.00214633919423936040.00104664425230301270.00132115262971306680.0022549427618465290.00134928435608288290.0001667725845567.17061447722e-050.000323299264031527040.00012485135265614495.216713698159221e-052.138339742620693e-050.000103821775847501538.991326104289762e-053.49472355834e-051.12535803251e-050.000104841706750176920.00.00.03.376074097516242e-050.00.00.00.00.00.00.00.00.00.00.00.00.00.00.00.00.00.001023279174830.00083298544150200010.0014537644688984160.00084528637086483720.00077078668474123470.00099809809519991260.00080127886293178050.00069957936637510440.001023279174830.00083298544150200010.0014537644688984160.00084528637086483720.00077078668474123470.00099809809519991260.00080127886293178050.00069957936637510444.0235986369000004e-052.59132888197e-050.000120707959107028590.00.00.07.773986645912772e-050.04.0235986369000004e-052.59132888197e-050.000120707959107028590.00.00.07.773986645912772e-050.04.0235986369000004e-052.59132888197e-050.000120707959107028590.00.00.07.773986645912772e-050.01.77248724266e-050.05.317461727982829e-050.00.00.00.00.01.77248724266e-050.05.317461727982829e-050.00.00.00.00.01.77248724266e-050.05.317461727982829e-050.00.00.00.00.00.0004562855364650.0002497767830380.00106165033580213820.000174900501641267180.000132305771952843760.000215394813651202260.000405708188984011450.000128227346479720280.0004562855364650.0002497767830380.00106165033580213820.000174900501641267180.000132305771952843760.000215394813651202260.000405708188984011450.000128227346479720280.0004562855364650.0002497767830380.00106165033580213820.000174900501641267180.000132305771952843760.000215394813651202260.000405708188984011450.000128227346479720284.90398821206e-050.00.00014711964636191190.00.00.00.00.04.90398821206e-050.00.00014711964636191190.00.00.00.00.04.90398821206e-050.00.00014711964636191190.00.00.00.00.04.90398821206e-050.00.00014711964636191190.00.00.00.00.00.00015943787722774.54869334672e-050.00047831363168305470.00.03.2075096139310394e-052.0277349068673407e-058.41083551937045e-053.4983300842e-052.18400613027e-050.000104949902525976870.00.00.02.0277349068673407e-054.5242834839532036e-053.4983300842e-052.18400613027e-050.000104949902525976870.00.00.02.0277349068673407e-054.5242834839532036e-053.4983300842e-052.18400613027e-050.000104949902525976870.00.00.02.0277349068673407e-054.5242834839532036e-051.84483366073e-050.05.534500982186211e-050.00.00.00.00.01.84483366073e-050.05.534500982186211e-050.00.00.00.00.01.84483366073e-050.05.534500982186211e-050.00.00.00.00.09.39853821487e-050.00.000281956146445970460.00.00.00.00.09.39853821487e-050.00.000281956146445970460.00.00.00.00.09.39853821487e-050.00.000281956146445970460.00.00.00.00.01.20208576297e-052.36468721645e-053.6062572889245254e-050.00.03.2075096139310394e-050.03.8865520354172465e-051.20208576297e-052.36468721645e-053.6062572889245254e-050.00.03.2075096139310394e-050.03.8865520354172465e-051.20208576297e-052.36468721645e-053.6062572889245254e-050.00.03.2075096139310394e-050.03.8865520354172465e-051.48842232781e-050.04.4652669834323417e-050.00.00.00.00.01.48842232781e-050.04.4652669834323417e-050.00.00.00.00.01.48842232781e-050.04.4652669834323417e-050.00.00.00.00.01.48842232781e-050.04.4652669834323417e-050.00.00.00.00.00.000340658298790000040.000382014210805000050.00067693946088085130.000209916249342343470.000135119186147738880.000450374464091226460.000328402751981188570.00036726541634239460.000340658298790000040.000382014210805000050.00067693946088085130.000209916249342343470.000135119186147738880.000450374464091226460.000328402751981188570.00036726541634239460.000340658298790000040.000382014210805000050.00067693946088085130.000209916249342343470.000135119186147738880.000450374464091226460.000328402751981188570.00036726541634239460.000340658298790000040.000382014210805000050.00067693946088085130.000209916249342343470.000135119186147738880.000450374464091226460.000328402751981188570.00036726541634239466.36331191058e-057.03944058406e-050.000160301214717767283.059814259951553e-050.06.40366521152958e-056.955310176121957e-057.759346364514432e-056.36331191058e-057.03944058406e-050.000160301214717767283.059814259951553e-050.06.40366521152958e-056.955310176121957e-057.759346364514432e-056.36331191058e-057.03944058406e-050.000160301214717767283.059814259951553e-050.06.40366521152958e-056.955310176121957e-057.759346364514432e-056.36331191058e-057.03944058406e-050.000160301214717767283.059814259951553e-050.06.40366521152958e-056.955310176121957e-057.759346364514432e-056.36331191058e-057.03944058406e-050.000160301214717767283.059814259951553e-050.06.40366521152958e-056.955310176121957e-057.759346364514432e-050.0099096570950311990.00369623669699500020.0102681475475670540.0093310631356293170.0101297606018871840.0036221319470147770.0039160408225462280.00355053732142851650.0091991934901499980.003260659790340.009400934459563470.0086884207395959910.0095082252712796550.0031118911868909780.00351298951490428870.00315709866922781650.0091991934901499980.003260659790340.009400934459563470.0086884207395959910.0095082252712796550.0031118911868909780.00351298951490428870.00315709866922781650.0091991934901499980.003260659790340.009400934459563470.0086884207395959910.0095082252712796550.0031118911868909780.00351298951490428870.00315709866922781650.0091991934901499980.003260659790340.009400934459563470.0086884207395959910.0095082252712796550.0031118911868909780.00351298951490428870.00315709866922781650.0053972788159399990.00105778768770000020.00524267938828090.0052308306258507360.0057183264336904460.00110185182882752660.00115513936519894520.00091637186907525960.003801914674210.002202872102640.00415825507128257140.0034575901137452550.00378989883758920920.0020100393580634510.00235785014970534350.0022407268001525570.00071046360488119990.0004355769066550.00086721308800358460.0006426423960333250.00062153533060752830.00051024076012379920.000403051307641939130.000393438652200699837.94552391942e-050.08.920741714708035e-057.582434459967665e-057.33339558357045e-050.00.00.07.94552391942e-050.08.920741714708035e-057.582434459967665e-057.33339558357045e-050.00.00.07.94552391942e-050.08.920741714708035e-057.582434459967665e-057.33339558357045e-050.00.00.07.94552391942e-050.08.920741714708035e-057.582434459967665e-057.33339558357045e-050.00.00.00.00.0004355769066550.00.00.00.00051024076012379920.000403051307641939130.000393438652200699830.00.0004355769066550.00.00.00.00051024076012379920.000403051307641939130.000393438652200699830.00.0004355769066550.00.00.00.00051024076012379920.000403051307641939130.000393438652200699830.00.0004355769066550.00.00.00.00051024076012379920.000403051307641939130.000393438652200699830.00063100836568699980.00.00077800567085650430.00056681805143364830.00054820137477182380.00.00.00.00063100836568699980.00.00077800567085650430.00056681805143364830.00054820137477182380.00.00.00.00063100836568699980.00.00077800567085650430.00056681805143364830.00054820137477182380.00.00.00.00063100836568699980.00.00077800567085650430.00056681805143364830.00054820137477182380.00.00.00.002827442578630.005321803553320.0041193454448738440.0021325278214212410.00223045446961087430.0058964500155934970.0049639824590176930.0051049781853392780.002827442578630.005321803553320.0041193454448738440.0021325278214212410.00223045446961087430.0058964500155934970.0049639824590176930.0051049781853392780.002827442578630.005321803553320.0041193454448738440.0021325278214212410.00223045446961087430.0058964500155934970.0049639824590176930.0051049781853392780.002827442578630.005321803553320.0041193454448738440.0021325278214212410.00223045446961087430.0058964500155934970.0049639824590176930.0051049781853392780.002827442578630.005321803553320.0041193454448738440.0021325278214212410.00223045446961087430.0058964500155934970.0049639824590176930.0051049781853392780.002827442578630.005321803553320.0041193454448738440.0021325278214212410.00223045446961087430.0058964500155934970.0049639824590176930.0051049781853392780.001888981818430.00152164917411999980.0030765902155975330.00134391439912644140.0012464408405808140.00130479649462400470.0016610239239076430.00159912710382287310.00093846076020000010.00380015437920.00104275522927631130.00078861342229479950.00098401362903006020.0045916535209694930.00330295853511005040.0035058510815164050.03615991761220.03042568849390.056859006211831020.027263671738224970.0243570748866600360.0306476795295069180.030809619430895850.02981976652123850.03615991761220.03042568849390.056859006211831020.027263671738224970.0243570748866600360.0306476795295069180.030809619430895850.02981976652123850.03615991761220.03042568849390.056859006211831020.027263671738224970.0243570748866600360.0306476795295069180.030809619430895850.02981976652123850.03615991761220.03042568849390.056859006211831020.027263671738224970.0243570748866600360.0306476795295069180.030809619430895850.02981976652123850.03615991761220.03042568849390.056859006211831020.027263671738224970.0243570748866600360.0306476795295069180.030809619430895850.02981976652123850.03615991761220.03042568849390.056859006211831020.027263671738224970.0243570748866600360.0306476795295069180.030809619430895850.02981976652123850.03615991761220.03042568849390.056859006211831020.027263671738224970.0243570748866600360.0306476795295069180.030809619430895850.0298197665212385
