# Supplementary material for: Sample Preservation and Storage Significantly Impact Taxonomic and Functional Profiles in Metaproteomics Studies of the Human Gut Microbiome
Source: Microorganisms. 2019 Sep 19;7(9):367. doi: 10.3390/microorganisms7090367 (PMC6780314; doi:10.3390/microorganisms7090367)
Supplement: Supplementary file 1 [file microorganisms-07-00367-s001.zip › Supplement/Supplementary Figure K2 - Functional Annotation according to Prophane.html]

Javascript must be enabled to view this page.

amountdescaff (mean)arl (mean)aff::aff\_01aff::aff\_02aff::aff\_03arl::arl\_01arl::arl\_02arl::arl\_030.99999999999966060.99999999999960710.99999999999999980.99999999999999220.99999999999999310.99999999999999290.99999999999999190.99999999999999370.100165320426189640.116707290982080740.123231919916899140.09170970328813530.085554338073590950.113438480793439350.116664776930990950.120018615221834570.00229001986450970.00136012225399790.00260044563026633550.00205927072860604160.00221034323466747250.00138028193103920160.00155888175066226130.00114120308029232915.674648339479999e-050.00020088906766050.000119467201818116435.077224836630331e-050.00.000216650948773306130.000211005048780975090.000175011205427171865.674648339479999e-050.00020088906766050.000119467201818116435.077224836630331e-050.00.000216650948773306130.000211005048780975090.000175011205427171863.68352830918e-050.05.9733600909058216e-055.077224836630331e-050.00.00.00.00.01.80429304385e-050.00.00.00.05.412879131554968e-050.00.00.0001828461372220.00.00.00.000216650948773306130.00015687625746542540.000175011205427171861.9911200303e-050.05.9733600909058216e-050.00.00.00.00.00.00.00.00.00.00.00.00.00.00.00.00.00.00.00.00.00.00.00.00.00.00.00.00.00.000400644571768999950.000644317593410.00.00049043831400641910.00071149540129960120.00076980230734344940.00069676422863633090.00046638624425006960.000400644571768999950.000644317593410.00.00049043831400641910.00071149540129960120.00076980230734344940.00069676422863633090.00046638624425006960.000400644571768999950.000644317593410.00.00049043831400641910.00071149540129960120.00076980230734344940.00069676422863633090.00046638624425006960.00130904706888623.38371222851e-050.00132143094227998840.00119195345143948060.0014137568129520650.04.7982298528802273e-055.352906832662581e-050.00130904706888623.38371222851e-050.00132143094227998840.00119195345143948060.0014137568129520650.04.7982298528802273e-055.352906832662581e-050.00.00.00.00.00.00.00.00.03.38371222851e-050.00.00.00.04.7982298528802273e-055.352906832662581e-050.00.00.00.00.00.00.00.08.32704628127e-050.00.000127319506162969160.00.000122491882275022940.00.00.00.00.00.00.00.00.00.00.00.00.00.00.00.00.00.00.00.00.00.00.00.00.00.00.04.85027966165e-050.05.445593335886029e-054.6286346904220285e-054.476610958645416e-050.00.00.00.0001182110302170.00.000148461978901710388.412628013978725e-050.000122044831609785620.00.00.00.00.00.00.00.00.00.00.00.001059062779240.00.00099119352385644850.0010615408243954730.00112445398948080230.00.00.00.00.00.00.00.00.00.00.00.00012669679858074.9436410336300006e-050.000207018701424199278.798067390229224e-058.509102041580632e-050.00.000148309231008930120.00.00012669679858074.9436410336300006e-050.000207018701424199278.798067390229224e-058.509102041580632e-050.00.000148309231008930120.00.00.00.00.00.00.00.00.06.35901870739e-050.00.00010390442456977944.4158239000577975e-054.2707897651444776e-050.00.00.00.00.00.00.00.00.00.00.00.01.62319299856e-050.00.00.00.04.869578995673985e-050.00.00.00.00.00.00.00.00.00.00.00.00.00.00.00.00.00.00.00.00.00.00.00.00.00.00.00.00.00.00.00.00.06.31066115068e-053.32044803507e-050.000103114276854419894.382243490171426e-054.238312276436155e-050.09.961344105219026e-050.00.000396884941879000050.0001859103097840.00095252878474403160.000238126040891546490.09.967137312711329e-050.000216515165262198670.00024154439096146850.000396884941879000050.0001859103097840.00095252878474403160.000238126040891546490.09.967137312711329e-050.000216515165262198670.00024154439096146850.000396884941879000050.0001859103097840.00095252878474403160.000238126040891546490.09.967137312711329e-050.000216515165262198670.00024154439096146850.04.03686067767e-050.00.00.00.00.00.000121105820330128560.04.03686067767e-050.00.00.00.00.00.000121105820330128560.04.03686067767e-050.00.00.00.00.00.000121105820330128560.00.000183176720776400030.00.00.00.000262251915159405150.000203651896174222658.362635099686466e-050.00.000183176720776400030.00.00.00.000262251915159405150.000203651896174222658.362635099686466e-050.00.00.00.00.00.00.00.00.00.00.00.00.00.00.00.00.00.000122421589367000030.00.00.00.000186401373345690.000138870763569298834.1992631187266794e-050.02.63177617773e-050.00.00.00.03.731956552240177e-054.163371980959786e-050.03.44373696321e-050.00.00.07.585054181371515e-052.7461567082522054e-050.00.02.21864229689e-050.00.00.03.19053866359278e-053.465388227080164e-050.00.02.21864229689e-050.00.00.03.19053866359278e-053.465388227080164e-050.00.02.21864229689e-050.00.00.03.19053866359278e-053.465388227080164e-050.00.0061085365638750.0130813527362906980.0059548264877989730.00658675039036666750.0057840328134469420.0124369889367514810.0140569451580323320.012750124114087667.79390473606e-056.84822133704e-050.000105551839778100124.1495230888031874e-058.677007141572522e-059.388086143940424e-055.895214895752934e-055.2613629714379293e-057.79390473606e-056.84822133704e-050.000105551839778100124.1495230888031874e-058.677007141572522e-059.388086143940424e-055.895214895752934e-055.2613629714379293e-057.79390473606e-056.84822133704e-050.000105551839778100124.1495230888031874e-058.677007141572522e-059.388086143940424e-055.895214895752934e-055.2613629714379293e-050.09.42548117396e-050.00.00.00.00.000172682057417214960.000110082377801648560.09.42548117396e-050.00.00.00.00.000172682057417214960.000110082377801648560.09.42548117396e-050.00.00.00.00.000172682057417214960.000110082377801648560.00.00068331392880869990.00.00.00.00069411431069817020.00084814753586523160.00050767993986302230.00.00068331392880869990.00.00.00.00069411431069817020.00084814753586523160.00050767993986302230.00.0006192199127860.00.00.00.00069411431069817020.00084814753586523160.000315397891795011130.06.40940160227e-050.00.00.00.00.00.000192282048068011160.09.69183162522e-050.00.00.06.0200845998572594e-050.000108978098988045080.000121576003770123510.09.69183162522e-050.00.00.06.0200845998572594e-050.000108978098988045080.000121576003770123510.09.69183162522e-050.00.00.06.0200845998572594e-050.000108978098988045080.000121576003770123510.06.29862583094e-050.00.00.00.000126949854193481150.06.200892073480416e-050.06.29862583094e-050.00.00.00.000126949854193481150.06.200892073480416e-050.04.23166180645e-050.00.00.00.000126949854193481150.00.00.02.06696402449e-050.00.00.00.00.06.200892073480416e-050.0053949983606324990.009265527870174920.00544495670391237450.0059987617892983090.0047412765886747270.0084312977386493560.0097244972394425060.0096407886324336950.0053949983606324990.009265527870174920.00544495670391237450.0059987617892983090.0047412765886747270.0084312977386493560.0097244972394425060.0096407886324336950.0004848837432730.001288227873140.000451156436902572470.00051012471153140220.00049337008138550890.00136198426858570240.00173321899364935720.0007694803571952460.0006767389395810.001426674195540.00051557138427209940.00104843108406365430.000466214350407976950.0012363926720737520.00099613441052190260.00204749550402745940.03.58570710936e-050.00.00.06.971234767850121e-053.785886560220526e-050.00.002135219923950.00265281065567000030.00227829457776174880.00219027558018415570.0019370896139040970.00236138770113958340.0031416702536724320.00245537401218555140.000221140393006999980.0003535835106030.000200881887501573620.000256117786203352250.00020642150531531640.000402007871612690350.00038812348143297840.000270619178762385960.00.0001015439340960.00.00.00.00.00030463180228751210.00.001195385992220.001894624765060.0012454677025097560.00135587203787047380.00098481823626798230.00159341301839211780.0016371754125900180.00245328586419864880.01.1939081415999998e-050.00.00.00.00.03.581724424796286e-050.00.00.00.00.00.00.00.00.05.47319687385e-050.00.00.00.00.00.000164195906215380330.000401740525535000050.00059255663786999990.00048863161824707070.00041532613978921990.000301263818568299640.00056658122320446070.0006480463601627170.00056304233024293988.07736325147e-050.000244512472650000040.00.00.00024232089754406710.000131089523351964230.00028476451083397870.000317683383764540140.00.00.00.00.00.00.00.00.08.729650927819999e-060.00.00.00.00.02.618895278345672e-055.19821427088e-050.000259689684655.0127642907047e-058.521479023401738e-052.060399498526264e-050.00024521737085432130.000290644390805211060.00024320729229122330.0001471330678430.0001547140287150.000214825453810506320.00013739965942203378.91740902962167e-050.000191071976625325760.000156162624294210130.000116907485225350780.00.0001853323400050.00.00.00.00027243976513093780.000106066133589983990.000177491121293548720.0001632053561830.00141320276922430.000183236856842651550.000155747302420957440.000150631909284149820.00159642168887974380.0013797635498170480.00126342306897207330.0001632053561830.00141320276922430.000183236856842651550.000155747302420957440.000150631909284149820.00159642168887974380.0013797635498170480.00126342306897207330.05.63405053693e-050.00.00.00.00.00016902151610790990.00.00.000469651153346999940.00.00.00.00045510325088229090.0004943082074853970.000459542001671976260.00.00051019996415300010.00.00.00.00065238994549553220.00037054716992887950.0005076627770331610.0001632053561830.0003770111463550.000183236856842651550.000155747302420957440.000150631909284149820.00048892849250192070.000345886656294861670.000296218290266936140.00016349517272260.000257384686331399967.554054265379507e-050.000128415603110315920.000286529372402283750.000201563835348981180.00025541552225220370.0003151747013928160.00016349517272260.000257384686331399967.554054265379507e-050.000128415603110315920.000286529372402283750.000201563835348981180.00025541552225220370.0003151747013928165.41104916846e-052.36463360084e-050.00.00.000162331475053792540.00.07.093900802509149e-050.000109384681037999980.000233738350322999987.554054265379507e-050.000128415603110315920.00012419789734849120.000201563835348981180.00025541552225220370.00024423569336772450.09.98777320776e-050.00.00.00.000299633196233061050.00.00.09.98777320776e-050.00.00.00.000299633196233061050.00.00.04.99388660388e-050.00.00.00.000149816598116530520.00.00.04.99388660388e-050.00.00.00.000149816598116530520.00.00.0001076981766860.0002430860255720.00.00.00032309453005875610.00034957206227190460.00037968601444530490.00.0001076981766860.0002430860255720.00.00.00032309453005875610.00034957206227190460.00037968601444530490.00.0001076981766860.0002430860255720.00.00.00032309453005875610.00034957206227190460.00037968601444530490.06.46978654283e-057.21478666574e-050.00.000118006488523728837.608710776127704e-058.23224310469673e-050.000134121168925218630.06.46978654283e-057.21478666574e-050.00.000118006488523728837.608710776127704e-058.23224310469673e-050.000134121168925218630.06.46978654283e-057.21478666574e-050.00.000118006488523728837.608710776127704e-058.23224310469673e-050.000134121168925218630.00.00.000219904608499980020.00.00.00.00022056150388996230.000232933338658402130.00020621898295243530.00.000219904608499980020.00.00.00.00022056150388996230.000232933338658402130.00020621898295243530.00.000143329238737000010.00.00.00.00013888947579709070.000150854136257539580.000140244104157090650.06.89284535351e-050.00.00.08.167202809287162e-055.9138453717214524e-056.597487879534467e-050.07.64691622788e-060.00.00.00.02.294074868364802e-050.00.000136502584862000020.00050426564927280.000145540544612052420.000144323976125323680.000119643233850022410.000280470608101876960.00076176848326362370.000470557856452664130.000136502584862000020.00050426564927280.000145540544612052420.000144323976125323680.000119643233850022410.000280470608101876960.00076176848326362370.000470557856452664130.03.52128158558e-050.00.00.00.00.000105638447567443730.00.000136502584862000020.0004690528334170.000145540544612052420.000144323976125323680.000119643233850022410.000280470608101876960.000656130035696180.000470557856452664130.01443359301379360.017844842966461730.0134048625037270970.0149163457202026140.0149795708174391420.0176786451524188760.0164528648788327680.019403018868132979.27129658783e-050.03.459063113866381e-050.000109352116021302380.000134196150474860650.00.00.09.27129658783e-050.03.459063113866381e-050.000109352116021302380.000134196150474860650.00.00.09.27129658783e-050.03.459063113866381e-050.000109352116021302380.000134196150474860650.00.00.09.87600718008e-060.00.02.962802154023355e-050.00.00.00.09.87600718008e-060.00.02.962802154023355e-050.00.00.00.09.87600718008e-060.00.02.962802154023355e-050.00.00.00.00.00.00.00.00.00.00.00.00.000313203321064999960.00.000209683413500353840.000356452589045902650.00037347396064781480.00.00.00.000313203321064999960.00.000209683413500353840.000356452589045902650.00037347396064781480.00.00.00.000313203321064999960.00.000209683413500353840.000356452589045902650.00037347396064781480.00.00.00.00.00.00.00.00.00.00.00.00.00.00.00.00.00.00.00.00.00.00.00.00.00.00.08.13231445988e-050.00023808137338620.00.000243969433796388670.06.437848477783297e-050.00059786052629485415.200510908601013e-058.13231445988e-050.00023808137338620.00.000243969433796388670.06.437848477783297e-050.00059786052629485415.200510908601013e-050.00.00.00.00.00.00.00.07.44872606254e-050.000175978714877000020.00.000223461781876191420.00.00.00052793614463099460.06.8358839734e-066.21026585092e-050.02.0507651920197244e-050.06.437848477783297e-056.992438166385955e-055.200510908601013e-050.0001032503239870.000353818800469100070.00.000196008509849504320.000113742462112521290.000302221389954675570.00031188494043721480.000447350071015372730.0001032503239870.000353818800469100070.00.000196008509849504320.000113742462112521290.000302221389954675570.00031188494043721480.000447350071015372730.0001032503239870.000335120759929000050.00.000196008509849504320.000113742462112521290.000246127268334300160.00031188494043721480.000447350071015372730.01.86980405401e-050.00.00.05.6094121620375394e-050.00.01.36102091417e-050.0001107486765450.00.04.083062742500764e-050.000135508271330120327.359082864248887e-050.000123146929661535231.36102091417e-050.0001107486765450.00.04.083062742500764e-050.000135508271330120327.359082864248887e-050.000123146929661535230.00.0001107486765450.00.00.00.000135508271330120327.359082864248887e-050.000123146929661535231.36102091417e-050.00.00.04.083062742500764e-050.00.00.04.23831227644e-054.58564112106e-050.00.00.000127149368293084650.000137569233631719120.00.04.23831227644e-054.58564112106e-050.00.00.000127149368293084650.000137569233631719120.00.04.23831227644e-054.58564112106e-050.00.00.000127149368293084650.000137569233631719120.00.04.52651319066e-050.000155122259618700020.05.5410097976686795e-058.03852977429838e-050.000115963809119045280.000173760714289065870.000175642255446740964.52651319066e-050.000155122259618700020.05.5410097976686795e-058.03852977429838e-050.000115963809119045280.000173760714289065870.000175642255446740960.00.00.00.00.00.00.00.04.52651319066e-050.0001391865327350.05.5410097976686795e-058.03852977429838e-050.000115963809119045280.000125953533638105940.000175642255446740960.01.59357268837e-050.00.00.00.04.780718065095993e-050.00.00.00.00.00.00.00.00.00.00.00.00.00.00.00.00.00.00.00.00.00.00.00.00.00.00.00.00.00.00.00.00.00.01.8931078741e-050.00.00.02.7224009364290538e-052.9569226858607262e-050.00.01.8931078741e-050.00.00.02.7224009364290538e-052.9569226858607262e-050.00.00.00.00.00.00.00.00.00.00.00.00.00.00.00.00.00.01.8931078741e-050.00.00.02.7224009364290538e-052.9569226858607262e-050.00.00.00.00.00.00.00.00.00.0001332426327890.000109953427520999990.000113945608456774920.000145276895535514920.000140505394374290980.000152019783382950135.5038518900684964e-050.00012280198027872980.0001332426327890.000109953427520999990.000113945608456774920.000145276895535514920.000140505394374290980.000152019783382950135.5038518900684964e-050.00012280198027872980.0001332426327890.000109953427520999990.000113945608456774920.000145276895535514920.000140505394374290980.000152019783382950135.5038518900684964e-050.00012280198027872980.02.26239131399e-050.00.00.02.0580607761741825e-052.2353528154203102e-052.4937603503701103e-050.02.26239131399e-050.00.00.02.0580607761741825e-052.2353528154203102e-052.4937603503701103e-050.02.26239131399e-050.00.00.02.0580607761741825e-052.2353528154203102e-052.4937603503701103e-050.0001579278345320.0003034214929940.000135595274063562150.000115253003791508490.00022293522574054170.00042150825344464920.000196487512475445320.00029226871306337690.0001579278345320.0003034214929940.000135595274063562150.000115253003791508490.00022293522574054170.00042150825344464920.000196487512475445320.00029226871306337690.04.0000783244999996e-050.00.00.00.000120002349735131450.00.00.0001579278345320.0002634207097490.000135595274063562150.000115253003791508490.00022293522574054170.000301505903709517740.000196487512475445320.00029226871306337691.01099126133e-050.000173113972231999980.03.032973783987066e-050.00.000158687317741851430.00020682896050046880.00015382563845440891.01099126133e-050.000173113972231999980.03.032973783987066e-050.00.000158687317741851430.00020682896050046880.00015382563845440890.00.00.00.00.00.00.00.01.01099126133e-050.000173113972231999980.03.032973783987066e-050.00.000158687317741851430.00020682896050046880.00015382563845440890.00.00.00.00.00.00.00.00.00017683561209670.0001928270391280.000179306265606512030.00030273639117466874.846417950881342e-050.000199893416823989640.000217113273453530690.00016147442710683810.00017683561209670.0001928270391280.000179306265606512030.00030273639117466874.846417950881342e-050.000199893416823989640.000217113273453530690.00016147442710683812.47806971343e-050.00.05.0110001648481956e-052.423208975440671e-050.00.00.05.96614424559e-050.02.947723349207873e-050.000125275004121204922.423208975440671e-050.00.00.09.23934725065e-050.0001928270391280.00014982903211443330.000127351385404981770.00.000199893416823989640.000217113273453530690.00016147442710683810.00109469842819750.00081024940434040.00141036852224911240.00091985810211950550.00095386866022349680.00075113370642383090.00081606294731267530.00086355155928372440.00109469842819750.00081024940434040.00141036852224911240.00091985810211950550.00095386866022349680.00075113370642383090.00081606294731267530.00086355155928372442.18420632622e-055.23952552535e-050.03.3310116702748124e-053.221607308389331e-053.4856173839250605e-053.785886560220526e-058.447072631889507e-054.45653002122e-054.89210815497e-055.003515648101924e-054.252878368690351e-054.113196046873463e-054.450271641468896e-054.8336411433073875e-055.392411680136106e-050.05.09815676732e-050.00.00.07.331450546128091e-057.963019755843781e-050.06.78494177388e-050.07.617712026042816e-056.474887853455534e-056.262225442150048e-050.00.00.00.0002953544843030.0004779466268010.00035682966858832140.000235897960976771790.000293335823342818060.00042316618064493720.00049792157157520230.0005127521281813630.00.00.00.00.00.00.00.00.00.00.00.00.00.00.00.03.70792791664e-050.000180004873062999987.88344616648617e-050.03.2403375834381074e-050.00017529413006367310.000152315901143756060.000212404587982105330.00.00.00.00.00.00.00.00.00.00.00.00.00.00.00.00.00.00.00.00.00.00.00.04.93959488878e-050.08.071147265688223e-053.4301489223663244e-053.3174884782818705e-050.00.00.02.47384889403e-050.04.0116944989219566e-053.409852183180725e-050.00.00.00.00.000448110383768999970.00.00047857155551845460.000406775307499441770.000458984288289350570.00.00.00.00.00.00.00.00.00.00.02.95414540443e-050.08.862436213304715e-050.00.00.00.00.07.62216078735e-050.00.000160467779956878266.81970436636145e-050.00.00.00.00.00.00.00.00.00.00.00.00.00.000105587630623999990.00.00.00.000205280615291586520.000111482276581812940.00.00.000105587630623999990.00.00.00.000205280615291586520.000111482276581812940.00.00.000105587630623999990.00.00.00.000205280615291586520.000111482276581812940.00.00020023576217040.00059773663364089990.00050292867872969170.09.777860778093934e-050.00059361053200576770.00071466451497026740.000484934853947994470.00020023576217040.00059773663364089990.00050292867872969170.09.777860778093934e-050.00059361053200576770.00071466451497026740.000484934853947994477.06225385748e-050.000203825726164999970.000211867615724315850.00.00.000235551487273060680.000204674492161922180.000171251199060572420.0001118883511690.000270891362860.00023788644572554760.09.777860778093934e-050.000211583090322468550.000344714934167447860.00025637606409068150.05.54472045581e-050.00.00.05.243580934078569e-050.000113905804333591490.01.77248724266e-056.75723400578e-055.317461727982829e-050.00.09.404014506945286e-055.136928430730596e-055.7307590796740575e-050.01.87784888102e-050.00.00.01.7082487462295623e-051.855406161241221e-052.069891735576324e-050.01.87784888102e-050.00.00.01.7082487462295623e-051.855406161241221e-052.069891735576324e-050.01.87784888102e-050.00.00.01.7082487462295623e-051.855406161241221e-052.069891735576324e-057.60807438462e-058.28600818748e-050.00.000116026513884068970.000112215717654635087.537647592737943e-058.186979686476888e-059.13339728323053e-057.60807438462e-058.28600818748e-050.00.000116026513884068970.000112215717654635087.537647592737943e-058.186979686476888e-059.13339728323053e-057.60807438462e-050.00.00.000116026513884068970.000112215717654635080.00.00.00.08.28600818748e-050.00.00.07.537647592737943e-058.186979686476888e-059.13339728323053e-050.05.19285936981e-050.00.00.07.46763848196948e-058.110939627469363e-050.00.05.19285936981e-050.00.00.07.46763848196948e-058.110939627469363e-050.00.05.19285936981e-050.00.00.07.46763848196948e-058.110939627469363e-050.00.0001642903019290.0002104976232070.00.000250550008242409840.00024232089754406710.00017478603113595230.00.00045670683848459040.0001642903019290.0002104976232070.00.000250550008242409840.00024232089754406710.00017478603113595230.00.00045670683848459040.08.1639305325e-050.00.00.00.00.00.000244917915974896940.0001642903019290.0001288583178820.00.000250550008242409840.00024232089754406710.00017478603113595230.00.000211788922509693440.0001121721023920.000162618254947999970.000125939883031172260.000107046133552484680.000103530290591582840.00014935276963938960.00020277349068673410.00013572850451859610.0001121721023920.000162618254947999970.000125939883031172260.000107046133552484680.000103530290591582840.00014935276963938960.00020277349068673410.00013572850451859610.0001121721023920.000162618254947999970.000125939883031172260.000107046133552484680.000103530290591582840.00014935276963938960.00020277349068673410.00013572850451859610.02.11447417246e-050.00.00.00.06.343422517367081e-050.00.02.11447417246e-050.00.00.00.06.343422517367081e-050.00.02.11447417246e-050.00.00.00.06.343422517367081e-050.00.00045309640600870.00166977397398430.00049862164475835340.00022442486501811180.00063624270824987320.0022243665895125050.00103276216636070.00175219316607812350.00045309640600870.00166977397398430.00049862164475835340.00022442486501811180.00063624270824987320.0022243665895125050.00103276216636070.00175219316607812350.00.00.00.00.00.00.00.00.00.00.00.00.00.00.00.00.00.00.00.00.00.00.00.00.01.43406837643e-050.00.00.01.9451993787710826e-050.02.3570057505111045e-050.00.00.00.00.00.00.00.00.0002784802734930.0006389873042610.000275366153324796470.00022442486501811180.00033564980213640210.00079502991430776370.0003657896799855010.00075614231848830779.84147868339e-050.0003487319870660.000162038663413890.00.000133205697087708040.00034259500478230220.000318422804305423270.00038517815210956021.71225211782e-050.00050516689319800010.00.05.136756353468703e-050.00079430711000916040.000318980455211168360.000402213114372932145.90788245036e-050.000162547105694999986.121682801966687e-050.00.000116019645491076080.000272982566625567932.9569226858607262e-050.00018508952360221240.00.00.00.00.00.00.00.00.00.00.00.00.00.00.00.00.00.00.00.00.00.00.00.00.03.37172908581e-050.00.00.00.00.000101151872574231820.00.03.37172908581e-050.00.00.00.00.000101151872574231820.00.03.37172908581e-050.00.00.00.00.000101151872574231820.00.0001162266112080.000108341698738999980.000123268430966874680.000104775457992280440.000120635944664795327.30923402932164e-050.000119083340894209250.000132849415028807720.0001162266112080.000108341698738999980.000123268430966874680.000104775457992280440.000120635944664795327.30923402932164e-050.000119083340894209250.000132849415028807729.85333393238e-050.000108341698738999980.000123268430966874680.000104775457992280446.755612901228538e-057.30923402932164e-050.000119083340894209250.000132849415028807721.76932718842e-050.00.00.05.307981565250994e-050.00.00.00.00.0002299047644190.00.00.00.00026476438281410980.000287572561217915770.00013737734922416660.00.0002299047644190.00.00.00.00026476438281410980.000287572561217915770.00013737734922416660.00.0002299047644190.00.00.00.00026476438281410980.000287572561217915770.00013737734922416660.00.0001318995140420.00.00.00.00.000148013192071898540.000247685350053709260.00.0001318995140420.00.00.00.00.000148013192071898540.000247685350053709260.00.0001318995140420.00.00.00.00.000148013192071898540.000247685350053709260.00.00.00.00.00.00.00.00.00.00.00.00.00.00.00.00.00.00.00.00.00.00.00.00.00.00.00.00.00.00.00.07.67939184987e-050.05.286365460567725e-050.00011233236236989136.518573852062624e-050.00.00.07.67939184987e-050.05.286365460567725e-050.00011233236236989136.518573852062624e-050.00.00.07.67939184987e-050.05.286365460567725e-050.00011233236236989136.518573852062624e-050.00.00.00.00025759087765570.000218668181118799960.000217532525235661210.00031680671121769160.000238433396513948380.000340555774895581557.004902405541721e-050.00024539974440400270.00025759087765570.000218668181118799960.000217532525235661210.00031680671121769160.000238433396513948380.000340555774895581557.004902405541721e-050.00024539974440400270.00.00.00.00.00.00.00.02.34254072747e-057.87274551828e-050.07.027622182409055e-050.00.000147076050590008660.08.910631495834663e-050.000234165470380999980.000139940725935999970.000217532525235661210.000246530489393601070.000238433396513948380.000193479724305572887.004902405541721e-050.000156293429445656120.00.00.00.00.00.00.00.07.39749915322e-059.52844328033e-059.341494829862483e-058.004584678913352e-054.846417950881342e-050.000104196479505509765.621960299726617e-050.000125437215907028727.39749915322e-059.52844328033e-059.341494829862483e-058.004584678913352e-054.846417950881342e-050.000104196479505509765.621960299726617e-050.000125437215907028722.17184656152e-050.03.5219551704821337e-052.993584514065156e-050.00.00.00.05.2256525917e-059.52844328033e-055.8195396593803494e-055.0110001648481956e-054.846417950881342e-050.000104196479505509765.621960299726617e-050.000125437215907028722.09926496909e-050.00012484110147760.03.201472327541903e-053.096322579729746e-050.000113565930268964630.000123349089055799360.00013760828510813222.09926496909e-050.00012484110147760.03.201472327541903e-053.096322579729746e-050.000113565930268964630.000123349089055799360.00013760828510813222.09926496909e-057.36534061109e-050.03.201472327541903e-053.096322579729746e-056.700131193544838e-057.277315276868345e-058.118575362871582e-050.05.11876953667e-050.00.00.04.656461833351625e-055.057593628711591e-055.6422531479416395e-050.05.82123838423e-050.00.00.09.564560745576292e-050.07.899154407118297e-050.05.82123838423e-050.00.00.09.564560745576292e-050.07.899154407118297e-050.01.01517139296e-050.00.00.03.0455141788840172e-050.00.00.04.80606699127e-050.00.00.06.519046566692275e-050.07.899154407118297e-059.56627074522e-050.00024873139310850.00.000145889878217099370.000141098244139583340.00021715445850330120.00030498616532060350.000224053555502791819.56627074522e-050.00024873139310850.00.000145889878217099370.000141098244139583340.00021715445850330120.00030498616532060350.000224053555502791810.05.7871874283999996e-050.00.00.06.449324143519095e-057.004902405541721e-053.907335736141403e-059.56627074522e-050.000167817887340999980.00.000145889878217099370.000141098244139583340.000152661217068110240.000165812246814721780.00018498019814137780.00.00.00.00.00.00.00.00.02.30416314835e-050.00.00.00.06.912489445046447e-050.01.66245507636e-056.12221313234e-050.00.04.987365229094893e-052.698039406796579e-055.8609250551959816e-059.807674935012648e-051.66245507636e-056.12221313234e-050.00.04.987365229094893e-052.698039406796579e-055.8609250551959816e-059.807674935012648e-051.66245507636e-056.12221313234e-050.00.04.987365229094893e-052.698039406796579e-055.8609250551959816e-059.807674935012648e-050.000272134622646100040.00.000292954508296910260.000190500832713237170.0003329485269271840.00.00.00.000272134622646100040.00.000292954508296910260.000190500832713237170.0003329485269271840.00.00.00.00.00.00.00.00.00.00.00.000230330411659000020.00.00022412441993977210.000190500832713237170.000276365982322985640.00.00.04.18042109871e-050.06.883008835713814e-050.05.65825446041984e-050.00.00.03.76596182917e-050.000156194447869310.02.8958041153645354e-058.402081372130969e-050.000253866316156895533.291248115166588e-050.000181804546299479273.76596182917e-050.000156194447869310.02.8958041153645354e-058.402081372130969e-050.000253866316156895533.291248115166588e-050.000181804546299479270.05.91188046489e-050.00.00.00.000177356413946775120.00.03.76596182917e-055.77887023503e-050.02.8958041153645354e-058.402081372130969e-053.030210087532841e-053.291248115166588e-050.000110151525023885770.03.40659920837e-050.00.00.04.620780133479199e-050.05.599017491635572e-050.00.00.00.00.00.00.00.00.05.2209487864099995e-060.00.00.00.00.01.5662846359237773e-050.0008361688526810.0009708422671840.00097172448970267960.00073796507429019350.00079881699404925610.00080634376985751510.00098873026590039390.00111745276579245860.0008361688526810.0009708422671840.00097172448970267960.00073796507429019350.00079881699404925610.00080634376985751510.00098873026590039390.00111745276579245860.0005238275675730.00062063618268800010.00062104705677967410.00043989696103629210.0005105386849020040.00059840866385095120.00064995869266686740.0006135411915452570.0003123412851080.0003502060844960.00035067743292300550.000298068113253901340.000288278309147252170.000207935106006563950.00033877157323352640.00050391157424720170.0002599695113990.0002988111569860.000101442848426605080.000344896769450898260.000333568916320012560.000270678616796574530.00026133002490499790.000364424829256081030.0002599695113990.0002988111569860.000101442848426605080.000344896769450898260.000333568916320012560.000270678616796574530.00026133002490499790.000364424829256081030.0002599695113990.0002988111569860.000101442848426605080.000344896769450898260.000333568916320012560.000270678616796574530.00026133002490499790.000364424829256081030.0001069591379040.0002621557112130.000142357243111351339.075039668622716e-058.776977391359909e-050.000158270815595547360.000206286102336425520.000421910215708286850.0001069591379040.0002621557112130.000142357243111351339.075039668622716e-058.776977391359909e-050.000158270815595547360.000206286102336425520.000421910215708286850.0001069591379040.0002621557112130.000142357243111351339.075039668622716e-058.776977391359909e-050.000158270815595547360.000206286102336425520.000421910215708286851.35605368455e-054.73500885624e-050.00.04.0681610536595204e-055.868728052740005e-054.780718065095993e-053.5555804508926635e-051.35605368455e-054.73500885624e-050.00.04.0681610536595204e-055.868728052740005e-054.780718065095993e-053.5555804508926635e-051.35605368455e-054.73500885624e-050.00.04.0681610536595204e-055.868728052740005e-054.780718065095993e-053.5555804508926635e-058.03579094447e-055.72683071273e-050.09.50234378334726e-050.00014605029050060335.209605247680652e-055.658387688277762e-056.312499202232762e-058.03579094447e-055.72683071273e-050.09.50234378334726e-050.00014605029050060335.209605247680652e-055.658387688277762e-056.312499202232762e-055.30430841497e-050.00.06.493126974169493e-059.419798270727114e-050.00.00.02.7314825295e-050.00.03.009216809177768e-055.1852307793332174e-050.00.00.00.05.72683071273e-050.00.00.05.209605247680652e-055.658387688277762e-056.312499202232762e-050.00066282822127865.98701230191e-050.00051989505569476370.00081874979355831220.00064983981458350730.00.000131697465276445834.791290378088146e-050.00066282822127865.98701230191e-050.00051989505569476370.00081874979355831220.00064983981458350730.00.000131697465276445834.791290378088146e-059.68725242494e-050.09.383755990557934e-050.000119639796323365217.71402165192186e-050.00.00.00.01.52670949864e-050.00.00.00.04.580128495931126e-050.06.93800362058e-050.04.791352440408557e-058.145088607173745e-057.877569814153418e-050.00.00.02.72465730004e-050.00.08.173971900106986e-050.00.00.00.00.0004693290878230.00.00037814397138509890.00053591939216213960.00049392389992275450.00.00.00.04.46030280327e-050.00.00.00.08.589618031713456e-054.791290378088146e-050.03.78042352045e-050.00.00.00.00.000113412705613532640.00.03.78042352045e-050.00.00.00.00.000113412705613532640.00.03.78042352045e-050.00.00.00.00.000113412705613532640.01.12661782787e-051.2767219784000002e-050.03.3798534836219506e-050.00.03.8301659351938646e-050.01.12661782787e-051.2767219784000002e-050.03.3798534836219506e-050.00.03.8301659351938646e-050.01.12661782787e-051.2767219784000002e-050.03.3798534836219506e-050.00.03.8301659351938646e-050.00.000143167040990.000218457407878200020.000178906421393636130.00.00025059470157649138.950082484883643e-050.00012737456081900020.000438496837966800740.000143167040990.000218457407878200020.000178906421393636130.00.00025059470157649138.950082484883643e-050.00012737456081900020.000438496837966800740.07.16344884959e-050.00.00.00.00.00.00021490346548777729.67995870022e-052.93442482995e-050.000122525850057435670.00.00016787291094920310.00.08.803274489860752e-051.87935237787e-051.81554643082e-055.6380571336200476e-050.00.00.05.446639292458637e-050.02.75739302091e-059.93232067746e-050.00.08.272179062728822e-058.950082484883643e-057.290816789441384e-050.000135560627580416030.00022215623594210.00.000237753187548344150.000226828965613180660.000201886554665137360.00.00.00.00022215623594210.00.000237753187548344150.000226828965613180660.000201886554665137360.00.00.03.7777199836999997e-050.06.219966700163401e-050.05.113193250929856e-050.00.00.01.2891834875999997e-050.00.03.8675504628022994e-050.00.00.00.00.00.00.00.00.00.00.00.02.55316009754e-050.00.03.893682560523936e-053.7657977321037455e-050.00.00.07.36414412043e-050.08.268004516070863e-057.027622182409055e-056.796805662821394e-050.00.00.00.00.00.00.00.00.00.00.01.50428627355e-050.00.00.04.512858820658739e-050.00.00.05.72712963139e-050.09.287347538600148e-057.894041355582774e-050.00.00.00.00.00.00.00.00.00.00.00.04.24153778045e-060.00.00.01.2724613341355119e-050.00.00.04.24153778045e-060.00.00.01.2724613341355119e-050.00.00.04.24153778045e-060.00.00.01.2724613341355119e-050.00.00.00.00.00.00.00.00.00.00.00.00.00.00.00.00.00.00.00.00.00.00.00.00.00.00.00.000333997628684700050.0002302561806620.000146589485474121250.000186561822469835040.00066884157811070730.000256600769114483150.00027870569145453230.000155462081416689860.000333997628684700050.0002302561806620.000146589485474121250.000186561822469835040.00066884157811070730.000256600769114483150.00027870569145453230.000155462081416689860.00.00.00.00.00.00.00.06.40618464772e-050.00.00.00.00019218553943150150.00.00.00.000150648539517000030.00.000146589485474121250.000124597841936765960.000180758291140979750.00.00.06.06072095936e-050.00.06.196398053306909e-050.000119857648247603070.00.00.01.91525108025e-050.00.00.05.745753240735611e-050.00.00.03.95275222944e-050.0002302561806620.00.00.000118582566883266870.000256600769114483150.00027870569145453230.000155462081416689860.000294206434419000050.000235215530155999980.00035682966858832140.00037912172299838320.000146667911671409030.000158687317741851430.000258536200625585930.00028842307210201670.000294206434419000050.000235215530155999980.00035682966858832140.00037912172299838320.000146667911671409030.000158687317741851430.000258536200625585930.00028842307210201670.000294206434419000050.000235215530155999980.00035682966858832140.00037912172299838320.000146667911671409030.000158687317741851430.000258536200625585930.00028842307210201670.000164415341520.000343933729498000030.000206490265071414430.000117008125676658350.000169747633812595220.00055097525550977860.0003324661801614980.00014835975282404920.000164415341520.000343933729498000030.000206490265071414430.000117008125676658350.000169747633812595220.00055097525550977860.0003324661801614980.00014835975282404920.000164415341520.000343933729498000030.000206490265071414430.000117008125676658350.000169747633812595220.00055097525550977860.0003324661801614980.00014835975282404921.79509593556e-059.96017563212e-060.02.7376010401783496e-052.647686766514748e-051.4323320841307254e-051.555720605506297e-050.01.79509593556e-059.96017563212e-060.02.7376010401783496e-052.647686766514748e-051.4323320841307254e-051.555720605506297e-050.00.00.00.00.00.00.00.00.00.00.00.00.00.00.00.00.00.00.00.00.00.00.00.00.00.00.00.00.00.00.00.00.01.79509593556e-059.96017563212e-060.02.7376010401783496e-052.647686766514748e-051.4323320841307254e-051.555720605506297e-050.00.00.00.00.00.00.00.00.00.00.00.00.00.00.00.00.00.00.00.00.00.00.00.00.00.00.0001924263594230.00.00.00.000196634285027946370.000142382255416989350.000238262537823405080.00.0001924263594230.00.00.00.000196634285027946370.000142382255416989350.000238262537823405080.00.0001924263594230.00.00.00.000196634285027946370.000142382255416989350.000238262537823405080.0020208067600060.00271892530596510.0019127288701909210.00210514671342464530.002044544696401660.00265903054694304350.00240000124965541450.0030977441212959130.0020208067600060.00271892530596510.0019127288701909210.00210514671342464530.002044544696401660.00265903054694304350.00240000124965541450.0030977441212959130.02.35092322581e-050.00.00.07.052769677415619e-050.00.00.0002699133524880.0003254007242040.000274855285263977270.00023362095363143620.000301263818568299640.00032595232833461380.00035403155400981140.000296218290266936140.0004237612757020.000389674713156999940.00047577289145109520.00040439650453160880.00039111443112375740.00028211078709662480.0004596199122232640.000427293440151135840.000116816590112999980.0003497386447268.119477488836057e-056.90137747254542e-050.000200241220725037460.00028886793169774150.00023531438619813810.00052503361628151560.0002994697609950.0004287081964390.00041340022580354310.00028110488729636220.00020390416988464180.000441228151770025850.00039936486275497010.000445531574791733130.0001127562567254.8144655283e-050.00.00013802754945090840.000200241220725037460.000144433965848870760.00.00.0001632053561830.0002358222573120.000183236856842651550.000155747302420957440.000150631909284149820.000244464246250960330.00026552366550735850.00019747886017795740.00063488416780.00079102989406500020.00048426883594129330.00082323574136791790.00059714792609073670.00086144543917005040.000467827410655822150.00104381683236920320.00.000126896988521000010.00.00.00.00.000218319458306050360.000162371507257431640.00116591174667559980.00232810404309330.00068658184171025740.00167621867059352340.00113493472772495150.00204384842503322740.0019877412089527150.00295272249529315770.00116591174667559980.00232810404309330.00068658184171025740.00167621867059352340.00113493472772495150.00204384842503322740.0019877412089527150.00295272249529315770.00.00.00.00.00.00.00.08.84205038771e-055.50739974102e-053.4502614265537445e-050.000117305856276344550.000113453041089334236.137524757445654e-056.666243001711463e-053.7184314639106485e-050.0002800180074850.00057531137403499990.000215230593751685960.000447890709863005070.000176932718841699770.000425771883727726170.00046245008409655980.00083771215428173294.92939656365e-050.0002200798779260.06.0341886801836917e-058.754001010754256e-050.00022099909172425380.000171455071968102370.00026778547008424597.15576424604e-050.0001722026289433.568296685883214e-059.098921351961198e-058.800074700284543e-050.00019042478129022170.000172357467083723930.00015382563845440893.05751806519e-051.01774144712e-053.4327917484446115e-052.917797564341988e-052.8219648827916675e-053.0532243413622055e-050.00.00.000162161013480.04.9487326300570116e-050.000222145958494125680.000214849755646393410.00.00.00.0002363871426880.00048939015722099990.000142357243111351330.00033275145451616630.000234052730436264260.000411504120548423170.000481334238784992850.00057533211232948220.00.00.00.00.00.00.00.05.1669869068999995e-055.69581078479e-053.568296685883214e-056.065947567974132e-055.866716466856361e-056.347492709674056e-056.894298683348958e-053.8456409613602225e-055.81587789887e-050.00.00.000117605105909702575.687123105626064e-050.00.00.00.000137669642338999980.00068467514867199990.00013931021307900220.000197351033889569367.634768004813073e-050.0005197637799226520.00056453893016873170.00096972273592456490.06.4235336567e-050.00.00.00.000120002349735131450.07.270365996601417e-050.001265123172750.001587077542650.00146653599473840680.00127693313781217340.00105190038569333680.00160477392510568250.00144370310182043550.00171275560102689150.001265123172750.001587077542650.00146653599473840680.00127693313781217340.00105190038569333680.00160477392510568250.00144370310182043550.00171275560102689150.001265123172750.001587077542650.00146653599473840680.00127693313781217340.00105190038569333680.00160477392510568250.00144370310182043550.00171275560102689150.00026682229863610.00031964301845860.000223508693511366170.000189977478777211830.000386980723620454670.00041869371663728080.00029935453126727280.000240880807469816170.00026682229863610.00031964301845860.000223508693511366170.000189977478777211830.000386980723620454670.00041869371663728080.00029935453126727280.000240880807469816170.000219489978945999990.000240620428660.000223508693511366170.000189977478777211830.00024498376455004590.000265060135129246350.000215920343379610230.000240880807469816174.73323196901e-057.90225897986e-050.00.00.000141996959070408760.000153633581508034498.343418788766256e-050.00.0002316875288960.000231708560930999980.000215230593751685960.000243921701146049720.000235910291788933060.00025524309308742240.000207923293624809840.000231959296082045230.0002316875288960.000231708560930999980.000215230593751685960.000243921701146049720.000235910291788933060.00025524309308742240.000207923293624809840.000231959296082045230.0002316875288960.000231708560930999980.000215230593751685960.000243921701146049720.000235910291788933060.00025524309308742240.000207923293624809840.000231959296082045230.00.00.00.00.00.00.00.00.00.00.00.00.00.00.00.00.00.00.00.00.00.00.00.00.00.00.00.00.00.00.00.00.00.00.00.00.00.00.00.00.00.00.00.00.00.00.00.02.99327315814e-050.08.979819474408088e-050.00.00.00.00.02.99327315814e-050.08.979819474408088e-050.00.00.00.00.02.99327315814e-050.08.979819474408088e-050.00.00.00.00.09.127742831217e-050.00.00.000110509775236417350.000163322509700030560.00.00.09.127742831217e-050.00.00.000110509775236417350.000163322509700030560.00.00.08.25856482374e-050.00.08.443443501209415e-050.000163322509700030560.00.00.08.69178007477e-060.00.02.6075340224323195e-050.00.00.00.00.00127623114731820.0001440107219350.00106015573083752130.00148283089499620660.00128570681611775840.00018554209459047250.000201525653820969574.496441739436569e-050.00127623114731820.0001440107219350.00106015573083752130.00148283089499620660.00128570681611775840.00018554209459047250.000201525653820969574.496441739436569e-057.96343739222e-050.00.000129138356251011540.000109764765515722410.00.00.00.00.0001104140959490.00.000124399334003268027.049113381743641e-050.000136351820024796160.00.00.00.00.00.00.00.00.00.00.03.22837545634e-050.00.09.685126369034327e-050.00.00.00.00.000211142804160.0001440107219350.000250329736732730140.000177312313525397730.000205786362222038530.00018554209459047250.000201525653820969574.496441739436569e-052.20509619921e-050.00.00.06.615288597642188e-050.00.00.00.0003096717014750.00.000347680189906569640.00029552052254232950.00028581439197505350.00.00.00.000292285145687000050.00.00.0005910410450846590.00028581439197505350.00.00.00.000173982199982999980.00.000208608113943941760.000141849850820318160.000171488635185032120.00.00.04.47661095865e-050.00.00.00.00013429832875936250.00.00.00.00068658016225900010.00058625944438400010.00089731993029367630.00055908042128466250.00060334013519658020.00040767318917112470.00051638310267780340.00083472204130161770.00068658016225900010.00058625944438400010.00089731993029367630.00055908042128466250.00060334013519658020.00040767318917112470.00051638310267780340.00083472204130161770.0001776058287870.00.000199404814799356080.000169489711458100760.00016392296010333950.00.00.00.000259159622028000030.0003109202663850.000393206634452607640.000195344074222895750.000188928157407238760.000204410782175944240.00022201978810784780.0005063302288708570.0002498147114440.0002753391779990.000304708481041712650.0001942466356036660.00025048901768600190.000203262406995180460.00029436331456995550.000328391812430760670.02.9304625276000003e-050.00.00.00.08.791387582793975e-050.00.02.9304625276000003e-050.00.00.00.08.791387582793975e-050.00.02.9304625276000003e-050.00.00.00.08.791387582793975e-050.00.014261122329349080.0082955508869978920.0272796994064960360.0106820889622686250.0048215786193275460.0066620254051158540.010611908118486250.0076127191374101173.26342416519e-050.09.790272495564052e-050.00.00.00.00.03.26342416519e-050.09.790272495564052e-050.00.00.00.00.03.26342416519e-050.09.790272495564052e-050.00.00.00.00.02.94345746882e-050.08.830372406454843e-050.00.00.00.00.02.94345746882e-050.08.830372406454843e-050.00.00.00.00.02.94345746882e-050.08.830372406454843e-050.00.00.00.00.00.00294872320172999980.001720050155260.0051544778839230820.00256556344056440170.00112612828070992850.0013010186255958640.00233272845861259240.00152640338158099270.00294872320172999980.001720050155260.0051544778839230820.00256556344056440170.00112612828070992850.0013010186255958640.00233272845861259240.00152640338158099270.00294872320172999980.001720050155260.0051544778839230820.00256556344056440170.00112612828070992850.0013010186255958640.00233272845861259240.00152640338158099275.2152028486e-050.00.000156456085457956330.00.00.00.00.05.2152028486e-050.00.000156456085457956330.00.00.00.00.05.2152028486e-050.00.000156456085457956330.00.00.00.00.00.00.00.00.00.00.00.00.00.00.00.00.00.00.00.00.00.00.00.00.00.00.00.00.07.721826541210001e-060.02.31654796236154e-050.00.00.00.00.07.721826541210001e-060.02.31654796236154e-050.00.00.00.00.07.721826541210001e-060.02.31654796236154e-050.00.00.00.00.00.0002037544577217.83489529267e-050.00055851600300780745.2747370156296803e-050.05.5195588779774416e-050.000179851270000407580.00.0002037544577217.83489529267e-050.00055851600300780745.2747370156296803e-050.05.5195588779774416e-050.000179851270000407580.00.0002037544577217.83489529267e-050.00055851600300780745.2747370156296803e-050.05.5195588779774416e-050.000179851270000407580.00.002137275802390.001443377744960.0036129719894945940.0018684676377114540.00093038777997657870.00090984125035937930.00166105013221617750.00175924185230764650.002137275802390.001443377744960.0036129719894945940.0018684676377114540.00093038777997657870.00090984125035937930.00166105013221617750.00175924185230764650.002137275802390.001443377744960.0036129719894945940.0018684676377114540.00093038777997657870.00090984125035937930.00166105013221617750.00175924185230764650.00.00.00.00.00.00.00.00.00.00.00.00.00.00.00.00.00.00.00.00.00.00.00.01.09306952087e-050.03.279208562601261e-050.00.00.00.00.01.09306952087e-050.03.279208562601261e-050.00.00.00.00.01.09306952087e-050.03.279208562601261e-050.00.00.00.00.02.85463734871e-050.08.563912046119714e-050.00.00.00.00.02.85463734871e-050.08.563912046119714e-050.00.00.00.00.02.85463734871e-050.08.563912046119714e-050.00.00.00.00.00.00.00.00.00.00.00.00.00.00.00.00.00.00.00.00.00.00.00.00.00.00.00.00.00.0003201258151950.000163782283672999980.00086429589528498348.831647800115595e-057.765072300262686e-060.000142824113216629780.000287442546986022346.108019081784261e-050.0003201258151950.000163782283672999980.00086429589528498348.831647800115595e-057.765072300262686e-060.000142824113216629780.000287442546986022346.108019081784261e-050.0003201258151950.000163782283672999980.00086429589528498348.831647800115595e-057.765072300262686e-060.000142824113216629780.000287442546986022346.108019081784261e-052.00881887502e-050.06.0264566250472073e-050.00.00.00.00.02.00881887502e-050.06.0264566250472073e-050.00.00.00.00.02.00881887502e-050.06.0264566250472073e-050.00.00.00.00.04.84961638282e-050.00.00014548849148450870.00.00.00.00.04.84961638282e-050.00.00014548849148450870.00.00.00.00.04.84961638282e-050.00.00014548849148450870.00.00.00.00.00.00.00.00.00.00.00.00.00.00.00.00.00.00.00.00.00.00.00.00.00.00.00.00.04.03745924337e-051.73996554478e-057.67557505571081e-052.255440387309364e-052.1813622870894493e-052.360124490876851e-050.02.8597721434772694e-054.03745924337e-051.73996554478e-057.67557505571081e-052.255440387309364e-052.1813622870894493e-052.360124490876851e-050.02.8597721434772694e-052.36344351419e-051.73996554478e-052.6535278681714708e-052.255440387309364e-052.1813622870894493e-052.360124490876851e-050.02.8597721434772694e-051.67401572918e-050.05.022047187539339e-050.00.00.00.00.00.00.00.00.00.00.00.00.00.00.00.00.00.00.00.00.00.00.00.00.00.00.00.00.07.2086801735e-060.02.162604052050433e-050.00.00.00.00.07.2086801735e-060.02.162604052050433e-050.00.00.00.00.00.00.00.00.00.00.00.00.07.2086801735e-060.02.162604052050433e-050.00.00.00.00.00.0043029357973020.002795904028210.0081115245248399480.00337099605981610930.00142628680726318940.0025271632493480470.00335963735130026530.00250091148397369940.0043029357973020.002795904028210.0081115245248399480.00337099605981610930.00142628680726318940.0025271632493480470.00335963735130026530.00250091148397369941.78285911766e-051.0386237138000002e-052.8911572295002588e-052.457420123486322e-050.00.00.03.1158711414006076e-050.000959110078190.0005395308310240.00216033148508048160.00054696340782410820.000170035341666514870.0005314680336574550.00066605936432354330.000421065095091305740.00.00.00.00.00.00.00.00.003107524069790.002176976724590.0053901226956943950.0027381536615063360.00119429585218147330.00191016162598576440.00262390156411308920.0019968669836628241.65702808138e-050.02.728275132063625e-050.02.242809112077885e-050.00.00.02.40417152595e-050.07.212514577849051e-050.00.00.00.00.08.43224475746e-053.12482309682e-050.000192333722075974634.086985950053493e-051.9763761147211147e-052.138339742620693e-054.645094857575539e-052.5910346902781645e-050.00.00.00.00.00.00.00.09.35386144975e-053.77620044898e-050.00024041715259496842.0434929750267465e-051.9763761147211147e-056.415019227862079e-052.3225474287877688e-052.5910346902781645e-053.64503424902e-050.00.000109351027470614640.00.00.00.00.03.64503424902e-050.00.000109351027470614640.00.00.00.00.00.00.00.00.00.00.00.00.00.00.00.00.00.00.00.00.03.64503424902e-050.00.000109351027470614640.00.00.00.00.06.45691781255e-050.00.000193707534376517310.00.00.00.00.06.45691781255e-050.00.000193707534376517310.00.00.00.00.06.45691781255e-050.00.000193707534376517310.00.00.00.00.05.749545482522e-050.06.40979596560676e-050.000108388404819600480.00.00.00.05.749545482522e-050.06.40979596560676e-050.000108388404819600480.00.00.00.05.02982534418e-050.04.250635550581885e-050.000108388404819600480.00.00.00.07.1972013834199995e-060.02.159160415024875e-050.00.00.00.00.09.27147173084e-050.00.000278144151925255660.00.00.00.00.09.27147173084e-050.00.000278144151925255660.00.00.00.00.09.27147173084e-050.00.000278144151925255660.00.00.00.00.02.728417588815e-059.481317726710001e-068.18525276645049e-050.00.02.844395318014318e-050.00.02.728417588815e-059.481317726710001e-068.18525276645049e-050.00.02.844395318014318e-050.00.05.96416424295e-060.01.789249272886239e-050.00.00.00.00.02.13200116452e-059.481317726710001e-066.396003493564252e-050.00.02.844395318014318e-050.00.02.65482670707e-050.07.964480121207761e-050.00.00.00.00.02.65482670707e-050.07.964480121207761e-050.00.00.00.00.02.65482670707e-050.07.964480121207761e-050.00.00.00.00.05.45392686388e-050.08.979819474408088e-050.07.381961117236481e-050.00.00.05.45392686388e-050.08.979819474408088e-050.07.381961117236481e-050.00.00.05.45392686388e-050.08.979819474408088e-050.07.381961117236481e-050.00.00.09.68877480687e-060.02.0599358004339106e-050.08.466966416275796e-060.00.00.09.68877480687e-060.02.0599358004339106e-050.08.466966416275796e-060.00.00.09.68877480687e-060.02.0599358004339106e-050.08.466966416275796e-060.00.00.00.0009315675470480.0003193523486530.0023844084210329780.00026859810205648170.000141696118055429060.000204410782175944240.00044403957621569560.000309606687567136560.0009315675470480.0003193523486530.0023844084210329780.00026859810205648170.000141696118055429060.000204410782175944240.00044403957621569560.000309606687567136560.0009315675470480.0003193523486530.0023844084210329780.00026859810205648170.000141696118055429060.000204410782175944240.00044403957621569560.000309606687567136561.27200069478e-050.03.816002084340398e-050.00.00.00.00.01.27200069478e-050.03.816002084340398e-050.00.00.00.00.01.27200069478e-050.03.816002084340398e-050.00.00.00.00.03.16072899915e-052.81124385743e-059.482186997451898e-050.00.08.4337315722942e-050.00.03.16072899915e-052.81124385743e-059.482186997451898e-050.00.08.4337315722942e-050.00.03.16072899915e-052.81124385743e-059.482186997451898e-050.00.08.4337315722942e-050.00.01.48842232781e-050.04.4652669834323417e-050.00.00.00.00.01.48842232781e-050.04.4652669834323417e-050.00.00.00.00.01.48842232781e-050.04.4652669834323417e-050.00.00.00.00.07.38497422224e-057.55430651578e-062.3459389976394835e-050.000159519728431153643.85701082596093e-050.02.266291954734087e-050.07.38497422224e-057.55430651578e-062.3459389976394835e-050.000159519728431153643.85701082596093e-050.02.266291954734087e-050.07.38497422224e-057.55430651578e-062.3459389976394835e-050.000159519728431153643.85701082596093e-050.02.266291954734087e-050.00.00.00.00.00.00.00.00.00.00.00.00.00.00.00.00.00.00.00.00.00.00.00.00.00.002443622465610.001641793249210.0041378839502964980.00214633919423936040.00104664425230301270.00132115262971306680.0022549427618465290.00134928435608288290.002443622465610.001641793249210.0041378839502964980.00214633919423936040.00104664425230301270.00132115262971306680.0022549427618465290.00134928435608288290.002443622465610.001641793249210.0041378839502964980.00214633919423936040.00104664425230301270.00132115262971306680.0022549427618465290.00134928435608288292.05447384945e-050.06.163421548343734e-050.00.00.00.00.02.05447384945e-050.06.163421548343734e-050.00.00.00.00.02.05447384945e-050.06.163421548343734e-050.00.00.00.00.05.779849704330001e-060.01.7339549112987486e-050.00.00.00.00.05.779849704330001e-060.01.7339549112987486e-050.00.00.00.00.05.779849704330001e-060.01.7339549112987486e-050.00.00.00.00.07.63917036978e-050.00.000229175111093344430.00.00.00.00.07.63917036978e-050.00.000229175111093344430.00.00.00.00.07.63917036978e-050.00.000229175111093344430.00.00.00.00.09.04621436141e-057.03944058406e-050.000240788288242715293.059814259951553e-050.06.40366521152958e-056.955310176121957e-057.759346364514432e-059.04621436141e-057.03944058406e-050.000240788288242715293.059814259951553e-050.06.40366521152958e-056.955310176121957e-057.759346364514432e-059.04621436141e-057.03944058406e-050.000240788288242715293.059814259951553e-050.06.40366521152958e-056.955310176121957e-057.759346364514432e-050.004405239664280490.004288348734254630.0056495869437079530.0039172013339266540.00364893071520315440.0042432965983437030.0043171076927661280.0043046419116695488.95155387441e-050.00.000147386167460393620.00.000121160448772033540.00.00.08.95155387441e-050.00.000147386167460393620.00.000121160448772033540.00.00.08.95155387441e-050.00.000147386167460393620.00.000121160448772033540.00.00.08.711810306389999e-060.00.00.02.613543091917253e-050.00.00.08.711810306389999e-060.00.00.02.613543091917253e-050.00.00.08.711810306389999e-060.00.00.02.613543091917253e-050.00.00.04.16383134365e-050.0004104759127110.06.350027757107908e-056.141466273844125e-050.000431909283550824330.000396944469647364330.000402573984935780874.16383134365e-050.0004104759127110.06.350027757107908e-056.141466273844125e-050.000431909283550824330.000396944469647364330.000402573984935780874.16383134365e-050.0004104759127110.06.350027757107908e-056.141466273844125e-050.000431909283550824330.000396944469647364330.000402573984935780870.02.72547709568e-050.00.00.08.176431287037769e-050.00.00.02.72547709568e-050.00.00.08.176431287037769e-050.00.00.02.72547709568e-050.00.00.08.176431287037769e-050.00.00.00063127070785120.000304777820868300040.00078016636277507090.0006685795551214640.00044506620565723530.00025782615445257780.00044317248109922360.00021333482705355980.00063127070785120.000304777820868300040.00078016636277507090.0006685795551214640.00044506620565723530.00025782615445257780.00044317248109922360.00021333482705355980.0001798718368650.00.000231391252668194830.000118006488523728830.000190217769403192650.00.00.00.05.68574093712e-050.00.00.08.176431287037769e-058.880791524313912e-050.00.0002653851735030.0002254132508470.00029692395780342080.000336505120559148950.000162726442146380810.000176061841582200120.00028684308390575960.00021333482705355989.98115401447e-050.00.000112062209969886089.525041635661859e-059.212199410766188e-050.00.00.08.62021573385e-052.25071606501e-050.00013978894233356920.000118817529681967530.00.06.752148195032484e-050.06.14944553576e-050.00.00018448336607287370.00.00.00.00.06.14944553576e-050.00.00018448336607287370.00.00.00.00.06.14944553576e-050.00.00018448336607287370.00.00.00.00.00.00.00.00.00.00.00.00.00.00.00.00.00.00.00.00.00.00.00.00.00.00.00.00.00.00.00.00.00.00.00.00.00.00.00.00.00.00.00.00.00.00.00.00.00.00.00.00.00.000144634959001000020.00.000433904877003398870.00.00.00.00.00.000144634959001000020.00.000433904877003398870.00.00.00.00.00.000144634959001000020.00.000433904877003398870.00.00.00.00.00.000198222726344300046.28322895733e-050.00031053340540654840.000209324295190532037.48104784364234e-055.7157517290903846e-056.208136255148351e-056.925798887757748e-050.000198222726344300046.28322895733e-050.00031053340540654840.000209324295190532037.48104784364234e-055.7157517290903846e-056.208136255148351e-056.925798887757748e-050.00.00.00.00.00.00.00.00.000137173192076000030.00.00018200707927994920.000154702018512091987.48104784364234e-050.00.00.06.10495342683e-056.28322895733e-050.00012852632612659925.462227667844005e-050.05.7157517290903846e-056.208136255148351e-056.925798887757748e-050.00012205103541030.0001237475189390.00017458618978141050.00.00019156691645049880.00025880335082362040.000112439205994532340.00.00012205103541030.0001237475189390.00017458618978141050.00.00019156691645049880.00025880335082362040.000112439205994532340.01.60154616193e-050.00.00.04.804638485787537e-050.00.00.00.000106035573791000010.0001237475189390.00017458618978141050.00.000143520531592623420.00025880335082362040.000112439205994532340.00.00055856538932300010.00058234251248599990.00073727926795790280.00049043831400641910.00044797858600345270.00059873512793379420.00052644408385856110.00062184832566675930.00055856538932300010.00058234251248599990.00073727926795790280.00049043831400641910.00044797858600345270.00059873512793379420.00052644408385856110.00062184832566675930.00055856538932300010.00058234251248599990.00073727926795790280.00049043831400641910.00044797858600345270.00059873512793379420.00052644408385856110.00062184832566675933.54975412261e-050.05.8446238820500914e-050.04.804638485787537e-050.00.00.03.54975412261e-050.05.8446238820500914e-050.04.804638485787537e-050.00.00.03.54975412261e-050.05.8446238820500914e-050.04.804638485787537e-050.00.00.00.00.000110707845857000010.00.00.00.00.00.000332123537572019270.00.000110707845857000010.00.00.00.00.00.000332123537572019270.00.000110707845857000010.00.00.00.00.00.000332123537572019270.001347451114910.00141846607950.00149219679591479880.00132347946937617420.0012266770794336030.0012406463023453840.00144153517924569130.00157321675692009140.001347451114910.00141846607950.00149219679591479880.00132347946937617420.0012266770794336030.0012406463023453840.00144153517924569130.00157321675692009140.001347451114910.00141846607950.00149219679591479880.00132347946937617420.0012266770794336030.0012406463023453840.00144153517924569130.00157321675692009140.00.000122669238140.00.00.00.00013512869634040010.000110077037801369930.00012280198027872980.00.000122669238140.00.00.00.00013512869634040010.000110077037801369930.00012280198027872980.00.000122669238140.00.00.00.00013512869634040010.000110077037801369930.00012280198027872980.00081108356478100010.00076757854426300010.00086550174934188580.00079696226026043130.00077078668474123470.00080187740348275990.00080127886293178050.00069957936637510440.00081108356478100010.00076757854426300010.00086550174934188580.00079696226026043130.00077078668474123470.00080187740348275990.00080127886293178050.00069957936637510440.00081108356478100010.00076757854426300010.00086550174934188580.00079696226026043130.00077078668474123470.00080187740348275990.00080127886293178050.00069957936637510440.0003551025075890.000333375100867999950.000465102523173168260.00036491716240055470.000235287837193183880.00035003323913506010.000380186919477554660.00026990514398992590.0003551025075890.000333375100867999950.000465102523173168260.00036491716240055470.000235287837193183880.00035003323913506010.000380186919477554660.00026990514398992590.0003551025075890.000333375100867999950.000465102523173168260.00036491716240055470.000235287837193183880.00035003323913506010.000380186919477554660.00026990514398992590.09.80507003933e-060.00.00.02.941521011800173e-050.00.00.09.80507003933e-060.00.00.02.941521011800173e-050.00.00.09.80507003933e-060.00.00.02.941521011800173e-050.00.00.01.43160300529e-050.00.00.00.04.294809015856728e-050.00.01.43160300529e-050.00.00.00.04.294809015856728e-050.00.01.43160300529e-050.00.00.00.04.294809015856728e-050.00.00.00.00.00.00.00.00.00.00074262774882519990.00128641468639470.00068103090347336890.00067613549328010350.00087071684972183190.00200677212175272030.00103844972812931580.00081402220930062960.0001355694828520.000108054780440.000125939883031172260.000142728178069979590.00013804038745544380.000112014577229542220.000121664094412040439.048566967906407e-050.0001355694828520.000108054780440.000125939883031172260.000142728178069979590.00013804038745544380.000112014577229542220.000121664094412040439.048566967906407e-050.0001355694828520.000108054780440.000125939883031172260.000142728178069979590.00013804038745544380.000112014577229542220.000121664094412040439.048566967906407e-050.0001700275878330.0001260602357229.10035396399746e-050.000232053027768137880.00018702619609105850.000202352955509743420.00017582775165587950.00.0001700275878330.0001260602357229.10035396399746e-050.000232053027768137880.00018702619609105850.000202352955509743420.00017582775165587950.00.0001700275878330.0001260602357229.10035396399746e-050.000232053027768137880.00018702619609105850.000202352955509743420.00017582775165587950.00.00027098210487090.00037653224633140.000207695168149876380.000239516413067263720.00036573473339452610.00030341336469283420.000458536204982544340.00036764716931728680.00027098210487090.00037653224633140.000207695168149876380.000239516413067263720.00036573473339452610.00030341336469283420.000458536204982544340.00036764716931728680.0002587155749640.0003621061167340.000207695168149876380.00022697528098984170.00034147627575137680.00029029014799165820.000444282486268329960.000351745715940606861.22665299069e-051.44261295974e-050.01.2541132077422035e-052.4258457643149265e-051.3123216701175965e-051.4253718714214386e-051.590145337667992e-050.00.00.00.00.00.00.00.00.00.00.00.00.00.00.00.00.00.00.00.00.00.00.00.07.343807879100001e-056.71920461363e-056.773491951371282e-056.183787437472243e-059.074144248458684e-055.801408693023098e-056.785877704979918e-057.57032744289968e-057.343807879100001e-056.71920461363e-056.773491951371282e-056.183787437472243e-059.074144248458684e-055.801408693023098e-056.785877704979918e-057.57032744289968e-057.343807879100001e-056.71920461363e-056.773491951371282e-056.183787437472243e-059.074144248458684e-055.801408693023098e-056.785877704979918e-057.57032744289968e-052.67270579626e-050.08.018117388778314e-050.00.00.00.00.02.67270579626e-050.08.018117388778314e-050.00.00.00.00.02.67270579626e-050.08.018117388778314e-050.00.00.00.00.00.00.000113474597211000020.00.00.06.737562094067435e-050.000109769560042148210.000163278610649931250.00.000113474597211000020.00.00.06.737562094067435e-050.000109769560042148210.000163278610649931250.00.000113474597211000020.00.00.06.737562094067435e-050.000109769560042148210.000163278610649931250.00.0003890398757540.00.00.00.00116711962726264930.00.00.00.0003890398757540.00.00.00.00116711962726264930.00.00.00.0003890398757540.00.00.00.00116711962726264930.00.00.00.00.00.00.00.00.00.00.00.00.00.00.00.00.00.00.00.00.00.00.00.00.00.06.58834365157e-050.000106060904799999990.000108476219250849720.08.91740902962167e-059.648188918704568e-050.000104793339986904160.000116907485225350786.58834365157e-050.000106060904799999990.000108476219250849720.08.91740902962167e-059.648188918704568e-050.000104793339986904160.000116907485225350786.58834365157e-050.000106060904799999990.000108476219250849720.08.91740902962167e-059.648188918704568e-050.000104793339986904160.000116907485225350780.0067703248011441590.0085429086165278690.0079665644273300350.0050613102829458550.0072830996931508010.009077180105443940.0091301573549577750.007421388389166030.0003471673978380.0003355932123290.00039114021364489090.000221640391906747180.000428721587962580330.000347891427357135870.000377860600914317860.000281027608714785550.0003471673978380.0003355932123290.00039114021364489090.000221640391906747180.000428721587962580330.000347891427357135870.000377860600914317860.000281027608714785550.0003471673978380.0003355932123290.00039114021364489090.000221640391906747180.000428721587962580330.000347891427357135870.000377860600914317860.000281027608714785550.0001463902588790.00.00016435790795583290.000139700610656373940.000135112258024570730.00.00.00.0001463902588790.00.00016435790795583290.000139700610656373940.000135112258024570730.00.00.00.0001463902588790.00.00016435790795583290.000139700610656373940.000135112258024570730.00.00.00.00.00.00.00.00.00.00.00.0001035029086740.00.00.00.00031050872602179160.00.00.00.0001035029086740.00.00.00.00031050872602179160.00.00.04.80463848579e-050.00.00.00.000144139154573626140.00.00.05.54565238161e-050.00.00.00.000166369571448165460.00.00.00.00152705729184710020.00282639221576860.0024307288466490950.00063572733109462040.0015147156977980530.0027529862285651010.0029895939826822430.00273659643605689670.00152705729184710020.00282639221576860.0024307288466490950.00063572733109462040.0015147156977980530.0027529862285651010.0029895939826822430.00273659643605689670.0003117244495840.0006741159203380.00045964499682563449.767203711144788e-050.00037785631481447750.00061323234652783270.00066605936432354330.00074305605016112775.83205479843e-050.0002445952302910.000174961643952983420.00.00.00031123190060337320.00042255379026977490.00.00054398129262099990.0009543717872390.00102470678451527590.000174195650602929340.000433041442745006570.00098971709547280090.00098991693477329130.00088348133147236660.0002076270658940.000402389377150.00026076014242992720.000147760261271164760.00021436079398129010.00030923682431745410.000335876089701615850.00056205521742957110.00.00.00.00.00.00.00.00.0003526127206070.0004971604912490.00042373523144863170.000216099382109078430.000418003548263515660.00045225885556427670.00049121878118861320.00054800383699383165.27912151568e-055.37594095016e-058.692004747664241e-050.07.145359799376338e-057.730920607936352e-058.396902242540398e-050.00.000244734278154000030.0001276867522430.00064630731202841828.789552243394357e-050.08.047822024658243e-050.000274720386047651532.7861650435021632e-050.000244734278154000030.0001276867522430.00064630731202841828.789552243394357e-050.08.047822024658243e-050.000274720386047651532.7861650435021632e-050.000244734278154000030.0001276867522430.00064630731202841828.789552243394357e-050.08.047822024658243e-050.000274720386047651532.7861650435021632e-050.000210296892977999980.0002673167164330.000207015685593224640.000211150541297420170.000212724452042501650.00028539490122122290.000259983477066747040.00025657177100983480.000210296892977999980.0002673167164330.000207015685593224640.000211150541297420170.000212724452042501650.00028539490122122290.000259983477066747040.00025657177100983480.000210296892977999980.0002673167164330.000207015685593224640.000211150541297420170.000212724452042501650.00028539490122122290.000259983477066747040.00025657177100983480.00044291925671970.00053242831563730.00051603672626635040.000478371874596755160.000334349169295872950.00071288267780410140.00059694883213156260.00028745343697694520.00044291925671970.00053242831563730.00051603672626635040.000478371874596755160.000334349169295872950.00071288267780410140.00059694883213156260.00028745343697694528.17751815112e-058.86144564267e-058.32724303358621e-057.077973620768178e-059.127337798998638e-059.875321308807133e-050.000107260327519860965.9829828672134485e-054.45347190618e-051.68556759586e-058.52800465808567e-054.832411060440609e-050.05.05670278758101e-050.00.00.000247172761240.000426958183251999960.0002112075919993180.00028723490041497450.000243075791305886580.000563562436840220.00048968850461170170.00022762360830481072.40110424566e-050.00.07.203312736969282e-050.00.00.00.04.54255524501e-050.00.00013627665735031370.00.00.00.00.09.05780053865e-060.02.717340161594432e-050.00.00.00.00.09.05780053865e-060.02.717340161594432e-050.00.00.00.00.09.05780053865e-060.02.717340161594432e-050.00.00.00.00.09.39853821487e-057.42863941306e-050.000281956146445970460.00.00.00.000222859182391809420.09.39853821487e-057.42863941306e-050.000281956146445970460.00.00.00.000222859182391809420.09.39853821487e-057.42863941306e-050.000281956146445970460.00.00.00.000222859182391809420.01.96602243736e-052.70895100761e-051.904428006510704e-052.428082945045825e-051.565556360537512e-054.2346334790662604e-051.839770716062222e-052.0524488276922542e-051.96602243736e-052.70895100761e-051.904428006510704e-052.428082945045825e-051.565556360537512e-054.2346334790662604e-051.839770716062222e-052.0524488276922542e-051.96602243736e-052.70895100761e-051.904428006510704e-052.428082945045825e-051.565556360537512e-054.2346334790662604e-051.839770716062222e-052.0524488276922542e-050.00.00.00.00.00.00.00.00.00.00.00.00.00.00.00.00.00.00.00.00.00.00.00.00.05.50244962296e-050.00.00.07.681679075401726e-054.171709394383128e-054.653960399098359e-050.05.50244962296e-050.00.00.07.681679075401726e-054.171709394383128e-054.653960399098359e-050.00.00.00.00.00.00.00.00.05.50244962296e-050.00.00.07.681679075401726e-054.171709394383128e-054.653960399098359e-050.00.00.00.00.00.00.00.00.00.00.00.00.00.00.00.00.00.00.00.00.00.00.00.00.000112218828349999999.61033268339e-059.224168303643685e-050.00013067233989966950.000113742462112521296.836868564841671e-050.000103961646812404920.000115979648041022610.000112218828349999999.61033268339e-059.224168303643685e-050.00013067233989966950.000113742462112521296.836868564841671e-050.000103961646812404920.000115979648041022610.000112218828349999999.61033268339e-059.224168303643685e-050.00013067233989966950.000113742462112521296.836868564841671e-050.000103961646812404920.000115979648041022618.16479703674e-058.92865670234e-059.662371073412979e-058.212803120535049e-056.619216916286868e-058.593992504784353e-057.778603027531484e-050.000104133745747046428.16479703674e-058.92865670234e-059.662371073412979e-058.212803120535049e-056.619216916286868e-058.593992504784353e-057.778603027531484e-050.000104133745747046428.16479703674e-058.92865670234e-059.662371073412979e-058.212803120535049e-056.619216916286868e-058.593992504784353e-057.778603027531484e-050.000104133745747046426.23426547419e-050.00.000187027964225602960.00.00.00.00.06.23426547419e-050.00.000187027964225602960.00.00.00.00.06.23426547419e-050.00.000187027964225602960.00.00.00.00.00.0008478466400140.0008095384000540.00101306813955534970.00076172962275997020.0007687421577260060.00086639627502734970.00101631471970057920.00054590420543446830.0008478466400140.0008095384000540.00101306813955534970.00076172962275997020.0007687421577260060.00086639627502734970.00101631471970057920.00054590420543446830.0008478466400140.0008095384000540.00101306813955534970.00076172962275997020.0007687421577260060.00086639627502734970.00101631471970057920.00054590420543446831.08650059346e-050.03.25950178037409e-050.00.00.00.00.01.08650059346e-050.03.25950178037409e-050.00.00.00.00.01.08650059346e-050.03.25950178037409e-050.00.00.00.00.04.12843010631e-050.00061628916676400010.00.00.000123852903189189840.00053601049548358710.00058218522214946760.00073067178265844244.12843010631e-050.00061628916676400010.00.00.000123852903189189840.00053601049548358710.00058218522214946760.00073067178265844244.12843010631e-050.00061628916676400010.00.00.000123852903189189840.00053601049548358710.00058218522214946760.00073067178265844240.00078181181742899990.001002861228040.00064955819910688470.00082816529670545030.00086771195647516250.000938820778017660.0010196956735252650.00105006723256303110.00078181181742899990.001002861228040.00064955819910688470.00082816529670545030.00086771195647516250.000938820778017660.0010196956735252650.00105006723256303110.00078181181742899990.001002861228040.00064955819910688470.00082816529670545030.00086771195647516250.000938820778017660.0010196956735252650.00105006723256303112.92073826739e-062.5977891542e-068.762214802168797e-060.00.07.79336746260466e-060.00.02.92073826739e-062.5977891542e-068.762214802168797e-060.00.07.79336746260466e-060.00.02.92073826739e-062.5977891542e-068.762214802168797e-060.00.07.79336746260466e-060.00.01.61615344533e-050.04.8484603360034134e-050.00.00.00.00.01.61615344533e-050.04.8484603360034134e-050.00.00.00.00.01.61615344533e-050.04.8484603360034134e-050.00.00.00.00.00.00.00.00.00.00.00.00.00.00.00.00.00.00.00.00.00.00.00.00.00.00.00.00.00.000104386365087000027.02995066973e-050.000258276712502023145.488238275786121e-050.08.614454391700507e-050.000124753976174885920.00.000104386365087000027.02995066973e-050.000258276712502023145.488238275786121e-050.08.614454391700507e-050.000124753976174885920.00.000104386365087000027.02995066973e-050.000258276712502023145.488238275786121e-050.08.614454391700507e-050.000124753976174885920.00.00030405851561010.0004894536921880.00037259692066538210.000241065281331478780.000298513344833550740.00056174076435802420.0005690688089599850.000337551503243885640.00030405851561010.0004894536921880.00037259692066538210.000241065281331478780.000298513344833550740.00056174076435802420.0005690688089599850.000337551503243885645.50090220805e-050.000178457381559000038.50127110116377e-053.6129468273200156e-054.3884886956799546e-050.000160900393409517260.000133697967580112770.000240773783686476078.88415354559e-050.00.000111143667265214879.44696752389414e-056.091126386353599e-050.00.00.00.00.00.00.00.00.00.00.02.99327315814e-050.000218837322912000038.979819474408088e-050.00.00.000268310303383883730.00029142394579346549.67777195574096e-058.94445990673e-050.08.664234764444864e-050.000110466137819337247.122531173819224e-050.00.00.04.0830627424999996e-059.2158987717e-050.00.00.000122491882275022940.000132530067564623170.000143946895586406810.00.00.00.00.00.00.00.00.00.00126000873767561980.00112066132692586980.00052356874127344810.00116390022684975650.0020925572449007570.00162716868974262850.00085431001502108620.00088050527601674260.00126000873767561980.00112066132692586980.00052356874127344810.00116390022684975650.0020925572449007570.00162716868974262850.00085431001502108620.00088050527601674260.00.00.00.00.00.00.00.02.7728261908000004e-059.25861686446e-050.00.08.318478572408273e-050.000180003524602697149.775498133106732e-050.06.21118225767e-050.00.00.000106715744251396777.96197234787649e-050.00.00.00.00.00.00.00.00.00.00.00.00.0002056868073340.00.00.00.00028714847972335020.000155942470218607380.000173969472061533885.010911827919999e-060.01.5032735483765204e-050.00.00.00.00.00.000120193052793000010.00.000160467779956878266.81970436636145e-050.0001319143347577170.00.00.04.4498049049999996e-050.00.00.00.0001334941471500250.00.00.00.02.06404458378e-050.00.00.00.00.06.19213375134273e-052.19857224596e-050.0002095056220930.00.06.59571673788585e-050.00021408703221977590.000155019733708438080.000259410100352109660.00.000379536841688000040.00.00.00.00060041682348310140.00021745458212713560.000320739119453795550.00.00.00.00.00.00.00.00.0002849943008560.00.000105933807862157920.000292239661477920360.0004568094332265760.00.00.00.03.63479088257e-060.00.00.01.090437264772216e-050.00.00.05.72109000533e-050.00.00.07.986911356543515e-054.337472681577159e-054.838885977870479e-050.00.00.00.00.00.00.00.04.42331797104e-050.00.00.00.000132699539131274820.00.00.00.00.00.00.00.00.00.00.00.09.98389033601e-050.00.00.00.00014357423986167510.000155942470218607380.00.00.00.00.00.00.00.00.01.59437845754e-050.00.02.431497970284989e-052.3516374023263888e-050.00.00.00.00.00.00.00.00.00.00.00.00.00.00.00.00.00.00.02.19857224596e-050.00.00.06.59571673788585e-050.00.00.00.00.00.00.00.00.00.00.00.00056886007693699990.00.000242134417970646670.0006724327977539750.00079201301508531150.00.00.00.00.00.00.00.00.00.00.04.2463852522e-050.00.00.00.000127391557566023810.00.00.00.02.82333990081e-050.00.00.03.98027595656129e-052.8821050601458788e-051.607638685717145e-050.00.00.00.00.00.00.00.00.00.00.00.00.00.00.00.00.02.37874480244e-050.00.00.07.136234407325863e-050.00.00.00.00.00.00.00.00.00.00.042749346370829140.051724623995933610.0474905927909572150.0408767392622622350.039880707059312710.0499948015575143250.0494307972317055560.0557482731985838453.99846389772e-050.00.07.992963941996501e-054.002427751176692e-050.00.00.03.99846389772e-050.00.07.992963941996501e-054.002427751176692e-050.00.00.01.28487183714e-050.00.03.8546155114216895e-050.00.00.00.02.71359206058e-050.00.04.138348430574812e-054.002427751176692e-050.00.00.00.00019195283784510.00.000162406891961450450.000237398327678203740.000176053293895606980.00.00.00.00019195283784510.00.000162406891961450450.000237398327678203740.000176053293895606980.00.00.06.40812309896e-050.00.000104706775338658054.44992292631307e-054.303768836689995e-050.00.00.03.31186792504e-050.00.09.935603775130043e-050.00.00.00.02.91789725433e-050.00.04.44992292631307e-054.303768836689995e-050.00.00.05.13923249256e-050.05.7700116622792396e-054.904383140064192e-054.743302675330675e-050.00.00.01.41816301362e-050.00.00.04.254489040850033e-050.00.00.02.56961624628e-050.02.8850058311396198e-052.452191570032096e-052.371651337665337e-050.00.00.02.56961624628e-050.02.8850058311396198e-052.452191570032096e-052.371651337665337e-050.00.00.00.00.00.00.00.00.00.00.02.56961624628e-050.02.8850058311396198e-052.452191570032096e-052.371651337665337e-050.00.00.00.00.00.00.00.00.00.00.00.00010645378029940.00.00.000177180344089707420.000142180996808655740.00.00.00.00010645378029940.00.00.000177180344089707420.000142180996808655740.00.00.03.70458523957e-050.00.05.649657048603359e-055.4640986701113175e-050.00.00.06.94079279037e-050.00.00.000120683773603673838.754001010754256e-050.00.00.00.0020927182450830.00.0022783767465088940.00218320394494885730.00181657404380560030.00.00.00.0020927182450830.00.0022783767465088940.00218320394494885730.00181657404380560030.00.00.00.0001743366710980.00.00017724872426609430.000175767108178771160.000169994180847907640.00.00.00.0010984742120.00.00113370841360496250.00120971056356620640.00095200365884228030.00.00.00.0002110534632010.00.000264719623985262340.000175004561071704920.00019343620454710780.00.00.00.000333768401282999960.00.00040337266883897210.000342857906015929230.000255074628993754860.00.00.00.0002750854975010.00.0002993273158136030.00027986380611624580.00024606537057454940.00.00.00.00026144063654120.00.00018576398365088380.000344606309220848160.00025395161675225280.00.00.00.00026144063654120.00.00018576398365088380.000344606309220848160.00025395161675225280.00.00.03.02865672013e-050.04.9487326300570116e-052.1031570034946812e-052.0340805268297602e-050.00.00.04.44550228749e-050.00.06.77958845832403e-056.55691840413358e-050.00.00.00.00.00.00.00.00.00.00.00.0001866990464650.00.00013627665735031370.000255778854602661060.00016804162744261940.00.00.00.00020520073951550.00.00029556840218330480.00025122664792516326.88071684384388e-050.00.00.00.00020520073951550.00.00029556840218330480.00025122664792516326.88071684384388e-050.00.00.07.45505947995e-050.08.370078645898896e-057.114382950093118e-056.88071684384388e-050.00.00.00.0001306501447160.00.000211867615724315850.000180082818424232030.00.00.00.00.00026837728109110.00116418130313127.639170369778149e-050.0003250391546037980.000403700984970885670.0007479035358253460.00123946895437359740.00150517141919472240.00026837728109110.00116418130313127.639170369778149e-050.0003250391546037980.000403700984970885670.0007479035358253460.00123946895437359740.00150517141919472241.74534731841e-050.000100356367257000020.02.6617321891803352e-052.574309766057064e-055.5705478745407436e-057.973943243213729e-050.000165624190593544620.07.87741504408e-050.00.00.05.289577258061714e-057.660331870387729e-050.000106823360037783960.0002509238079070.00090748790216299997.639170369778149e-050.00029842183271199460.0003779578873103150.00058622542519871930.00096782785224153170.00116841042904871340.00.00.00.00.00.00.00.00.05.16832911029e-050.00.00.03.536726143220149e-057.68279618672318e-054.2854650009292797e-050.02.58795921675e-050.00.00.01.7709597868400454e-053.847038912881944e-052.1458789505387438e-056.76519803852e-050.00.00.000119408479691351958.354746146405263e-050.00.00.06.76519803852e-050.00.00.000119408479691351958.354746146405263e-050.00.00.00.00.00.00.00.00.00.00.01.10079277738e-050.00.03.302378332134914e-050.00.00.00.05.66440526114e-050.00.08.638469637000282e-058.354746146405263e-050.00.00.00.001099940818269290.002113850412684210.00097284736454543690.0013227028938791210.00100427219638025460.0014722025428429040.00167988127202782170.0031894674231801960.001099940818269290.002113850412684210.00097284736454543690.0013227028938791210.00100427219638025460.0014722025428429040.00167988127202782170.0031894674231801962.23580943126e-055.65696563926e-051.904428006510704e-053.237443926727767e-051.565556360537512e-055.0815601748795114e-053.679541432124444e-058.209795310769015e-050.00.00.00.00.00.00.00.00.0001495306817790.0002982622872668.6366416600995e-050.000220228669665302890.000141996959070408760.000230450372262051710.00029201965760681890.00037231683192786880.04.31092001653e-050.00.00.06.775413566506016e-050.06.157346483076761e-055.27033630592e-064.5951607831000004e-050.00.01.5811008917768916e-053.4213435881931093e-050.00.000103641387611126580.0004863368076450.0008431037003720.00043187576420733620.00053866221307813440.0004884724456482770.00060140253050826060.00072794391659608850.00119996465401182937.97051964724e-050.000212738444884000029.535532634568364e-058.1049932342833e-056.271033072870373e-050.000118736502164085770.000128965080855894720.00039051375163179760.00.00.00.00.00.00.00.00.06.84149609231e-060.00.00.00.00.02.0524488276922542e-058.27907860254e-050.000154943454291000025.7052709984668505e-050.000113151616625604427.816803146582809e-056.765910882682025e-050.000110231423548636910.0002869398304970040.0001476198873870.0002808293775180.000171398520585963380.000161872196336388360.000109588945237625840.000135508271330120320.00027596560740933330.00043101425381537330.000120645941370.0001715011878720.000111754346755683090.000158314565647676519.18689117062672e-050.000165662584455778950.000107960171689805120.000240880807469816175.683086971969999e-060.00.01.7049260915903624e-050.00.00.00.08.51832991807e-060.00.02.555498975421475e-050.00.00.00.08.51832991807e-060.00.02.555498975421475e-050.00.00.00.00.00.00.00.00.00.00.00.08.51832991807e-060.00.02.555498975421475e-050.00.00.00.00.08.97931307131e-050.00.00.00.00.000100762826910484770.000168616565228871320.08.97931307131e-050.00.00.00.00.000100762826910484770.000168616565228871320.08.97931307131e-050.00.00.00.00.000100762826910484770.000168616565228871320.00071613826252960.000845507263626380.00036468038356548680.00093042722572269550.00085330717830149540.00075243796230216860.00080656911150991550.00097751471706618470.00071613826252960.000845507263626380.00036468038356548680.00093042722572269550.00085330717830149540.00075243796230216860.00080656911150991550.00097751471706618470.00.00.00.00.00.00.00.00.00.00.00.00.00.00.00.04.56438828127e-050.03.918938556750351e-053.3310116702748124e-056.443214616778662e-050.00.00.03.09819902665e-050.00.09.294597079960364e-050.00.00.00.00.00.00.00.00.00.00.00.00.0002421846619530.00.000171599838541705640.00029912623102951990.00025582791628872080.00.00.07.99719988225e-050.00.00.000121960850573024870.000117955145894466530.00.00.01.07752696673e-050.01.7473617791696147e-051.4852191210245941e-050.00.00.00.00.00.00.00.00.00.00.00.00.08.79765179414e-050.00.00.00.00.000124753976174885920.000139175577649227130.07.70841196073e-050.00.00.08.394595926947131e-054.558874999430285e-050.00010171764955801988.73682530489e-050.00.00.00013324046681099250.000128864292335573250.00.00.00.00.0001382450650580.00.00.08.954473754218768e-050.000149033951692783030.000176156505938460270.00.00.00.00.00.00.00.06.72769849983e-050.06.26788632034956e-055.327565660624431e-058.587643518510852e-050.00.00.00.00.00.00.00.00.00.00.00.00.00.00.00.00.00.00.00.00.00.00.00.00.00.00.04.36564408301e-060.00.01.3096932249035055e-050.00.00.00.00.00.0001086665655380.00.00.00.000128756969555665989.323250888514605e-050.000104010218172020269.18741545662e-050.04.22415183998636e-057.180872510374361e-050.000161572220195058060.00.00.08.73682530489e-060.00.01.3324046681099253e-051.2886429233557326e-050.00.00.00.00.00.00.00.00.00.00.00.00.00.00.00.00.00.00.03.25158647016e-050.03.149716006122233e-054.015784104233746e-052.589259300122436e-050.00.00.00.00.00.00.00.00.00.00.00.00.00.00.00.00.00.00.00.01.49783093387e-050.00.00.02.7981986423157094e-050.01.69529415930033e-050.04.71840224898e-060.00.00.01.4155206746925714e-050.00.01.44427323047e-050.0004138382838940.04.332819691410094e-050.00.00040805310276476080.000393959924762797630.0004395018241554540.00.00.00.00.00.00.00.05.85669245322e-052.41688099166e-050.08.831647800115595e-058.738429559544111e-057.250642974978381e-050.00.05.85669245322e-052.41688099166e-050.08.831647800115595e-058.738429559544111e-057.250642974978381e-050.00.04.36747918842e-050.00.08.831647800115595e-054.2707897651444776e-050.00.00.00.02.41688099166e-050.00.00.07.250642974978381e-050.00.01.4892132648e-050.00.00.04.4676397943996336e-050.00.00.01.82584132466e-050.00.00.05.477523973969084e-050.00.00.01.82584132466e-050.00.00.05.477523973969084e-050.00.00.01.82584132466e-050.00.00.05.477523973969084e-050.00.00.00.07.00490240554e-050.00.00.00.00.000210147072166251670.00.07.00490240554e-050.00.00.00.00.000210147072166251670.00.07.00490240554e-050.00.00.00.00.000210147072166251670.07.84326625187e-051.52854704035e-050.000129138356251011540.00.000106159631305019884.585641121057304e-050.00.07.84326625187e-051.52854704035e-050.000129138356251011540.00.000106159631305019884.585641121057304e-050.00.00.01.52854704035e-050.00.00.04.585641121057304e-050.00.07.84326625187e-050.00.000129138356251011540.00.000106159631305019880.00.00.00.00.0002159492180390.00.00.00.000117089671343502020.000317940958698131630.000212817024075274480.00.0002159492180390.00.00.00.000117089671343502020.000317940958698131630.000212817024075274480.00.0002159492180390.00.00.00.000117089671343502020.000317940958698131630.000212817024075274480.000349064327016999960.00111031604032030030.000331443039949911550.00040389563093304860.000311854310168547660.0012033060269046440.00088535145884676620.00124229063520933530.000349064327016999960.00111031604032030030.000331443039949911550.00040389563093304860.000311854310168547660.0012033060269046440.00088535145884676620.00124229063520933530.01.17094836964e-050.00.00.00.00.03.5128451089348186e-050.000349064327016999960.00093019357658600010.000331443039949911550.00040389563093304860.000311854310168547660.00093234840210561450.00082751848975907070.00103071383789304940.06.92855881185e-050.00.00.05.32460757102901e-055.7832969087695444e-059.67777195574096e-050.01.27183948243e-050.00.00.00.00.03.8155184473025705e-050.02.5259158903e-050.00.00.03.4262034512445194e-050.04.151544219650241e-050.06.11498381921e-050.00.00.00.00018344951457629420.00.00.00105853209777790.00457947013813140.00108408350890406150.0008818669914284590.00120964579299157240.004094459413329530.0044612668771789620.0051826841238815440.00105853209777790.00457947013813140.00108408350890406150.0008818669914284590.00120964579299157240.004094459413329530.0044612668771789620.0051826841238815440.00.00.00.00.00.00.00.02.73859327835e-050.0005110644505663.0747227678812275e-052.6134467979933906e-052.52761026916714e-050.00046490706240923370.00050495657023168120.00056332971905639550.05.69050104176e-050.00.00.08.022330032182289e-055.8089434582541115e-053.2402296348489685e-050.001012885789020.00236398728563000020.00102327081025771870.00085573252344852510.00115965403334418680.00200758455426358750.00233187577346177870.00275250152916185550.00.00033377807392400010.00.00.00.000294805772515972830.00034931113328968050.00035721731596634960.06.61980529352e-050.00.00.09.241560266958396e-055.0188381219781675e-055.599017491635572e-050.01.47610461143e-050.00.00.00.00.04.42831383429359e-050.00.00075672977197600010.00.00.00.00069992441932566620.0007602195423157110.00081004535428651711.82603759744e-050.0004382856309273.0065470967530408e-050.02.471565695571416e-050.00045459870182366310.00040662604207778780.000453632148878855570.03.77608156413e-050.00.00.00.00.00.000113282446923789531.54789125643e-050.04.643673769300074e-050.00.00.00.00.01.54789125643e-050.04.643673769300074e-050.00.00.00.00.01.54789125643e-050.04.643673769300074e-050.00.00.00.00.00.00115751532988410.00.0017078894075186590.00089705989135860190.00086759669077514390.00.00.00.00115751532988410.00.0017078894075186590.00089705989135860190.00086759669077514390.00.00.00.00060823032151199990.00.00080952402426007240.00051605822593212760.00049910871434449640.00.00.07.53307078131e-050.00.000225992123439270250.00.00.00.00.00.000473954300559000060.00.00067237325981931640.000381001665426474350.00036848797643064750.00.00.00.0006968886872730.00.00066375308982163290.00072536855533117240.00070154441666604030.00.00.00.0006968886872730.00.00066375308982163290.00072536855533117240.00070154441666604030.00.00.00.0006968886872730.00.00066375308982163290.00072536855533117240.00070154441666604030.00.00.00.00023155726929860.00.000342356094043696160.000179099037848617740.000173216676004921440.00.00.00.00023155726929860.00.000342356094043696160.000179099037848617740.000173216676004921440.00.00.00.000161136299284000020.00.00026329179429817890.000111896120185930590.0001082209833691950.00.00.07.04209700146e-050.07.906429974551728e-056.720291766268717e-056.499569263572645e-050.00.00.00.0002881774287650.0006046435813490.000305394761404419230.000207663069894609920.00035147445499634950.00065190465666922760.00076706836702125790.00039495772035591480.0002881774287650.0006046435813490.000305394761404419230.000207663069894609920.00035147445499634950.00065190465666922760.00076706836702125790.00039495772035591480.0002881774287650.0006046435813490.000305394761404419230.000207663069894609920.00035147445499634950.00065190465666922760.00076706836702125790.00039495772035591480.00134115181031250.00176952726378329980.00137718207759928490.00109140132093445840.00155487203240142630.00154852409194781650.00156330150649544070.00219675619290841470.00134115181031250.00176952726378329980.00137718207759928490.00109140132093445840.00155487203240142630.00154852409194781650.00156330150649544070.00219675619290841474.52536161002e-057.93869047902e-050.06.90137747254542e-056.67470735750125e-057.221698292443538e-057.84381287327127e-058.750560271358593e-055.24816242273e-050.0001199839709450.08.003680818854757e-057.740806449324365e-050.000167503279838620949.096644096085432e-050.000101482192035894770.08.24093961172e-050.00.00.07.221698292443538e-050.00.000175011205427171860.000478409295428000050.0004763214287410.00048716864933016340.00041408264835272520.00053397658860009990.000433301897546612260.00047062877239627620.00052503361628151560.000429224385270999970.000659755773220.00056327782811595980.000250550008242409840.00047384531945498260.00051267684875273140.00068653622166409730.00078005424924362320.02.91685342379e-050.00.00.00.00.08.750560271358593e-050.00.00.00.00.00.00.00.00.0003357828892860.000322501255731999970.00032673560015316180.000277718081425321660.000402894986278087460.000290608099960980930.000236731942741500350.00044016372449303760.00091769942509080.00078110828098940010.00087369862029793990.00068179821571546310.00119760143925994070.0010779955295835040.00062967162292691430.00063565769045756740.00091769942509080.00078110828098940010.00087369862029793990.00068179821571546310.00119760143925994070.0010779955295835040.00062967162292691430.00063565769045756740.000178921427519000040.00.000200881887501573620.000170745190802234830.000165137204252253150.00.00.01.91525108025e-054.58310512054e-050.00.05.745753240735611e-056.216616571330262e-050.07.53269879029322e-050.0001454411129350.00.000100440943750786780.000170745190802234830.000165137204252253150.00.00.00.0001127963266970.00.00.000172019408644042550.000166369571448165460.00.00.02.83632602723e-050.0001072480546840.00.08.508978081700064e-050.000154229037074987420.000167515126975996750.00.0002825608911470.00051669346375400010.00037149390154400598.291583006583347e-050.0003932729418306590.00067747458407184420.000462156495950917570.00041044931124008290.04.99604637715e-050.00.00.00.00.00.000149881391314552280.06.13752475745e-050.00.00.00.00018412574272336960.00.00.0001504638957180.00.000200881887501573628.537259540111742e-050.000165137204252253150.00.00.06.64147545521e-050.00.000109351027470614640.08.989323618570229e-050.00.00.06.64147545521e-050.00.000109351027470614640.08.989323618570229e-050.00.00.06.64147545521e-050.00.000109351027470614640.08.989323618570229e-050.00.00.00.00200434566082370030.00264212705672899970.00173593346197546640.0021441170058024540.00213298651470606150.0027526439927997650.0027360112244437820.00243772595295818240.00200434566082370030.00264212705672899970.00173593346197546640.0021441170058024540.00213298651470606150.0027526439927997650.0027360112244437820.00243772595295818240.00175954672993000020.00219578627073999980.00166380831619697580.00183759305954844210.0017772388140562610.00236774283912804040.00224827626439835040.0019713397087081135.99624520447e-050.0001874893858090.06.130478925080239e-050.000118582566883266870.000128300384557241580.00027870569145453230.000155462081416689860.000184836478849000020.000258851400187.212514577849051e-050.000245219157003209560.000237165133766533750.000256600769114483150.000209029268590899230.000310924162833379674.0153420416699995e-050.00065582637534080.00.00.00012046026125023490.0006158763597627960.00060534295484477470.00074625981141421514.0153420416699995e-050.00065582637534080.00.00.00012046026125023490.0006158763597627960.00060534295484477470.00074625981141421511.80368305615e-050.0004293053521050.00.05.41104916845975e-050.000409813849702257150.000381529150437757870.00049657305617564060.06.86922958548e-050.00.00.06.248827019886377e-056.787133418840944e-057.571728317704066e-052.21165898552e-050.000157828727381000020.00.06.634976956563741e-050.00014357423986167510.000155942470218607380.000173969472061533880.00430175286483340.00766298953536440.0054941427907359620.00232856661922922980.0050825491845312030.00757857406913157740.0073001325658774570.0081102619710780330.00430175286483340.00766298953536440.0054941427907359620.00232856661922922980.0050825491845312030.00757857406913157740.0073001325658774570.0081102619710780330.00051330207342300010.001190085163640.0006684274073555880.000243492261531356050.00062798655138180770.00110410612626020570.0009224765843917620.00154367278026431480.00094706819067499990.00165325243897000010.0013666953239681228.116408717711867e-050.00139334516087838550.00150752951854758840.00184765411373782390.00160457368462083850.02.83104134939e-050.00.00.08.493124048155428e-050.00.00.000474837270834999970.0009674102307720.00023788644572554760.00040439650453160880.00078222886224751480.00095748661150536480.00091923982444652750.0010255042563627260.00063949507293300010.001040971666870.00092241683036436830.000313613615759206830.00068245477267512770.00114859391889340090.00098020981280267490.0009941112689230510.00055471019986699990.001048248520560.00067604481370721880.00043233557927488950.00055575020661815970.00105942245368016680.00098896848634364680.00109635462165274350.0003522335941950.0005578997367290.00057700116622792410.000163479438002139720.000316220178355378350.00059873512793379420.000556757144196730.0005182069380556330.03.09672990505e-050.00.00.00.09.290189715151078e-050.00.0003582386802970.0005684634056020.00058446238820500930.000298068113253901340.00019218553943150150.00062380531801969180.000451695430978035170.00062988946780900190.0004360650944440.0004246696080640.000461208415182184160.000392017019699008630.000454969848450085150.00041021211389050040.000267329948946184070.00059646676135383052.58026881644e-050.0001527110516130.00.07.740806449324365e-058.375163991931047e-050.000272899322882562870.000101482192035894777.38801306356e-050.000420245915899000060.00.000221640391906747180.00.00058319663226859670.000251907067276211940.000425634048150548967.38801306356e-050.000420245915899000060.00.000221640391906747180.00.00058319663226859670.000251907067276211940.000425634048150548967.38801306356e-050.0001612782285050.00.000221640391906747180.00.000231927618238090570.000251907067276211940.00.00.000258967687394000040.00.00.00.00035126901403050610.00.000425634048150548960.00.000181619825547999970.00.00.00.00028544937629303450.00.000259410100352109660.00.000181619825547999970.00.00.00.00028544937629303450.00.000259410100352109660.00.000181619825547999970.00.00.00.00028544937629303450.00.000259410100352109660.00093736303513600010.00157660476274000020.00100440943750786780.00056915063600744940.00123852903189189830.0014889180430099640.00161718117263740990.0016237150725743160.00093736303513600010.00157660476274000020.00100440943750786780.00056915063600744940.00123852903189189830.0014889180430099640.00161718117263740990.0016237150725743160.00093736303513600010.00157660476274000020.00100440943750786780.00056915063600744940.00123852903189189830.0014889180430099640.00161718117263740990.0016237150725743167.29006849804e-050.00.000218702054941229250.00.00.00.00.07.29006849804e-050.00.000218702054941229250.00.00.00.00.07.29006849804e-050.00.000218702054941229250.00.00.00.00.04.03557363284e-050.00.000121067208985323320.00.00.00.00.04.03557363284e-050.00.000121067208985323320.00.00.00.00.04.03557363284e-050.00.000121067208985323320.00.00.00.00.01.77946553889e-050.05.338396616675675e-050.00.00.00.00.01.77946553889e-050.05.338396616675675e-050.00.00.00.00.01.77946553889e-050.05.338396616675675e-050.00.00.00.00.05.6579776039e-061.36046161765e-050.01.697393281170965e-050.00.01.9291851985807102e-052.1521996543694915e-055.6579776039e-061.36046161765e-050.01.697393281170965e-050.00.01.9291851985807102e-052.1521996543694915e-055.6579776039e-061.36046161765e-050.01.697393281170965e-050.00.01.9291851985807102e-052.1521996543694915e-057.032776073456e-052.865190087543e-050.000210983282203561420.00.00.04.0712867786868445e-054.5242834839532036e-057.032776073456e-052.865190087543e-050.000210983282203561420.00.00.04.0712867786868445e-054.5242834839532036e-053.4983300842e-052.18400613027e-050.000104949902525976870.00.00.02.0277349068673407e-054.5242834839532036e-052.82932235918e-050.08.487967077531276e-050.00.00.00.00.07.051236300760001e-066.811839572730001e-062.1153708902271787e-050.00.00.02.0435518718195042e-050.00.00.00.00.00.00.00.00.00.000379786048978000050.000280779776840.00042640023290428350.00036243082953304560.00035052708449770710.000227551625441145450.00024715410374269850.00036763360133758110.000379786048978000050.000280779776840.00042640023290428350.00036243082953304560.00035052708449770710.000227551625441145450.00024715410374269850.00036763360133758110.000379786048978000050.000280779776840.00042640023290428350.00036243082953304560.00035052708449770710.000227551625441145450.00024715410374269850.00036763360133758110.05.63138175459e-050.00.00.00.00.00.00016894145263779010.05.63138175459e-050.00.00.00.00.00.00016894145263779010.05.63138175459e-050.00.00.00.00.00.00016894145263779010.00.00.00.00.00.00.00.00.00.00.00.00.00.00.00.00.00.00.00.00.00.00.00.00.00.00.00.00.00.00.00.00.00.00.00.00.00.00.00.00.00.00.00.00.00.00.00.06.42735830253e-050.00011904624165750.0001582102374267023.4610511649101656e-050.00.09.3833578068503e-050.000263305146903943146.42735830253e-050.00011904624165750.0001582102374267023.4610511649101656e-050.00.09.3833578068503e-050.000263305146903943143.18964879767e-058.77683823013e-056.107895228088384e-053.4610511649101656e-050.00.00.00.000263305146903943143.23770950486e-053.12778593562e-059.713128514581816e-050.00.00.09.3833578068503e-050.00.0001185231778445.76230136632e-050.000106210397438821496.018433618355536e-050.000189174799910381384.7233300842222085e-056.8402963437927e-055.723277670953856e-050.0001185231778445.76230136632e-050.000106210397438821496.018433618355536e-050.000189174799910381384.7233300842222085e-056.8402963437927e-055.723277670953856e-050.0001185231778445.76230136632e-050.000106210397438821496.018433618355536e-050.000189174799910381384.7233300842222085e-056.8402963437927e-055.723277670953856e-058.67942548387e-056.62302347078e-059.161842842132578e-059.344838145257447e-057.531595464207491e-054.8892849250192073e-057.080631080196228e-057.899154407118297e-058.67942548387e-056.62302347078e-059.161842842132578e-059.344838145257447e-057.531595464207491e-054.8892849250192073e-057.080631080196228e-057.899154407118297e-058.67942548387e-056.62302347078e-059.161842842132578e-059.344838145257447e-057.531595464207491e-054.8892849250192073e-057.080631080196228e-057.899154407118297e-050.000134277527112000020.0001044813905469.897465260114023e-050.00016825256027957450.000135605368455317350.000146718201318500129.561436130191986e-057.111160901785327e-050.000134277527112000020.0001044813905469.897465260114023e-050.00016825256027957450.000135605368455317350.000146718201318500129.561436130191986e-057.111160901785327e-050.000134277527112000020.0001044813905469.897465260114023e-050.00016825256027957450.000135605368455317350.000146718201318500129.561436130191986e-057.111160901785327e-050.0001003627374736.67643603382e-060.000248798668006536043.5245566908718203e-051.704397750309952e-050.02.0029308101472507e-050.00.0001003627374736.67643603382e-060.000248798668006536043.5245566908718203e-051.704397750309952e-050.02.0029308101472507e-050.00.0001003627374736.67643603382e-060.000248798668006536043.5245566908718203e-051.704397750309952e-050.02.0029308101472507e-050.05.04697049864e-059.9753814296e-060.000151409114959312550.00.02.9926144288785883e-050.00.05.04697049864e-059.9753814296e-060.000151409114959312550.00.02.9926144288785883e-050.00.05.04697049864e-059.9753814296e-060.000151409114959312550.00.02.9926144288785883e-050.00.03.42412308241e-050.00.000102723692472395560.00.00.00.00.03.42412308241e-050.00.000102723692472395560.00.00.00.00.03.42412308241e-050.00.000102723692472395560.00.00.00.00.00.0069328107648075790.0068967000299292610.0048965741385070970.0101653764717243820.0057364816842230440.0050278343801387460.0072108542677028620.0084514114419447180.0069328107648075790.0068967000299292610.0048965741385070970.0101653764717243820.0057364816842230440.0050278343801387460.0072108542677028620.0084514114419447184.24504618017e-054.91227841649e-050.00.000127351385404981770.06.663113894132988e-050.08.073721355341904e-050.00.0001481615651540.00.00.00.000129679958584738820.000236237878526350937.856685835037017e-050.00130630777285999980.000188467562757999970.0009839761342258850.00181172228397320280.00112322490038378425.110269554398605e-050.000305079422159687350.000209220570571050566.32639751094e-052.31289829534e-052.222873345304297e-059.44696752389414e-057.309351663624319e-050.02.147404507928364e-054.791290378088146e-051.22485476316e-058.50820921106e-050.01.8679579220665885e-051.8066063674274044e-059.927062486837379e-058.492166935729672e-057.105398210616945e-050.000170175223827999974.4638427946e-059.13099488643516e-050.000194028625911630470.000225187096707617924.060685571845357e-054.410494107192936e-054.920348704770655e-055.05322023327e-052.03813607436e-055.673442429437747e-054.8223014138706484e-054.663916856496688e-050.00.06.114408223083199e-052.09932611642e-050.0001009566599330.06.29797834926276e-050.00.00.000143160300528557560.000159709679269604880.04.09339934262e-050.00.00.00.00.00.00012280198027872988.59455658401e-060.00.02.578366975201533e-050.00.00.00.06.65817468466e-060.00.01.997452405398761e-050.00.00.00.00.01.43638512663e-050.00.00.01.948341865651165e-050.02.360813514243756e-050.000189804471240999991.65123566702e-050.000183857998730253720.00023441288906747490.000151142525925790980.00.04.953707001074185e-050.00.00051220052849699990.00.00.00.00044388823588270470.00050463423352348040.00058807911608429522.55521077858e-052.26463557007e-052.176489150297948e-053.699935916260305e-051.789207269185728e-050.02.1025951040711108e-054.691311606153723e-055.49796817449e-050.00.00.000111176530988593415.3762514246111356e-050.00.00.00.000197729940549000020.0004073369175048.629776285170963e-050.0003118155528236740.000195076505972522940.000326043922282253640.000291654881745624830.00060431194848387685.48451124197e-050.03.7875774878089976e-056.438715295615e-056.227240942473232e-050.00.00.03.19260979614e-050.0001176018051312.2048012042855634e-053.748065163951497e-053.62496302017141e-055.883042023600346e-050.000127796756081590440.000166178239075792167.48914587764e-050.0005513265640944.335583076128211e-059.217294539645498e-058.914560017152141e-050.000405223934585591770.00052396669993452080.00072478905776291751.83265605816e-054.45540243594e-050.03.705884366286448e-051.7920838082037116e-050.06.3179264461558e-057.048280861656999e-050.02.5259158903e-050.00.00.03.4262034512445194e-050.04.151544219650241e-053.82650829809e-058.03359034624e-050.05.8355951286839746e-055.643929765583335e-053.0532243413622055e-059.948734808883306e-050.000110988118884826680.0002552355868497.856685835039999e-060.000218702054941229250.000297427106558731630.000249577599047863250.00.02.3570057505111045e-050.00.00.00.00.00.00.00.00.00.00.00.00.00.00.00.00.0001016948004570.0001468666855510.000112996061719635130.000192088339652514160.00.000100501967903172560.000218319458306050360.000121778630443073730.001014932479750.0001289930730490.00077259698409827720.00134602238624878870.00092617806891320166.872357516683827e-050.000128328893393799050.000189926750587414120.000197774275075999960.00.0001155641540314450.000351270345362948840.000126488325833023720.00.00.09.762518548600001e-052.89596778238e-052.166058691111216e-050.000161286647504817460.000109928322042209941.926555295268484e-052.0925187697065525e-054.668829282162571e-059.34073880476e-057.83142317962e-064.359976657992352e-050.000111176530988593410.000125445866574259820.00.02.349426953885667e-051.18898787062e-050.00.00.03.5669636118486675e-050.00.00.02.26560188761e-050.00.00.06.796805662821394e-050.00.00.00.00063162571600699990.001040238721850.00071387322109387860.00103624079333024620.000144763133597754370.0010554212653328170.00097622167370417740.00108907322649989640.0005068884720840.000521167246460.000179684963057040470.00082978707164115010.00051119338155460190.000447765789090461350.00041433468776007030.00070140126252901834.69382883717e-050.0002130550325882.329815705559487e-057.921168645464502e-053.830502160490408e-050.000198321510251026820.00025075462417543950.000190088963337869830.00152331726522000020.002276690980480.00114514867741413360.00218995098629094470.00123485213196951760.0014109713984977440.00271210294559269620.0027069985973602716.78757383931e-063.20296083351e-050.02.0362721517934367e-050.02.130783771798712e-052.3143405474139607e-055.163758181331748e-055.44931460007e-050.00.00.000163479438002139720.00.00.00.03.83007707684e-056.2942865236e-050.08.903975711490468e-052.5862555190318067e-054.3072271958502536e-059.356548213116442e-055.2190841618460165e-053.83007707684e-056.2942865236e-050.08.903975711490468e-052.5862555190318067e-054.3072271958502536e-059.356548213116442e-055.2190841618460165e-052.07663069895e-050.00.06.229892096838298e-050.00.00.00.00.00.00.00.00.00.00.00.00.00.00.00.00.00.00.00.00.00.00.00.00.00.00.00.01.75344637789e-050.00.02.6740836146521696e-052.5862555190318067e-050.00.00.00.00.00.00.00.00.00.00.00.06.2942865236e-050.00.00.04.3072271958502536e-059.356548213116442e-055.2190841618460165e-050.00.00.00.00.00.00.00.00.00.00.00.00.00.00.00.00.00.00.00.00.00.00.00.00.00.00.00.00.00.00.00.00.00091205344897729990.00058929807951659990.00064526116234972230.00080075181499899880.00129014736958321530.00086314897110043570.00060570023995413880.0002990450274972010.00091205344897729990.00058929807951659990.00064526116234972230.00080075181499899880.00129014736958321530.00086314897110043570.00060570023995413880.0002990450274972010.000143659689745999980.00.00013978894233356920.000118817529681967530.000172372597222068360.00.00.00.08.93080841426e-050.00.00.00.000124335635062354596.787133418840944e-057.571728317704066e-050.0001266745177180.08.748082197649171e-050.00014871355327936580.000143829177897123680.00.00.01.92517466097e-050.000107735826324000010.00.05.7755239829155896e-050.00018746481059659130.000135742668376818870.00.0001643095678950.00.0001405132373715670.000179149746826179030.000173265719487467650.00.00.01.92517466097e-050.00.00.05.7755239829155896e-050.00.00.07.60838988836e-050.00.05.8504062838329186e-050.000169747633812595220.00.00.00.000144404040470.000134769275380.0001405132373715670.000119433164550786010.000173265719487467650.000124976540397727570.000203614002565228327.571728317704066e-051.99055274251e-050.00.05.9716582275393004e-050.00.00.00.00.0001407574737910.000257484893669999970.000136964923296527420.00011641717554697830.000168890322530713440.000426371985043762370.000198472234823682140.000147610461143119685.77552398292e-050.00.00.00.000173265719487467680.00.00.00.0001143809661730.00.00018832676953272520.00.00015481612898648730.00.00.00.0001143809661730.00.00018832676953272520.00.00015481612898648730.00.00.00.00.00.00.00.00.00.00.00.0001143809661730.00.00018832676953272520.00.00015481612898648730.00.00.00.00.00.00.00.00.00.00.00.00.00.00.00.00.00.00.00.00016413203205730.00.000218923413296432939.33222702765251e-050.00018015041259887710.00.00.00.00016413203205730.00.000218923413296432939.33222702765251e-050.00018015041259887710.00.00.07.94921062601e-050.05.489687209051099e-059.33222702765251e-059.02571764131748e-050.00.00.08.46399257972e-050.00.000164026541205921930.08.989323618570229e-050.00.00.00.0055131882569771010.00486736442346130.0071781823137546420.0052889495381533110.0040724329190294850.00620181550110309850.00389014470388115640.00451013306539700850.0055131882569771010.00486736442346130.0071781823137546420.0052889495381533110.0040724329190294850.00620181550110309850.00389014470388115640.00451013306539700850.0005457004533680.00.00075330707813090080.00051223557240670450.00037155870956756960.00.00.08.66887322575e-050.0003600955071810.000142731867435328560.00.000117334329337127220.00063474927096740570.000137885973666979150.00030765127690881780.00.00.00.00.00.00.00.00.0001284808123140.00.000144250291556981010.000122609578501604780.000118582566883266870.00.00.00.05.73075907967e-050.00.00.00.00.00.000171922772390221744.51972563888e-050.00.00.00013559176916648060.00.00.00.00.000318602613152999970.00.00045198424687854050.000256117786203352250.00024770580637837970.00.00.08.0397381795e-050.00.00.000122609578501604780.000118582566883266870.00.00.03.95275222944e-054.27667948524e-050.00.00.000118582566883266870.000128300384557241580.00.00.00.00.00.00.00.00.00.00.00063675852602199990.000107202099097000010.00054238109625424860.00092202403033206790.00044587045148108340.000321606297290152240.00.00.0002583039618340.0005978666448340.000288500583113962030.00036782873550481440.000118582566883266870.00076980230734344950.00055741138290906470.00046638624425006960.000297831484128000030.0004605123613240.000288500583113962030.00036782873550481450.000237165133766533750.00051320153822896630.00055741138290906470.000310924162833379670.0002156514636830.000730412210370.00028698215899230950.000242637902718965280.000117334329337127220.00101965042463913050.00055301076672820380.00061857543974219740.0008769499803520.00051401864663599990.0012982526240128290.00085826704951123350.00047433026753306750.00064150192278620770.00027870569145453230.00062184832566675930.09.458748865799999e-050.00.00.00.000128300384557241580.00.000155462081416689860.0003854424369420.000273022975513999970.000432750874670942960.00036782873550481450.00035574770064980060.00038490115367172480.00027870569145453230.000155462081416689860.00.0001410384372340.00.00.00.000128300384557241580.000139352845727266160.000155462081416689860.00068266524756900010.00.00111666696287639440.000406775307499441770.00052455347233068640.00.00.00.0002222829610010.00.00042819560230598570.000121318951359482640.000117334329337127220.00.00.08.76109528134e-050.0004284850569310.000144250291556981010.00.000118582566883266870.00038490115367172470.00027870569145453230.00062184832566675930.08.92177434282e-050.00.00.00.000128300384557241580.000139352845727266160.00.000135694383332000020.0002767071292380.000288500583113962030.00.000118582566883266870.000256600769114483150.000418058537181798460.000155462081416689860.00.00.00.00.00.00.00.00.000469402087729999970.0006941237373670.00057092746974131430.000485275805437930560.00035200298801138170.00076169912516088690.00055154389466791660.00076912819227204450.03.26835667978e-050.00.00.09.805070039333912e-050.00.00.03.26835667978e-050.00.00.09.805070039333912e-050.00.00.03.26835667978e-050.00.00.09.805070039333912e-050.00.06.00276061414e-068.99301361994e-050.01.8008281842423204e-050.09.422059490922428e-056.140234764857665e-050.000114167466040381596.00276061414e-068.99301361994e-050.01.8008281842423204e-050.09.422059490922428e-056.140234764857665e-050.000114167466040381596.00276061414e-068.99301361994e-050.01.8008281842423204e-050.09.422059490922428e-056.140234764857665e-050.000114167466040381590.00.0002478058523360.00.00.00.0002254249747360880.000244844252305850830.00027314832996577280.00.0002478058523360.00.00.00.0002254249747360880.000244844252305850830.00027314832996577280.00.0002478058523360.00.00.00.0002254249747360880.000244844252305850830.00027314832996577280.000186411957652000030.0005700671302999.91555934651277e-050.000358190322569587670.000101889956919808840.00046300723787201990.00076631327229911620.000480880880725848740.000186411957652000030.0005700671302999.91555934651277e-050.000358190322569587670.000101889956919808840.00046300723787201990.00076631327229911620.000480880880725848740.000186411957652000030.0005700671302999.91555934651277e-050.000358190322569587670.000101889956919808840.00046300723787201990.00076631327229911620.000480880880725848740.0001396207671390.0001785064472030.000156757542270014040.00013324046681099250.000128864292335573250.000139424695357002420.000227153193613231550.00016894145263779010.0001396207671390.0001785064472030.000156757542270014040.00013324046681099250.000128864292335573250.000139424695357002420.000227153193613231550.00016894145263779010.0001396207671390.0001785064472030.000156757542270014040.00013324046681099250.000128864292335573250.000139424695357002420.000227153193613231550.00016894145263779010.000302136622701330.000325573586377980.000130334074964631250.000388084176290868530.00038799161684848080.00035706405880758540.00013901287355487050.000480643826771535130.000302136622701330.000325573586377980.000130334074964631250.000388084176290868530.00038799161684848080.00035706405880758540.00013901287355487050.000480643826771535131.86493533643e-050.00.05.594806009296529e-050.00.00.00.00.00.00.00.00.00.00.00.00.00.00.00.00.00.00.00.04.4516417068900006e-060.00.01.3354925120684648e-050.00.00.00.04.70863170637e-050.00.07.180872510374361e-056.945022608739618e-050.00.00.08.8723502546e-050.07.617712026042816e-056.474887853455534e-050.000125244508843000960.00.00.00.00.00.00.00.00.00.00.00.00.00.00.00.00.00.00.03.58294712523e-050.000136556530992000022.4743663150285058e-054.2063140069893625e-054.0681610536595204e-050.00015405411138442519.561436130191986e-050.000160001120290169847.53780000514e-050.02.9413291553918043e-050.000100002606326688529.67181022735539e-050.00.00.00.00.00.00.00.00.00.00.00.00.00.00.00.00.00.00.00.00.00.00.00.00.00.00.04.32045011125e-060.00.00.01.2961350333752424e-050.00.00.00.00.00.00.00.00.00.00.00.00.00.00.00.00.00.00.00.00.00.00.00.00.00.00.00.00.00.00.00.00.00.00.00.00.00.00.00.00.00.00.00.00.00.00.00.00.00.00.00.00.00.00.00.00.00.00.00.00.00.00.00.00.00.00.00.00.00.00.00.00.00.00.01.98590952644e-050.00.04.015784104233746e-051.9419444750918268e-050.00.00.00.05.25437926128e-060.00.00.00.01.576313778383035e-050.00.00.00.00.00.00.00.00.00.04.09339934262e-050.00.00.00.00.00.00012280198027872980.00.00.00.00.00.00.00.00.00.00.00.00.00.00.00.07.838791341089999e-060.000106611757327999980.00.02.3516374023263888e-059.435917131162242e-052.763537446912029e-050.00019784072620263550.03.62169253705e-050.00.00.00.000108650776111537910.00.00.00.00.00.00.00.00.00.00.00.00.00.00.00.00.00.00.00.00.00.00.00.00.00.00.00.00.00.00.00.00.00.01.92517466097e-050.00.00.05.7755239829155896e-050.00.00.01.92517466097e-050.00.00.05.7755239829155896e-050.00.00.00.00.00.00.00.00.00.00.01.92517466097e-050.00.00.05.7755239829155896e-050.00.00.00.03.32237910424e-050.00.00.09.967137312711329e-050.00.00.03.32237910424e-050.00.00.09.967137312711329e-050.00.00.03.32237910424e-050.00.00.09.967137312711329e-050.00.00.000428251913480100040.00079137797289930.00058174225339946370.000208558562339899680.00049445492470089220.00052066972013853210.00084057628052776430.00101288791803278960.000428251913480100040.00079137797289930.00058174225339946370.000208558562339899680.00049445492470089220.00052066972013853210.00084057628052776430.00101288791803278960.00.00.00.00.00.00.00.03.73425838761e-050.00.00.00.000112027751628412910.00.00.00.02.5370548008999998e-050.00.00.00.00.07.611164402692107e-050.0001128589455970.00.000126724555199590775.385654382780771e-050.000157995737763209160.00.00.00.02.27623608305e-050.00.00.00.00.06.82870824914432e-050.00.0001718882720370.00.00.00.000115963809119045280.000188930300457158960.000210770706536089170.06.44735713268e-050.00.00.00.00.000124162725102967026.925798887757748e-050.000278050384007000040.0005068832206960.000455017698199872970.000154702018512091980.000224431435309270160.000404705911019486840.00052748325496763840.00058846049610075870.07.51416582454e-050.00.00.00.0002254249747360880.00.00.07.51416582454e-050.00.00.00.0002254249747360880.00.00.07.51416582454e-050.00.00.00.0002254249747360880.00.05.86529281382e-050.000211546588942999980.00.000175958784414516830.00.00.000299980935077015750.00033465883175195835.86529281382e-050.000211546588942999980.00.000175958784414516830.00.00.000299980935077015750.00033465883175195835.86529281382e-050.000211546588942999980.00.000175958784414516830.00.00.000299980935077015750.00033465883175195830.000303092981897000050.0004065339250850.00063809540735793950.00027118353833296120.00.00056754052462968040.00030821570584383580.00034384554478044350.000303092981897000050.0004065339250850.00063809540735793950.00027118353833296120.00.00056754052462968040.00030821570584383580.00034384554478044350.000303092981897000050.0004065339250850.00063809540735793950.00027118353833296120.00.00056754052462968040.00030821570584383580.00034384554478044350.000117734913293999999.60642986182e-050.000109057861177127720.000154494643152156188.965223555249667e-058.083268195965623e-057.02368230475229e-050.000137123390847428840.000117734913293999999.60642986182e-050.000109057861177127720.000154494643152156188.965223555249667e-058.083268195965623e-057.02368230475229e-050.000137123390847428840.000117734913293999999.60642986182e-050.000109057861177127720.000154494643152156188.965223555249667e-058.083268195965623e-057.02368230475229e-050.000137123390847428840.0002916027399224.86299844693e-050.0008748082197649170.00.00.000145889953407831160.00.00.0002916027399224.86299844693e-050.0008748082197649170.00.00.000145889953407831160.00.00.0002916027399224.86299844693e-050.0008748082197649170.00.00.000145889953407831160.00.09.85861998559e-050.0001075330985910.0001064536008349850.000101793624545983968.751137418666994e-050.00010651827805243020.000115694315490939330.000100386702229815459.85861998559e-050.0001075330985910.0001064536008349850.000101793624545983968.751137418666994e-050.00010651827805243020.000115694315490939330.000100386702229815459.85861998559e-050.0001075330985910.0001064536008349850.000101793624545983968.751137418666994e-050.00010651827805243020.000115694315490939330.000100386702229815451.04078069907e-050.00.00.03.1223420972064664e-050.00.00.01.04078069907e-050.00.00.03.1223420972064664e-050.00.00.01.04078069907e-050.00.00.03.1223420972064664e-050.00.00.08.015255657800001e-052.33496746851e-050.06.163262234840027e-050.00017882504738546130.07.004902405541721e-050.08.015255657800001e-052.33496746851e-050.06.163262234840027e-050.00017882504738546130.07.004902405541721e-050.00.00.00.00.00.00.00.00.08.015255657800001e-052.33496746851e-050.06.163262234840027e-050.00017882504738546130.07.004902405541721e-050.00.00057785969126940010.0007378711724920.00061711314943571140.00068298076320893920.00043348516116216450.00055834426612873660.00089753555081376250.0007577337005346810.00057785969126940010.0007378711724920.00061711314943571140.00068298076320893920.00043348516116216450.00055834426612873660.00089753555081376250.0007577337005346812.99327315814e-050.08.979819474408088e-050.00.00.00.00.00.00054792695968800010.0007378711724920.00052731495469163050.00068298076320893920.00043348516116216450.00055834426612873660.00089753555081376250.0007577337005346812.45991277068e-051.52924055126e-052.356357340359257e-052.2253910753332402e-052.7979898963381373e-051.6300763282229183e-051.2646425466656708e-051.6930027788957925e-052.45991277068e-051.52924055126e-052.356357340359257e-052.2253910753332402e-052.7979898963381373e-051.6300763282229183e-051.2646425466656708e-051.6930027788957925e-052.45991277068e-051.52924055126e-052.356357340359257e-052.2253910753332402e-052.7979898963381373e-051.6300763282229183e-051.2646425466656708e-051.6930027788957925e-050.0001438060524180.000330705133510.000133591403018287840.000151399676573410180.000146427077662096370.000257443464579555430.00049471404345213380.000239957892498667430.0001438060524180.000330705133510.000133591403018287840.000151399676573410180.000146427077662096370.000257443464579555430.00049471404345213380.000239957892498667430.0001438060524180.000330705133510.000133591403018287840.000151399676573410180.000146427077662096370.000257443464579555430.00049471404345213380.000239957892498667433.15521289269e-050.09.465638678084616e-050.00.00.00.00.03.15521289269e-050.09.465638678084616e-050.00.00.00.00.03.15521289269e-050.09.465638678084616e-050.00.00.00.00.00.0001864983542957.66364957914e-050.00055949506288363020.00.00.00.000158970479349065827.093900802509149e-050.0001864983542957.66364957914e-050.00055949506288363020.00.00.00.000158970479349065827.093900802509149e-050.0001864983542957.66364957914e-050.00055949506288363020.00.00.00.000158970479349065827.093900802509149e-050.000227701317205000038.809906570899999e-050.00068310395161710280.00.00.00.00026429719712701390.00.000227701317205000038.809906570899999e-050.00068310395161710280.00.00.00.00026429719712701390.00.000113563881125000012.19416541011e-050.000340691643375784350.00.00.06.582496230333176e-050.00.000114137436080000016.61574116079e-050.00034241230824131850.00.00.00.000198472234823682140.00.0001291383562510.00.00038741506875303470.00.00.00.00.00.0001291383562510.00.00038741506875303470.00.00.00.00.00.0001291383562510.00.00038741506875303470.00.00.00.00.00.08.97203253313e-050.00.00.00.00.00026916097599376070.00.08.97203253313e-050.00.00.00.00.00026916097599376070.00.08.97203253313e-050.00.00.00.00.00026916097599376070.00.0001175947351320.0006243306032020.00.000195344074222895750.000157440131172698960.0005791638828318420.00059205276828759430.00070177515848550960.0001175947351320.0006243306032020.00.000195344074222895750.000157440131172698960.0005791638828318420.00059205276828759430.00070177515848550960.0001175947351320.0006243306032020.00.000195344074222895750.000157440131172698960.0005791638828318420.00059205276828759430.00070177515848550967.227922265410001e-056.64475820847e-050.000112062209969886080.000104775457992280440.00.000199342746254226580.00.07.227922265410001e-056.64475820847e-050.000112062209969886080.000104775457992280440.00.000199342746254226580.00.03.7354069990000004e-056.64475820847e-050.000112062209969886080.00.00.000199342746254226580.00.03.49251526641e-050.00.00.000104775457992280440.00.00.00.00.00.000139553822104999990.00.00.00.000126949854193481150.000137885973666979150.00015382563845440890.00.000139553822104999990.00.00.00.000126949854193481150.000137885973666979150.00015382563845440890.00.000139553822104999990.00.00.00.000126949854193481150.000137885973666979150.00015382563845440897.29739961179e-052.42905058376e-058.193067919248468e-056.9639277215413e-056.735203194578299e-057.287151751287438e-050.00.07.29739961179e-052.42905058376e-058.193067919248468e-056.9639277215413e-056.735203194578299e-057.287151751287438e-050.00.07.29739961179e-052.42905058376e-058.193067919248468e-056.9639277215413e-056.735203194578299e-057.287151751287438e-050.00.00.000118596449617999990.000236426225139000030.000355789348854827860.00.00.00063289869000248637.637998541319544e-050.00.000118596449617999990.000236426225139000030.000355789348854827860.00.00.00063289869000248637.637998541319544e-050.00.000118596449617999990.000236426225139000030.000355789348854827860.00.00.00063289869000248637.637998541319544e-050.02.68505493195e-050.08.055164795855177e-050.00.00.00.00.02.68505493195e-050.08.055164795855177e-050.00.00.00.00.02.68505493195e-050.08.055164795855177e-050.00.00.00.00.00.0003819746678830.000533457784140.000412457107417679540.00039440191411542840.00033906498211489240.000458564112105730360.00069729408736533190.000444515152948101870.0003819746678830.000533457784140.000412457107417679540.00039440191411542840.00033906498211489240.000458564112105730360.00069729408736533190.000444515152948101870.0003819746678830.000533457784140.000412457107417679540.00039440191411542840.00033906498211489240.000458564112105730360.00069729408736533190.000444515152948101870.00046300825290.000215711540865000030.00138902475869990480.00.00.0006471346225960380.00.00.00046300825290.000215711540865000030.00138902475869990480.00.00.0006471346225960380.00.00.00046300825290.000215711540865000030.00138902475869990480.00.00.0006471346225960380.00.06.20979338204e-050.00.000186293801461223720.00.00.00.00.06.20979338204e-050.00.000186293801461223720.00.00.00.00.06.20979338204e-050.00.000186293801461223720.00.00.00.00.05.31746172798e-051.5765014573e-050.00015952385183948490.00.04.729504371914004e-050.00.05.31746172798e-051.5765014573e-050.00015952385183948490.00.04.729504371914004e-050.00.05.31746172798e-051.5765014573e-050.00015952385183948490.00.04.729504371914004e-050.00.01.512201631192e-055.85826243863e-063.196044305838735e-050.01.3405605877362705e-050.00.01.7574787315897588e-051.512201631192e-055.85826243863e-063.196044305838735e-050.01.3405605877362705e-050.00.01.7574787315897588e-059.90430494824e-065.85826243863e-061.6307308967355642e-050.01.3405605877362705e-050.00.01.7574787315897588e-055.2177113636800004e-060.01.5653134091031706e-050.00.00.00.00.04.68377457905e-050.00.0001405132373715670.00.00.00.00.04.68377457905e-050.00.0001405132373715670.00.00.00.00.04.68377457905e-050.00.0001405132373715670.00.00.00.00.00.000261432181887999970.000304931255915000060.000310523528389837030.000175958784414516830.00029781423285950230.000230157178404212020.000349977757589851650.00033465883175195830.000261432181887999970.000304931255915000060.000310523528389837030.000175958784414516830.00029781423285950230.000230157178404212020.000349977757589851650.00033465883175195830.000261432181887999970.000304931255915000060.000310523528389837030.000175958784414516830.00029781423285950230.000230157178404212020.000349977757589851650.00033465883175195830.0001281236929540.00.00.00.0003843710788630030.00.00.00.0001281236929540.00.00.00.0003843710788630030.00.00.00.0001281236929540.00.00.00.0003843710788630030.00.00.00.00104170558926408980.00256243514920030.000384202686737748150.0011790574365805640.00156185664447423740.0025966084181320910.00193954670965773830.003151150319811660.00104170558926408980.00256243514920030.000384202686737748150.0011790574365805640.00156185664447423740.0025966084181320910.00193954670965773830.003151150319811668.96483260822e-061.57267059312e-050.01.3671767946798165e-051.3222729877849452e-051.4306329950629547e-051.5538751480857674e-051.733503636200338e-050.000126703698372999980.0006632307224025.4675513735307313e-050.00.000325435581383917630.00078412606485038240.000404215654123208760.00080135044823094448.704104558799999e-050.0002166612209745.739482500044959e-059.756868045841993e-050.000106159631305019880.000191432319815566830.000180200187808168530.000278351155298454250.000399054433943999960.0006100055434390.000116739287644821440.00050859492477549740.00057182908941169870.00057720175183308670.00047577061964717360.0007770442588380640.00.00.00.00.00.00.00.02.34319833999e-051.80635792993e-050.04.273906197460143e-052.755688822503606e-050.00.05.419073789803034e-050.05.48840983571e-050.00.00.07.444590215049821e-050.09.020639292079534e-058.82821730213e-050.0001204820180794.86004566536065e-059.638841416255192e-050.000119857648247603070.000129679958584738820.000109550982662534210.000122215112989464646.4896130701e-050.000134893625637999982.082876713725993e-053.540798887603948e-050.00013845163608958610.00020537484512471474.024321812093094e-050.000159062813669164560.000225153571867000030.0006816843630445.603110498494304e-050.000360086170683025460.00025934343993352640.00054201535992865930.00071402729581486440.00078901043338843670.00.00.00.00.00.00.00.08.20014256788e-063.36657598925e-050.02.4600427703630415e-050.03.8613349461197574e-050.06.238393021630244e-050.01.31375121442e-050.00.00.03.9412536432616705e-050.00.09.97757719379e-060.02.9932731581360298e-050.00.00.00.00.00.00149545243697259980.00157535156716670.00197567196502572730.00128497387529516190.00122571147059385080.00156314436968219110.00154264920671007820.0016202611251074180.00149545243697259980.00157535156716670.00197567196502572730.00128497387529516190.00122571147059385080.00156314436968219110.00154264920671007820.0016202611251074180.000212392277242999980.00.00026674480143651570.000151151480382306260.000219280549908729580.00.00.00.000238100616190.00.00038964159213667280.00013247471700173390.00019218553943150150.00.00.02.16022505563e-050.00.00.06.480675166876213e-050.00.00.02.14773820559e-050.00.00.06.443214616778662e-050.00.00.00.00.000473857977165999970.00.00.00.00067566844942033970.00054008244470799730.000205823037368575330.000221245293829000040.0001103478187170.00031533784665944680.00020102268103170090.000147375353794888720.07.615795057187803e-050.00025488550557852641.62001522179e-051.56501403804e-054.86004566536065e-050.00.00.04.695042114108609e-050.00.02.50941906099e-050.00.00.00.07.528257182967252e-050.05.25110098906e-050.00.09.57781194943284e-056.175491017743538e-050.00.00.00.05.37065952549e-050.00.00.00.07.615795057187803e-058.496183519284212e-050.000118143385082999990.00.000156757542270014040.00013324046681099256.443214616778662e-050.00.00.01.86001747687e-050.05.5800524305992666e-050.00.00.00.00.09.53852806109e-050.00.00015585663685466916.623735850086696e-056.406184647716716e-050.00.00.00.00.0001570929200670.00.00.00.000140235304050938487.615795057187803e-050.00025488550557852647.06268792837e-057.75299011694e-057.929548190851587e-056.73994174219348e-056.518573852062624e-057.052769677415619e-057.660331870387729e-058.545868803022717e-050.00.000187333158994000020.00.00.00.00022196753647326460.000160725981575006380.000179305958934587080.0003543817659250.0004503747513770.00034327917484446110.000437669634651298160.000282196488279166740.000381653042670275650.00041453061703680450.00055494059442413355.47859693186e-052.43641134311e-050.00016435790795583290.00.07.30923402932164e-050.00.00.00015842641084356.93096519194e-059.255650106727792e-050.000196677480872881450.00018604525059047749.967137312711329e-050.000108257582631099360.00.00015842641084356.93096519194e-059.255650106727792e-050.000196677480872881450.00018604525059047749.967137312711329e-050.000108257582631099360.02.39715296495e-050.00.00.07.191458894856185e-050.00.00.00.06.93096519194e-050.00.00.09.967137312711329e-050.000108257582631099360.00.0001344548811940.09.255650106727792e-050.000196677480872881450.000114130661641915550.00.00.00.00010950787277360.0008464558323510.00013978894233356920.00.000188734675987077050.00079750357389530080.00078604673044642540.00095581719271187270.00010950787277360.0008464558323510.00013978894233356920.00.000188734675987077050.00079750357389530080.00078604673044642540.00095581719271187270.00.00.00.00.00.00.00.00.00.000429107537109000040.00.00.00.000236475218595700150.00034246189538203970.00070838549734859872.46065370575e-050.0001167152467950.00.07.381961117236481e-057.986911356543515e-050.000173498907263086379.67777195574096e-058.49013357161e-050.0003006330484470.00013978894233356920.00.000114915064814712230.00048115924173416560.000270085927801299370.00015065397580586440.00.00.00.00.00.00.00.00.00057853641660513.28153974559e-050.000292816867198826360.0008604897279576630.0005823026546607950.04.653345470111197e-055.191273766667442e-050.00057853641660513.28153974559e-050.000292816867198826360.0008604897279576630.0005823026546607950.04.653345470111197e-055.191273766667442e-050.0001421909925030.02.801555249247152e-050.000214313436802391860.000184243988215323760.00.00.06.13639561003e-050.02.14210543544332e-050.000127451978916360133.521883503010138e-050.00.00.08.41238248852e-050.06.865583496889223e-050.00012727634203077985.643929765583335e-050.00.00.08.339721861199999e-053.28153974559e-054.816883625703806e-050.000122827357504271957.919546207479279e-050.04.653345470111197e-055.191273766667442e-055.88834350996e-050.00.05.348167229304339e-050.000123168633005824160.00.00.00.0001485769894050.00.000126555589125991350.000215138940410815920.000104036438678919490.00.00.03.38807333952e-050.0001570657442150.00.00.000101642200185657330.000183286263653202280.000199075493896094548.883547509525136e-053.38807333952e-050.0001570657442150.00.00.000101642200185657330.000183286263653202280.000199075493896094548.883547509525136e-053.38807333952e-050.0001570657442150.00.00.000101642200185657330.000183286263653202280.000199075493896094548.883547509525136e-050.00.00.00.00.00.00.00.00.00.00.00.00.00.00.00.00.00.00.00.00.00.00.00.02.75052936245e-055.51741308043e-054.4603708573540174e-053.7912172299838316e-050.07.934365887092572e-058.617873354186196e-050.02.75052936245e-055.51741308043e-054.4603708573540174e-053.7912172299838316e-050.07.934365887092572e-058.617873354186196e-050.02.75052936245e-055.51741308043e-054.4603708573540174e-053.7912172299838316e-050.07.934365887092572e-058.617873354186196e-050.05.29255558406e-060.01.5877666752173557e-050.00.00.00.00.05.29255558406e-060.01.5877666752173557e-050.00.00.00.00.00.00.00.00.00.00.00.00.05.29255558406e-060.01.5877666752173557e-050.00.00.00.00.03.7509066131e-050.00.000112527198392997630.00.00.00.00.03.7509066131e-050.00.000112527198392997630.00.00.00.00.03.7509066131e-050.00.000112527198392997630.00.00.00.00.00.00.00.00.00.00.00.00.00.00.00.00.00.00.00.00.00.00.00.00.00.00.00.00.00.00.00.00.00.00.00.00.00.00.00.00.00.00.00.00.00.00.00.00.00.00.00.00.09.72009133072e-050.00.000291602739921639060.00.00.00.00.09.72009133072e-050.00.000291602739921639060.00.00.00.00.09.72009133072e-050.00.000291602739921639060.00.00.00.00.00.000411263910240999960.00.00099912307204730.00.000234668658674254430.00.00.00.000411263910240999960.00.00099912307204730.00.000234668658674254430.00.00.00.000411263910240999960.00.00099912307204730.00.000234668658674254430.00.00.00.008404510069583270.010283126105221710.012204310823142120.0069338611142765040.00607535827132134850.009958488985059240.0100676650174185540.010823224313191443.42812063063e-050.00.04.925342042372159e-055.359019849532254e-050.00.00.03.42812063063e-050.00.04.925342042372159e-055.359019849532254e-050.00.00.01.78633994984e-050.00.00.05.359019849532254e-050.00.00.01.64178068079e-050.00.04.925342042372159e-050.00.00.00.09.20245177831e-050.00.000173901882795813053.48196386077065e-056.735203194578299e-050.00.00.09.20245177831e-050.00.000173901882795813053.48196386077065e-056.735203194578299e-050.00.00.04.77123367166e-050.04.096533959624234e-053.48196386077065e-056.735203194578299e-050.00.00.04.43121810665e-050.00.000132936543199570720.00.00.00.00.00.0003397351560540.00.00058976154527444310.000383382925835389844.606099705383094e-050.00.00.00.0003397351560540.00.00058976154527444310.000383382925835389844.606099705383094e-050.00.00.00.000236600246599000010.00.0004216682303196140.000288132509478771260.00.00.00.00.0001031349094550.00.00016809331495482919.525041635661859e-054.606099705383094e-050.00.00.05.18334607619e-050.000169418342128899985.8195396593803494e-054.9464808494209664e-054.7840177197541135e-050.000233523454644338367.119112770849468e-050.00020354044403401435.18334607619e-050.000169418342128899985.8195396593803494e-054.9464808494209664e-054.7840177197541135e-050.000233523454644338367.119112770849468e-050.00020354044403401430.00.00.00.00.00.00.00.00.09.39004990005e-050.00.00.00.000131089523351964237.119112770849468e-057.942084594113503e-050.00.00.00.00.00.00.00.00.03.73580837557e-050.00.00.05.067326112765004e-050.06.14009901393649e-055.18334607619e-053.81597593727e-055.8195396593803494e-054.9464808494209664e-054.7840177197541135e-055.176067016472408e-050.06.271860795351436e-050.00092780650466230010.001784267064924670.00054696373008170990.0013931963336654610.00084325945023949340.00137957398967140670.00142969851624048360.0025435286888573140.00092780650466230010.001784267064924670.00054696373008170990.0013931963336654610.00084325945023949340.00137957398967140670.00142969851624048360.0025435286888573140.06.78432481577e-060.00.00.00.00.02.0352974447310373e-054.47906025477e-058.31026121209e-053.830374973546953e-054.883601855572394e-054.723203935180968e-055.110269554398605e-057.400659603594927e-050.000124198544782678940.000170480847264000010.000259181835351999962.3459389976394835e-050.000267672370397384150.000220310781417659570.000193685904744943680.000165045174964330220.000418814426347023543.2696820389000006e-050.000124356502834999981.8885135663448767e-054.8155851166368455e-053.10494743371228e-050.000100781917674490590.000109463795250944460.0001628237955784830.0006526919424930.001122857296460.00046631545470639670.00096840633673596780.00052335403603724740.00092069607669345390.00093306845839094530.00151480735429189295.3506501296e-063.20332918321e-050.01.6051950388789483e-050.01.6796986279081768e-051.824396587515741e-056.105892334193111e-052.1795641839e-058.69886252758e-050.04.407380642122696e-052.1313119095654085e-054.611944989820538e-057.513862809768463e-050.000139707797831442140.06.89625762331e-050.00.00.05.0390958837245296e-055.473189762547223e-050.000101764872236551853.94746067143e-059.72758916059e-050.000118423820142849020.00.00.00.000109159729153025150.00018266794566461063.94746067143e-059.72758916059e-050.000118423820142849020.00.00.00.000109159729153025150.00018266794566461060.09.72758916059e-050.00.00.00.00.000109159729153025150.00018266794566461060.00.00.00.00.00.00.00.03.94746067143e-050.00.000118423820142849020.00.00.00.00.00.0005318521523910.00049709989088460.000351283093428917470.000427005668958455360.0008172676947867650.00049512237057090140.00040203211351794680.00059414518856625220.0005318521523910.00049709989088460.000351283093428917470.000427005668958455360.0008172676947867650.00049512237057090140.00040203211351794680.00059414518856625220.08.67404704196e-050.00.00.05.7704479178855065e-056.267544257589961e-050.00013984148950400818.534123901e-050.00.0001405132373715670.00.000115510479658311790.00.00.00.00.0001582140151090.00.00.00.00018746481059659130.000135742668376818870.000151434566354081320.0004465109133810.000252145405355999970.00021076985605735050.000427005668958455360.00070175721512845330.00024995308079545510.000203614002565228320.000302869132708162650.00.00.00.00.00.00.00.00.00.00.00.00.00.00.00.00.00.00.00.00.00.00.00.00.05.74296959447e-050.00.00.00.000172289087834010140.00.00.05.74296959447e-050.00.00.00.000172289087834010140.00.00.05.74296959447e-050.00.00.00.000172289087834010140.00.02.05992857536e-050.00017528950620120.06.179785726086248e-050.06.466614556772498e-050.000265101179673272360.000196101193362452042.05992857536e-050.00017528950620120.06.179785726086248e-050.06.466614556772498e-050.000265101179673272360.000196101193362452042.05992857536e-059.58519050504e-050.06.179785726086248e-050.06.466614556772498e-050.000105355234571284360.000117534335012081880.05.32486483673e-050.00.00.00.00.000159745945101988030.00.02.61889527835e-050.00.00.00.00.07.856685835037017e-050.00.00.00.00.00.00.00.00.00.00.00.00.00.00.00.00.00.00.00.00.00.00.00.00.09.767047077959999e-050.00.00.04.291898985188864e-056.848464759509894e-050.000181607774891860470.09.767047077959999e-050.00.00.04.291898985188864e-056.848464759509894e-050.000181607774891860470.01.03421342202e-050.00.00.00.00.03.1026402660655724e-050.07.19067432642e-050.00.00.04.291898985188864e-054.661625444257303e-050.000126184985498034740.01.54215932952e-050.00.00.00.02.186839315252591e-052.439638673317003e-050.00.00.00.00.00.00.00.00.0005834639618010.00086362167321050.0006808704101845960.00052904687038373650.00054047460483261780.00087870702879334370.00065564562239067160.0010565123684474560.0005834639618010.00086362167321050.0006808704101845960.00052904687038373650.00054047460483261780.00087870702879334370.00065564562239067160.0010565123684474565.8627166783999995e-058.38722903694e-056.582294857454473e-055.594806009296529e-055.41104916845975e-050.000117089671343502026.358819173962631e-057.093900802509149e-051.94533355795e-054.65509106484e-050.00.05.83600067383617e-056.314259763550109e-050.07.651013430978455e-050.03.65893989047e-050.00.00.04.963060143366548e-050.06.0137595280530236e-050.0001756527221610.000209758893249000040.000233784955282003660.000149034056626950640.000144139154573626090.000207935106006563950.00016938578661676320.000251955787123600836.55302217975e-050.000110021685477000015.6263599196498815e-054.7822823150003526e-059.250424304586792e-050.000100084947289466460.000108706784218780230.000121273324922563030.000128640142490.0002039632784610.000208608113943941760.000177312313525397730.00.00018554209459047250.000201525653820969570.000224822086971828430.0001355603729890.000172865216100999960.0001163907931876079.892961698841933e-050.000191360708790164540.00015528201049417220.000112439205994532340.000250874431814057444.6156361436999995e-050.000164690845962000020.00.00.000138469084310895480.000149816598116530520.00016272257761941640.000181533362151165824.6156361436999995e-050.000164690845962000020.00.00.000138469084310895480.000149816598116530520.00016272257761941640.000181533362151165824.6156361436999995e-050.000164690845962000020.00.00.000138469084310895480.000149816598116530520.00016272257761941640.000181533362151165820.0002857503146940.0001565587812620.00076442973297578669.282121110725517e-050.00.000129505891526235780.00028132440264940725.8846049610075907e-050.0002857503146940.0001565587812620.00076442973297578669.282121110725517e-050.00.000129505891526235780.00028132440264940725.8846049610075907e-050.0002857503146940.0001565587812620.00076442973297578669.282121110725517e-050.00.000129505891526235780.00028132440264940725.8846049610075907e-050.0003943486717735.8609250552000006e-050.00118304601531966950.00.00.00.00017582775165587950.00.0003943486717735.8609250552000006e-050.00118304601531966950.00.00.00.00017582775165587950.00.0003943486717735.8609250552000006e-050.00118304601531966950.00.00.00.00017582775165587950.00.000258367881910.000332060380279999940.000291602739921639060.000123927961066138180.000359572944742809160.000259359917169477650.00042255379026977490.00031426743340148070.000258367881910.000332060380279999940.000291602739921639060.000123927961066138180.000359572944742809160.000259359917169477650.00042255379026977490.00031426743340148070.000258367881910.000332060380279999940.000291602739921639060.000123927961066138180.000359572944742809160.000259359917169477650.00042255379026977490.00031426743340148070.000196318386890.0001190370453560.00020467211179405610.000173966798175861920.000210316250698624240.000136530975264687230.00.000220580160802548640.000196318386890.0001190370453560.00020467211179405610.000173966798175861920.000210316250698624240.000136530975264687230.00.000220580160802548640.000196318386890.0001190370453560.00020467211179405610.000173966798175861920.000210316250698624240.000136530975264687230.00.000220580160802548640.09.79798252174e-050.00.00.03.536726143220149e-050.000172862914201271548.570930001858559e-050.09.79798252174e-050.00.00.03.536726143220149e-050.000172862914201271548.570930001858559e-050.09.79798252174e-050.00.00.03.536726143220149e-050.000172862914201271548.570930001858559e-050.00.000199596747350.00.00.00.00.00031640501203775410.000282385230012924630.00.000199596747350.00.00.00.00.00031640501203775410.000282385230012924630.00.000199596747350.00.00.00.00.00031640501203775410.000282385230012924630.00.0002007318111020.00.00.00.000281405510128883230.000174655566644840280.000146134356531688470.00.0002007318111020.00.00.00.000281405510128883230.000174655566644840280.000146134356531688470.00.0002007318111020.00.00.00.000281405510128883230.000174655566644840280.000146134356531688471.12015922399e-050.03.360477671959409e-050.00.00.00.00.01.12015922399e-050.03.360477671959409e-050.00.00.00.00.01.12015922399e-050.03.360477671959409e-050.00.00.00.00.08.27205229967e-050.000105758956732999999.287347538600148e-057.894041355582774e-057.634768004813073e-058.260435718068978e-050.000134580487996880370.000100092025021704428.27205229967e-050.000105758956732999999.287347538600148e-057.894041355582774e-057.634768004813073e-058.260435718068978e-050.000134580487996880370.000100092025021704428.27205229967e-050.000105758956732999999.287347538600148e-057.894041355582774e-057.634768004813073e-058.260435718068978e-050.000134580487996880370.000100092025021704420.00.00.00.00.00.00.00.00.00.00.00.00.00.00.00.00.00.00.00.00.00.00.00.00.09.648188918700002e-050.00.00.00.0002894456675611370.00.00.09.648188918700002e-050.00.00.00.0002894456675611370.00.00.09.648188918700002e-050.00.00.00.0002894456675611370.00.00.00.00.00.00.00.00.00.00.00.00.00.00.00.00.00.00.00.00.00.00.00.00.00.00.00.00.00.00.00.00.00.00.00.00.00.00.00.00.00.00.00.00.00.00.00.00.00.00.00.00.00.00.00.00.00.00.00.00.00.00.00.00.00.00.00.00.00.00.00.00.00.00.00.00.00.00.00.00.00.00.00.00.00.00.00.00.00.00.00.00.00.00.00.00.00.00.00.00.00.00.00.00.00.00.00.00.00.00.00.00.00.00.00.00.00.00.00.00.00.00.00.00.00.00.00.00.00.00.00.00.00.00.00.00.00.08.31001519476e-050.000187333158994000020.00.00010176915269882920.000147531303143959160.00022196753647326460.000160725981575006380.000179305958934587088.31001519476e-050.000187333158994000020.00.00010176915269882920.000147531303143959160.00022196753647326460.000160725981575006380.000179305958934587084.96605329709e-050.00.05.077224836630331e-059.820935054649417e-050.00.00.00.00.00.00.00.00.00.00.03.34396189767e-050.00.05.0996904332525894e-054.932195259746498e-050.00.00.00.00.000187333158994000020.00.00.00.00022196753647326460.000160725981575006380.000179305958934587080.08.31693174499e-050.00.00.00.00.000249507952349771850.00.08.31693174499e-050.00.00.00.00.000249507952349771850.00.08.31693174499e-050.00.00.00.00.000249507952349771850.00.000219204500339000020.0003438849330490.00021297687025690390.00021119660380642920.00023344002695344680.000426212534039632370.000222891593558533040.00038255067154892270.000219204500339000020.0003438849330490.00021297687025690390.00021119660380642920.00023344002695344680.000426212534039632370.000222891593558533040.00038255067154892270.000219204500339000020.0003438849330490.00021297687025690390.00021119660380642920.00023344002695344680.000426212534039632370.000222891593558533040.00038255067154892270.0002453859521380.0002502610348420.000231178827911646970.000241842368611689980.000263136659890475460.000205617140890425260.000257688540951403740.00028747742268528880.0002453859521380.0002502610348420.000231178827911646970.000241842368611689980.000263136659890475460.000205617140890425260.000257688540951403740.00028747742268528880.0002453859521380.0002502610348420.000231178827911646970.000241842368611689980.000263136659890475460.000205617140890425260.000257688540951403740.00028747742268528884.83405611635e-050.00.000145021683490440820.00.00.00.00.04.83405611635e-050.00.000145021683490440820.00.00.00.00.04.83405611635e-050.00.000145021683490440820.00.00.00.00.05.52713393588e-056.97752163779e-059.10035396399746e-050.07.48104784364234e-050.000121411773305846048.791387582793975e-050.05.52713393588e-056.97752163779e-059.10035396399746e-050.07.48104784364234e-050.000121411773305846048.791387582793975e-050.05.52713393588e-056.97752163779e-059.10035396399746e-050.07.48104784364234e-050.000121411773305846048.791387582793975e-050.00.00044022207841930.000351384845699000030.000466865787137976550.000446267251692621150.00040753319642728530.00029338991809045810.00039427645521971050.0003664881637870630.00044022207841930.000351384845699000030.000466865787137976550.000446267251692621150.00040753319642728530.00029338991809045810.00039427645521971050.0003664881637870635.94151326144e-052.68532976275e-057.88344616648617e-056.70075603439003e-053.2403375834381074e-050.03.8078975285939015e-054.248091759642107e-056.69058376559e-051.25111437425e-053.885251405832726e-056.604756664269828e-059.581743226670848e-050.03.75334312274012e-050.00.0001641783733640.0001609364943580.000175338716461502760.000149034056626950640.000168162347002563830.000155951329504922950.00016938578661676320.000157472366952250490.0001497227347850.000151083909971000020.000173840094953284820.000164178068079071960.000111150041323631910.000137438588585535160.000149278262089607070.000166534879238391430.01.62621570433e-050.00.00.00.04.878647112984365e-050.00.01.62621570433e-050.00.00.00.04.878647112984365e-050.00.01.62621570433e-050.00.00.00.04.878647112984365e-050.08.44830367997e-067.514165824539999e-062.534491103991816e-050.00.02.2542497473608804e-050.00.08.44830367997e-067.514165824539999e-062.534491103991816e-050.00.02.2542497473608804e-050.00.08.44830367997e-067.514165824539999e-062.534491103991816e-050.00.02.2542497473608804e-050.00.00.0002156160399010.000163394790297000020.000286974125002247870.00018294127585953730.000176932718841699770.00012762154654371120.000207923293624809840.000154639530721363460.0002156160399010.000163394790297000020.000286974125002247870.00018294127585953730.000176932718841699770.00012762154654371120.000207923293624809840.000154639530721363460.0002156160399010.000163394790297000020.000286974125002247870.00018294127585953730.000176932718841699770.00012762154654371120.000207923293624809840.000154639530721363463.38481655775e-050.00.00.000101544496732606640.00.00.00.03.38481655775e-050.00.00.000101544496732606640.00.00.00.03.38481655775e-050.00.00.000101544496732606640.00.00.00.00.03.6487979159e-050.00.00.00.00.00.000109463937476920210.03.6487979159e-050.00.00.00.00.00.000109463937476920210.03.6487979159e-050.00.00.00.00.00.000109463937476920210.0006693416222640.00075239163483100010.00071365933717664280.00070769388293031540.00058667164668563610.00052895772580617140.00057452489027907980.00115369228840806670.0006693416222640.00075239163483100010.00071365933717664280.00070769388293031540.00058667164668563610.00052895772580617140.00057452489027907980.00115369228840806670.0006693416222640.00075239163483100010.00071365933717664280.00070769388293031540.00058667164668563610.00052895772580617140.00057452489027907980.00115369228840806670.0003637709746240.0003406371991830.000408419500191452260.00034714760178165210.00033574582189840620.00036326012495122620.00039455323790250060.00026409823469582250.0003637709746240.0003406371991830.000408419500191452260.00034714760178165210.00033574582189840620.00036326012495122620.00039455323790250060.00026409823469582250.0003637709746240.0003406371991830.000408419500191452260.00034714760178165210.00033574582189840620.00036326012495122620.00039455323790250060.00026409823469582255.60311049849e-051.10745970141e-050.00016809331495482910.00.03.32237910423711e-050.00.05.60311049849e-051.10745970141e-050.00016809331495482910.00.03.32237910423711e-050.00.05.60311049849e-051.10745970141e-050.00016809331495482910.00.03.32237910423711e-050.00.03.14670781183e-052.18087452954e-057.355982318095594e-052.0841411173871334e-050.06.542623588633296e-050.00.03.14670781183e-052.18087452954e-057.355982318095594e-052.0841411173871334e-050.06.542623588633296e-050.00.03.14670781183e-052.18087452954e-057.355982318095594e-052.0841411173871334e-050.06.542623588633296e-050.00.00.001496803363060.0007009571752750.0036220655400540570.00086834454911410530.00.00082604357180689780.00107664390397504280.000200184050043408850.001496803363060.0007009571752750.0036220655400540570.00086834454911410530.00.00082604357180689780.00107664390397504280.000200184050043408850.001496803363060.0007009571752750.0036220655400540570.00086834454911410530.00.00082604357180689780.00107664390397504280.000200184050043408854.5133443479e-050.02.1022523110629788e-054.935857450468721e-056.501923282177446e-050.00.00.04.5133443479e-050.02.1022523110629788e-054.935857450468721e-056.501923282177446e-050.00.00.02.06485078927e-050.00.03.14898917463138e-053.0455631931767994e-050.00.00.00.00.00.00.00.00.00.00.02.44849355863e-050.02.1022523110629788e-051.786868275837341e-053.456360089000647e-050.00.00.00.00012626496443540.00105474570460339.549434888829497e-050.0001632059531077720.000120094591310265160.00135379063931637230.00095799606139319810.00085245041310151610.00012626496443540.00105474570460339.549434888829497e-050.0001632059531077720.000120094591310265160.00135379063931637230.00095799606139319810.00085245041310151610.00.000222245814359000030.00.00.00.000319602607652770650.000347134835425204630.01.3864130954000002e-050.00.00.04.159239286204137e-050.00.00.00.02.97783608602e-050.00.00.08.933508258059785e-050.00.04.50641655131e-050.05.0595251516254526e-054.300485216101064e-054.159239286204137e-050.00.00.01.44972331813e-050.00.04.349169954396547e-050.00.00.00.00.02.90125525988e-050.00.00.00.08.703765779643203e-050.00.00.0002180742979160.00.00.00.000301505903709517740.000109159729153025150.000243557260886147470.00.000446126737095999940.00.00.00.000402007871612690350.000327479187459075540.00060889315221536870.06.70013119354e-050.00.00.00.000201003935806345170.00.01.28487183714e-052.8048387566e-050.03.8546155114216895e-050.04.033523795445053e-054.3809924743689033e-050.00.00.00.00.00.00.00.00.03.99907164156e-051.44582422719e-054.489909737204044e-053.8163246288578975e-053.690980558618241e-050.04.337472681577159e-050.00.00013946483190510.000138620096537000020.00.00.00041839449571515480.000241204722967614150.000174655566644840280.00.00013946483190510.000138620096537000020.00.00.00041839449571515480.000241204722967614150.000174655566644840280.02.26560188761e-050.00.00.06.796805662821394e-050.00.00.00.00.00.00.00.00.00.00.04.6156361436999995e-050.00.00.00.000138469084310895480.00.00.00.00.00.00.00.00.00.00.00.00.00.00.00.00.00.00.02.11112903163e-050.00.00.06.333387094901754e-050.00.00.04.95411612757e-050.000138620096537000020.00.00.000148623483827027830.000241204722967614150.000174655566644840280.00.00.000340349817247999950.00.00.00.00025125491975793140.00036386576384341720.00040592876814357910.00.000340349817247999950.00.00.00.00025125491975793140.00036386576384341720.00040592876814357910.00.000340349817247999950.00.00.00.00025125491975793140.00036386576384341720.00040592876814357910.000230861010019800020.00013949546781780.00055300196048726999.408408472776204e-054.549698484500852e-050.000245727061393919255.346598978923682e-050.000119293352270766090.000230861010019800020.00013949546781780.00055300196048726999.408408472776204e-054.549698484500852e-050.000245727061393919255.346598978923682e-050.000119293352270766090.000194113716049000030.000106811901020.000442760078574896869.408408472776204e-054.549698484500852e-050.00014767636100058015.346598978923682e-050.000119293352270766090.03.26835667978e-050.00.00.09.805070039333912e-050.00.01.84483366073e-050.05.534500982186211e-050.00.00.00.00.01.82989573635e-050.05.489687209051099e-050.00.00.00.00.00.19588359737865930.21014731933136960.202429154888405350.189456125754939590.195765511492606570.21351048982100950.214072981586962640.20285848658614790.00098964120504190.000903774627740620.00142133088090872420.0009800736910988350.00056751904311855110.00119363792576710160.00050870553391701060.00100898042353608820.00.00.00.00.00.00.00.00.00.00.00.00.00.00.00.00.00.00.00.00.00.00.00.00.00.00.00.00.00.00.00.00.00.00.00.00.00.00.00.00.00.00.00.00.00.00.00.00.07.55216312825e-050.00.00.00.00.00.000226564893847579050.07.55216312825e-050.00.00.00.00.00.000226564893847579050.07.55216312825e-050.00.00.00.00.00.000226564893847579058.21715846006e-059.52625288182e-060.00019278948918989882.731113833922003e-052.6414126272576035e-052.8578758645451923e-050.00.08.21715846006e-059.52625288182e-060.00019278948918989882.731113833922003e-052.6414126272576035e-052.8578758645451923e-050.00.08.21715846006e-059.52625288182e-060.00019278948918989882.731113833922003e-052.6414126272576035e-052.8578758645451923e-050.00.03.58717656253e-056.38107732719e-050.000107615296875842950.00.00.000191432319815566830.00.03.58717656253e-056.38107732719e-050.000107615296875842950.00.00.000191432319815566830.00.03.58717656253e-056.38107732719e-050.000107615296875842950.00.00.000191432319815566830.00.00.00.00.00.00.00.00.00.00.0001229648420860.0001307532931450.000199404814799356080.000169489711458100760.00.000177356413946775120.00.00021490346548777720.0001229648420860.0001307532931450.000199404814799356080.000169489711458100760.00.000177356413946775120.00.00021490346548777720.0001229648420860.0001307532931450.000199404814799356080.000169489711458100760.00.000177356413946775120.00.00021490346548777720.00074863301273000010.000592918542060.00092152128004362620.00078327284130151410.0005411049168459750.00070253802806101220.00050870553391701060.00056751206420073180.00074863301273000010.000592918542060.00092152128004362620.00078327284130151410.0005411049168459750.00070253802806101220.00050870553391701060.00056751206420073180.00074863301273000010.000592918542060.00092152128004362620.00078327284130151410.0005411049168459750.00070253802806101220.00050870553391701060.00056751206420073180.03.12441350994e-050.00.00.09.373240529829567e-050.00.00.03.12441350994e-050.00.00.09.373240529829567e-050.00.00.03.12441350994e-050.00.00.09.373240529829567e-050.00.00.00.00.00.00.00.00.00.00.00.00.00.00.00.00.00.00.00.00.00.00.00.00.00.00.00.00.00.00.00.00.00.00.00.00.00.00.00.00.00.00.00.00.00.00.00.00.00.00.00.00.00.00.00.00.00.00.004211769490395780.00769835252882553060.0043564111731250450.0034578735098751590.00482102378819090.0070719626752524140.0078785506595754290.0081445442516520312.752347174292e-050.02.2156090533261788e-051.8832190162011194e-054.158213453355153e-050.00.00.02.752347174292e-050.02.2156090533261788e-051.8832190162011194e-054.158213453355153e-050.00.00.01.97339809763e-050.02.2156090533261788e-051.8832190162011194e-051.821366223370439e-050.00.00.00.00.00.00.00.00.00.00.07.78949076662e-060.00.00.02.336847229984714e-050.00.00.00.00.00.00.00.00.00.00.00.00.00.00.00.00.00.00.00.00.00.00.00.00.00.00.00.00.00.00.00.00.00.00.01.9600850985e-050.0003026776910980.05.8802552954851284e-050.00.000369750171835827430.0001866980412680840.00035158486018865581.9600850985e-050.0003026776910980.05.8802552954851284e-050.00.000369750171835827430.0001866980412680840.00035158486018865580.00.00.00.00.00.00.00.00.00.000119723251221000010.00.00.00.000149032803673691846.683248723654602e-050.000143304462752348641.9600850985e-050.000182954439877000020.05.8802552954851284e-050.00.00022071736816213560.000119865554031537990.000208280397436307180.00.00.00.00.00.00.00.00.00.00.00.00.00.00.00.00.00.00.00.00.00.00.00.00.00.00.00.00.00.00.00.00.00.00.00.00.00.00.00.00.00.00.00.00.00.00.00.00.00.00.00.00.00.00.00.00.00.00.00.00.00.00.00.00.00.00.00.00.00.00.00.00.00.000105571821530.00.00.00.00.000149704771409863090.00016701069317907250.00.000105571821530.00.00.00.00.000149704771409863090.00016701069317907250.00.000105571821530.00.00.00.00.000149704771409863090.00016701069317907250.02.03087868192e-050.00.00.00.06.0926360457502414e-050.00.02.03087868192e-050.00.00.00.06.0926360457502414e-050.00.02.03087868192e-050.00.00.00.06.0926360457502414e-050.00.0005534478479110.00169618869808800010.00076357944737814030.00039290796747105170.0005038561288832950.00135525380960338760.00176640288993077080.00196690939473206970.0005534478479110.00169618869808800010.00076357944737814030.00039290796747105170.0005038561288832950.00135525380960338760.00176640288993077080.00196690939473206970.00.0002499460831530.00.00.00.000134002623870896770.00029109261107473380.000324743014514863330.0001417252448140.0005351034131350.00030132283125236030.00.000123852903189189840.00053601049548358710.00058218522214946760.000487114521772294940.00.0001082476715050.00.00.00.00.00.000324743014514863330.000411722603096999970.0008028915302950.000462256616125780.00039290796747105170.00038000322569410520.00068524069024890390.00089312505670656950.00083030884393004818.869812228259999e-050.03.088730616482053e-050.000143858637525080359.134842315796348e-050.00.00.08.869812228259999e-050.03.088730616482053e-050.000143858637525080359.134842315796348e-050.00.00.00.00.00.00.00.00.00.00.00.00.00.00.00.00.00.00.02.75106978531e-050.03.088730616482053e-052.6253531615377788e-052.539125577910498e-050.00.00.00.00.00.00.00.00.00.00.02.19857224596e-050.00.00.06.59571673788585e-050.00.00.03.92017019699e-050.00.00.000117605105909702570.00.00.00.04.7080475405e-060.00.01.4124142621508395e-050.00.00.00.04.7080475405e-060.00.01.4124142621508395e-050.00.00.00.04.7080475405e-060.00.01.4124142621508395e-050.00.00.00.00.00065023127105070990.00115604302714389980.00067577201715278630.00080668746451377120.00046823433148735930.00127970450397903170.00115199406840545740.0010364305090494220.00065023127105070990.00115604302714389980.00067577201715278630.00080668746451377120.00046823433148735930.00127970450397903170.00115199406840545740.0010364305090494221.08218782903e-050.00.03.2465634870847464e-050.00.00.00.00.01.17890871441e-050.00.00.03.536726143220149e-050.00.01.07698176686e-050.00.00.03.230945300587561e-050.00.00.08.943614069499999e-050.00.00.0001363940873272290.0001319143347577170.00.00.02.13484573128e-050.00.03.255734570381596e-053.148802623453979e-050.00.00.00.00.00.00.00.00.00.00.00.0002230158942520.000240268564356999990.00036165169113681220.000307395991620310730.00.000158687317741851430.000172357467083723930.00038976090824596851.95509760461e-050.0002877647585970.05.8652928138172275e-050.00.000306876237872282670.000333312150085573030.000223105887834638870.01.2692991762e-050.00.00.00.03.8078975285939015e-050.09.75219710151e-060.00.00.02.9256591304533033e-050.00.00.00.02.4809029353e-050.00.00.00.07.44270880588808e-050.00.00.00.00.00.00.00.00.00.00.00.00.00.00.00.00.00.00.00.00.00.00.00.00.01.07311921594e-050.00.03.2193576478075e-050.00.00.00.01.9036155890999998e-050.00.02.9030983322798107e-052.807748435019417e-050.00.00.00.00.00.00.00.00.00.00.00.00.00.00.00.00.00.00.00.00.00.00.00.00.00.00.00.07.19519274737e-050.00.00.00.000122232123125480144.425394425122642e-054.936971504448935e-050.01.14859391889e-050.00.00.03.4457817566802024e-050.00.00.04.53482724832e-050.00.00.06.330832623821895e-053.438101705607092e-053.835547415529881e-050.0002357685616340.0004320238346610.000314120326015974070.00017799691705252280.000215188441834499730.0005587754200021950.000455183426584043160.0002821126573970820.01.7908622124e-050.00.00.00.00.05.372586637194429e-059.00469790541e-050.00024714802950260.00.00015247653966401110.000117664397498419830.00023671508919938180.00025274831536730130.00025198068394051039.00469790541e-050.00024714802950260.00.00015247653966401110.000117664397498419830.00023671508919938180.00025274831536730130.00025198068394051033.93612181704e-050.00.06.0027606141410684e-055.805604836993274e-050.00.00.00.00.00.00.00.00.00.00.00.06.15586768626e-050.00.00.06.55447616759821e-050.00.000119131268911702570.00.00.00.00.00.00.00.00.00.000185589352640.00.00.00.000171170327523399680.00025274831536730130.000132849415028807725.06857608837e-050.00.09.24489335226004e-055.960834912848708e-050.00.00.00.00.00.00.00.00.00.00.00.00.00.00.00.00.00.00.00.00.00.00.00.00.00.00.05.7162878395000006e-050.000178983046574999980.00.00.000171488635185032120.00018554209459047250.000201525653820969570.000149881391314552285.7162878395000006e-050.000178983046574999980.00.00.000171488635185032120.00018554209459047250.000201525653820969570.000149881391314552285.7162878395000006e-050.000178983046574999980.00.00.000171488635185032120.00018554209459047250.000201525653820969570.000149881391314552280.000148371741508910.00.00015859096381703170.00013479883484386960.000151725425866974870.00.00.00.000148371741508910.00.00015859096381703170.00013479883484386960.000151725425866974870.00.00.07.117982941910001e-060.00.00.02.1353948825722388e-050.00.00.00.0001412537585670.00.00015859096381703170.00013479883484386960.000130371477041252480.00.00.00.00.00.00.00.00.00.00.00.00070255649756510.000381712763521999950.00085828085022717070.00039084265484057030.00085854598762879040.00031749831470948130.00034484926776378940.00048279070809126780.00070255649756510.000381712763521999950.00085828085022717070.00039084265484057030.00085854598762879040.00031749831470948130.00034484926776378940.00048279070809126780.0001869331722080.00.000248798668006536040.000158605051089231920.000153395797527895680.00.00.00.000257357173889000040.00.00042373523144863170.00.000348336290219596440.00.00.02.47855922132e-050.000381712763521999950.07.435677663968291e-050.00.00031749831470948130.00034484926776378940.00048279070809126780.0001824071971150.00.000185746950772002940.000157880827111655480.000203593813461681950.00.00.05.10733621399e-050.00.00.00.00015322008641961630.00.00.00.02.83104134939e-050.00.00.08.493124048155428e-050.00.00.02.83104134939e-050.00.00.08.493124048155428e-050.00.00.02.83104134939e-050.00.00.08.493124048155428e-050.00.00.02.17662776799e-050.00.00.00.04.191733599476167e-052.338149704507016e-050.02.17662776799e-050.00.00.00.04.191733599476167e-052.338149704507016e-050.02.17662776799e-050.00.00.00.04.191733599476167e-052.338149704507016e-050.00.00.00.00.00.00.00.00.00.00.00.00.00.00.00.00.00.00.00.00.00.00.00.00.00.00.00.00.00.00.00.00.00.00.00.00.00.00.00.00.00.00.00.00.00.00.00.00.00.00.00.00.00.00.00.00.00.00.00.00.00.00.00.00.00.00.00.00.00.00.00.00.00.0003843513623750.00.00.00.00043855404175929850.00071450004536525570.00.00.0003843513623750.00.00.00.00043855404175929850.00071450004536525570.00.00.0003843513623750.00.00.00.00043855404175929850.00071450004536525570.00.00073453487595380.00110590622121270.0008057105112337840.000372638032178861040.0010252560844483070.00103069451221086460.00094419517160097490.00134282897982904740.00073453487595380.00110590622121270.0008057105112337840.000372638032178861040.0010252560844483070.00103069451221086460.00094419517160097490.00134282897982904748.21577983504e-056.04845165568e-059.224168303643685e-057.840340393980171e-057.582830807501421e-058.204242277810006e-050.09.941112689230508e-050.00.00.00.00.00.00.00.02.5105318214000002e-050.00.00.07.531595464207491e-050.00.00.05.41804576609e-052.64478862903e-058.920741714708035e-050.07.33339558357045e-057.934365887092572e-050.00.00.000182808503559000010.000232723452270999980.00017667324139510050.000150168373499673680.000221583895781825480.000157138294607965270.00025573453226580890.000285297529940483377.81519873104e-050.000172754126557000028.920741714708035e-050.00.000145248544784266370.000157151634021769020.000170689491595816920.000190421254054449760.02.61044072476e-050.00.00.07.831322174273189e-050.00.05.49028637631e-050.09.039684937570812e-050.07.431174191351391e-050.00.00.00.00.00.00.00.00.00.00.00.00.00.00.00.00.00.00.00.0001112895887090.000391227010930000030.000183236856842651550.00.000150631909284149820.00032595232833461380.00035403155400981140.00049369715044489350.000145938358386999980.000196164821360000038.474704628972636e-050.000144066254739385630.000209001774131757830.00015075295185475890.000163739593729537770.00027400191849691587.5828308075e-050.000480642548748000040.00.00.000227484924225042520.00041021211389050040.00053465989789236810.00049705563446152557.5828308075e-050.000480642548748000040.00.00.000227484924225042520.00041021211389050040.00053465989789236810.00049705563446152557.5828308075e-050.000480642548748000040.00.00.000227484924225042520.00041021211389050040.00053465989789236810.00049705563446152553.83037497355e-050.00.00011491124920640860.00.00.00.00.03.83037497355e-050.00.00011491124920640860.00.00.00.00.03.83037497355e-050.00.00011491124920640860.00.00.00.00.00.01.26749723052e-050.00.00.03.8024916915546115e-050.00.00.01.26749723052e-050.00.00.03.8024916915546115e-050.00.00.01.26749723052e-050.00.00.03.8024916915546115e-050.00.01.7703994438e-055.07940064413e-060.05.311198331405922e-050.00.00.01.5238201932397131e-051.7703994438e-055.07940064413e-060.05.311198331405922e-050.00.00.01.5238201932397131e-050.00.00.00.00.00.00.00.00.05.07940064413e-060.00.00.00.00.01.5238201932397131e-050.00.00.00.00.00.00.00.00.00.00.00.00.00.00.00.00.00.00.00.00.00.00.00.00.00.00.00.00.00.00.00.00.00.00.00.00.00.00.00.01.7703994438e-050.00.05.311198331405922e-050.00.00.00.00.00.00.00.00.00.00.00.00.000232033617276840.00077563016900200029.259548716374948e-050.00027866184117969950.00032484352348728170.00043016681066451780.00086429524582254450.00103242845051836650.000232033617276840.00077563016900200029.259548716374948e-050.00027866184117969950.00032484352348728170.00043016681066451780.00086429524582254450.00103242845051836650.01.41897609583e-050.00.00.00.02.012160906046547e-052.244767381439147e-051.9669700908e-050.02.208392085725768e-051.877084752304699e-051.815433434369232e-050.00.00.00.00.00.00.00.00.00.00.02.63996072149e-050.02.248677845166868e-053.822653525423168e-051.8485507938685053e-050.00.00.00.08.89772691273e-050.00.00.08.094118220389738e-058.791387582793975e-059.807674935012648e-059.07598889314e-060.02.7227966679430146e-050.00.00.00.00.00.000158365012963999980.00052900484250400010.00.000203987617330103580.00027110742156222470.000239607340696305480.00059114637410350650.00075626081271162980.01.41679975212e-050.00.00.00.02.00907476968758e-052.2413244866823385e-051.85233072968e-053.45017599445e-052.079682117539297e-051.7676841072317256e-051.709625964267958e-051.849729470610538e-054.01814953937516e-054.482648973364677e-050.05.93773024848e-050.00.00.07.265202499024523e-053.9455323790250065e-056.602455867395563e-050.00.00.00.00.00.00.00.00.01.51086130316e-050.00.00.00.04.532583909468174e-050.00.02.03026234303e-050.00.00.01.8468968067964333e-052.0059980855073543e-052.237892136779303e-050.08.83028227642e-050.00.00.00.000171675959407554559.323250888514605e-050.00.08.83028227642e-050.00.00.00.000171675959407554559.323250888514605e-050.00.03.10775029617e-050.00.00.00.09.323250888514605e-050.00.05.72253198025e-050.00.00.00.000171675959407554550.00.00.00025586895526470.00065652725802070.00025748599903727760.000218857441959169330.00029126342479868180.00068092247794050160.00057090108559064070.00071775821053142660.00025586895526470.00065652725802070.00025748599903727760.000218857441959169330.00029126342479868180.00068092247794050160.00057090108559064070.00071775821053142660.06.7095664156e-060.00.00.00.00.02.0128699246789046e-050.04.7466186307999995e-050.00.00.06.626503378231159e-053.5986723896601703e-054.014680124496937e-050.05.94042099722e-050.00.00.00.00.00.000178212629916693260.000211951877600999970.0004476450236330.000208178626650527720.000176947258762257140.000250729747391310560.00052694663580633030.00048728102533635520.000328707409756992840.03.24743014515e-050.00.00.00.00.09.742290435445898e-050.00.00.00.00.00.00.00.04.39170776637e-056.28279702404e-054.9307372386749875e-054.191018319691218e-054.053367740737122e-058.77108083518597e-054.763333635768371e-055.313976601152308e-050.00.00.00.00.00.00.00.00.00046276076697270.00.00057644125121061370.00030322212582883120.0005086189238778250.00.00.00.00046276076697270.00.00057644125121061370.00030322212582883120.0005086189238778250.00.00.00.0001619193380060.00.000212863293950397560.000130453354956491980.0001424413651116950.00.00.00.0001740909227320.00.000255137389702816.87393660784345e-050.000198396012413942120.00.00.01.54202963787e-050.00.01.1857304916821864e-053.44035842192194e-050.00.00.00.0001113302098560.00.000108440567557406189.217209987708284e-050.000133377962132968460.00.00.02.65170311885e-055.05272183011e-050.04.043965045316088e-053.9111443112375736e-054.231661806449372e-050.00.00010926503683864762.65170311885e-055.05272183011e-050.04.043965045316088e-053.9111443112375736e-054.231661806449372e-050.00.00010926503683864762.65170311885e-053.11972769609e-050.04.043965045316088e-053.9111443112375736e-054.231661806449372e-050.05.127521281813631e-050.00.00.00.00.00.00.00.00.01.93299413402e-050.00.00.00.00.05.798982402051129e-052.58704834549e-050.00.07.761145036465219e-050.00.00.00.02.58704834549e-050.00.07.761145036465219e-050.00.00.00.02.58704834549e-050.00.07.761145036465219e-050.00.00.00.00.01767886358948120.021344714556362980.0190090176653344640.015784850338483960.0182427227646337340.021367211030905110.019408071633603690.0232588610045919337.38928396896e-050.04.290989685555764e-057.294493910854968e-050.000105823683104687510.00.00.07.38928396896e-050.04.290989685555764e-057.294493910854968e-050.000105823683104687510.00.00.06.2134652678e-050.04.290989685555764e-057.294493910854968e-057.054912206979167e-050.00.00.01.17581870116e-050.00.00.03.5274561034895836e-050.00.00.00.0001825512938270.00.000211267424178354590.000146923092652064650.000189463364652018470.00.00.00.0001825512938270.00.000211267424178354590.000146923092652064650.000189463364652018470.00.00.00.00.00.00.00.00.00.00.00.0001825512938270.00.000211267424178354590.000146923092652064650.000189463364652018470.00.00.04.65918369316e-050.00.07.105462093729364e-056.872088985732477e-050.00.00.04.65918369316e-050.00.07.105462093729364e-056.872088985732477e-050.00.00.02.29010723901e-050.00.03.4925152664093486e-053.377806450614269e-050.00.00.02.36907645415e-050.00.03.6129468273200156e-053.4942825351182085e-050.00.00.05.79466983178e-050.00.000173840094953284820.00.00.00.00.05.79466983178e-050.00.000173840094953284820.00.00.00.00.05.79466983178e-050.00.000173840094953284820.00.00.00.00.00.00.00.00.00.00.00.00.00.00.00.00.00.00.00.00.00.00.00.00.00.00.00.00.00.00.00.00.00.00.00.00.00.00.00.00.00.00.00.00.00.00.00.00.00.00.00.00.00.00.00032067504216910.00.00.00.000169038989175754430.000299075180171717270.00049391095715840490.00.00032067504216910.00.00.00.000169038989175754430.000299075180171717270.00049391095715840490.04.26731289736e-050.00.00.02.9201540310849177e-056.343422517367081e-053.5383621436244176e-050.00.0001487724823540.00.00.00.000139837448864905269.775498133106732e-050.000208725016864573480.04.59619912223e-050.00.00.00.00.000137885973666979150.00.08.32674396192e-050.00.00.00.00.00.000249802318857587232.92565913045e-060.00.00.08.776977391359911e-060.00.00.02.92565913045e-060.00.00.08.776977391359911e-060.00.00.02.92565913045e-060.00.00.08.776977391359911e-060.00.00.00.00036808638204499990.00085817538324940.00032673560015316180.000207663069894609920.00056986047608702460.00057105224981955780.00087542023069353460.00112805366923378010.00036808638204499990.00085817538324940.00032673560015316180.000207663069894609920.00056986047608702460.00057105224981955780.00087542023069353460.00112805366923378010.000198444085890999950.0003194605566260.00032673560015316180.00.0002685966575187250.000290608099960980930.000315642590322000470.000352130979594430130.0001696422961540.0003220969421960.00.000207663069894609920.000301263818568299640.000217301552223075820.00035403155400981140.00039495772035591480.05.04781887847e-050.00.00.00.00.00.000151434566354081320.00.000117775641277000010.00.00.06.314259763550109e-050.000137164057574481910.00015302026861956910.04.83640543657e-050.00.00.00.06.858202878724094e-057.651013430978455e-050.00.00.00.00.00.00.00.00.00.00.00.00.00.00.00.00.00.00.00.00.00.00.00.00.00198650250588245038.452075934087e-050.00208629020282059950.00144124855063152870.0024319687641957559.521905879235094e-059.655279363553841e-056.179042559479429e-050.00198650250588245038.452075934087e-050.00208629020282059950.00144124855063152870.0024319687641957559.521905879235094e-059.655279363553841e-056.179042559479429e-050.00.00.00.00.00.00.00.00.00.00.00.00.00.00.00.01.3317516472000002e-050.00.00.03.9952549415867686e-050.00.00.00.00.00.00.00.00.00.00.00.00.00.00.00.00.00.00.00.00.00.00.00.00.00.00.00.00.00.00.00.00.00.00.03.31984871534e-050.05.4383131308915304e-051.5408155587100067e-052.980417456424354e-050.00.00.00.00.00.00.00.00.00.00.00.000469999631241999940.00.00052086925482763260.000298303335324159150.00059082630357539540.00.00.04.11710761968e-050.07.575154975617997e-053.2193576478075e-051.5568102356183083e-050.00.00.00.0001100609895240.00.000105020293709402160.000121473942035125520.000103688732828062560.00.00.00.00.00.00.00.00.00.00.00.00.00.00.00.00.00.00.00.00.00.00.00.00.00.00.01.2768340535e-050.00.00.03.830502160490408e-050.00.00.08.67158634109e-056.2423582548599995e-066.31655003401687e-055.368928748051639e-050.000143292802411889831.8727074764566315e-050.00.04.71521205035e-060.00.00.01.41456361510496e-050.00.00.00.00.00.00.00.00.00.00.04.333342148899999e-050.05.452893058856387e-052.3174196472823432e-055.229713740562306e-050.00.00.05.31318036417e-050.08.748082197649171e-050.07.191458894856185e-050.00.00.05.1291474885e-050.03.5037538517716324e-050.00.000118836886137373930.00.00.04.99642003719e-050.07.004148088034294e-053.7787870095576564e-054.2063250139724845e-050.00.00.00.00.00.00.00.00.00.00.02.9354616084000006e-050.02.5203582539695565e-052.142249141106106e-054.1437774301215935e-050.00.00.00.00.00.00.00.00.00.00.04.8185953825e-060.01.4455786147501294e-050.00.00.00.00.01.20839400001e-050.01.3710341159106385e-050.02.254147884130857e-050.00.00.00.00.00.00.00.00.00.00.00.06.14119432647e-060.00.00.00.01.8423582979413533e-050.00.00.00.00.00.00.00.00.00.00.00.00.00.00.00.00.00.07.580536746739999e-060.00.00.00.02.2741610240213573e-050.00.00.00.00.00.00.00.00.00.00.00.00.00.00.00.00.08.95138498611e-050.00.000120776523483941822.6928271913903856e-050.000120836754185454680.00.00.00.000370132514604000050.00.00040401220471008480.00037829792877658040.000328087410324686630.00.00.00.00.00.00.00.00.00.00.00.00.00.00.00.00.00.00.06.93335219149e-050.00.00.000105736700726154580.000102263865018597120.00.00.00.00.00.00.00.00.00.00.00.00.00.00.00.00.00.00.00.00.00.00.00.00.00.00.00.000379979102301000046.45566700128e-050.00037267200059752810.00026803024137560120.00049923506492935197.649198402778463e-055.53876004159113e-056.179042559479429e-056.16183487628e-050.06.918126227732762e-055.8802552954851284e-055.687123105626064e-050.00.00.00.00.00.00.00.00.00.00.00.00.0001356160335130.00.00.00.00.00040684810054026790.00.00.0001356160335130.00.00.00.00.00040684810054026790.00.00.00.00.00.00.00.00.00.00.00.00.00.00.00.00.00.00.0001356160335130.00.00.00.00.00040684810054026790.00.00.00.00.00.00.00.00.00.00.00.00.00.00.00.00.00.000188706193085999980.00.000211867615724315850.000180082818424232030.000174168145109798220.00.00.00.000188706193085999980.00.000211867615724315850.000180082818424232030.000174168145109798220.00.00.00.000188706193085999980.00.000211867615724315850.000180082818424232030.000174168145109798220.00.00.02.4205779125e-050.00.00.07.261733737476927e-050.00.00.02.4205779125e-050.00.00.07.261733737476927e-050.00.00.00.00.00.00.00.00.00.00.01.21028895625e-050.00.00.03.630866868738464e-050.00.00.01.21028895625e-050.00.00.03.630866868738464e-050.00.00.00.00.0005146114610230.00.00.00.000398685492508453150.00054128791315549660.00060386097740367140.00.0005146114610230.00.00.00.000398685492508453150.00054128791315549660.00060386097740367140.00.0005146114610230.00.00.00.000398685492508453150.00054128791315549660.00060386097740367140.0002044236470140.00.00017458618978141050.00024732404247104830.000191360708790164540.00.00.00.0002044236470140.00.00017458618978141050.00024732404247104830.000191360708790164540.00.00.00.0002044236470140.00.00017458618978141050.00024732404247104830.000191360708790164540.00.00.00.00.000462656135091999960.00.00.00.00054819255219912310.000396944469647364330.00044283138342935910.00.000462656135091999960.00.00.00.00054819255219912310.000396944469647364330.00044283138342935910.00.000462656135091999960.00.00.00.00054819255219912310.000396944469647364330.00044283138342935910.00.0001567564090140.00.00.00.0002254249747360880.000244844252305850830.00.00.0001567564090140.00.00.00.0002254249747360880.000244844252305850830.00.00.0001567564090140.00.00.00.0002254249747360880.000244844252305850830.00.00.00.00.00.00.00.00.00.00.00.00.00.00.00.00.00.00.00.00.00.00.00.00.00.07.44459021505e-050.00.00.00.000223337706451494570.00.00.07.44459021505e-050.00.00.00.000223337706451494570.00.00.07.44459021505e-050.00.00.00.000223337706451494570.00.04.26400232904e-054.11923506238e-050.000127920069871285040.00.00.00.000123577051871349240.04.26400232904e-054.11923506238e-050.000127920069871285040.00.00.00.000123577051871349240.00.00.00.00.00.00.00.00.04.26400232904e-054.11923506238e-050.000127920069871285040.00.00.00.000123577051871349240.00.00011429442741170.00026412452926625.7700116622792396e-050.000144972331813218230.000140210833799082830.000305661545096120250.000302895242033604930.000183816800668790520.00011429442741170.00026412452926625.7700116622792396e-050.000144972331813218230.000140210833799082830.000305661545096120250.000302895242033604930.000183816800668790521.92333722076e-056.99005332532e-055.7700116622792396e-050.00.00.000153960461468689925.574113829090647e-050.09.50610552041e-050.0001942239960130.00.000144972331813218230.000140210833799082830.000151701083627430330.00024715410374269850.000183816800668790520.00.000107202099097000010.00.00.00.000321606297290152240.00.00.00.000107202099097000010.00.00.00.000321606297290152240.00.00.00.000107202099097000010.00.00.00.000321606297290152240.00.00.000210589828857999960.00206588176355830984.791352440408557e-050.000296802092428073640.00028705386974301470.0021785628482306070.001395014450672530.00262406799177406940.000210589828857999960.00206588176355830984.791352440408557e-050.000296802092428073640.00028705386974301470.0021785628482306070.001395014450672530.00262406799177406940.05.56027484941e-060.00.00.01.6680824548244413e-050.00.00.02.19496905629e-050.00.00.01.9967278391358787e-052.16873634078858e-052.41944298893524e-050.01.35447718238e-050.00.00.00.01.920699046680795e-052.1427325004646398e-050.00.00.00.00.00.00.00.00.000210589828857999960.00146051752601999984.791352440408557e-050.000296802092428073640.00028705386974301470.00145752753361574610.00116794047322416680.00175608457122058820.02.08520744721e-050.00.00.01.8273085073304103e-050.04.42831383429359e-050.04.22457118551e-050.00.00.03.299654212963258e-055.3758553344855074e-053.998204009074924e-050.00.000365251355720999940.00.00.00.000457983651204330662.5634378666072445e-050.00061213603729367890.00.0001359603582540.00.00.00.000175133933267990430.0001067866915627420.000125960449932118630.00.00.00.00.00.00.00.00.00012253196722170.0004323134706510.00.000125275004121204920.00024232089754406710.00039326857005589270.000427146766250968050.00047652507564681020.00012253196722170.0004323134706510.00.000125275004121204920.00024232089754406710.00039326857005589270.000427146766250968050.00047652507564681022.01934081287e-050.0001203741143140.00.06.058022438601677e-050.000131089523351964237.119112770849468e-050.000158841691882270070.0001023385590930.0003119393563370.00.000125275004121204920.000181740673158050330.000262179046703928460.00035595563854247340.000317683383764540140.0004249444505520.00154945584932259980.00030832258728808990.000441178503673457350.00052533226069427290.00128639120403584120.00151775417133233490.00184422217261401680.0004249444505520.00154945584932259980.00030832258728808990.000441178503673457350.00052533226069427290.00128639120403584120.00151775417133233490.00184422217261401680.09.72372040405e-050.00.00.00.00.000137885973666979150.00015382563845440890.000167738838298999970.000250687993290.00018832676953272520.000160073616377095140.00015481612898648738.375163991931047e-050.00036386576384341720.000304446576107684340.04.96180587059e-050.00.00.00.00.00014885417611776160.00.0002572056122530.00106147233942999990.000119995817755364720.00028110488729636220.000370516131707785670.00120263956411653080.00078317923527877320.00119859821890873330.09.04402538562e-050.00.00.00.08.396902242540398e-050.000187351739143190350.00.00.00.00.00.00.00.00.00042714179298630.00119840845873360.00026132806055397840.000445211892092825540.00057488542631272580.00093272382620024460.00134735902133410850.00131514252866552740.00042714179298630.00119840845873360.00026132806055397840.000445211892092825540.00057488542631272580.00093272382620024460.00134735902133410850.00131514252866552740.00.00.00.00.00.00.00.04.90737265502e-058.60883967531e-050.07.483961285162891e-057.238156679887718e-057.831322174273189e-058.505952921014947e-059.489243930629124e-050.000179806321359000030.0006246669059090.000174961643952983420.00014871355327936580.000215743766845685570.00062246380120674630.00059157530637768470.0006599616101431092.49465376172e-055.4457583651000004e-050.07.483961285162891e-050.07.831322174273189e-058.505952921014947e-050.04.82543778659e-055.99839895055e-050.00.00.000144763133597754370.08.505952921014947e-059.489243930629124e-050.0001250608295940.000373211582915000048.6366416600995e-050.000146819113110201940.000141996959070408760.000153633581508034490.00050060512732597530.00046539603990983580.00.00.00.00.00.00.00.00.00.00.00.00.00.00.00.00.00.00.00.00.00.00.00.03.2587184346e-060.09.776155303789627e-060.00.00.00.00.03.2587184346e-060.09.776155303789627e-060.00.00.00.00.03.2587184346e-060.09.776155303789627e-060.00.00.00.00.00.00.00.00.00.00.00.00.00.00.00.00.00.00.00.00.00.00.00.00.00.00.00.00.00.00.00.00.00.00.00.00.00.00.00.00.00.00.00.00.00.00.00.00.00.00.00.00.00.00.00.00.00.00.00.00.00.00.00.00.00.00.00.00.00.00.00.00.00.00.00.00.00.00.00.00.00.00.00.00.00.00.00.00.00.00.00.00.00.00.00.00.00.00.00.00.00.00.00.00.00.00.00.00.00.00.00.00.00.00.00.00.00.00.00.00.00.00.00.00.01.45801369961e-050.04.3740410988245855e-050.00.00.00.00.01.45801369961e-050.04.3740410988245855e-050.00.00.00.00.01.45801369961e-050.04.3740410988245855e-050.00.00.00.00.00.00111266631140350.00247461956918560.00135545284595263610.00100848629440734750.00097405979385090730.0024398178818014570.0021430826303398680.0028409581954182850.00111266631140350.00247461956918560.00135545284595263610.00100848629440734750.00097405979385090730.0024398178818014570.0021430826303398680.0028409581954182852.82490154299e-050.000113304739485999998.474704628972636e-050.00.07.537647592737943e-058.186979686476888e-050.00018266794566461060.0002278716293870.000166762428930.000255840139742570070.000217458497719827420.000210316250698624320.000151701083627430330.000164769402495132350.000183816800668790520.00.000280779776840.00.00.00.000227551625441145450.00024715410374269850.00036763360133758112.82490154299e-050.08.474704628972636e-050.00.00.00.00.02.82490154299e-050.08.474704628972636e-050.00.00.00.00.00.000250458474160.00.0002542411388691790.000288132509478771260.000209001774131757830.00.00.02.40110424566e-050.00.07.203312736969282e-050.00.00.00.05.14714347779e-050.000174194054707000048.474704628972636e-050.06.966725804391929e-057.537647592737943e-058.186979686476888e-050.00036533589132922124.72334618045e-052.51254919758e-050.07.203312736969282e-056.966725804391929e-057.537647592737943e-050.00.00.00.0005077750293350.00.00.00.00046694258464103880.00058903723978028520.00046734526358348420.02.48153007168e-050.00.00.07.444590215049821e-050.00.00.02.97783608602e-050.00.00.08.933508258059785e-050.00.00.00.00.00.00.00.00.00.04.6156361436999995e-050.00.00.00.000138469084310895480.00.00.05.25887374958e-050.0002732491041898.52800465808567e-057.248616590660913e-050.00.000379252709068575670.000164769402495132350.000275725201003185770.02.51254919758e-050.00.00.07.537647592737943e-050.00.00.000328128123594999970.00085370979016999990.00042110333560112470.000286342866562754040.000276938168621790970.00074908299058265280.0008136128880970820.0009984334918314123.25168522934e-050.09.755055688026056e-050.00.00.00.00.03.25168522934e-050.09.755055688026056e-050.00.00.00.00.03.25168522934e-050.09.755055688026056e-050.00.00.00.00.00.06.59029297726e-050.00.00.00.000197708789317716520.00.00.06.59029297726e-050.00.00.00.000197708789317716520.00.00.06.59029297726e-050.00.00.00.000197708789317716520.00.08.99593002511e-050.00030227952786749.897465260114023e-050.00.000170903248152174160.00046671461807089730.000160725981575006380.00027939798395629158.99593002511e-050.00030227952786749.897465260114023e-050.00.000170903248152174160.00046671461807089730.000160725981575006380.00027939798395629153.2991550867e-050.09.897465260114023e-050.00.00.00.00.00.00.000113343980169999990.00.00.00.00.000160725981575006380.000179305958934587083.20309232386e-050.00.00.09.609276971575076e-050.00.00.02.49368261455e-050.00.00.07.48104784364234e-050.00.00.00.08.84335797944e-050.00.00.00.000165208714361379560.00.000100092025021704420.00.000100501967903000010.00.00.00.000301505903709517740.00.00.0012968032997280.0033423402924740.00164357907955832940.00140237920697360020.00084445161265356720.0035535800712474650.00291696079083028550.0035564800153444920.0012968032997280.0033423402924740.00164357907955832940.00140237920697360020.00084445161265356720.0035535800712474650.00291696079083028550.0035564800153444920.00.0002096616970560.00.00.00.000301505903709517740.000327479187459075540.00.00.001006433671250.00.00.00.00110200759211310890.00079999577728930340.00111729764434484440.000136964923297000020.00.000410894769889582340.00.00.00.00.00.0003659756471970.0003355884399060.000410894769889582340.00034925152664093490.000337780645061426840.000365461701466082140.000198472234823682140.00044283138342935910.0003659756471970.000381271152589999960.000410894769889582340.00034925152664093490.000337780645061426840.00050250983951586280.000198472234823682140.00044283138342935910.000118208209017000010.0006118898353830.00.000354624627050795450.00.00055662628377141720.00060457696146290860.00067446626091548530.000309678873020.0004017458515140.000410894769889582340.00034925152664093490.000168890322530713440.000365461701466082140.000396944469647364330.00044283138342935910.00.0003957496447750.00.00.00.000360007049205394340.00039101992532426930.0004362219597960850.000182260343470799990.00049069257965820010.00.000229106539068509880.00031767449134388420.00052185808371596060.00038273395263465950.00056748570262212730.000182260343470799990.00049069257965820010.00.000229106539068509880.00031767449134388420.00052185808371596060.00038273395263465950.00056748570262212730.00.00.00.00.00.00.00.00.00.0002540699269780.00.00.00.00052185808371596060.000240351697217670130.06.80842692678e-050.00.00.000103831534947304960.000100421272856099880.00.00.08.21451509644e-050.0001533552130610.00.000125275004121204920.000121160448772033540.00.000142382255416989350.000317683383764540140.08.32674396192e-050.00.00.00.00.00.000249802318857587233.20309232386e-050.00.00.09.609276971575076e-050.00.00.00.08.46783231795e-050.00.00.00.000114859391889340090.00.000139175577649227130.08.46783231795e-050.00.00.00.000114859391889340090.00.000139175577649227130.08.46783231795e-050.00.00.00.000114859391889340090.00.000139175577649227130.00.00.00.00.00.00.00.00.00.00.00.00.00.00.00.00.00.00.00.00.00.00.00.00.00167199739009019990.00158803840809270.00210733021726388670.00133959230756357630.00156906964544487660.00155558370194613030.00155163649142846030.00165689503090025980.00167199739009019990.00158803840809270.00210733021726388670.00133959230756357630.00156906964544487660.00155558370194613030.00155163649142846030.00165689503090025980.0004242878760240.00.00069287694988523530.000316629131295353030.00026335754689129930.00.00.00.01.52700477045e-050.00.00.00.00.04.5810143113381955e-050.00.00.00.00.00.00.00.03.78595497101e-050.04.250635550581885e-053.6129468273200156e-053.4942825351182085e-050.00.00.00.04.35844310483e-050.00.00.05.911880464892504e-050.07.163448849592572e-050.000124507178942999980.00.00013978894233356920.000118817529681967530.000114915064814712230.00.00.00.07.05276967742e-050.00.00.00.000211583090322468550.00.07.251595447800001e-050.0002596245594164.2110333560112455e-057.158571664068851e-050.000103851813233171620.000307873109129470340.00024408386642912460.000226916702688957230.000139395042748999980.00.00017002542202327540.000108388404819600480.000139771301404728340.00.00.01.20431560911e-050.00.03.6129468273200156e-050.00.00.00.00.03.6351829983e-050.00.00.00.00.00.000109055489949021253.73276191536e-050.000274062648290.03.8163246288578975e-057.381961117236481e-050.00025868742254986360.00032203532104725880.000241465201271952646.61971200473e-055.41620433107e-050.000127519066517456563.6129468273200156e-053.4942825351182085e-057.561276582056871e-054.1063220998003205e-054.5810143113381955e-050.0001368404467540.00.00012712056943458950.000144066254739385630.000139334516087838580.00.00.00.00.00.00.00.00.00.00.02.5979273074000003e-050.04.27745344049092e-050.03.516328481712015e-050.00.00.00.000139395042748999981.52700477045e-050.00017002542202327540.000108388404819600480.000139771301404728340.00.04.5810143113381955e-050.000417789580607000040.00080391505615699990.00051007626606982630.00028903574618560120.00045425672956536710.0006427085094748340.00094445408295407370.00082458257604087540.00.00.00.00.00.00.00.03.78595497101e-051.52700477045e-054.250635550581885e-053.6129468273200156e-053.4942825351182085e-050.00.04.5810143113381955e-050.000248646366845999970.09.7200913307213e-050.00028916524248765570.000359572944742809160.00.00.00.000248646366845999970.09.7200913307213e-050.00028916524248765570.000359572944742809160.00.00.00.000248646366845999970.09.7200913307213e-050.00028916524248765570.000359572944742809160.00.00.00.00.0001637516316320.00.00.09.845090733372009e-050.000213863959156947320.000178940028406149140.00.0001637516316320.00.00.09.845090733372009e-050.000213863959156947320.000178940028406149140.00.0001637516316320.00.00.09.845090733372009e-050.000213863959156947320.000178940028406149145.22586089406e-060.00.00.01.5677582682175932e-050.00.00.05.22586089406e-060.00.00.01.5677582682175932e-050.00.00.05.22586089406e-060.00.00.01.5677582682175932e-050.00.00.00.03.93368393344e-050.00.00.00.00.000118010518003270440.00.03.93368393344e-050.00.00.00.00.000118010518003270440.00.03.93368393344e-050.00.00.00.00.000118010518003270440.00.00.00.00.00.00.00.00.00.00.00.00.00.00.00.00.00.00.00.00.00.00.00.00.00.00.0001184299279980.00.00.00.00.000167938044850807950.000187351739143190350.00.0001184299279980.00.00.00.00.000167938044850807950.000187351739143190350.00.0001184299279980.00.00.00.00.000167938044850807950.000187351739143190350.00.000114017347909999990.00.00.07.585054181371515e-058.238470124756618e-050.000183816800668790520.00.000114017347909999990.00.00.07.585054181371515e-058.238470124756618e-050.000183816800668790520.00.000114017347909999990.00.00.07.585054181371515e-058.238470124756618e-050.000183816800668790520.00.00.00.00.00.00.00.00.00059356453185699990.00038584068736640.00083269660754422180.000456950235296729770.00049104675273247080.00042503034884161090.0003462335021593750.000386258211096973940.00059356453185699990.00038584068736640.00083269660754422180.000456950235296729770.00049104675273247080.00042503034884161090.0003462335021593750.000386258211096973940.0003917798998851.92351945644e-050.00047786880727246570.000304633490197819840.00039283740218597660.05.770558369322918e-050.00.000201784631971999980.0003666054928020.00035482780027175610.000152316745098909929.820935054649417e-050.00042503034884161090.00028852791846614580.000386258211096973940.00.0001841335152770.00.00.00.000167503279838620940.00018193288192170860.000202964384071789540.00.0001841335152770.00.00.00.000167503279838620940.00018193288192170860.000202964384071789540.00.0001841335152770.00.00.00.000167503279838620940.00018193288192170860.000202964384071789540.000154117679435000026.53671335956e-050.00.00028110488729636220.00018124815100857050.000196101400786678240.00.00.000154117679435000026.53671335956e-050.00.00028110488729636220.00018124815100857050.000196101400786678240.00.00.000154117679435000026.53671335956e-050.00.00028110488729636220.00018124815100857050.000196101400786678240.00.00.08.60148071973e-050.00.00.07.537647592737943e-050.00.00018266794566461060.08.60148071973e-050.00.00.07.537647592737943e-050.00.00018266794566461060.08.60148071973e-050.00.00.07.537647592737943e-050.00.00018266794566461063.95275222944e-050.00.00.00.000118582566883266870.00.00.09.97473045819e-050.00.00.00.00029924191374569360.00.00.09.97473045819e-050.00.00.00.00029924191374569360.00.00.09.97473045819e-050.00.00.00.00029924191374569360.00.00.00.09.74229043545e-050.00.00.00.00.00.00029226871306337690.09.74229043545e-050.00.00.00.00.00.00029226871306337690.09.74229043545e-050.00.00.00.00.00.00029226871306337690.00.000158299857910.00.00.00.000144002819682157710.00015640797012970770.0001744887839184340.00.000158299857910.00.00.00.000144002819682157710.00015640797012970770.0001744887839184340.00.000158299857910.00.00.00.000144002819682157710.00015640797012970770.0001744887839184340.00316511913616274050.0001528367925120.0041285031585514230.00231908648025542150.00304776776968143430.000128642518916060880.000193475792524298140.00013639206609624260.00316511913616274050.0001528367925120.0041285031585514230.00231908648025542150.00304776776968143430.000128642518916060880.000193475792524298140.00013639206609624260.00.00.00.00.00.00.00.00.000450988522338000040.00.00063785390234492550.000312245726584086840.00040286593808528750.00.00.00.00.00.00.00.00.00.00.00.00.00.00.00.00.00.00.00.00.00.00.00.00.00.00.00.00.00.00.00.00.00.00.01.19009097752e-050.01.9594692783751755e-050.01.6108036541946656e-050.00.00.08.71997406934e-050.00.00018448336607287373.920170196990085e-053.7914154037507105e-050.00.00.00.00.00.00.00.00.00.00.05.95954092573e-050.05.116802794851402e-054.349169954396547e-058.41265002794497e-050.00.00.04.68951215117e-050.03.25950178037409e-052.7705048988343397e-058.03852977429838e-050.00.00.03.53112692874e-060.01.0593380786215793e-050.00.00.00.00.00.000203757157707000010.00.00027119054812712430.000172879505687262760.000167201419305406330.00.00.00.000152319332077000030.00.00018651593521029529.850684084744318e-050.000171935220172493120.00.00.00.00.00.00.00.00.00.00.00.00.00.00.00.00.00.00.00.0001909064146680.00.00027957788466713840.000178226294522951330.000114915064814712230.00.00.00.00.00.00.00.00.00.00.05.67960659618e-050.09.351398211280148e-050.07.687421577260059e-050.00.00.00.00.00.00.00.00.00.00.05.4103164687e-050.06.768482898347527e-055.753061753319893e-053.709404754418331e-050.00.00.03.94108495937e-050.02.7392984659305482e-052.3283435109395654e-056.755612901228538e-050.00.00.00.00.00.00.00.00.00.00.00.000172387674809000020.00.000187940566471008280.000141126127091643050.000188096330863854630.00.00.00.00.00.00.00.00.00.00.03.1541486380900003e-060.00.00.09.462445914284454e-060.00.00.00.0001223318945450.00.000137658023334639789.167596484765096e-050.00013766169545356670.00.00.00.000412952018039000050.00.000431044474853384640.00037936170624948420.000428449873015624870.00.00.00.00.00.00.00.00.00.00.04.38538929768e-050.03.7354069989962016e-056.350027757107908e-053.0707331369220626e-050.00.00.03.03065831621e-060.00.00.09.09197494863547e-060.00.00.00.00.00.00.00.00.00.00.01.88322481519e-050.02.6297531644487022e-050.03.019921281117234e-050.00.00.07.09950824523e-050.00.000116892477641001830.09.609276971575076e-050.00.00.02.95414540443e-050.08.862436213304715e-050.00.00.00.00.00.00.00.00.00.00.00.00.03.0907214747e-060.00.09.272164424095616e-060.00.00.00.00.0001441118352511.2095260848000001e-050.00015024407098455640.000127704159325771190.000154387275443588450.03.628578254394189e-050.00.0004169886168550.00.00072762093509686220.0002166650296082220.00030667988586046250.00.00.00.00.00.00.00.00.00.00.00.00.00.00.00.00.00.00.00.000122923118142999980.00.000138010457062149780.000117305856276344550.000113453041089334230.00.00.00.00.00.00.00.00.00.00.00.00.00.00.00.00.00.00.00.000191826322030.000140741531664000020.000198873068626557820.000153670671722011360.00022293522574054170.000128642518916060880.000157190009980356250.00013639206609624260.00.00.00.00.00.00.00.05.16956352376e-050.02.577856921360497e-056.573365235257139e-056.357468414654231e-050.00.00.00.00.00.00.00.00.00.00.00.00.00.00.00.00.00.00.00.00.00.00.00.00.00.00.00.00240040289676530030.00087320320519669990.00244947869331640440.00252574975586092230.0022259802411189180.00102472059629591650.00105657419064886820.00053831482864364310.00240040289676530030.00087320320519669990.00244947869331640440.00252574975586092230.0022259802411189180.00102472059629591650.00105657419064886820.00053831482864364310.0004002364049930.00.000432176172314142330.000413257782519353230.000355275260144289630.00.00.06.77813132877e-050.0001256596910130.000111601048611985330.09.174289125125176e-050.000148891804300996420.000107812078175827320.000120275190561060470.00.00.00.00.00.00.00.03.22837545634e-050.00.09.685126369034327e-050.00.00.00.00.0008443435796270.00.00096853767188258670.00088203829432276930.00068245477267512770.00.00.00.00.00.00.00.00.00.00.00.0001294357004230.000196568600744000030.00011069001964372420.000141126127091643050.00013649095453502550.000196901814667440170.000213863959156947320.000178940028406149140.00.00.00.00.00.00.00.00.000177726528934000020.00.00015178575454876360.00021502426080505320.000166369571448165460.00.00.02.16022505563e-050.000195034136795999970.00.06.480675166876213e-050.000280470608101876960.00030463180228751210.00.0001118258921990.00.000125551179688483470.000106715744251396770.00010321075265765820.00.00.04.99057700724e-050.05.603110498494304e-054.762520817830929e-054.606099705383094e-050.00.00.00.00.0001073490939270.00.00.09.76537339949855e-050.000106066133589983990.000118327414195699180.000158520540349999980.00.000161422945313764460.000137205956894652980.000176932718841699770.00.00.00.0002234645572750.00.000216952438501699430.000230506007583016980.00022293522574054170.00.00.00.03.423048115199999e-050.00.00.04.922545366686005e-055.346598978923682e-050.02.79367450803e-050.00.00.08.381023524080516e-050.00.00.01.58785773319e-055.30194299076e-050.00.04.7635731995842264e-050.000103078941439151365.597934828360266e-050.00.00.00.00.00.00.00.00.03.45521043878e-050.000109567050412999985.603110498494304e-054.762520817830929e-050.09.967137312711329e-050.000108257582631099360.000120772195480734285.22822353139e-050.05.869925284136888e-054.989307523441927e-054.825437786591812e-050.00.00.05.26269423706e-050.00.00.000157880827111655480.00.00.00.00.01.62756223325e-050.00.00.04.882686699749275e-050.00.00.00.00.00.00.00.00.00.00.03.54990989116e-050.00.00.00.00.000106497296734658720.00.00170823894396439990.00172643621791930.00173521334385186670.00175022564665988340.00163927784138156490.00203199974049297730.00109776450786658080.0020495444053997670.00170823894396439990.00172643621791930.00173521334385186670.00175022564665988340.00163927784138156490.00203199974049297730.00109776450786658080.0020495444053997670.00.00.00.00.00.00.00.00.0001195669474250.0003455206437260.000228960125060423880.000129740717213705650.00.00054305003294772420.00.00049351189823084290.00.0001021979762510.00.00.00.000138623404004375950.00.000167970524749067184.4077358969e-050.04.9487326300570116e-054.2063140069893625e-054.0681610536595204e-050.00.00.00.00.00.00.00.00.00.00.00.00.00.00.00.00.00.00.00.00.00.00.00.00.00.00.08.49140886374e-050.00.00.000129497757069110670.000125244508843000960.00.00.00.0004483255983860.0008082635202760.00052731495469163050.00038417667930502840.00043348516116216450.00080401574322538070.00072773152768683450.00089304328991587380.05.41238357525e-050.00.00.00.00.00.000162371507257431640.0001148219312650.07.929548190851587e-050.00013479883484386960.000130371477041252480.00.00.00.000209431150707999970.00.000235136313405021060.000199860700216488710.00019329643850335990.00.00.02.2466472474e-050.00.06.73994174219348e-050.00.00.00.00.04.51694237767e-050.00.00.00.000135508271330120320.00.00.000197006174897999980.00.000221186320787873840.000188003512987073050.000181828690919012680.00.00.00.0002175806389880.00.000160467779956878260.000212720579904386920.000279553557102446570.00.00.00.000117414022669999990.00.000156757542270014046.662023340549625e-050.000128864292335573250.00.00.00.05.16931624061e-050.00.00.07.011765202546924e-050.08.496183519284212e-050.0001326345595440.0003194676557317.660749947093906e-050.000195344074222895750.000125952104938159160.000340684636959906970.00037003298017974640.000247685350053709267.81820963029e-050.06.712637329879314e-055.705594247104382e-050.000110363973138882030.00.00.07.81820963029e-050.06.712637329879314e-055.705594247104382e-050.000110363973138882030.00.00.07.81820963029e-050.06.712637329879314e-055.705594247104382e-050.000110363973138882030.00.00.00.07.66180371364e-050.00.00.03.45565505684261e-050.00.000195297560840923140.07.66180371364e-050.00.00.03.45565505684261e-050.00.000195297560840923140.00.00.00.00.00.00.00.00.00.00.00.00.00.00.00.00.03.94337224973e-050.00.00.03.45565505684261e-050.08.37446169236037e-050.00.00.00.00.00.00.00.00.03.71843146391e-050.00.00.00.00.00.000111552943917319430.00.000131038560660.00.00.00.000188441189818448550.000204674492161922180.00.00.000131038560660.00.00.00.000188441189818448550.000204674492161922180.00.00.00.00.00.00.00.00.00.00.000131038560660.00.00.00.000188441189818448550.000204674492161922180.02.84111048448e-050.00.04.332819691410094e-054.190511762040258e-050.00.00.02.84111048448e-050.00.04.332819691410094e-054.190511762040258e-050.00.00.02.84111048448e-050.00.04.332819691410094e-054.190511762040258e-050.00.00.00.0002756624674960.000357379813328999970.00031171327370933820.00026193864498070120.000253335483796070140.00027724680800875190.000458951582479690430.000335941049498134350.0002756624674960.000357379813328999970.00031171327370933820.00026193864498070120.000253335483796070140.00027724680800875190.000458951582479690430.000335941049498134350.0001717580429260.00.00.00026193864498070120.000253335483796070140.00.00.00.000103904424569999990.0003047727149420.00031171327370933820.00.00.00027724680800875190.000301130287318690150.000335941049498134350.05.2607098387e-050.00.00.00.00.000157821295161000260.00.173003323093740420.18020047761844050.177642395169037130.169233328215481650.172134245896663370.183877678189084880.18627765375986650.170446100906367860.001206314407764870.00.00069709964490404210.00158175902406935440.00134008455432135540.00.00.00.001206314407764870.00.00069709964490404210.00158175902406935440.00134008455432135540.00.00.09.83469038128e-050.07.876161133725064e-050.000105363307096527570.000110915793004576440.00.00.00.00.00.00.00.00.00.00.00.0005507166392710.00.00024720771053765070.00074125288029740590.00066368932697841180.00.00.06.98654380583e-050.08.268004516070863e-057.027622182409055e-055.66400471901783e-050.00.00.08.955167349769999e-060.00.02.686550204930268e-050.00.00.00.00.0004784302592730.00.000288450277868432140.00063800111280202770.00050883938714818890.00.00.00.000613051911432320.00.00053649245698576840.00098753076091996150.000315132516391618140.00.00.00.000613051911432320.00.00053649245698576840.00098753076091996150.000315132516391618140.00.00.01.40295457413e-050.02.275088490999365e-051.9337752314011494e-050.00.00.00.06.46762086372e-060.00.01.9402862591163047e-050.00.00.00.00.0001629423256950.00.000112527198392997630.00019129129260001410.000185008486091735830.00.00.01.29688903863e-050.00.00.03.890667115890781e-050.00.00.00.0004166435287460.00.000401214373682777160.00075749885341477299.121735914097452e-050.00.00.00.0013551633397130.00.0009770282548961970.00141102210372353160.00167743966051950150.00.00.00.0013551633397130.00.0009770282548961970.00141102210372353160.00167743966051950150.00.00.00.0001941299330930.07.575154975617997e-050.00025754861182460.00024908963769892930.00.00.00.0001726675487750.07.575154975617997e-050.000193161458868450.00024908963769892930.00.00.00.0001771605955520.00.00015150309951235990.000193161458868450.000186817228274196950.00.00.00.000346566307661999960.00.000152354240520856320.00032374439267277670.00056360028979350430.00.00.00.000464638954631000030.00.0005216678153506210.00044340618148925480.00042884286705394170.00.00.00.00266629137950999970.00.00347170270926269640.00242744214723960260.0020997292820252940.00.00.00.00266629137950999970.00.00347170270926269640.00242744214723960260.0020997292820252940.00.00.00.0004243600279130.00.000431221719636770.000447979873394556050.00039387849070767090.00.00.00.0006280634230550.00.00079213733989022560.00059408764840983760.0004979652808637530.00.00.00.0001542853825580.00.00014374057321225670.00012217632910760620.000196939245353835450.00.00.00.0004305910102940.00.00059168846864099830.00033528146557529750.0003648030966663410.00.00.00.0001029270570150.09.970240739967804e-050.000127117283593575578.196148005166974e-050.00.00.00.0009260644786750.00.0014132122004827680.00080079954715872940.00056418168838202380.00.00.08.98073369831e-050.00.05.5185856018663716e-050.00021423615493066890.00.00.08.98073369831e-050.00.05.5185856018663716e-050.00021423615493066890.00.00.03.88053045935e-050.00.03.967401163218882e-057.674190214820713e-050.00.00.05.10020323896e-050.00.01.55118443864749e-050.00013749425278246180.00.00.00.00093987147681320.00.00106994507069633470.00088944116473342080.00086022819501293010.00.00.00.00093987147681320.00.00106994507069633470.00088944116473342080.00086022819501293010.00.00.00.000141255757166999990.09.755055688026056e-050.000165831660131666930.000160385054489598380.00.00.00.000148187846662999970.00.000242134417970646670.000102904467670989729.952465434845612e-050.00.00.00.0001345969207120.00.00013038007121496360.000166230293930060340.000107180396990645080.00.00.00.000243983764797999970.00.000273929846593054840.00023283435109395660.000225187096707617920.00.00.02.17300118692e-050.06.51900356074818e-050.00.00.00.00.00.0002501171756040.00.00026076014242992720.000221640391906747180.000267950992476612650.00.00.00.00118997965585859970.00.0011722850199041380.00093343672716249930.00146421722050770250.00.00.00.00118997965585859970.00.0011722850199041380.00093343672716249930.00146421722050770250.00.00.00.000308984781390999970.00.00037047889088404960.00025191913397051040.000304556319317679870.00.00.00.0003378206008160.00.00035219551704821340.000299358451406515630.00036190783399438590.00.00.08.14593917396e-050.00.06.263750206060246e-050.000181740673158050330.00.00.00.000245296422830999970.00.00022853136078128450.0001942466356036660.00031311127210750240.00.00.00.000216418459080999950.00.000221079251190590440.000125275004121204920.00030290112193008390.00.00.00.0001720369124120.00.000167401572917977920.000142287659001862360.00020642150531531640.00.00.00.0001720369124120.00.000167401572917977920.000142287659001862360.00020642150531531640.00.00.00.0001720369124120.00.000167401572917977920.000142287659001862360.00020642150531531640.00.00.00.00049219050344219990.00.00045657753969794020.00038808087728232390.0006319130933481890.00.00.00.00049219050344219990.00.00045657753969794020.00038808087728232390.0006319130933481890.00.00.07.40932291872e-050.08.318728470157186e-057.070736428926902e-056.838503857071832e-050.00.00.00.000212747937485999960.00.000207015685593224640.000175958784414516830.0002552693424510020.00.00.05.7162878395000006e-050.00.00.00.000171488635185032120.00.00.00.000148186458373999960.00.000166374569403143720.000141414728578538050.000136770077141436640.00.00.03.91114431124e-050.00.00.00.000117334329337127220.00.00.03.91114431124e-050.00.00.00.000117334329337127220.00.00.03.91114431124e-050.00.00.00.000117334329337127220.00.00.00.0017122117767010.00.00212476233154374350.0011681047681571810.0018437682303998450.00.00.00.0017122117767010.00.00212476233154374350.0011681047681571810.0018437682303998450.00.00.00.000193744832322999970.00.000274855285263977270.000155747302420957440.000150631909284149820.00.00.00.0005184504136430.00.00064132899894928030.00031149460484191490.00060252763713659920.00.00.00.0002188501505370.00.000274855285263977270.000155747302420957440.000225947863926224750.00.00.00.000138023959180999980.00.00022725464926853990.00.000186817228274196950.00.00.00.00059088990692699990.00.00054971057052795470.00054511555847335120.00067784359177867430.00.00.05.225251409e-050.00.000156757542270014040.00.00.00.00.03.79141540375e-050.00.00.00.000113742462112521290.00.00.03.79141540375e-050.00.00.00.000113742462112521290.00.00.03.79141540375e-050.00.00.00.000113742462112521290.00.00.00.05.56227919251e-050.00.00.00.00.00016686837577532510.00.05.56227919251e-050.00.00.00.00.00016686837577532510.00.05.56227919251e-050.00.00.00.00.00016686837577532510.00.00.0001447898967850.00.00.00.000165208714361379560.00026916097599376070.00.00.0001447898967850.00.00.00.000165208714361379560.00026916097599376070.00.00.0001447898967850.00.00.00.000165208714361379560.00026916097599376070.00.00.0001148593918890.00.00.00.00034457817566802030.00.00.00.0001148593918890.00.00.00.00034457817566802030.00.00.00.0001148593918890.00.00.00.00034457817566802030.00.00.00.000287948361630.00.00.00.000221288736667535990.000240351697217670130.00040220465100464720.00.000287948361630.00.00.00.000221288736667535990.000240351697217670130.00040220465100464720.00.000287948361630.00.00.00.000221288736667535990.000240351697217670130.00040220465100464725.72526809752e-053.30787058039e-050.08.731288166023372e-058.444516126535672e-050.09.923611741184108e-050.05.72526809752e-053.30787058039e-050.08.731288166023372e-058.444516126535672e-050.09.923611741184108e-050.05.72526809752e-053.30787058039e-050.08.731288166023372e-058.444516126535672e-050.09.923611741184108e-050.08.862436213299999e-050.00.000265873086399141440.00.00.00.00.08.862436213299999e-050.00.000265873086399141440.00.00.00.00.08.862436213299999e-050.00.000265873086399141440.00.00.00.00.00.00.00.00.00.00.00.00.00.00.00.00.00.00.00.00.00.00.00.00.00.00.00.00.00.00045177354049340.00105460481968510.0003160363211997010.00049727232717813220.00054201197310278970.00090878335381682630.00105569811405350570.00119933299118597120.00045177354049340.00105460481968510.0003160363211997010.00049727232717813220.00054201197310278970.00090878335381682630.00105569811405350570.00119933299118597120.000138296499066000020.0003202440554674.445746690608595e-050.000151151480382306260.000219280549908729580.000276792305044803150.00030063663110997090.00038330323024705172.91789725433e-050.000140224490330.04.44992292631307e-054.303768836689995e-054.656461833351625e-050.00020350317446752570.00017060567818874920.00.0001047516722380.00.00.00.00014767636100058010.000106931979578473645.9646676135383045e-050.000144669154748999980.000210673199294999970.00014685408743346080.000166430330384849840.000120723046429896240.000174155034633656460.000191486298760393670.00026637826449067490.00.00.00.00.00.00.00.05.91142717517e-050.0001903273150223.4327917484446115e-055.8355951286839746e-058.465894648375002e-050.000183193460481732310.000165812246814721780.000221976237769653368.05146423834e-058.83840873331e-059.039684937570812e-057.683533586100568e-057.431174191351391e-058.040157432253806e-058.732778332242014e-059.742290435445898e-050.00077159919670480.0014317746088340.00080440130891266620.00078394344237897050.00072645283881930410.00134878209391922160.00131221715575522870.00163432457682690840.00077159919670480.0014317746088340.00080440130891266620.00078394344237897050.00072645283881930410.00134878209391922160.00131221715575522870.00163432457682690840.0003042360966420.0006211657918210.00031718192763406340.00026959766968773920.00032592869260313120.00063474927096740570.0004596199122232640.00076912819227204457.10423315148e-050.0001816565609777.976192591974243e-056.77958845832403e-056.55691840413358e-050.00014188513115742010.000231161779382876860.000171922772390221740.000152875903260.000167817887340999980.000171639587422230560.000145889878217099370.000141098244139583340.000152661217068110240.000165812246814721780.00018498019814137780.0002434448652880.0004611343686950.000235817867936629830.00030066000989089170.00019385671803525370.00041948647472628550.000455623217334365940.00050829341402326440.05.14519656129e-050.00.00.02.0615788287830277e-050.000108759876665285152.4980231885758714e-050.05.14519656129e-050.00.00.02.0615788287830277e-050.000108759876665285152.4980231885758714e-050.02.17997556382e-050.00.00.02.0615788287830277e-054.478347862688212e-050.00.01.38615529083e-050.00.00.00.04.158465872496197e-050.00.01.57906570664e-050.00.00.00.02.239173931344106e-052.4980231885758714e-051.2146651275e-050.01.9999302959227454e-050.01.6440650865821666e-050.00.00.01.2146651275e-050.01.9999302959227454e-050.01.6440650865821666e-050.00.00.01.2146651275e-050.01.9999302959227454e-050.01.6440650865821666e-050.00.00.00.03.67827538695e-050.00.00.05.289577258061714e-055.745248902790798e-050.00.03.67827538695e-050.00.00.05.289577258061714e-055.745248902790798e-050.00.03.67827538695e-050.00.00.05.289577258061714e-055.745248902790798e-050.00.00.00019531839740430.00.00.00.000185896522031255120.000202419042587877820.000197639627592381650.00.00019531839740430.00.00.00.000185896522031255120.000202419042587877820.000197639627592381650.02.29614639551e-050.00.00.00.00.06.888439186542436e-050.01.85952724662e-050.00.00.02.67411001072743e-052.9044717291270558e-050.00.00.000153761660982999980.00.00.00.000159155421923980820.000173374325296607260.00012875523572695730.00.00.00.00.00.00.00.04.24570443187e-050.000277556651083439970.06.474887853455534e-056.262225442150048e-050.000242675227591015180.00032570679442369650.000264287931237084774.24570443187e-050.000277556651083439970.06.474887853455534e-056.262225442150048e-050.000242675227591015180.00032570679442369650.000264287931237084770.00.00.00.00.00.00.00.00.04.4554991491e-050.00.00.00.00.000133664974473092040.04.24570443187e-050.000148961944942999990.06.474887853455534e-056.262225442150048e-050.000135508271330120320.000147181657284977750.000164195906215380330.07.58521816227e-050.00.00.08.260435718068978e-054.4860162665626785e-050.000100092025021704420.08.18753302674e-060.00.00.02.4562599080205107e-050.00.07.91188860215e-059.0941516732e-050.09.685126369034327e-050.000140505394374290980.000101346522255300080.000110077037801369936.14009901393649e-057.91188860215e-059.0941516732e-050.09.685126369034327e-050.000140505394374290980.000101346522255300080.000110077037801369936.14009901393649e-050.00.00.00.00.00.00.00.07.91188860215e-059.0941516732e-050.09.685126369034327e-050.000140505394374290980.000101346522255300080.000110077037801369936.14009901393649e-050.00.000326355375076999960.00.00.00.00052085497289777290.00025819805760056360.000200013094731318950.00.000326355375076999960.00.00.00.00052085497289777290.00025819805760056360.000200013094731318950.00.00.00.00.00.00.00.00.00.000224764470095999960.00.00.00.0004284393702281890.000157821295161000268.803274489860752e-050.00.0001015909049810.00.00.09.241560266958396e-050.000100376762439563350.000111980349832711450.00.00.00.00.00.00.00.00.00.00.00.00.00.00.00.00.00.00.00.00.00.00.00.00.00029602834087180.00082387046129840.00020723507972677390.00061370077850884196.714916437968124e-050.00117352488741727550.00089867867240102720.000399407824077015450.00029602834087180.00082387046129840.00020723507972677390.00061370077850884196.714916437968124e-050.00117352488741727550.00089867867240102720.000399407824077015457.742230798e-050.000240837686559000040.000125551179688483470.000106715744251396770.00.000223337706451494570.00036386576384341720.000135309589381192983.28356136158e-053.43596471464e-050.09.850684084744318e-050.00.000103078941439151360.00.04.3165919023000006e-050.000167795483944000020.00.000129497757069110670.00.000241299816491354580.00026208663534079370.07.27541949248e-050.0001890745266128.168390003829045e-056.942952035633043e-056.714916437968124e-050.000145304049980490470.000157821295161000260.00026409823469582250.07.356550773899999e-050.00.00.00.000105791545161234270.000114904978055815970.06.98503053282e-050.000118237609297999990.00.00020955091598456090.00.000354712827893550240.00.04.37127893609e-050.000347362268386400060.00.00.00013113836808267160.000425655393472260240.00061643141168767160.04.37127893609e-050.000347362268386400060.00.00.00013113836808267160.000425655393472260240.00061643141168767160.04.37127893609e-059.86643280264e-050.00.00.00013113836808267160.00014188513115742010.00015410785292191790.00.00.000248697940360.00.00.00.00028377026231484020.00046232355876575370.00.0007343519074090.00162655522800090.00055078286882961560.00077165952624607240.00088061332715067680.00187775985963498830.00184951527329153130.00115239055107748630.0007343519074090.00162655522800090.00055078286882961560.00077165952624607240.00088061332715067680.00187775985963498830.00184951527329153130.00115239055107748630.00.0002576676307410.00.00.00.00028714847972335020.00031188494043721480.000173969472061533880.06.00011748676e-050.00.00.00.000180003524602697140.00.00.0002813269559270.00057415121844000010.00016336780007658090.000277718081425321660.000402894986278087460.00058121619992196190.00078910647580500120.000352130979594430130.04.67451013503e-050.00.00.00.000140235304050938480.00.00.000453024951481999960.0006879901026020.00038741506875303470.00049394144482075080.000477718340872589350.00068915635133604060.00074852385704931540.00062629009942152220.0005348970464260.00099047213163290.00068482461648263710.00058208587773489150.000337780645061426840.00132245596460002150.00105851858572630470.00059044184457247870.0005348970464260.00099047213163290.00068482461648263710.00058208587773489150.000337780645061426840.00132245596460002150.00105851858572630470.00059044184457247870.00.000115963809118999990.00.00.00.000347891427357135870.00.00.0005348970464260.0007932946110770.00068482461648263710.00058208587773489150.000337780645061426840.00073092340293216430.00105851858572630470.00059044184457247870.08.12137114369e-050.00.00.00.000243641134310721380.00.00.000513118706628740.000382570389442639970.00036732519455069440.00072561926080851550.00044641166452728520.00032070844477766160.000410808619880116940.000416194103670740660.000513118706628740.000382570389442639970.00036732519455069440.00072561926080851550.00044641166452728520.00032070844477766160.000410808619880116940.000416194103670740660.00.00.00.00.00.00.00.02.56373446531e-050.02.848640211419373e-054.8425631845171634e-050.00.00.00.02.39751985361e-050.02.6639543038027923e-054.528605257033733e-050.00.00.00.00.00.00.00.00.00.00.00.00.00.00.00.00.00.00.00.00.00.00.00.00.00.00.00.01.49650572054e-051.66068670936e-050.02.282237698841753e-052.207279462777641e-052.388165573936774e-052.5938945541312908e-050.00.09.6458321143e-060.00.00.00.00.02.893749634290861e-053.2991550867e-050.09.897465260114023e-050.00.00.00.00.00.00.00.00.00.00.00.00.01.73118204179e-050.02.807355570674165e-052.386190554689617e-050.00.00.00.07.708686920489999e-068.34041227412e-060.00.02.3126060761466986e-052.502123682236661e-050.00.04.2826937438e-050.04.808343051899367e-054.086985950053493e-053.952752229442229e-050.00.00.05.73398028813e-050.0001302302017990.00.000172019408644042550.00.000135002643452022840.000146632471996600960.000109055489949021253.93612181704e-054.367952021999999e-050.06.0027606141410684e-055.805604836993274e-056.281372993948285e-056.822483072064073e-050.06.14631781875e-050.05.235338766932902e-058.89984585262614e-054.303768836689995e-050.00.00.00.09.58886853882e-060.00.00.00.00.02.8766605616474103e-050.00.00.00.00.00.00.00.00.00.00.00.00.00.00.00.06.29474673482e-058.23672706575e-052.772909490052396e-054.713824285951269e-050.000113975064284530547.398917882442152e-055.35753271916688e-050.000119537305956391415.00561130902e-050.02.8911572295002588e-057.372260370458968e-054.753416327090442e-050.00.00.00.00.00.00.00.00.00.00.09.35785190225e-060.02.807355570674165e-050.00.00.00.00.06.71764790109e-058.21114167453e-050.00.00010244711448134099.908232255135188e-050.00.000116437044429893530.000129897205805945280.00107269515928726980.001098243206995460.00084250611674765970.0012411863060946690.00113439305501883980.00092948171901303330.0012100825184829110.00115516538349001280.00107269515928726980.001098243206995460.00084250611674765970.0012411863060946690.00113439305501883980.00092948171901303330.0012100825184829110.00115516538349001281.05833795952e-050.00.03.175013878553954e-050.00.00.00.05.75168281064e-068.77683823013e-060.00.01.7255048431930473e-050.00.02.6330514690394322e-050.000306343400565999960.00.000294772334920787230.000349906045993710230.000274351820782650660.00.00.00.00.00.00.00.00.00.00.05.8832569572e-060.00.01.7649770871593955e-050.00.00.00.01.88118323326e-050.02.11207591999318e-051.7952181275935903e-051.7362556521849045e-050.00.00.04.19623634691e-050.02.1022523110629788e-053.573736551674682e-056.912720178001294e-050.00.00.04.28110288951e-059.43424274687e-054.165753427451986e-053.540798887603948e-055.136756353468703e-059.262854184624201e-050.000100608045302327348.979069525756588e-050.0003647910591880.00051350118336400010.00030693919791814960.00040221795793606790.000385216021710801630.00048425984547064160.00054625385156054510.00050998985306105830.00.00.00.00.00.00.00.05.91041045085e-066.71752179403e-060.01.773123135253977e-050.00.02.0152565382096954e-050.01.2940674467000001e-051.58176958627e-050.01.9735103388956935e-051.9086920012032682e-050.02.2430081332813392e-052.5023006255426106e-055.993395933e-060.00.01.798018779898729e-050.00.00.00.02.92565913045e-050.00.00.08.776977391359909e-050.00.00.00.00.000186726945070.00.00.00.000169862480963108570.000184495316878352430.000205823037368575331.86601330586e-050.00.02.845753180037247e-052.752286737537552e-050.00.00.02.90022898763e-050.02.0028844027114054e-053.404815473899809e-053.2929870862709266e-050.00.00.06.5004514264800005e-060.00.01.9501354279443068e-050.00.00.00.00.00.00.00.00.00.00.00.02.69905496026e-054.85854412499e-050.04.116178706839589e-053.980986173938245e-050.09.356548213116442e-055.2190841618460165e-050.0001284595032630.000223775153955999970.000136964923296527420.000135820038138141340.000112593548353808960.000182730850733041040.000242577175895611480.000246017435238532841.20431560911e-050.00.03.6129468273200156e-050.00.00.00.00.00010888258639328.059772458640001e-050.000225619711261122630.00.00010102804791867450.00.000141701148737456040.000100092025021704420.00010888258639328.059772458640001e-050.000225619711261122630.00.00010102804791867450.00.000141701148737456040.000100092025021704420.01.73269411354e-050.00.00.00.05.198082340620246e-050.07.46413555691e-050.00.000122896018788727050.00.00010102804791867450.00.00.00.06.327078345100001e-050.00.00.00.08.972032533125357e-050.000100092025021704420.00.00.00.00.00.00.00.00.00.00.00.00.00.00.00.00.00.00.00.00.00.00.00.03.42412308241e-050.00.000102723692472395560.00.00.00.00.00.00.00.00.00.00.00.00.00.00.00.00.00.00.00.00.00.00.00.00.00.00.00.00.00.00.00.00.00.00.00.00.00.0082950532941088990.0060790869933966980.0104421704117974530.0062678512725158040.0081751381980083880.0057411103814086960.0063932914536047780.0061028591451775940.0082950532941088990.0060790869933966980.0104421704117974530.0062678512725158040.0081751381980083880.0057411103814086960.0063932914536047780.0061028591451775947.11626320156e-050.0002562533758750.000115400233245584799.808766280128384e-050.00.000359241076760276460.000222964553163625880.000186554497700027830.09.45156374769e-050.00.00.00.000105329573348303130.000114403209592690116.381412948982027e-050.0003705150007750.00.000471635735873259660.00030066000989089170.000339249256561693950.00.00.00.000392699103050.00102599117239999980.00065272024442422189.978615046883853e-050.00042559091425690680.00084365322475657020.001115921332339470.00111839896010467440.0009349302411460.000174303938502000020.00083939931563157520.00054882382757861190.00141656758022635885.7429695944670045e-050.000187130964262328840.000278351155298454250.00.00.00.00.00.00.00.00.000245844413020.00.000294772334920787230.000200440006593927820.00024232089754406710.00.00.03.28580603858e-050.0003384501982460.05.0110001648481956e-054.846417950881342e-050.00041948647472628550.00034171741300077450.000254146707011632140.000329031595854000070.00.00044984214144309720.000273111383392200240.000264141262725760330.00.00.00.01.89843007223e-050.00.00.00.05.695290216679574e-050.00.000346647881065999951.95802204759e-050.00039818625003675130.000441815486667768660.00019994190649375940.05.874066142763687e-050.02.4834299278999996e-050.07.450289783712206e-050.00.00.00.00.00.02.10872087347e-050.00.00.00.00.06.326162620419415e-050.01.90672015988e-050.00.00.00.05.7201604796345055e-050.00.000319017964499000030.000347351945886999940.000469594022730951170.000149679225703257820.000337780645061426840.000208835257980618360.000453650822454130570.00037956975722516490.000378050527034000050.0005462414869590.00038376020961385510.000434916995439654730.000315474376047936440.00045510325088229090.0004943082074853970.00068931300250796440.00.00.00.00.00.00.00.00.0002278716293870.00.000255840139742570070.000217458497719827370.000210316250698624240.00.00.00.0001004320678598.44120492675e-050.000165360090321417260.00.000135936113256427889.349020270062564e-050.000159745945101988030.00.0001013301826769.42758995037e-050.000114299476806019550.000144194086376244024.549698484500852e-054.922545366686005e-050.000110418891956032570.00012318335288829110.000105019098761000010.00.000117908933968314910.000100220003296963919.692835901762684e-050.00.00.08.93166913752e-050.00.000171882016218024664.883601855572394e-054.723203935180968e-050.00.00.00.000113935814693999980.000125071821698000020.000127920069871285040.000108729248859913680.000105158125349312120.000113775812720572730.000123577051871349240.000137862600501592880.000175866891099000040.000208481849273000010.000234797011365475599.978615046883853e-050.000193017511463672520.000208835257980618360.000226825411227065280.000189784878612582450.0005848530675190.00.00070301450220399930.00050607624301846890.00054546845733358180.00.00.00.0001128801261227.88206883378e-050.00019160201769851175.0110001648481956e-059.692835901762684e-055.243580934078569e-055.695290216679574e-050.00012707335350581610.0003433983306790.00.00040390081635954680.00029426298840385150.000332031187273147340.00.00.00.00.0002229888286250.00.00.00.000257697353597878440.000223917393134410650.000187351739143190350.000228149126412999972.75205944504e-050.000293496264206844470.000149679225703257820.00024127188932959060.00.08.256178335123643e-050.0005903088308250.000116577189640.00087589493805033920.0004796243014926130.000415407252932686530.000172289087834010140.000113905804333591496.353667675290805e-050.00043863066927900010.0007419427708540.00053059020285741710.00030066000989089170.00048464179508813420.00078653714011178540.00074038772816834460.00069890344428198830.00075572781147400015.60444149619e-050.00083906047199566930.00051317681244060370.00091494614998640330.000104871618681571380.06.326162620419415e-050.00.00.00.00.00.00.00.05.20568808513e-050.0001504383365615.8446238820500914e-054.9678018875650217e-054.804638485787537e-050.000155951329504922950.00016938578661676320.000125977893561800420.000176413123555000020.0003632557046140.000234797011365475590.000149679225703257820.000144763133597754370.00031325288697092750.000396944469647364330.00037956975722516490.0002342047304455.78935069868e-050.0003552714604285470.000201315290465517040.000146027440441402885.266478667415157e-055.7201604796345055e-056.381412948982027e-050.0001305479057690.000206296758140999950.000293496264206844474.989307523441927e-054.825437786591812e-050.000208835257980618360.000283531764033831650.00012652325240838830.0002885185972020.0006832398936050.000324779099553442340.000207041324176362550.00033373536787506250.00072216982924435370.00062750502986170160.00070004482170868720.000400741824773630078.526547839327e-050.000274488645975057770.000451777528276939770.00047595930007015069.771818325817776e-053.643718358376361e-050.000121641068337878570.000400741824773630078.526547839327e-050.000274488645975057770.000451777528276939770.00047595930007015069.771818325817776e-053.643718358376361e-050.000121641068337878573.58571353572e-050.01.5603598856566417e-056.631358100777245e-052.5654226207196975e-050.00.00.04.03868162573e-050.00.00.00.000121160448772033540.00.00.00.0001288466487670.00.000125373596690338520.000158101581045716840.000103064768566204660.00.00.01.75384205694e-050.00.03.9788148604663466e-051.2827113103598487e-050.00.00.00.00.00.00.00.00.00.00.09.35316075337e-060.00.01.4263985617760956e-051.3795496642360254e-050.00.00.00.00.00.00.00.00.00.00.09.21628523015e-064.90253501967e-060.01.4055244364818113e-051.3593611325642787e-051.4707605059000864e-050.00.02.75766155144e-050.01.904428006510704e-053.237443926727767e-053.131112721075024e-050.00.00.00.00.00.00.00.00.00.00.00.00.00.00.00.00.00.00.00.00.00.00.00.00.00.00.00.00.00.00.00.00.00.00.01.47282882409e-051.08429560169e-051.6536009032141722e-051.4055244364818113e-051.3593611325642787e-051.4707605059000864e-050.01.7821262991669323e-052.19649283076e-053.68779223921e-051.8858869828033678e-051.6029625005773087e-053.1006290089087863e-053.354724936962645e-053.643718358376361e-054.0649334223000956e-051.01172353372e-050.01.6657896076604685e-050.01.369380993492271e-050.00.00.05.98669419168e-050.06.241439542626565e-055.3050864806217965e-056.413556551799244e-050.00.00.00.00.00.00.00.00.00.00.00.00.00.00.00.00.00.00.08.42492717774e-060.00.02.527478153322555e-050.00.00.00.06.15667755297e-060.00.01.8470032658895595e-050.00.00.00.01.07077437916e-053.26420649646e-050.00.03.2123231374717824e-053.475572377054959e-050.06.317047112320828e-050.00.00.00.00.00.00.00.00.000383333273011099970.00059052219825369990.000436213367225653550.00025674619686331090.0004570402549426780.00081070993505993170.00073118827044409020.00022966838925883710.000383333273011099970.00059052219825369990.000436213367225653550.00025674619686331090.0004570402549426780.00081070993505993170.00073118827044409020.00022966838925883713.47680189907e-050.00.000104304056971970880.00.00.00.00.00.01.53746097399e-050.00.00.00.04.612382921958811e-050.00.00.00.00.00.00.00.00.00.00.00.00.00.00.00.00.00.01.51701083627e-050.00.00.04.551032508822909e-050.00.00.04.66817362675e-050.00.00.04.246562024077714e-054.612382921958811e-055.145575934214383e-050.00.0001300227578640.00.00.00.00018698040540125130.000203087868191674760.00.02.94262030956e-050.00.00.04.231661806449372e-054.596199122232639e-050.06.8343813813e-050.00.000112527198392997630.09.250424304586792e-050.00.00.01.6084792622000004e-053.63050557673e-050.00.04.825437786591812e-055.2208814495154575e-055.670635280676632e-050.00.03.11884940437e-050.00.00.00.09.356548213116442e-050.04.81163201494e-050.05.402202153926778e-054.591753139103925e-054.4409407518036204e-050.00.00.00.0002160203274360.0002863532331130.000165360090321417260.000210828665472271640.000271872226512855770.000441228151770025850.000239618917652982070.000178212629916693260.00105763959021873970.00.00068831583057893060.00121939342869429650.00126520951138232280.00.00.00.00105763959021873970.00.00068831583057893060.00121939342869429650.00126520951138232280.00.00.02.06699175336e-050.00.02.5302525530517784e-053.670722707034167e-050.00.00.03.97504106022e-050.04.416784171451536e-057.508339009218796e-050.00.00.00.00.00072433204931399990.00.00053036301801354740.0007393151417468310.00090331798818123050.00.00.07.3721966184e-060.00.00.02.211658985521247e-050.00.00.08.54904286055e-060.00.01.3037670112161596e-051.2609458469487656e-050.00.00.02.18119400602e-050.00.03.983776839555676e-052.5598051784962533e-050.00.00.00.00.00.00.00.00.00.00.04.2270615422900006e-060.00.00.01.2681184626879504e-050.00.00.00.00.00.00.00.00.00.00.00.000111992673490.06.918126227732762e-050.000124618680551236950.000142178077640651580.00.00.00.00.00.00.00.00.00.00.01.68498543555e-050.00.05.05495630664511e-050.00.00.00.00.00.00.00.00.00.00.00.00.00.00.00.00.00.00.00.00.0001020844438420.04.4603708573540174e-050.000151648689199353320.000110000933753556770.00.00.07.77216419998e-050.00.00.000136236566981810339.692835901762684e-050.00.00.07.77216419998e-050.00.00.000136236566981810339.692835901762684e-050.00.00.06.57161207715e-050.00.00.000100220003296963919.692835901762684e-050.00.00.01.20055212283e-050.00.03.601656368484641e-050.00.00.00.00.00166817470690060.00082211770266900010.00073320367532377390.00261379172266148170.001657528722712240.00080838525552663380.00078867374745892230.00086929410502274140.00166817470690060.00082211770266900010.00073320367532377390.00261379172266148170.001657528722712240.00080838525552663380.00078867374745892230.00086929410502274149.65268038455e-060.00.02.8958041153645354e-050.00.00.00.06.80723424706e-050.05.8446238820500914e-054.9678018875650217e-059.609276971575076e-050.00.00.03.80372949807e-050.00.00.000114111884942087650.00.00.00.00.000145833334657000030.00.00.000405190550964069173.230945300587561e-050.00.00.01.9036155890999998e-050.00.02.9030983322798107e-052.807748435019417e-050.00.00.00.00.00.00.00.00.00.00.00.0001327141895750.00.000129138356251011540.000109764765515722410.00015923944695752980.00.00.00.00.00.00.00.00.00.00.00.0001150209176910.0002595483013240.000129138356251011540.000109764765515722410.000106159631305019880.00038502062244093770.000254448703881450470.000139175577649227130.0003588235130840.00.00011894322286277380.00064519374555551890.00031233357083353550.00.00.01.56169381831e-050.00.04.685081454939371e-050.00.00.00.01.83939955231e-050.00.00.05.5181986569441016e-050.00.00.00.0001491583004350.000235215530155999980.00.000227473033799030.000220001867507113590.000158687317741851430.000258536200625585930.00028842307210201679.93472485475e-063.67977784801e-050.00.02.980417456424354e-053.2246620717595476e-050.07.814671472282806e-057.64625098252e-054.85626853479e-050.00.000161831400463324266.755612901228538e-054.417668918820772e-054.7982298528802273e-055.352906832662581e-054.86299594057e-050.00.00.000145889878217099370.00.00.00.00.0001801111236520.00.000110240060214278150.000218637134563837270.00021145617617666560.00.00.07.11798294191e-050.0002064426011550.00.00.000213539488257223830.000138623404004375950.000200753524879126760.000279950874581778646.83222103546e-050.09.00965276169848e-050.000114870103446686210.00.00.00.01.52904818752e-053.5550806206e-050.00.04.587144562562587e-054.963060143366548e-052.6953019543956837e-053.0068797640265118e-050.0001278842046390.09.7200913307213e-050.000206546601776896927.990509883173539e-050.00.00.00.0065339022908398990.00238978494885481960.0051701934081329440.0085029737160144980.00592853974836856850.00158500874614046640.0023153916286567240.00326895447176823220.0065339022908398990.00238978494885481960.0051701934081329440.0085029737160144980.00592853974836856850.00158500874614046640.0023153916286567240.00326895447176823220.00.00.00.00.00.00.00.00.00.00.00.00.00.00.00.00.00.00.00.00.00.00.00.00.0001335739064330.05.836238481932373e-050.00019842697926802040.000143932355212688350.00.00.00.000554607401090.00.00051388341239664310.00068019633377244380.00046974245709952730.00.00.05.20568808513e-050.05.8446238820500914e-054.9678018875650217e-054.804638485787537e-050.00.00.04.57441469844e-055.77597516685e-063.8964159213667276e-056.623735850086696e-053.203092323858358e-051.7327925500546994e-050.00.00.0001273879329720.00.000193229951698074259.604416982625708e-059.28896773918924e-050.00.00.05.82085877735e-050.00.00.000174625763320467440.00.00.00.00.00.00.00.00.00.00.00.08.406491888510001e-060.00.02.521947566553797e-050.00.00.00.07.12758217635e-060.0003510440839430.02.138274652903683e-050.00.000199731620158984230.00031414842930975370.00053925220236033130.00.00.00.00.00.00.00.00.000118403885857999990.00.000132936543199570720.000112993140972067160.000109281973402226350.00.00.04.42951586788e-050.00.00.000116754128298199631.6131347738099978e-050.00.00.03.31207621871e-050.01.923337220759747e-053.269588760042795e-054.743302675330675e-050.00.00.00.00.00.00.00.00.00.00.01.18101825732e-054.55331044057e-051.9151874867734765e-051.627867285190798e-050.01.7034231847995348e-053.700329801797464e-058.256178335123643e-050.000327099117412999970.000471170462423000040.00029307335892700750.00041517652662647160.000273047466685101540.00029542365204967160.00052849667140369540.00058959106381661052.79897024697e-057.70343837862e-051.920612947075951e-054.8974364217354886e-051.5788613721001538e-055.124746238688687e-055.566218483723663e-050.000124193504134579440.000343232908890999960.00.000209683413500353840.00047527011872787010.000344745194444136660.00.00.01.06769744129e-051.78254979862e-050.00.03.203092323858358e-053.465585100109399e-051.882064295741813e-050.05.62199097158e-050.00.0001163907931876075.226893595986781e-050.00.00.00.00.00.00.00.00.00.00.00.02.97246967654e-050.00.00.08.91740902962167e-050.00.00.03.44570509487e-050.03.8686240816993485e-053.288245471940329e-053.180245730963506e-050.00.00.06.06914185316e-060.00.01.8207425559480013e-050.00.00.00.04.24681637469e-050.0002500738252421.985289517768113e-059.123130135293452e-051.6320294710142147e-050.000173273689904000950.000272711143119936660.00030423664270140416.717115032699999e-050.03.879693106253566e-059.892961698841933e-056.378690293005485e-050.00.00.00.0002353447355910.00.000158162605826748950.00032007365260416640.000227797948341326350.00.00.00.0004734048201366.490021543399999e-050.000386414678585082460.00055475719599755160.000479042585825717963.440866233489503e-055.6059204700555e-050.000104232779266539560.01.26116651544e-050.00.00.01.710671794096555e-050.02.072827752222532e-050.00.00.00.00.00.00.00.00.0004534750324150.00.000320823539392686630.00054035094310330880.00049925061474868570.00.00.00.000380192755786999940.00.000208608113943941760.00058899288304723690.000342977270370064230.00.00.00.000182637117454000040.09.897465260114023e-050.000234238730808901080.00021469796895239410.00.00.00.01.34002623871e-050.00.00.04.020078716126903e-050.00.00.00.00.00.00.00.00.00.04.17820555133e-050.03.558931077783783e-056.050026445748478e-052.9256591304533033e-050.00.00.00.00.00.00.00.00.00.00.00.00.00.00.00.00.00.00.01.82104912981e-050.00.02.777180814253217e-052.68596657518725e-050.00.00.00.00.00.00.00.00.00.00.07.32465801987e-050.0001187122375446.396003493564252e-050.00010320064298568085.257906267465607e-050.00010799060190427240.000117293472962636550.000130852637764223740.0005096471411850.00.0005991800851477020.000437198642308385760.00049256269609744390.00.00.03.53739071644e-054.02759191142e-050.09.003692887130834e-051.6084792621972712e-051.740293816505153e-051.8984300722265242e-058.444051845516348e-050.000184225715884000020.00.000127021334017388460.000242922022042992150.0001827337915906080.00.00.00.00.00.00.00.00.00.00.03.97108645505e-051.31028856508e-053.846674441519494e-054.904383140064192e-053.162201783553783e-050.01.858037943030216e-052.072827752222532e-050.000338669293812999960.00.000321751497777944140.00042975696329037080.000264499420370134330.00.00.01.0719650906e-050.00.01.6347943800213974e-051.5811008917768916e-050.00.00.00.000137465301993.42194692066e-050.000136178897911755380.000132284652845346940.000143932355212688350.01.8793640600233888e-058.386476701962038e-050.00.00.00.00.00.00.00.00.000103831534947000020.00.00.00031149460484191490.00.00.00.00.00.00.00.00.00.00.00.00.00.00.00.00.00.00.00.01.68498543555e-050.0001008767698920.05.05495630664511e-050.05.289577258061714e-055.745248902790798e-050.000192282048068011160.0001206919124050.06.427073933749753e-050.000161940637938633380.000135864359939140230.00.00.02.01778681642e-050.06.053360449266167e-050.00.00.00.00.00.0004585491232811.90482946839e-050.000312286914059013640.00059586867539904520.000467491780383590651.7327925500546994e-051.882064295741813e-052.0996315593633397e-050.00.00.00.00.00.00.00.09.10812380304e-050.00.000113945608456774929.685126369034327e-056.244684194412933e-050.00.00.00.0002408329797720.000321449592872999950.000210670623545082440.000244180092778619730.000267648222993588160.00025551347771993020.000296026384143797140.00041280891675618212.96066143765e-056.34534717604e-054.5961503495894875e-052.178695723847042e-052.107138239513627e-056.367766951429746e-054.952426275373543e-057.715848301327661e-050.06.228800522280001e-060.00.00.00.01.868640156685167e-050.00.07.04941420799e-060.00.00.00.00.02.114824262397807e-050.0001415245494560.000161103834673999989.749522004690301e-050.000198953147252524690.000128125281068508328.641630776903478e-050.00018753322422146690.00020936197203038270.00.00.00.00.00.00.00.00.00.00.00.00.00.00.00.00.00.00.00.00.00.00.00.04.57353189649e-055.19808234062e-050.00.000137205956894652980.00.00.000155942470218607380.05.57585891589e-060.00.01.6727576747679032e-050.00.00.00.00.00.00.00.00.00.00.00.05.48823827579e-060.0001429139593910.01.6464714827358357e-050.00.000103373452700406087.485238570493154e-050.00025051603976860880.00059417007286747990.000106767605792380010.0005641459337905140.00075968961244471150.000458674672366970657.795887823450812e-055.874066142763687e-050.000183603277715085960.00059417007286747990.000106767605792380010.0005641459337905140.00075968961244471150.000458674672366970657.795887823450812e-055.874066142763687e-050.000183603277715085964.87822925577e-050.02.421344179706467e-058.232357413679178e-053.980986173938245e-050.00.00.00.00.00.00.00.00.00.00.00.0004141729790740.00.000447330297174873160.0004274557619282680.00036773287811828960.00.00.00.00.00.00.00.00.00.00.00.00.00.00.00.00.00.00.07.30227667457e-050.06.219966700163401e-050.000105736700726154585.113193250929856e-050.00.00.00.00.00.00.00.00.00.00.00.00.00.00.00.00.00.00.01.87480028292e-053.95157001482e-053.0402527816942186e-052.5841480670741815e-050.02.704088822506885e-055.874066142763687e-053.27655507918584e-058.46204139438e-060.00.02.5386124183151654e-050.00.00.00.00.00.00.00.00.00.00.00.03.09819902665e-055.93539120958e-050.09.294597079960364e-050.02.7224009364290538e-050.00.000150837726923227550.00.00.00.00.00.00.00.00.07.89799354838e-060.00.00.02.369398064514874e-050.00.00.000178289061796829980.000364317585551399975.338396616675675e-050.000245060593428883470.000236422625795600720.00052563939503825290.000458693971793924230.000108619389820372230.000178289061796829980.000364317585551399975.338396616675675e-050.000245060593428883470.000236422625795600720.00052563939503825290.000458693971793924230.000108619389820372230.04.24402097081e-050.00.00.05.756675965814181e-050.06.975386946619976e-050.02.36468721645e-050.00.00.03.2075096139310394e-050.03.8865520354172465e-058.93434137919e-060.000121458357685000010.02.680302413756012e-050.00.000160061674623657340.00020431339843076240.08.44451612654e-060.00.00.02.533354837960701e-050.00.00.00.0001250593390910.05.338396616675675e-050.00016358340736004540.000158210643746820980.00.00.00.00.00.00.00.00.00.00.00.00.00.00.00.00.00.00.00.01.39103069762e-050.00.00.04.173092092865297e-050.00.01.73732273304e-050.00.02.6494943400346786e-052.5624738590866863e-050.00.00.00.00.00.00.00.00.00.00.00.01.95034136796e-050.00.00.02.804706081018769e-053.0463180228751207e-050.01.84776378697e-050.00.02.8179218530931177e-052.7253695078305834e-050.00.00.00.00.0001433584253380.00.00.00.000206157882878302770.000223917393134410650.01.83033847078e-050.00.00.05.4910154123286136e-050.00.00.01.83033847078e-050.00.00.05.4910154123286136e-050.00.00.01.83033847078e-050.00.00.05.4910154123286136e-050.00.00.00.00330793507063970.00327898605748900030.0043621866770390960.00263158733945851550.00293003119541805070.0031188530853891970.0033820488749580890.00333605621211747940.00330793507063970.00327898605748900030.0043621866770390960.00263158733945851550.00293003119541805070.0031188530853891970.0033820488749580890.00333605621211747943.12234209721e-050.00.00.09.3670262916194e-050.00.00.00.06.04845165568e-050.00.00.08.204242277810006e-050.09.941112689230508e-050.00.0002648699791790.00.00.00.00030609736417209920.000265972944129198330.00022253962923607380.000264626197877999970.00.00052966903931078969.004140921211602e-050.000174168145109798220.00.00.00.000291749492715999950.00.000412980530142828850.000292520314191645950.000169747633812595220.00.00.00.00.000132576131000000020.00.00.00.000120602361483807080.00013099167498363020.000146134356531688470.04.34466583694e-050.00.00.00.00.000130339975108089770.04.07694532894e-056.56317480197e-056.712637329879314e-050.05.5181986569441016e-055.970413934841935e-056.484736385328227e-057.234374085727152e-050.04.61876719192e-050.00.00.00.06.54958374918151e-057.306717826584424e-050.0002773132319470.0004157500363980.000269841341420024140.000229359211525390080.000332739142896330860.000420008224072960.00039101992532426930.0004362219597960850.00.00.00.00.00.00.00.00.000112924498903999992.14977471451e-050.000217532525235661216.163262234840027e-055.960834912848708e-056.449324143519095e-050.00.06.53880798509e-050.00.000137660176714276285.8504062838329186e-050.00.00.00.00.0001226111305330.000219736862764000020.000137660176714276280.000117008125676658350.00011316508920839680.00024487789133767930.000265972944129198330.00014835975282404920.02.17233291847e-050.00.00.00.06.516998755404487e-050.00.06.0225954361000004e-050.00.00.00.000117089671343502026.358819173962631e-050.00.04.25709029556e-050.00.00.06.121947283441984e-056.649323603229958e-050.00.00.00.00.00.00.00.00.00.00.000181486094334000020.00.00.00.000122438945668839670.000199479708096898820.00022253962923607380.000157076401970.00.000108476219250849720.00018440480606641360.00017834818059243340.00.00.00.0001031097762540.00.000137660176714276285.8504062838329186e-050.00011316508920839680.00.00.09.362167718999999e-059.61747798324e-050.000105112615553148968.934341379186706e-058.640900222501617e-050.00018698040540125130.000101543934095837370.00.0008834131328690.00.0011429316752537410.00080443347379608170.00070287424955668520.00.00.00.00.00.00.00.00.00.00.00.000138029970227999980.000127712708867000026.883008835713814e-050.000175512188514987540.000169747633812595220.000183658418503259560.000199479708096898820.00.06.45565132499e-050.00.00.00.000120602361483807080.07.306717826584424e-056.13055652666e-050.06.883008835713814e-055.8504062838329186e-055.65825446041984e-050.00.00.02.23754577663e-050.0001130765244256.712637329879314e-050.00.00.00.000194542091559846860.000144687481714543047.06268792837e-050.0001580115921147.929548190851587e-056.73994174219348e-056.518573852062624e-050.000141055393548312387.660331870387729e-050.00025637606409068150.000182635683644000030.00.000206490265071414435.8504062838329186e-050.000282912723020992050.00.00.00.06.59582741292e-050.00.00.06.0001174867565723e-056.516998755404487e-057.270365996601417e-057.97934558957e-050.000404185160421000050.00013038007121496365.5410097976686795e-055.359019849532254e-050.00040587333191665850.000314883834095264930.00049179831525087470.0003093415641820.0006731228722640.000474583459222467550.000230506007583016980.00022293522574054170.00042210826519332490.00072045421240996610.00087680613919013070.00024068400446070.000343415265519999940.000208608113943941768.665639382820188e-050.00042678750561086940.000278313141885708670.00030228848073145430.000449644173943656860.00024068400446070.000343415265519999940.000208608113943941768.665639382820188e-050.00042678750561086940.000278313141885708670.00030228848073145430.000449644173943656865.68222096897e-050.00.08.665639382820188e-058.381023524080516e-050.00.00.00.0001838617947710.000343415265519999940.000208608113943941760.00.000342977270370064230.000278313141885708670.00030228848073145430.000449644173943656860.00298130365440570.00435323309061980.00250480852372173860.00313288398003878750.00330621845945464930.0054762451988516540.0040244889803708710.00355896509264954850.00298130365440570.00435323309061980.00250480852372173860.00313288398003878750.00330621845945464930.0054762451988516540.0040244889803708710.00355896509264954850.000265428379150.000378788945713000060.000258276712502023140.000219529531031444830.00031847889391505960.00034457817566802030.00037426192852465770.00041752673294768130.00.000119806684032000010.00.00.00.000172289087834010140.000187130964262328840.00.0003509387881974.3696507784e-050.00043588675057435560.00049576916524561930.000121160448772033540.000131089523351964230.00.07.21473222461e-050.000162844036548999980.00.00.000216441966738390.000234179342687004040.000254352766958505230.00.0002357131858680.0002752677930520.00020238100606501810.000172019408644042550.000332739142896330860.000360007049205394340.00.000465796329951751830.07.98711226881e-050.00.00.00.000114859391889340090.000124753976174885920.03.80372949807e-050.00.00.000114111884942087650.00.00.00.00.00.00.00.00.00.00.00.00.00.000116478720587000010.00.00.00.000167503279838620940.00018193288192170860.08.07395055339e-050.0001693891354520.000132936543199570720.00.000109281973402226350.000236475218595700150.000128423210768264920.000143268976991851430.08.71688620966e-050.00.00.00.000118237609297850080.00.000143268976991851430.000119576201560000010.000390361671780999950.000134252746597586310.000114111884942087650.000110363973138882030.00047763311478735480.00025938945541312910.00043406244514362910.08.22202733554e-050.00.00.00.000118237609297850080.000128423210768264920.07.4825286027e-050.000390361671780999950.00.000114111884942087650.000110363973138882030.00047763311478735480.00025938945541312910.00043406244514362910.000117254333567999990.0001681272267240.000131645897149089420.000111896120185930590.0001082209833691950.00023532728064135210.00012717638347925260.000141878016050182980.0005379328834590.000110139142907999990.00037149390154400590.00063152330844662190.00061078144038504570.00033041742872275910.00.00.00.00.00.00.00.00.00.00.000207799782279000040.0003488845552620.00017841483429416070.000151648689199353320.000293335823342818060.000317374635483702870.000344714934167447860.00038456409613602230.000118403885857999990.000129976599018999970.000132936543199570720.000112993140972067160.000109281973402226350.000118237609297850080.000128423210768264920.000143268976991851430.0007625068056790.001221790360940.00052658358859635780.00089516896148744470.000865767866953560.00128798638477852250.00152611660175103150.00085126809630109780.07.80597808957e-050.00.00.00.000234179342687004040.00.00.00.0002598438820730.00.00.00.000194519937877108180.00034931113328968050.00023570057505111040.00.0002598438820730.00.00.00.000194519937877108180.00034931113328968050.00023570057505111040.00.000116437044430.00.00.00.00.00034931113328968050.00.00.0001434068376430.00.00.00.000194519937877108180.00.00023570057505111040.00017601419101730.00051995165768949990.00028546373487065710.00.00024257883818012820.00051122854687244320.00042295360461893610.00062567282157860720.00017601419101730.00051995165768949990.00028546373487065710.00.00024257883818012820.00051122854687244320.00042295360461893610.00062567282157860724.17481696143e-050.000148961944942999990.00.00.000125244508843000960.000135508271330120320.000147181657284977750.000164195906215380330.04.06068557185e-050.00.00.00.000121820567155360690.00.00.0001342660214030.0003303828570280.00028546373487065710.00.000117334329337127220.00025389970838696230.00027577194733395830.000461476915363226840.00.0006368897122130.00.00.00.00049225453666860030.00141841459996964750.00.00.0006368897122130.00.00.00.00049225453666860030.00141841459996964750.00.00.000342304811520.00.00.00.00049225453666860030.00053465989789236810.00.00.0001343504358810.00.00.00.00.000403051307641939130.00.00.000160234464812000020.00.00.00.00.000480703394435340260.00.08.20979531077e-050.00.00.00.00.00.000246293859323070460.08.20979531077e-050.00.00.00.00.00.000246293859323070460.08.20979531077e-050.00.00.00.00.00.000246293859323070460.00032856933723870.000113241653974999990.00053701098639034510.000117605105909702570.000331091919416646160.000339724961926217140.00.00.00032856933723870.000113241653974999990.00053701098639034510.000117605105909702570.000331091919416646160.000339724961926217140.00.08.15389065788e-050.00.000134252746597586310.00.000110363973138882030.00.00.00.000207828728689999990.00.00040275823979275880.00.000220727946277764120.00.00.03.92017019699e-050.00.00.000117605105909702570.00.00.00.00.00.00.00.00.00.00.00.00.00.000113241653974999990.00.00.00.000339724961926217140.00.00.0012606681869150.000299365734471599970.00139154542800840960.00097684392840367750.00141361520433202440.000354712827893550240.000256846421536529830.000286537953983702860.0012606681869150.000299365734471599970.00139154542800840960.00097684392840367750.00141361520433202440.000354712827893550240.000256846421536529830.000286537953983702860.000353974601716999978.71688620966e-050.000398809629598712160.000225986281944134370.00043712789360890530.000118237609297850080.00.000143268976991851430.000200380447249000020.000212196872374999980.000265873086399141440.000225986281944134370.000109281973402226350.000236475218595700150.000256846421536529830.000143268976991851430.0003511014803750.00.00032805308241184390.000185891941599207250.00053935941711421370.00.00.00.0003552116575740.00.000398809629598712160.00033897942291620150.0003278459202066790.00.00.07.53307078131e-050.00.000225992123439270250.00.00.00.00.07.53307078131e-050.00.000225992123439270250.00.00.00.00.07.53307078131e-050.00.000225992123439270250.00.00.00.00.03.55640182999e-050.00.05.4236707666592244e-055.245534723306864e-050.00.00.03.55640182999e-050.00.05.4236707666592244e-055.245534723306864e-050.00.00.03.55640182999e-050.00.05.4236707666592244e-055.245534723306864e-050.00.00.00.00088339555067199990.00.00113150995914749530.00066471478067554680.00085396191219503940.00.00.00.00088339555067199990.00.00113150995914749530.00066471478067554680.00085396191219503940.00.00.00.00.00.00.00.00.00.00.01.5165661615e-050.00.00.04.549698484500852e-050.00.00.00.0001815780734920.00.0001849026464503120.00015716318698842070.00020266838703685610.00.00.00.00.00.00.00.00.00.00.00.000466063754240999960.00.0006989447116678460.00029704382420491880.000402202726851492830.00.00.00.000220588061324000020.00.000247662601029337250.000210507769482207330.000203593813461681950.00.00.00.00136737332717869980.00109397600460290.00114144150047267780.00139470636813592140.0015659721129273650.00135947178149631180.00137353614023332030.00054892009207924480.00136737332717869980.00109397600460290.00114144150047267780.00139470636813592140.0015659721129273650.00135947178149631180.00137353614023332030.00054892009207924480.06.40188387958e-050.00.00.09.20628713616848e-059.999364502567192e-050.00.00.00.00.00.00.00.00.00.00.00.00.00.00.00.00.00.0001424236705070.00.000123268430966874680.00.00030400258055528420.00.00.00.03.06876237872e-050.00.00.09.20628713616848e-050.00.00.0007559186513440.00.00071906584730676910.00087312881660233720.00067556129012285370.00.00.00.000117168277345000010.000236707790047000030.00.000178686827583734130.000172818004450032340.000280470608101876960.000203087868191674760.000226564893847579055.81334914517e-056.45112914019e-050.08.865615676269886e-058.574431759251606e-059.277104729523625e-050.000100762826910484770.00.00.0001589422927110.00.00.00.000181356934562115930.00029546994357209820.00.00029372923653099990.000539108167860.000299107222199034140.00025423456718715120.0003278459202066790.00062074744881371290.00067422185653339070.000322355198231665760.00131283555730870.00114313339543740.0011479442451063650.00112302303069257250.00166753939612354240.00114593772555613520.00107990540233177780.00120355705841986210.00131283555730870.00114313339543740.0011479442451063650.00112302303069257250.00166753939612354240.00114593772555613520.00107990540233177780.00120355705841986215.29215776066e-050.000109352479848999998.581979371111528e-057.294493910854968e-050.00.000152661217068110248.290612340736089e-059.24900990706889e-050.0002227516539980.08.692004747664241e-050.00029552052254232950.00028581439197505350.00.00.00.0001530459427880.00.000172732833201997.340955655510097e-050.00021299543860561310.00.00.02.35163740233e-050.0002463403341670.00.07.054912206979167e-050.000305322434136220530.00024871837022208270.00018498019814137780.00.00.00.00.00.00.00.05.27912151568e-050.08.692004747664241e-050.07.145359799376338e-050.00.00.00.08.39089436707e-050.00.00.07.633060853405512e-058.290612340736089e-059.24900990706889e-050.000483329345099000060.00.000458092142106628930.00038936825605239360.00060252763713659920.00.00.04.78313537261e-058.39089436707e-050.07.294493910854968e-057.054912206979167e-057.633060853405512e-058.290612340736089e-059.24900990706889e-052.38178659979e-050.0001129743735010.00.07.145359799376338e-057.730920607936352e-050.000167938044850807959.367586957159516e-050.00.00.00.00.00.00.00.00.00.000143377171427000020.00.00.00.000152661217068110240.00.000277470297212066740.0002528302289130.00036327114915199990.000257459381133345830.000218834817325649080.000282196488279166740.000305322434136220530.00041453061703680450.00036996039628275560.00304616282444570.00343811373383060.0036647996546907720.00250645456474743760.00296723425389537180.003500456365745610.003022824735805570.00379106009994520250.00304616282444570.00343811373383060.0036647996546907720.00250645456474743760.00296723425389537180.003500456365745610.003022824735805570.00379106009994520250.0001429287106250.00.0001896437399490380.00016119301229581617.794937962956003e-050.00.00.00.0001327100037340.000243216058393000039.224168303643685e-057.840340393980171e-050.000227484924225042520.000164084845556200090.000267329948946184070.000298233380676915240.00.0001721767935060.00.00.00.000156626443485463780.000170119058420298940.000189784878612582450.000379560201450.0001626700993860.00058231099291100310.000282829457157076150.00027354015428287330.000147978357648843050.000160725981575006380.000179305958934587085.3598254530000007e-053.45471292037e-050.08.173971900106986e-057.905504458884459e-050.00.00.000103641387611126580.0002236517843980.00.000251102359376966940.00021343148850279350.00020642150531531640.00.00.00.000230361666139000050.00.0003792874798980760.00.00031179751851824010.00.00.08.71000964775e-050.00.000141245077149543880.000120055212282821370.00.00.00.08.47589834052e-050.09.616686103798732e-050.00.000158110089177689150.00.00.00.02.73474742594e-050.00.00.08.204242277810006e-050.00.03.2055620346e-059.40256248225e-059.616686103798732e-050.00.08.553358970482772e-059.290189715151078e-050.000103641387611126580.0002215449803450.00.00019097925924445378.116408717711867e-050.000392491594613629730.00.00.00.0002410022700640.000388098851129000040.00018448336607287370.000235210211819405080.000303313232300056840.00041021211389050040.000356439931928245360.00039764450756922030.0001335683690220.000264773964614000059.224168303643685e-050.00015680680787960340.000151656616150028420.000328169691112400170.000267329948946184070.000198822253784610160.00.00.00.00.00.00.00.00.06.55144282542e-050.00.00.00.09.290189715151078e-050.000103641387611126585.86992528414e-050.00.00017609775852410670.00.00.00.00.05.44931460007e-050.0002511095754170.00.000163479438002139720.00.000256600769114483150.000185803794303021610.000310924162833379675.93021933464e-056.3058325772e-059.616686103798732e-058.173971900106986e-050.08.553358970482772e-050.00.000103641387611126580.00.0002039632784610.00.00.00.00018554209459047250.000201525653820969570.000224822086971828430.00.00.00.00.00.00.00.08.5653874876e-050.0002165624462139.616686103798732e-058.173971900106986e-057.905504458884459e-050.000256600769114483150.000185803794303021610.00020728277522225320.00.00.00.00.00.00.00.00.00.00.00.00.00.00.00.00.00.000285656704620999970.00.00.00.000256600769114483150.000185803794303021610.00041456555044450630.0001041137617030.000114289768102999990.000116892477641001839.935603775130043e-059.609276971575076e-050.000103967553003281980.000112923857744508790.000125977893561800420.06.3058325772e-050.00.00.08.553358970482772e-050.00.000103641387611126580.000147282882409000030.00.000165360090321417260.00014055244364818110.000135936113256427880.00.00.00.0001976594312825.94784956188e-050.000192333722075974630.000163479438002139720.000237165133766533758.553358970482772e-059.290189715151078e-050.00.00.00.00.00.00.00.00.00.0001713077497520.0004701281241120.000192333722075974630.000163479438002139720.000158110089177689150.00042766794852413860.0004645094857575540.0005182069380556338.5653874876e-052.85111965683e-059.616686103798732e-058.173971900106986e-057.905504458884459e-058.553358970482772e-050.00.00.00.00.00.00.00.00.00.00.00.00.00.00.00.00.00.03.2055620346e-050.000188051249645000029.616686103798732e-050.00.00.000171067179409655430.000185803794303021610.00020728277522225320.00.00.00.00.00.00.00.08.71000964775e-054.18758199597e-050.000141245077149543880.000120055212282821370.00.00012562745987896570.00.00.00768184578541230.0063404988169837010.0081861263646249850.0069927038057968660.0078667071858162080.0049410460843885870.0055313278291588470.0085491225373860480.00768184578541230.0063404988169837010.0081861263646249850.0069927038057968660.0078667071858162080.0049410460843885870.0055313278291588470.0085491225373860480.0003229256608020.00.000313515084540028130.000333101167027481240.000322160730838933150.00.00.09.58973683769e-050.00115913327772000018.168390003829045e-050.000138859040712660866.714916437968124e-050.00078416564473084030.00064704325203583860.00204619093638703980.00041543499371199990.000184443157855999960.000391718303278197630.00031398633386542520.000540600343993040.000164279854286120177.84381287327127e-050.00031061149054822484.60091831503e-050.00.00.00013802754945090840.00.00.00.00.0005473617870160.00.0005084822777383580.00057626501895754250.00055733806435135430.00.00.00.000418862301416999960.00.00047027262681004210.000399721400432977430.00038659287700671970.00.00.00.0002744648819060.00.000233784955282003660.00039742415100520170.00019218553943150150.00.00.00.0003759156354417.79859594116e-050.0004274421158263120.000283352030437645360.000416952760057725157.094256557871005e-057.705392646095893e-058.596138619511087e-059.03945127777e-050.00.00.00027118353833296120.00.00.00.00.0001558297638590.00.000107615296875842950.00018294127585953730.000176932718841699770.00.00.00.00065749087202900010.000100451696604999990.00078834461664861710.00053606048275120240.00064806751668762130.000140235304050938487.615795057187803e-058.496183519284212e-050.000118627794526000020.00062318936061800010.00011491124920640860.000146508055667171859.446407870361935e-050.00035771886880790240.00070687182537278010.00080497738767455510.0003412128926940.00.000235136313405021060.00046634163383847360.000322160730838933150.00.00.05.98654631627e-050.00.000179596389488161770.00.00.00.00.04.93139494878e-050.08.119477488836057e-050.06.67470735750125e-050.00.00.00.000270012914486000030.0004093272810670.000251102359376966940.000283707710326884130.00027522867375375520.000295967854891005050.000483182179629470.00044883180867907930.00069471058283600010.0003940770783460.00086792067038639970.00065365030098524260.00056256077713644060.00029461956146350990.000385182934692873370.00050242873888076440.00.0001410384372340.00.00.00.000128300384557241580.000139352845727266160.000155462081416689864.54996108534e-050.000176194074415999967.491451605721665e-050.06.158431650291208e-050.000133262277882659777.23710911511769e-050.00032294885421367620.0004832248018590.0007743038114960.0003625542087261020.000431428356438801740.0006556918404133580.00090290538009267320.00056039219244333770.00085961386195110860.02.5385983524e-050.00.00.00.07.615795057187803e-050.00.0004151226160890.0001119701137440.00049307372386749870.00027940122131274790.00047289290308599770.00.000158777787858945670.00017713255337174360.0002711610226060.000189631987760.00015952385183948490.000232443032856823880.000421516183122872977.094256557871005e-050.00015410785292191790.00034384554478044350.0007100404860110.00.00124597900671004040.00040807283639098710.00047606961493342610.00.00.00.07.70791459301e-050.00.00.07.011765202546924e-057.615795057187803e-058.496183519284212e-052.06421505315e-050.00.00.06.192645159459492e-050.00.00.09.46245973524e-050.000195586797980999970.00.000115832164614581420.00016804162744261940.000242416807002627280.00019747488690999530.000146868700031847687.37717077786e-050.000447316450614999978.370078645898896e-050.00.00013761433687687760.000297783608601992840.0004935552929477810.00055061045029576380.0006334282346520.001253384202660.00071365933717664280.00040439650453160880.00078222886224751480.00098738775483818670.00114904978055815960.00162371507257431630.00509966772996130.00816986747721190.0044426957174112560.00653957184921331350.0043167356232574840.00901472892160190.0074037677157201970.008091105794319960.00509966772996130.00816986747721190.0044426957174112560.00653957184921331350.0043167356232574840.00901472892160190.0074037677157201970.008091105794319960.00.00.00.00.00.00.00.00.000110799966582999980.0001216294779810.000124399334003268020.000105736700726154580.000102263865018597120.000110644368333767970.000120175848608835040.000134068217001549070.0002673365350730.0001949648985290.000299107222199034140.00033897942291620150.00016392296010333950.000177356413946775120.000192634816152397360.00021490346548777720.00.00.00.00.00.00.00.03.07073313692e-050.00.00.09.212199410766188e-050.00.00.00.0002247264804540.000109567050412999980.000112062209969886080.00028575124906985580.000276365982322985649.967137312711329e-050.000108257582631099360.000120772195480734280.00.00.00.00.00.00.00.00.00059890235432000010.000307882175075999970.00070745360380988940.00070154002307874740.00038771343607050740.00031461485604471410.000227811608667182970.00038122006051744820.0001707352195320.0006537329515970.000117908933968314910.000200440006593927820.00019385671803525370.00062922971208942830.00056952902166795740.00076244012103489640.07.356550773899999e-050.00.00.00.000105791545161234270.000114904978055815970.00.0001312738734510.000388616962866999940.000147386167460393620.000125275004121204920.000121160448772033540.000262179046703928460.000427146766250968050.000476525075646810150.000435340895005999970.0006440432124210.00066627905544000160.00036588255171907460.00027386107785932660.00069747949567584780.00064365819591680260.00059099194566990636.68792379533e-050.00.00.000101993808665051799.864390519492996e-050.00.00.00.0003096717014750.00037429844458199990.000347680189906569640.00029552052254232950.00028581439197505350.000412315765756605540.000335876089701615850.00037470347828638070.000197148362315000010.0006537329515970.00.00030066000989089170.00029078507705288050.00062922971208942830.00056952902166795740.00076244012103489646.35143093278e-050.0003672648958790.00.00.000190542927983369050.00041409298888486670.00056172380519063340.000125977893561800420.0004857925158150.000334061015189000040.000471635735873259660.00050110001648481970.00048464179508813420.00041948647472628550.000455623217334365940.00012707335350581616.57161207715e-050.00.00.000100220003296963919.692835901762684e-050.00.00.07.28799076501e-050.00.000119995817755364720.09.864390519492996e-050.00.00.00.000204141887297999960.000450722395202999960.000117908933968314910.00030066000989089180.00019385671803525370.00062922971208942830.000341717413000774540.00038122006051744826.87032171702e-050.00.00.000104775457992280440.000101334193518428040.00.00.00.0001039218840010.0001925985829030.000117908933968314910.00.00019385671803525370.00020974323736314280.000113905804333591490.000254146707011632140.0002012866059590.0001146864095960.000225992123439270250.000192088339652514160.00018577935478378480.000100501967903172560.00.000243557260886147470.00.00.00.00.00.00.00.00.000389961371558000056.99144124544e-050.000471635735873259660.00060132001978178349.692835901762684e-050.00020974323736314280.00.06.57161207715e-057.29258076717e-050.00.000100220003296963919.692835901762684e-050.000104871618681571380.000113905804333591490.00.0001325294563030.0002305671843470.00.00030066000989089179.692835901762684e-050.00020974323736314280.000227811608667182970.000254146707011632140.00.000131263496038999980.00.00.00.00011940827869683870.000129694727706564540.000144687481714543040.03.55759178418e-050.00.00.00.0001067277535254930.00.00.0001384257665270.0003004815968020.000117908933968314910.000200440006593927829.692835901762684e-050.00041948647472628550.000227811608667182970.000254146707011632149.83718736687e-050.0003651771878550.00015952385183948490.00013559176916648060.00.00042565539347226030.00015410785292191790.00051576831717066530.000105019098761000010.0001502407984010.000117908933968314910.000100220003296963919.692835901762684e-050.00020974323736314280.000113905804333591490.00012707335350581610.00.000463825456228000050.00.00.00.00049025350196669550.000425989186938634860.000475233679777848850.00036016563684800010.0013685286880.00.00108049691054539240.00.00150752951854758840.0012280469529715330.00137000959248457960.00016361674482750.00114821211677339980.000156876259712596880.00023788120505412949.609276971575073e-050.00092787196214567280.00112390179114194280.0013928625970331870.00016361674482750.00114821211677339980.000156876259712596880.00023788120505412949.609276971575073e-050.00092787196214567280.00112390179114194280.0013928625970331872.13815874392e-050.000122328648466000030.04.492620837450107e-051.9218553943150147e-055.243580934078569e-050.000130598896347997130.00018395123970897450.00.00056415374893499990.00.00.00.00051320153822896630.00055741138290906470.00062184832566675934.5696676326e-050.000197728367565999986.336227759979539e-057.37277513780678e-050.00.000154299508569356810.00018363318922938810.00025525240489904980.00.00.00.00.00.00.00.00.05.04783299664e-050.00.00.00.07.158015026427878e-057.985483963480244e-059.65384810623e-050.000213523021849.351398211280148e-050.000119227245301560527.687421577260059e-050.000207935106006563950.000180678172391214080.000251955787123600830.00063690456583600010.00138062354105510010.00066724935331547110.00055114647912142520.00069231786507086630.00137252192893761960.00129479297293994050.00147455572128741780.00063690456583600010.00138062354105510010.00066724935331547110.00055114647912142520.00069231786507086630.00137252192893761960.00129479297293994050.00147455572128741780.07.77701809041e-050.00.00.00.00010963851043982467.938889392947285e-054.42831383429359e-050.000225684485589000030.0003838667905260.00015585663685466910.00026494943400346780.000256247385908668660.00034655851001093990.000301130287318690150.00050391157424720171.16771027144e-050.00.03.5031308143315664e-050.00.00.00.00.00.00.00.00.00.00.00.00.000262477945120.000355127344883000040.00032839943232410330.000173395601488426140.00028563880154748040.000328969416563792330.00031724993367868060.00041916268440514077.55116205278e-050.0001135141446690.000100814330158782264.284498282212212e-058.287554860243187e-050.00013450077488900429.73915799134797e-050.000108650079205716321.12593548354e-050.00.00.03.377806450614269e-050.00.00.03.65975647197e-050.000226766602980999974.1089476988958225e-053.4925152664093486e-053.377806450614269e-050.00025582319102625750.000158777787858945670.000265698830057615451.36964923297e-050.0001469867200974.1089476988958225e-050.00.00.000109638510439824640.000198472234823682140.000132849415028807720.07.659175699500001e-050.00.00.08.739301556797616e-050.000142382255416989350.00.000168008334608500010.00057336666188490.000144250291556981010.000122609578501604780.000237165133766533750.00078980919305964520.00054163558905314770.000388655203541724660.000168008334608500010.00057336666188490.000144250291556981010.000122609578501604780.000237165133766533750.00078980919305964520.00054163558905314770.000388655203541724661.97637611472e-054.46088717141e-050.00.05.929128344163344e-056.415019227862079e-056.967642286363308e-050.02.04349297503e-050.0001875621110540.06.130478925080239e-050.00.000291701817719766250.000193253474734982347.773104070834493e-050.00.00.00.00.00.00.00.00.07.05192186169e-050.00.00.06.415019227862079e-056.967642286363308e-057.773104070834493e-050.0001278096437110.0002115576558510.000144250291556981016.130478925080239e-050.00017787385032490030.000192450576835862350.000209029268590899230.00023319312212503480.05.91188046489e-050.00.00.00.000177356413946775120.00.00.00011881883784820.00024740834267730.09.22024030332068e-050.000264254110511301830.00041573086283213920.000209586679973808320.000116907485225350780.00011881883784820.00024740834267730.09.22024030332068e-050.000264254110511301830.00041573086283213920.000209586679973808320.000116907485225350785.83600067384e-054.20950650903e-050.00.00.000175080020215085120.000126285195271002180.00.06.04588311098e-050.0002053132775870.09.22024030332068e-058.91740902962167e-050.0002894456675611370.000209586679973808320.000116907485225350780.00074591047330120.00179622611262070.00081076037754012280.00047990894990041430.00094706209246373770.00187023507292705050.00155708152373184360.00196136174120067640.00074591047330120.00179622611262070.00081076037754012280.00047990894990041430.00094706209246373770.00187023507292705050.00155708152373184360.00196136174120067645.99288241238e-050.000213832469353999980.00.00.000179786472371404580.000194519937877108180.000211276895134887450.00023570057505111040.0002182282011870.0002588957204590.000273010618919923740.000232053027768137880.00014962095687284680.000404705911019486840.00017582775165587950.000196153498700252960.000255400699230999950.000449278265140.000218702054941229250.00024785592213227640.00029964412061900770.000453879855046585860.00042255379026977490.00047140115010222080.0001937748132810.0006238876752930.000319047703678969730.00.00026227673616534320.00056754052462968040.00061643141168767160.0006876910895608870.06.90443991977e-050.00.00.00.00012898648287038190.07.814671472282806e-051.85779354784e-050.000181287583177000020.00.05.573380643513542e-050.000120602361483807080.00013099167498363020.00029226871306337690.00016537210696340.0005263399840260.00037422351623277016.196398053306909e-055.9928824123801536e-050.00051775945127691910.000433889333937539860.00062737116686319310.00016537210696340.0005263399840260.00037422351623277016.196398053306909e-055.9928824123801536e-050.00051775945127691910.000433889333937539860.00062737116686319310.000100440943750999990.000294613624444000030.00030132283125236030.00.00.000268005247741793530.00029109261107473380.000324743014514863330.04.27462689127e-050.00.00.05.798190455952264e-050.07.025690217869639e-050.00.00.00.00.00.00.00.00.00.00.00.00.00.00.00.00.04.44561196692e-050.00.00.06.030118074190354e-050.07.306717826584424e-050.07.32464812153e-050.00.00.06.663113894132988e-057.23710911511769e-058.073721355341904e-056.49311632124e-057.12774897848e-057.290068498040976e-056.196398053306909e-055.9928824123801536e-056.483997929236941e-057.042563171162915e-057.856685835037017e-050.000105019098761000010.00046315688785490.000117908933968314910.000100220003296963919.692835901762684e-050.000421326327685611360.00045762156477881490.00051052277110231360.000105019098761000010.00046315688785490.000117908933968314910.000100220003296963919.692835901762684e-050.000421326327685611360.00045762156477881490.00051052277110231360.03.83016593519e-050.00.00.00.00.000114904978055815970.00.000105019098761000010.0002305671843470.000117908933968314910.000100220003296963919.692835901762684e-050.00020974323736314280.000227811608667182970.000254146707011632140.00.0001942880441560.00.00.00.000211583090322468550.000114904978055815970.00025637606409068150.00054380748844370.00076393082794530010.000322845890627528860.00072237641909215040.00058620015561322690.00072090577335773060.0009389508093229590.00063193590115634910.00054380748844370.00076393082794530010.000322845890627528860.00072237641909215040.00058620015561322690.00072090577335773060.0009389508093229590.00063193590115634910.0002875522942260.000267799374807000040.000322845890627528860.000274411913789305950.000265399078262549640.00014357423986167510.00031188494043721480.000347938944123067763.47251130437e-057.8378204507e-050.00.00.000104175339131094260.0001127124873680440.00012242212615292540.00.000107538874356000010.000125071821698000020.00.000217458497719827370.000105158125349312120.000113775812720572730.000123577051871349240.000137862600501592880.07.62406171113e-050.00.00.00.000109638510439824640.000119083340894209250.00.0001139912068180.000216440809822000020.00.000230506007583016980.000111467612870270840.000241204722967614150.00026198334996726040.000146134356531688470.00116913134661559980.001990189279060.00098639653776978770.0012579282802257040.00126306922184947230.00161072237015819640.0018379862453500020.002521859221669610.00116913134661559980.001990189279060.00098639653776978770.0012579282802257040.00126306922184947230.00161072237015819640.0018379862453500020.002521859221669618.16026780913e-050.0001190810963939.161842842132578e-057.787365121047872e-057.531595464207491e-058.148808208365345e-050.00017701577700490579.87394300889787e-050.03.2913143363e-050.00.00.00.00.09.87394300889787e-050.0001218928046570.0001571337167010.00.000185891941599207250.000179786472371404580.00.00.00047140115010222080.00.00.00.00.00.00.00.08.16026780913e-050.0002063196278119.161842842132578e-057.787365121047872e-057.531595464207491e-050.000244464246250960330.00017701577700490570.00019747886017795740.0005306635201690.0008652114610270.00036647371368530310.00062298920968382980.00060252763713659920.00073339273875288080.00097358677352698120.00088865487080080820.0001372474724460.0003288112380660.000183236856842651557.787365121047872e-050.000150631909284149820.00032595232833461380.00026552366550735850.00039495772035591480.000191016874946999990.0002478058523360.000253449110399181540.000215426175311230840.000104175339131094260.0002254249747360880.000244844252305850830.00027314832996577282.5105318214000002e-053.2913143363e-050.00.07.531595464207491e-050.00.09.87394300889787e-050.00018007590523940.000808139588690.00015585663685466910.00.0003843710788630030.00083174042402625570.00075282571829672530.00083985262374533590.00018007590523940.000808139588690.00015585663685466910.00.0003843710788630030.00083174042402625570.00075282571829672530.00083985262374533590.00.00.00.00.00.00.00.00.0001373680075880.0006095454298840.00015585663685466910.00.000256247385908668660.00055449361601750380.00060226057463738030.00067188209899626874.27078976514e-050.000198594158805999970.00.00.000128123692954334330.00027724680800875190.000150565143659345050.000167970524749067180.00.000211276895134999960.00.00.00.00.00063383068540466220.00.00.000211276895134999960.00.00.00.00.00063383068540466220.00.00.000211276895134999960.00.00.00.00.00063383068540466220.00.0001965148899470.00.00058954466984157450.00.00.00.00.00.0001965148899470.00.00058954466984157450.00.00.00.00.00.0001965148899470.00.00058954466984157450.00.00.00.00.00.00.00027380097030.00.00.00.000202126862822023040.000292718826779061850.00032655722129986250.00.00027380097030.00.00.00.000202126862822023040.000292718826779061850.00032655722129986250.00.00027380097030.00.00.00.000202126862822023040.000292718826779061850.00032655722129986250.00.0003238253837050.00.00.00.0003381374621041320.000428477441535238950.000204861247474329630.00.0003238253837050.00.00.00.0003381374621041320.000428477441535238950.000204861247474329630.00.0003238253837050.00.00.00.0003381374621041320.000428477441535238950.000204861247474329638.44451612654e-050.000403413438159000060.00.00.000253335483796070140.000274096276099561550.00052098961641216560.000415154421965024068.44451612654e-050.000403413438159000060.00.00.000253335483796070140.000274096276099561550.00052098961641216560.000415154421965024068.44451612654e-050.000403413438159000060.00.00.000253335483796070140.000274096276099561550.00052098961641216560.000415154421965024060.00.000300612235864000060.00.00.00.00033500655967724190.00036386576384341720.000202964384071789540.00.000300612235864000060.00.00.00.00033500655967724190.00036386576384341720.000202964384071789540.00.000300612235864000060.00.00.00.00033500655967724190.00036386576384341720.000202964384071789540.00.000101981639231000010.00.00.09.277104729523625e-050.000100762826910484770.000112411043485914220.00.000101981639231000010.00.00.09.277104729523625e-050.000100762826910484770.000112411043485914220.00.000101981639231000010.00.00.09.277104729523625e-050.000100762826910484770.000112411043485914220.07.86736786688e-050.00.00.00.00.000236021036006540920.00.07.86736786688e-050.00.00.00.00.000236021036006540920.00.07.86736786688e-050.00.00.00.00.000236021036006540920.00.000350311543689999940.0004242436191990.000325428657752549150.00036880961213282720.00035669636118486680.000385927556748182650.000419173359947616640.000467629940901403130.000350311543689999940.0004242436191990.000325428657752549150.00036880961213282720.00035669636118486680.000385927556748182650.000419173359947616640.000467629940901403130.000350311543689999940.0004242436191990.000325428657752549150.00036880961213282720.00035669636118486680.000385927556748182650.000419173359947616640.000467629940901403130.00.00.00.00.00.00.00.00.00.00.00.00.00.00.00.00.00.00.00.00.00.00.00.02.22652338364e-050.06.679570150914392e-050.00.00.00.00.02.22652338364e-050.06.679570150914392e-050.00.00.00.00.02.22652338364e-050.06.679570150914392e-050.00.00.00.00.00.00149994519576889970.00163178184111160.0015458451482745960.0013377116083327580.00161627883070005550.00159316725170143350.00158885519128470910.00171332308034767280.00149994519576889970.00163178184111160.0015458451482745960.0013377116083327580.00161627883070005550.00159316725170143350.00158885519128470910.00171332308034767283.45084652453e-050.0001721767935060.05.262694237055183e-055.0898453365420475e-050.000156626443485463780.000170119058420298940.000189784878612582450.0006233602214790.000309071225893999970.00069012959590609430.00045562567119882520.00072432539733242590.000221979378531335070.0003333494929961140.0003718848061545851.29462965006e-055.94971997835e-050.00.03.8838889501836537e-058.556048739841659e-059.293111195205908e-050.00.0001441443027440.00.000123268430966874680.00015716318698842070.00015200129027764210.00.00.00.00.00.00.00.00.00.00.00.00.00.00.00.00.00.00.06.74703707124e-054.68517758785e-057.575154975617997e-056.438715295615e-056.227240942473232e-056.737562094067435e-057.317970669476548e-050.00.00.00.00.00.00.00.00.06.90169304906e-052.23447028336e-050.00.000105253884741103640.000101796906730840950.00.06.703410850077454e-050.0001654410459930.000199967662174000040.000185746950772002940.000157880827111655480.000152695360096261460.00022027828581517280.00017944065066250710.000200184050043408854.108947698900001e-050.00.000123268430966874680.00.00.00.00.00.00.00.00.00.00.00.00.00.00.00.00.00.00.00.00.00.00.0002531507415060.00.00.00.00032595232833461380.000236021036006540920.00019747886017795740.0003419680856150.0005687217395360.000347680189906569640.000344773942966051150.00033345012397089580.00051539470719575690.00050381413455242380.00068695637685836470.000179422568545600020.000204715199242389980.000162009455928694330.00020614271519229920.000170115534515711080.000145437866985609470.000393262318381085137.54454123613111e-050.000179422568545600020.000204715199242389980.000162009455928694330.00020614271519229920.000170115534515711080.000145437866985609470.000393262318381085137.54454123613111e-050.00.00.00.00.00.00.00.00.00.00.00.00.00.00.00.00.00.00.00.00.00.00.00.04.2499026507e-050.02.934962642068444e-054.989307523441927e-054.825437786591812e-050.00.00.00.08.55335897048e-060.00.00.02.566007691144832e-050.00.00.08.27176690561e-060.00.00.02.4815300716832737e-050.00.00.01.23633127353e-050.00.00.00.00.03.70899382060123e-052.85183165612e-050.00.04.349169954396547e-054.2063250139724845e-050.00.00.02.86189487145e-050.03.21315815316498e-052.731113833922003e-052.6414126272576035e-050.00.00.00.00.00.00.00.00.00.00.02.6141117657e-050.02.934962642068444e-052.4946537617209636e-052.412718893295906e-050.00.00.00.00.00.00.00.00.00.00.05.36451591059e-050.000175526760630999987.117862155567566e-056.050026445748478e-052.9256591304533033e-059.496248935732841e-050.000393262318381085133.835547415529881e-050.000114260420760010.00024632604790270.00.00021133997879015810.000131441283489764180.00020980818431762410.00027845546929966470.000250714490090350160.000114260420760010.00024632604790270.00.00021133997879015810.000131441283489764180.00020980818431762410.00027845546929966470.000250714490090350162.00460315351e-050.00.03.057108853886167e-052.9567006066384838e-050.00.00.00.00.00.00.00.00.00.00.09.53292008201e-060.00.02.8598760246031892e-050.00.00.00.00.03.0975731542e-050.00.00.02.8178121842011e-053.0605531538231354e-053.41435412457216e-050.00.00.00.00.00.00.00.00.01.14346131871e-050.00.00.00.00.03.4303839561429214e-051.43349507203e-050.00.04.300485216101064e-050.00.00.00.02.35431585319e-050.00.03.5904362551871805e-053.472511304369809e-050.00.00.00.00.00.00.00.00.00.00.00.04.67488781786e-050.00.00.00.09.002864260043312e-055.021799193528813e-051.28487183714e-050.00.03.8546155114216895e-050.00.00.00.03.39546415193e-050.0001571668249950.03.471476017816521e-056.714916437968124e-050.00018163006247561310.000157821295161000260.000132049117347911220.00508446844111909960.00373217769056420.0035000436553434180.0060171157701603830.0057362458978630990.00370265677448902360.0032559774717684470.0042378988254187150.00508446844111909960.00373217769056420.0035000436553434180.0060171157701603830.0057362458978630990.00370265677448902360.0032559774717684470.0042378988254187150.000526736639590.0002514270349440.000471511490421238160.00052728148028956730.00058141694805884180.000154618412158727050.000251907067276211940.000347755625395921841.93282708152e-050.00.02.9476471557930572e-052.8508340887537306e-050.00.00.00.00.00.00.00.00.00.00.07.74179253687e-053.12252898572e-058.692004747664241e-057.388013063558238e-057.145359799376338e-050.00.09.367586957159516e-051.45801369961e-054.27664938709e-054.3740410988245855e-050.00.03.890398757542165e-054.2255379026977475e-054.714011501022209e-054.03907505197e-050.03.467909822597497e-052.9476471557930572e-055.701668177507461e-050.00.00.00.0007438582822050.001093938129690.00067628565617736730.00080475912871876260.00075053006172002820.0010225636634537260.00094732134028061760.00131192938532189150.00.00.00.00.00.00.00.02.95176686898e-050.04.86004566536065e-050.03.9952549415867686e-050.00.00.08.10587150968e-050.000167172027767000030.00.000123618141163472820.000119558004126984080.000120602361483807080.000140499135264700140.000240414586552132620.00100960136707000025.81474258771e-050.00068651556033669690.00154166786468755750.00080062067619782675.289577258061714e-055.745248902790798e-056.409401602267037e-054.95613942686e-050.00.00.000100220003296963914.846417950881342e-050.00.00.00.00.00.00.00.00.00.00.00.000330490839510.0002328603418090.000290770722117533670.000277070685072105360.00042363111134078580.000277478582669036830.000234378613937543660.000186723828819075763.71558709568e-050.00.00.00.000111467612870270840.00.00.00.0001351844615850.000134625285680.000103114276854419890.000175289739606857040.000127149368293084650.000137569233631719129.961344105219026e-050.000166693182355538180.000191003749235000020.06.191565025733431e-050.000157880827111655480.00035321477033452870.00.00.00.000157644801164999980.00055323187114.359976657992352e-050.000185829822320477380.000243504814594069250.00047905055222758270.0005110289377751080.00066961612329793972.87131503319e-050.00.05.8061966645596215e-052.807748435019417e-050.00.00.08.16010625061e-051.30944763917e-050.00.000154909951332672728.989323618570229e-050.00.03.9283429175185085e-050.00.00.00.00.00.00.00.06.6784184511e-053.02422582784e-054.612084151821842e-057.840340393980171e-057.582830807501421e-054.102121138905003e-050.04.970556344615254e-051.91325414904e-052.12315642589e-050.02.917797564341988e-052.8219648827916675e-053.0532243413622055e-053.3162449362944355e-050.04.86717753573e-052.23639143527e-050.09.028151963668166e-055.573380643513542e-053.216062972901522e-053.493111332896805e-050.00.000344235725050000052.91310051893e-050.000294772334920787230.000334066677656546430.00040386816257344528.739301556797616e-050.00.03.05751806519e-050.0001435549890283.4327917484446115e-052.917797564341988e-052.8219648827916675e-050.000183193460481732319.948734808883306e-050.000147984158513102260.0001915299514750.000107950528590000016.762856561773673e-050.000201189782179690650.000305771506626678150.000120301607465144240.000130665012452498967.288496585121619e-057.78883847668e-050.0001967277478390.00.00018816816945552414.549698484500852e-050.000364071917530929370.000174655566644840285.145575934214383e-050.000184585712797999980.000189508734755999980.000125158520512408350.000232150447175261420.00019644817070521760.000242925487647213420.000153904644081657730.000171696072539893980.000259981249872999950.00.000205967504906676680.00029177975643419880.000282196488279166740.00.00.00.0003072386492360.0004129785712850.00017841483429416070.000303297378398706640.000440003735014227170.000317374635483702870.000344714934167447860.00057684614420403340.03.63808028609e-050.00.00.00.000109142408582630860.00.00.03.63808028609e-050.00.00.00.000109142408582630860.00.00.03.63808028609e-050.00.00.00.000109142408582630860.00.00.00376310226930010040.00267788370401650030.0046372077025305970.0029958096656794050.00365628943968740850.00251318546229629870.00264491804971723080.00287554760003896730.00376310226930010040.00267788370401650030.0046372077025305970.0029958096656794050.00365628943968740850.00251318546229629870.00264491804971723080.00287554760003896732.27950128569e-050.00.00.06.838503857071832e-050.00.00.00.00.00.00.00.00.00.00.04.92946790103e-050.05.534500982186211e-054.704204236388103e-054.549698484500852e-050.00.00.06.09959411996e-050.06.848246164826371e-055.820858777348915e-055.629677417690448e-050.00.00.04.85027966165e-055.32434261043e-055.445593335886029e-054.6286346904220285e-054.476610958645416e-054.843468332683015e-055.260709838700008e-055.868849659907167e-050.00.00.00.00.00.00.00.04.92946790103e-050.05.534500982186211e-054.704204236388103e-054.549698484500852e-050.00.00.00.03.62907099341e-050.00.00.04.922545366686005e-050.05.9646676135383045e-050.00.00.00.00.00.00.00.00.000269375313127000050.00.00056976777642916769.725991881139959e-050.000141098244139583340.00.00.00.000340945497679000060.000139553822104999990.00042819560230598570.000242637902718965280.00035200298801138170.000126949854193481150.000137885973666979150.00015382563845440890.000266582899420.00.000334803145835955830.000189716878669149820.00027522867375375520.00.00.02.52761026917e-050.0001506723607740.00.07.582830807501421e-050.000164084845556200098.910998298206135e-050.000198822253784610160.000370964767210.000214293686149000030.0005346479483296090.000230855016742186380.000347391336558663870.000199342746254226580.00026238001897232090.000181158293221101430.000148309637927000020.09.970240739967804e-050.000175495252415277580.000169731253965268850.00.00.00.000336244198790.0007662685433180.00039548621601872290.000288132509478771260.00032511387087162340.00080401574322538070.00076411810407117630.00073067178265844240.00.00.00.00.00.00.00.00.0001612262028830.00.000112062209969886089.525041635661859e-050.000276365982322985640.00.00.03.31748847828e-050.00.00.09.952465434845612e-050.00.00.00.00.000101787997663999990.00.00.05.457120429131543e-050.000118544502247629160.000132248286454016710.000363576863593000070.00057540937758300010.00046043038379059960.00034412087249081160.000286179334497872560.00052040895295541280.0005093083300325750.00069651084975991220.0006886347249218.23551507761e-050.00087194837980953010.0006334238776126520.00056053191734117595.6356243684022e-050.00012242212615292546.82870824914432e-050.00.00.00.00.00.00.00.00.00052790806758199990.0005580086296090.00059653521799061380.00050033800097810120.0004868509837775340.000489795735142570.00058854191320456330.00059568824048057750.000128465296907066.96042639616e-056.98944711667846e-050.00019542798317818840.000120073436377595030.00014357423986167510.06.52385520230752e-050.000128465296907066.96042639616e-056.98944711667846e-050.00019542798317818840.000120073436377595030.00014357423986167510.06.52385520230752e-050.00.00.00.00.00.00.00.09.46247978584e-060.00.02.838743935751441e-050.00.00.00.08.93169974922e-060.00.00.02.679509924766127e-050.00.00.00.0001100711173720.06.98944711667846e-050.0001670405438206749.327833712993376e-050.00.00.00.02.09379099798e-050.00.00.06.281372993948285e-050.00.00.04.86663539818e-050.00.00.08.076050992219225e-050.06.52385520230752e-053.0846342403e-050.00.04.704204236388103e-054.549698484500852e-050.00.00.03.0846342403e-050.00.04.704204236388103e-054.549698484500852e-050.00.00.03.0846342403e-050.00.04.704204236388103e-054.549698484500852e-050.00.00.00.00271865698154259970.00362948844235270.00293030172847619270.00159000478179036130.0036356644343635030.00266658628582848270.0041089955803107060.0041128834609296720.00271865698154259970.00362948844235270.00293030172847619270.00159000478179036130.0036356644343635030.00266658628582848270.0041089955803107060.0041128834609296727.74179253687e-052.79896741418e-058.692004747664241e-057.388013063558238e-057.145359799376338e-050.08.396902242540398e-050.00.0001250536573650.08.692004747664241e-057.388013063558238e-050.00021436079398129010.00.00.00.00.0002011946885750.00.00.00.000154618412158727050.000167938044850807950.000281027608714785550.00.00.00.00.00.00.00.00.000638814630.001005973442870.00060844033233649680.00066492117572024150.00064308238194387040.00077309206079363530.00083969022425403960.00140513804357392750.0001830003556822.79896741418e-050.00026076014242992727.388013063558238e-050.00021436079398129010.08.396902242540398e-050.00.0003108267755680.0001295587499070.000272525925160410330.000107713087655615420.0005522413138884890.000129679958584738820.00012242212615292540.00013657416498288647.37717077786e-050.000106652418617999998.370078645898896e-050.00.00013761433687687760.000148891804300996428.08590586318705e-059.020639292079534e-050.05.9214963999e-050.00.00.00.08.396902242540398e-059.367586957159516e-050.0003726901458840.000141584367514000020.000455017698199872970.000154702018512091980.00050835072094147968.094118220389738e-050.000245735170988949.807674935012648e-053.89066711589e-054.83640543657e-050.00.00.00011672001347672340.06.858202878724094e-057.651013430978455e-058.52800465809e-050.00.000255840139742570070.00.00.00.00.04.29547641119e-050.0003085362014440.00.00.000128864292335573250.00020913704303550360.00037858865602205260.00033788290527558020.0001504638957180.0001964090829630.000200881887501573628.537259540111742e-050.000165137204252253150.000178670165161195670.00019406174071648920.000216495343009908840.0002604182810510.00.000347680189906569640.000147760261271164760.00028581439197505350.00.00.02.38178659979e-058.72046381408e-050.00.07.145359799376338e-050.00.000167938044850807959.367586957159516e-054.84445762098e-059.04402538562e-050.07.388013063558238e-057.145359799376338e-050.08.396902242540398e-050.000187351739143190350.08.39744357418e-050.00.00.00.00.000167938044850807958.398526237453359e-050.000100428711430.000259509372823999970.000113945608456774920.00.0001873405258323880.00020269304451060010.000330231113404109840.00024560396055745962.38178659979e-055.37594095016e-050.00.07.145359799376338e-057.730920607936352e-058.396902242540398e-050.00.000162549105639999980.0008011330137490.00015766892332972340.00013401512068780060.000195963272903161660.00071155340899982490.00092516621467359370.0007666794175735040.00219537239358580.00281633303750050.00238167738357853220.00158706972845561570.00261737006872450820.0035256585414978380.00274670151479923350.00217663905620450480.00219537239358580.00281633303750050.00238167738357853220.00158706972845561570.00261737006872450820.0035256585414978380.00274670151479923350.00217663905620450488.94607137593e-050.000199484988694000030.000100440943750786788.537259540111742e-058.256860212612657e-058.933508258059785e-050.00029181672204755650.00021730316145397573.47680189907e-050.00.000104304056971970880.00.00.00.00.09.81174771788e-050.00.000208608113943941760.08.574431759251606e-050.00.00.00.0001562509686316.45112914019e-050.000208608113943941768.865615676269886e-050.000171488635185032129.277104729523625e-050.000100762826910484770.00.0002629243378370.0002880689143030.000255840139742570070.000217458497719827370.000315474376047936440.000341327438161718160.00024715410374269850.000275725201003185770.04.10212113891e-050.00.00.00.000123063634167150080.00.00.03.09236824317e-050.00.00.09.277104729523625e-050.00.00.0003604376920793.35876089702e-050.00022757248793884560.000390409475780466550.000463331112518104950.00.000100762826910484770.00.00.00.00.00.00.00.00.00.0001783733829870.0004519839834370.00020238100606501810.00.000332739142896330860.00054001057380809140.00048877490665533650.00032716646984706370.000158343147147000010.0002462524590010.000109351027470614640.000185891941599207250.000179786472371404580.00029177990681566230.000211276895134887450.00023570057505111040.000121482949640.0004013798915250.000104304056971970888.865615676269886e-050.000171488635185032120.000463855236476181140.000403051307641939130.000337233130457742630.000116029287962000010.0002967343257560.000131645897149089420.00.000216441966738390.000463855236476181140.000201525653820969570.000224822086971828438.768554370600001e-050.00.00.000177312313525397738.574431759251606e-050.00.00.00.00.00.00.00.00.00.00.00.06.53671335956e-050.00.00.00.000196101400786678240.00.00.000286121432157000040.0003017191689790.00042045046221259580.000178686827583734130.000259227006675048540.00037396081080250260.00030463180228751210.000226564893847579050.0002453774415110.0003952983780170.00030817107741718670.000174625763320467440.000253335483796070140.00045682712683260260.000396944469647364330.000332123537572019270.00471673673207970.00672409367148660.0051249844323354590.0046625477603260510.004362678003576180.0065833548500784720.0064980333789371930.0070908927854430820.00471673673207970.00672409367148660.0051249844323354590.0046625477603260510.004362678003576180.0065833548500784720.0064980333789371930.0070908927854430823.83050216049e-050.0008033834976740.00.00.000114915064814712230.0004973293257064210.00103448006308799570.00087834110422664330.000393642229374000030.000219134100825999950.00033618662990965820.000476252081783092970.00036848797643064750.000199342746254226580.000216515165262198670.00024154439096146856.19205230411e-050.0001363064535160.000101951333882377540.08.381023524080516e-059.067846728105797e-059.848998119069941e-050.0002197509120777273.39837779608e-050.00.000101951333882377540.00.00.00.00.05.68222096897e-053.02261557604e-050.08.665639382820188e-058.381023524080516e-059.067846728105797e-050.00.00.0001816119753010.0003557010577490.000203902667764755090.000173312787656403760.000167620470481610320.000453392336405289850.000393959924762797630.0002197509120777278.63569748619e-050.000368667145710.00.000174625763320467448.444516126535672e-050.000365461701466082140.00029770835223552320.00044283138342935910.0003911606956826.68513077733e-050.000407805335529510170.00034662557531280750.000419051176204025859.067846728105797e-050.00.00010987545603886350.0004540299382529.96813015035e-050.00050975666941188770.00043328196914100940.000419051176204025859.067846728105797e-059.848998119069941e-050.00010987545603886350.0003150416844620.0005506839900480.000433725166177572340.000259969181484605640.00025143070572241550.00029508924945700210.00087555923956816680.00048140348111942730.00.00020625940650.00.00.00.00018412574272336969.999364502567192e-050.00033465883175195830.0004233538846060.00073748050045300010.00037665353906545040.000454162353441990930.000439245761310498850.00095048372745636080.00051618166498717330.00074577610891494780.00.000264406773350.00.00.00.000274096276099561550.00029770835223552320.000221415691714679559.14939117993e-050.0003013093886360.000102723692472395568.731288166023372e-058.444516126535672e-050.000274096276099561550.000297708352235523240.000332123537572019270.0002155957532620.0001261122989810.00030585400164713260.000173312787656403760.000167620470481610320.000181356934562115930.000196979962381398810.05.72526809752e-053.69026152858e-050.08.731288166023372e-058.444516126535672e-050.00.00.000110707845857339779.08059876505e-056.30561494906e-050.000101951333882377548.665639382820188e-058.381023524080516e-059.067846728105797e-059.848998119069941e-050.00.0004251444736430.0001601336130240.00050975666941188770.00034662557531280750.000419051176204025850.000272035401843173879.848998119069941e-050.00010987545603886350.00.00.00.00.00.00.00.00.00051689918082299990.0001299074572640.00061170800329426520.00051993836296921130.000419051176204025850.000181356934562115939.848998119069941e-050.00010987545603886353.39837779608e-050.00.000101951333882377540.00.00.00.00.00.00.0001004364628790.00.00.09.136542536652052e-059.923611741184108e-050.000110707845857339770.000460076971133000040.00093755202382800010.00061170800329426520.00043328196914100940.000335240940963220640.00081610620552952160.00078791984952559530.00120863001642749859.08059876505e-050.000266213910780.000101951333882377548.665639382820188e-058.381023524080516e-050.000272035401843173870.000196979962381398810.00032962636811659050.03.662515201300001e-050.00.00.00.00.00.00010987545603886350.0002984490923470.00072706290844199990.000205447384944791170.00043656440830116860.000253335483796070140.00082228882829868460.00069465282188288740.00066424707514403840.00349807674606879980.00409597501832839950.0036840207476223460.00336755986199644430.00344264962858692460.00358323739075419680.0044797374573382310.0042249502068948890.00349807674606879980.00409597501832839950.0036840207476223460.00336755986199644430.00344264962858692460.00358323739075419680.0044797374573382310.0042249502068948892.54492266827e-050.000120712344287999980.00.07.634768004813073e-058.260435718068978e-050.00017944065066250710.000100092025021704429.43530965431e-053.14068649697e-050.000105933807862157929.004140921211602e-058.708407255489911e-059.422059490922428e-050.00.08.82291214426e-056.327078345100001e-050.000185746950772002947.894041355582774e-050.00.08.972032533125357e-050.000100092025021704420.00.00.00.00.00.00.00.05.1762697868000004e-050.0001517043632450.07.894041355582774e-057.634768004813073e-050.000165208714361379568.972032533125357e-050.000200184050043408850.0001335683690220.09.224168303643685e-050.00015680680787960340.000151656616150028420.00.00.02.63134711853e-050.00.07.894041355582774e-050.00.00.00.00.0002426529705440.000393129051821999960.000185746950772002940.00023682124066748320.000305390720192522860.00033041742872275910.000448601626656267750.00040036810008681770.0001090339941820.09.287347538600148e-050.000157880827111655487.634768004813073e-050.00.00.00.05.94066553214e-050.00.00.00.00.000178219965964122680.00.00.00.00.00.00.00.00.00.0001446361732549.08055691779e-050.00027862042615800447.894041355582774e-057.634768004813073e-058.260435718068978e-058.972032533125357e-050.000100092025021704423.09578251287e-050.09.287347538600148e-050.00.00.00.00.00.0001645768014910.0001540763526290.000185746950772002947.894041355582774e-050.00022904304014439228.260435718068978e-050.00017944065066250710.000200184050043408850.00054722159134500010.0003023234826440.00055724085231600880.00047364248133496640.00061078144038504570.000247813071542069350.00035888130132501420.000300276075065113340.0004242705755020.0009996027062360.00024873011224067060.00063810167624294070.000385979938021105340.00072373760070092860.00129802279099928520.00097704772700784430.05.50695714538e-050.00.00.00.000165208714361379560.00.08.27205229967e-050.0003298582683719.287347538600148e-057.894041355582774e-057.634768004813073e-050.00033041742872275910.00035888130132501420.000300276075065113340.02.99067751104e-050.00.00.00.08.972032533125357e-050.00.00042754411353699990.000315657454760999970.00050859284139953180.000432292740900961430.00034174675831068040.00028714847972335020.00031188494043721480.000347938944123067760.0001654410459930.000270044718149999960.000185746950772002940.000157880827111655480.000152695360096261460.00033041742872275910.00017944065066250710.000300276075065113340.0003677175563896.5826286726e-050.00068530584459151680.000155747302420957440.00026209952215442070.00.00.00019747886017795745.1762697868000004e-052.99067751104e-050.07.894041355582774e-057.634768004813073e-050.08.972032533125357e-050.00.09.08055691779e-050.00.00.08.260435718068978e-058.972032533125357e-050.000100092025021704422.54492266827e-059.08055691779e-050.00.07.634768004813073e-058.260435718068978e-058.972032533125357e-050.000100092025021704420.000294415668412000030.0004516558565060.000185746950772002940.000315761654223310960.00038173840024065360.00049562614308413870.00035888130132501420.00050046012510852220.00.00.00.00.00.00.00.00.00285006725835680.00378713802149919980.00318809879995729340.0021908944794761250.0031712084956369590.0031657516747330720.0040816521475004910.00411401024226714950.00285006725835680.00378713802149919980.00318809879995729340.0021908944794761250.0031712084956369590.0031657516747330720.0040816521475004910.00411401024226714953.44035842192e-050.0003344279841230.00.00.00010321075265765820.000233489420381108020.00036386576384341720.00040592876814357910.0001774470050920.00.000152354240520856320.000129497757069110670.00025048901768600190.00.00.00.0002556878881720.00.000248798668006536040.000211473401452309170.000306791595055791370.00.00.00.00.00.00.00.00.00.00.06.00855540175e-050.06.746033535500603e-055.733980288134752e-055.545652381605515e-050.00.00.09.324179115899999e-052.45302762142e-050.000152354240520856326.474887853455534e-056.262225442150048e-050.07.359082864248887e-050.02.11112903163e-050.00.00.06.333387094901754e-050.00.00.09.58973683769e-050.0001597302783138.168390003829045e-050.000138859040712660866.714916437968124e-050.000145304049980490470.000157821295161000260.000176065489797215040.00.0001620620067340.00.00.00.00.0002298099561116320.00025637606409068150.0006946779894620.00.00091673714943503050.000476305037907828030.00069099178104346920.00.00.00.000114824750677.44809724717e-050.000152354240520856320.000129497757069110676.262225442150048e-056.775413566506016e-057.359082864248887e-058.209795310769015e-053.99525494159e-054.32266528616e-050.00.00.000119857648247603070.000129679958584738820.00.00.0001103064620580.0006449083322027.617712026042816e-050.000129497757069110670.000125244508843000960.00054203308532048140.00073590828642488880.00065678362486152130.00.0001128571420210.00.00.00.0001067277535254930.00023184367253739860.04.09722863904e-050.06.746033535500603e-050.05.545652381605515e-050.00.00.00.0001774470050927.44809724717e-050.000152354240520856320.000129497757069110670.00025048901768600196.775413566506016e-057.359082864248887e-058.209795310769015e-059.80918226906e-050.0002470362689448.422066712022491e-057.158571664068851e-050.000138469084310895480.00022472489717479580.00024408386642912460.00027230004322674870.000181534874110999960.0007823292118537.617712026042816e-050.000142622529745034040.000325804972328076850.00047427894965542110.00085425029464726970.00101845839125485890.0006543850371140.00112706792328999980.0009599665420429180.00050996904332525880.00049321952597464980.0011740052887804230.00104329652641829350.00116390195467716480.00130582012873730020.00258892669854239980.00185636387110829120.00104775457992280460.00101334193518428060.0025423422710683970.0033977583679054280.00182667945664610580.00130582012873730020.00258892669854239980.00185636387110829120.00104775457992280460.00101334193518428060.0025423422710683970.0033977583679054280.00182667945664610585.13618462362e-050.00.000154085538708593340.00.00.00.00.00.04.56827126833e-050.00.00.00.000137048138049780770.00.00.000229391434370.00.00030817107741718670.00.00038000322569410520.00.00.09.35844268689e-050.000249890811730.000154085538708593340.00.000126667741898035070.000137048138049780770.000446562528353284750.000166061768786009630.000232259154764999980.000350927447341999930.00030817107741718670.00026193864498070120.000126667741898035070.000274096276099561550.000446562528353284750.000332123537572019270.00.0006979195486610.00.00.00.00068524069024890390.00074427088058880790.00066424707514403840.09.53007713892e-050.00.00.00.000137048138049780770.00014885417611776160.00.000411722603096999970.000451964082953999960.000462256616125780.00039290796747105170.00038000322569410520.00041114441414934230.000446562528353284750.00049818530635802880.00.0001049719816350.00.00.00.00.00014885417611776160.000166061768786009635.38076484379e-050.00.000161422945313764460.00.00.00.00.00.0001386747278964.96180587059e-050.000154085538708593340.00026193864498070120.00.00.00014885417611776160.05.13618462362e-050.0002362842554620.000154085538708593340.00.00.00041114441414934230.00029770835223552320.00.00.000306367027980000030.00.00.00.00034957206227190460.00056952902166795740.04.36564408301e-050.00.00.000130969322490350570.00.00.00.00.00417313636083130.00402896063070670.0055431747587112030.004028197107810670.00294803721596190430.0044418202584085820.0048231849523142370.0028218766814019320.00417313636083130.00402896063070670.0055431747587112030.004028197107810670.00294803721596190430.0044418202584085820.0048231849523142370.0028218766814019320.000177728744515999970.00.000235817867936629830.000200440006593927829.692835901762684e-050.00.00.00.000117254333567999998.96847998431e-050.000131645897149089420.000111896120185930590.0001082209833691950.00.00012717638347925260.000141878016050182983.72987067286e-050.00.00.000111896120185930590.00.00.00.06.94502260874e-050.0004077988076120.00.00.00020835067826218850.0003381374621041320.00061211063076462720.00027314832996577280.000132936543199999980.00.000398809629598712160.00.00.00.00.00.0001118961201860.00.00.00033568836055779180.00.00.00.00.00.000121455781412999990.00.00.00.000114859391889340090.000249507952349771850.00.08.39690224254e-050.00.00.00.00.000251907067276211940.00.0004270699200273.49572062272e-050.00058954466984157450.000400880013187855640.00029078507705288050.000104871618681571380.00.00.00.0001271763834790.00.00.00.00.000381529150437757870.00.0003171174660370.0003053530491610.00027119054812712430.000345759011374525460.000334402838610812660.00036180708445142130.00026198334996726040.00029226871306337699.82574449736e-050.000182314519179000020.000294772334920787230.00.00.000262179046703928460.00028476451083397870.00.000440280724770.0001010417817140.00104768480483894260.000138859040712660860.00013429832875936250.000145304049980490470.000157821295161000260.00.00086973993965499990.0006042355976210.00074585635742060030.00105293608357156410.00081042737797403260.00079313369776787940.00073303514111089280.000286537953983702860.001286342259650.00184562122911999990.00156456085457956360.0013298423514404830.00096462357291580570.00208734856414281460.00176334947093348360.00168616565228871348.77639314327e-050.000125352452912000020.00026329179429817890.00.00.000234179342687004040.00.000141878016050182980.00193381536714639980.00249625362976859960.00272368097548746120.0018210186571609290.00125674646878831830.00259899216515125430.0023030668470847110.002586701877071040.00193381536714639980.00249625362976859960.00272368097548746120.0018210186571609290.00125674646878831830.00259899216515125430.0023030668470847110.002586701877071040.05.12752128181e-050.00.00.00.00.00.00015382563845440890.000190309156580000020.00.00057092746974131430.00.00.00.00.00.00.000256728608639999960.00.00.00.00036919090250145030.00040099492341927610.00.0001138680907520.00.00.00011641717554697830.000225187096707617920.00.00.06.79805631044e-050.000107785472357000020.000110240060214278159.370162909878742e-050.09.805070039333912e-050.000106497296734658720.000118808419944462188.66887322575e-050.000139553822104999990.000142731867435328560.00.000117334329337127220.000126949854193481150.000137885973666979150.00015382563845440898.85530060695e-050.000142554979570.000145801369960819530.00.000119857648247603070.000129679958584738820.00014085126342325830.000157133716700740340.000127128382711000020.000185515813327000040.000142731867435328560.000121318951359482640.000117334329337127220.000126949854193481150.00027577194733395830.00015382563845440890.000127128382711000020.000190829034922999960.000142731867435328560.000121318951359482640.000117334329337127220.000126949854193481150.000137885973666979150.00030765127690881780.000176033879196999980.00.00028546373487065710.000242637902718965280.00.00.00.00.000309689231920.00.00050220471875393390.0004268629770055870.00.00.00.00.00.000279107644209999970.00.00.00.00025389970838696230.00027577194733395830.00030765127690881780.0001658225328180.0004143004093740.000141245077149543880.000240110424565642680.000116112096739865480.00037688237963689720.00040934898432384440.000456669864161526450.08.4633236129e-050.00.00.00.00025389970838696230.00.00.0002569616246280.0005695234941650.000288500583113962030.000245219157003209560.000237165133766533750.00051320153822896630.000418058537181798460.00077731040708344920.0002236517843987.44459021505e-050.000251102359376966940.00021343148850279350.00020642150531531640.000223337706451494570.00.01.9036155890999998e-050.0001204357600310.02.9030983322798107e-052.807748435019417e-050.000151892142926709179.898615238057698e-050.000110428984784651241.9036155890999998e-050.0001204357600310.02.9030983322798107e-052.807748435019417e-050.000151892142926709179.898615238057698e-050.000110428984784651241.9036155890999998e-050.0001204357600310.02.9030983322798107e-052.807748435019417e-050.000151892142926709179.898615238057698e-050.000110428984784651240.04.87864711298e-050.00.00.00.00.000146359413389530950.00.04.87864711298e-050.00.00.00.00.000146359413389530950.00.04.87864711298e-050.00.00.00.00.000146359413389530950.00.00.000103985150393999990.00.00.00.000113241653975405716.149843895945079e-050.000137215358245716860.00.000103985150393999990.00.00.00.000113241653975405716.149843895945079e-050.000137215358245716860.00.000103985150393999990.00.00.00.000113241653975405716.149843895945079e-050.000137215358245716860.00.000320118931890999950.00.00.00.00036994589412210760.00032145196315001280.000268958938401880640.00.000320118931890999950.00.00.00.00036994589412210760.00032145196315001280.000268958938401880640.00.000320118931890999950.00.00.00.00036994589412210760.00032145196315001280.000268958938401880640.03.57900751321e-050.00.00.00.00.00010737022539641820.00.03.57900751321e-050.00.00.00.00.00010737022539641820.00.03.57900751321e-050.00.00.00.00.00010737022539641820.00.00.0001466275299020.00.00.00.00.000207923293624809840.000231959296082045230.00.0001466275299020.00.00.00.00.000207923293624809840.000231959296082045230.00.0001466275299020.00.00.00.00.000207923293624809840.000231959296082045230.00.0003290988917350.00.00.00.000459437567557360360.000249507952349771850.000278351155298454250.00.0003290988917350.00.00.00.000459437567557360360.000249507952349771850.000278351155298454250.00.0003290988917350.00.00.00.000459437567557360360.000249507952349771850.000278351155298454253.79141540375e-054.4554991491e-050.00.00.000113742462112521290.00.000133664974473092040.03.79141540375e-054.4554991491e-050.00.00.000113742462112521290.00.000133664974473092040.03.79141540375e-054.4554991491e-050.00.00.000113742462112521290.00.000133664974473092040.00.00.00.00.00.00.00.00.00.00.00.00.00.00.00.00.03.17571546639e-050.00.00.09.527146399168453e-050.00.00.03.17571546639e-050.00.00.09.527146399168453e-050.00.00.03.17571546639e-050.00.00.09.527146399168453e-050.00.00.00.01.0169405465e-050.00.00.00.00.03.0508216394924518e-050.01.0169405465e-050.00.00.00.00.03.0508216394924518e-050.01.0169405465e-050.00.00.00.00.03.0508216394924518e-050.00394179359731380.00211434506175029940.0048001693375360480.0032917863016493040.00373342515275977470.00195126058396160930.0022151994556763030.00217657514561337730.00394179359731380.00211434506175029940.0048001693375360480.0032917863016493040.00373342515275977470.00195126058396160930.0022151994556763030.00217657514561337730.06.55144282542e-050.00.00.00.09.290189715151078e-050.000103641387611126580.001013152727390.000129976599018999970.00132050299578240260.0008192002720474870.00089975491434499660.000118237609297850080.000128423210768264920.000143268976991851435.80634440265e-052.34189673929e-056.51900356074818e-055.5410097976686795e-055.359019849532254e-050.00.07.025690217869639e-051.99993029592e-050.05.999790887768237e-050.00.00.00.00.00.00.0002336143277530.00.00.00.000212515174420805470.00023082233477291670.00025750547406464930.00.00.00.00.00.00.00.06.33455247109e-050.0001004364628790.000102723692472395568.731288166023372e-050.09.136542536652052e-059.923611741184108e-050.000110707845857339770.0004305541348710.00073678556206399980.00064569178125505770.000274411913789305950.00037155870956756960.00068915635133604060.00068614686896187250.00083505346589536264.78804044445e-054.87585341991e-057.88344616648617e-050.06.480675166876213e-057.011765202546924e-057.615795057187803e-050.00.00.00.00.00.00.00.00.00.04.17836283839e-050.00.00.00.00.000125350885151799250.00.0003842790843313.9935561344e-050.00045198424687854050.000329294296547167130.00037155870956756965.7429695944670045e-056.237698808744296e-050.00.00053327870091199991.90525057636e-050.00058021334306433650.00043750091463975430.00058212184503102975.7157517290903846e-050.00.00.0001535062667270.000207549374484000030.00019278948918989880.000109244553356880090.00015848475763545620.000228630069163615380.000186244087654450530.00020777396663273245.47634339763e-050.0001921393202890.08.351666941413661e-058.077363251468903e-050.00017478603113595230.00018984300722265250.000211788922509693440.06.06164443661e-050.00.00.00.000113241653975405710.06.860767912285843e-050.000194054435911000012.07923293625e-050.000258276712502023140.000164647148273583570.00015923944695752980.06.237698808744296e-050.00.000272487098434.1584658724999996e-050.00038741506875303470.000164647148273583570.000265399078262549640.00.000124753976174885920.00.0005335369640630.00.00050069296463366320.00050190451179525080.00059801341575996530.00.00.00.00.00.00.00.00.00.00.01.83816592969e-050.00.05.5144977890673926e-050.00.00.00.09.46601099363e-050.0001523863574710.00015585663685466910.00.000128123692954334330.000138623404004375950.000150565143659345050.000167970524749067186.98503053282e-050.00.00.00020955091598456090.00.00.00.00.000359426229091999970.000417705274929680030.000334878949523753940.000250158433918955560.00049324130383371130.000352950553325533870.000458993054540819550.000441172216922813460.000359426229091999970.000417705274929680030.000334878949523753940.000250158433918955560.00049324130383371130.000352950553325533870.000458993054540819550.000441172216922813460.00.00.00.00.00.00.00.00.00.00.00.00.00.00.00.00.09.78141609985e-060.00.00.00.00.02.9344248299535835e-053.0834747681999995e-050.00.00.09.250424304586792e-050.00.00.07.34790354133e-050.07.450289783712206e-054.2625017125516885e-050.000103309191277236320.00.00.01.67568822366e-056.84387016633e-060.02.555498975421475e-052.471565695571416e-050.02.0531610499001603e-050.02.81072681381e-050.04.627825053363896e-050.03.804355388063852e-050.00.00.00.00.00.00.00.00.00.00.00.01.7234205940000003e-050.00.00.00.02.443874533276683e-052.726387248725531e-050.02.31025881805e-050.00.00.00.06.930776454160328e-050.00.00.00.00.00.00.00.00.00.0002102482956220.000360743194543000050.000214097801152992880.000181978427039223950.000234668658674254430.000352950553325533870.000344714934167447860.00038456409613602230.00.00.00.00.00.00.00.00.00.00.00.00.00.00.00.00.00.00.00.00.00.00.00.00.00.00.00.00.00.00.00.00.00526139328272290.00457010296290580.005407015602118080.0051288433313258590.0052483209147327260.0047665059055853960.0044147288193785290.0045290741637434120.00526139328272290.00457010296290580.005407015602118080.0051288433313258590.0052483209147327260.0047665059055853960.0044147288193785290.0045290741637434120.0001176350294560.0004840989702020.000132073318893080010.000112259419277443350.000108572350198315760.000441019959814173360.00052433754770893190.00048693940308384030.00210114646276999980.000213094279567000020.00214992506811695180.00204554249600464970.00210797182419139760.0002134555070509860.00023184367253739860.00019398365911286081.46273219055e-050.04.388196571636316e-050.00.00.00.00.02.97473936027e-050.0005002299305928.924218080823314e-050.00.00.00042794386332963810.00050706454832372970.00056568138012266526.900941599699999e-050.00.000132936543199570723.766438032402239e-053.642732446740878e-050.00.00.00.00.000100445081961000020.00.00.00.000119015488306388558.617873354186196e-059.614102403400555e-050.0001360341219050.04.359976657992352e-050.000185294218314322370.000179208380820371180.00.00.01.24732688086e-050.00050399261066600010.03.741980642581445e-050.00.000469879330456391230.000425297646050747340.00061680085549089290.00.00.00.00.00.00.00.00.02.83104134939e-050.00.00.08.493124048155428e-050.00.00.000462243770319000035.66688175759e-050.00060899930370290620.0003325793662051940.00044515264104960863.877889436778363e-058.42390192820773e-054.698853907771333e-052.70649249628e-057.93869047902e-058.119477488836057e-050.00.07.221698292443538e-057.84381287327127e-058.750560271358593e-050.00.00.00.00.00.00.00.00.0005442725302390.000229926964402000030.00.00076637197216309770.00086644561855321970.00039541757863543310.00029436331456995550.00.00.0001125861099430.00.00.00.000116711962726264920.000126766137080932469.428023002044418e-055.19405765548e-050.0001781205341870.07.921168645464502e-057.661004320980815e-050.00024866466285321050.000135042963900649690.00015065397580586441.44866745794e-050.04.3460023738321205e-050.00.00.00.00.00.0004829955432280.00.00046490910583750860.00055749069980243140.00042658682404471280.00.00.00.01.31805859545e-050.00.00.03.954175786354331e-050.00.01.45801369961e-055.57344897293e-054.3740410988245855e-050.00.07.780797515084329e-054.2255379026977475e-054.714011501022209e-050.000155333715208999970.0001845557606620.000174399066319694060.000148235374651457920.000143366704656296930.000155115577471134520.000210597548205193240.00018795415631085330.001027802396190.001829771509180.0013986540733289210.00082677391170278040.00085797920354158620.00186600512415361450.00166830418041736140.00195500522296046440.00252065348301880.00325586311998890020.00100937571275604570.00369577955832343060.0028568051779747470.00308467140079707150.00294669489628705640.00373622306287242030.00252065348301880.00325586311998890020.00100937571275604570.00369577955832343060.0028568051779747470.00308467140079707150.00294669489628705640.00373622306287242030.000142118062514999980.00088516763453899990.00.000332156204836519079.419798270727114e-050.00088807227052663670.00081312859471130990.00095430203837924040.0008119854866278.34671102227e-050.000178850858043785530.00132631213477721960.00093079346705927360.000116524020757301516.328100240755082e-057.059630750323114e-050.00.0001231114290360.00.00.00.00011213321610864320.000121573563168202320.000135627507832616270.00.00.00.00.00.00.00.00.00.00.00.00.00.00.00.00.03.0161889893000002e-050.00.00.00.00.09.048566967906407e-050.03.49572062272e-050.00.00.00.000104871618681571380.00.00.00.00.00.00.00.00.00.08.55214422685e-050.000262637001353.7354069989962016e-050.000127088262707864039.212199410766188e-050.000332880359421767870.00025329881902911920.000201731825600416151.29915632716e-050.00.00.03.8974689814780014e-050.00.00.00.01.51749072203e-050.00.00.00.00.04.552472166096214e-050.000243937520842.41145802858e-056.712637329879314e-050.00030587822886786080.00035880796035206410.00.07.234374085727152e-050.0005244419507590.00.0003822033636930670.00060550490164570980.00058561758693761850.00.00.06.11106783455e-050.000314004895242999960.09.319649902278316e-059.013553601369612e-050.000195044250378124680.00035307729106099790.000393893144290265370.000117131971901000021.45932612699e-056.42631630632996e-050.000163866830035320160.000123265922605354830.02.069378751716117e-052.3085996292525825e-056.09578646084e-055.7871874283999996e-050.00.00012326524469680055.960834912848708e-056.449324143519095e-057.004902405541721e-053.907335736141403e-054.19852993818e-050.0002137522615680.06.402944655083806e-056.192645159459492e-050.00024438608022498080.00018193288192170860.000214937822556600120.000418471642500999950.001196849068850.00027957788466713840.00055448180518251530.00042135523765394480.00102626634326285420.00106965993241558930.0014946209308588131.371660081724e-053.00971759955e-050.02.0507651920197244e-052.0642150531531643e-050.05.796091813434965e-053.2330609852143466e-051.371660081724e-053.00971759955e-050.02.0507651920197244e-052.0642150531531643e-050.05.796091813434965e-053.2330609852143466e-050.00.00.00.00.00.00.00.00.03.00971759955e-050.00.00.00.05.796091813434965e-053.2330609852143466e-050.00.00.00.00.00.00.00.06.8807168438399994e-060.00.00.02.0642150531531643e-050.00.00.06.8358839734e-060.00.02.0507651920197244e-050.00.00.00.00.0001391148014790.00.000109942114105590910.000186896762905148920.000120505527427319850.00.00.00.0001391148014790.00.000109942114105590910.000186896762905148920.000120505527427319850.00.00.00.0001391148014790.00.000109942114105590910.000186896762905148920.000120505527427319850.00.00.00.00188639446941620.00274119872014980.00240617101800375160.00174854006120838450.0015044723290373840.00314776232920290730.0029617732770917660.0021140605541554840.00188639446941620.00274119872014980.00240617101800375160.00174854006120838450.0015044723290373840.00314776232920290730.0029617732770917660.0021140605541554840.000135007042039999986.59029297726e-050.000222287334530429750.00.0001827337915906080.000197708789317716520.00.06.85377808347e-050.00.000111143667265214879.44696752389414e-050.00.00.00.00.00.00.00.00.00.00.00.06.29797834926e-050.00049070775192599990.00.00018893935047788280.00.00048789427041307470.00074466446645902950.000239564518904407323.04556319318e-050.00.00.09.1366895795304e-050.00.00.00.06.87415400184e-050.00.00.09.885439465885827e-050.00010737022539641820.03.14898917463e-050.0001774104998540.09.44696752389414e-050.00.000197708789317716520.00021474045079283640.000119782259452203693.70478890884e-050.00.000111143667265214870.00.00.00.00.06.75035210202e-057.28788847037e-050.000111143667265214870.09.1366895795304e-059.885439465885827e-050.00.000119782259452203690.03.29514648863e-050.00.00.09.885439465885827e-050.00.00.0003655180191340.000108668959836000010.00044457466906085950.00037787870095576560.0002741006873859129.885439465885827e-050.00010737022539641820.000119782259452203690.000155112512395000020.0002478058523360.000253449110399181540.000107713087655615420.000104175339131094260.0002254249747360880.000244844252305850830.00027314832996577287.14667458277e-050.000113312932477999980.00011589339663552329.850684084744318e-050.00.000103078941439151360.000111958696567205330.000124901159428793610.04.14441104755e-050.00.00.00.000124332331426605240.00.09.89934127665e-050.000108668959836000010.000111143667265214879.44696752389414e-059.1366895795304e-059.885439465885827e-050.00010737022539641820.000119782259452203690.00.00.00.00.00.00.00.00.03.29514648863e-050.00.00.09.885439465885827e-050.00.00.0001304833045130.000286079459690.000111143667265214870.00018893935047788289.1366895795304e-050.00029656318397657480.000322110676189254560.000239564518904407320.0001312738734510.0001441044902170.000147386167460393620.000125275004121204920.000121160448772033540.000131089523351964230.000142382255416989350.000158841691882270070.0005005250611750.00074956941923399990.00066686200359128920.00037787870095576560.000456834478976519940.00079083515727086620.00085896180317134560.00059891129726101820.00223229718144079970.00266574779707540.0019380536572479880.00194915577072558750.00280968211634907580.00288758546685182830.00214414520309178180.00296551272128437860.00223229718144079970.00266574779707540.0019380536572479880.00194915577072558750.00280968211634907580.00288758546685182830.00214414520309178180.00296551272128437860.0001051971780626.73756209407e-050.00.00012877430591230.000186817228274196950.000202126862822023040.00.00.00.000406624061243000050.00.00.00.00034457817566802030.00037426192852465770.00050103207953721767.01054168995e-050.00.00.00.000210316250698624240.00.00.00.0005791426767360.0001937716063730.00052233573946304660.00065006953666624830.00056502275408015050.000266748080268377867.485238570493154e-050.000239714353145086722.09920174897e-050.00.00.06.297605246907958e-050.00.00.02.15829595115e-050.00.06.474887853455534e-050.00.00.00.00.09.58453472257e-050.00.00.00.00013783127026720810.000149704771409863090.00.0002063166250010.00.000232449041251820860.000131717718618866860.00025478311513204770.00.00.03.9738899418999996e-050.000118442978790000020.00.00.000119216698256974160.00012898648287038197.004902405541721e-050.000156293429445656120.0001757616213340.000121701545897999990.000148848947468271220.000192377194298608160.000186058722234587440.000206746905400812167.485238570493154e-058.350534658953627e-051.98694497095e-050.00.00.05.960834912848708e-050.00.00.00.000118403885857999990.0001102703308030.000132936543199570720.000112993140972067160.000109281973402226355.911880464892504e-050.000128423210768264920.000143268976991851430.0001706496885680.000210381231510999980.000242402859675713967.027622182409055e-050.00019926998420544540.000215600119614899050.000154300060609874820.00026124351430969810.0001273778642310.0002768947665750.00.000130229382815263850.00025190420987631830.000204410782175944240.000296026384143797140.00033024713340494570.000136576239340999970.0005610239598240.00015496602750121396.474887853455534e-050.00019001381198752430.00048240944593522840.000449114314229589260.00075154811930582640.00035013400501599993.16541631191e-050.0004270717293340540.00027225119005868150.000351079095654396379.496248935732841e-050.00.00.04.41766891882e-050.00.00.00.000132530067564623170.00.00.00.00.00.00.00.00.00.09.04486542646e-050.0003518277064427.704276935429667e-050.000130969322490350576.333387094901754e-050.000342620345124451940.00029770835223552320.000415154421965024060.07.57577891427e-050.00.00.06.891563513360405e-057.485238570493154e-058.350534658953627e-050.00.00.00.00.00.00.00.00.00449674051260589950.0064928780702290990.0050591043333775060.0050204643638747140.00341065284055967360.0073598268317667470.00692778549461341150.0051910218843198560.00449674051260589950.0064928780702290990.0050591043333775060.0050204643638747140.00341065284055967360.0073598268317667470.00692778549461341150.0051910218843198563.65882551719e-050.0002989178230250.00.000109764765515722410.00.000229718783778680180.000249507952349771850.00041752673294768130.0006071125309240.0002880689143030.00063960034935642530.00076110474201939570.00042063250139724850.000341327438161718160.00024715410374269850.000275725201003185778.73128816602e-059.13654253665e-050.00.00026193864498070120.00.000274096276099561550.00.07.96343739222e-050.0001262629819040.000129138356251011540.000109764765515722410.00.000114859391889340090.000124753976174885920.000139175577649227138.1180672445e-053.90298904478e-050.000131645897149089420.000111896120185930590.00.000117089671343502020.00.00.0001150209176910.0001262629819040.000129138356251011540.000109764765515722410.000106159631305019880.000114859391889340090.000124753976174885920.000139175577649227130.0005397180446857.98711226881e-050.00064569178125505770.00054882382757861190.000424638525220079530.000114859391889340090.000124753976174885920.00.000193453580209000020.00054663658634300010.000258276712502023140.000109764765515722410.000212319262610039760.000459437567557360360.00062376988087442950.00055670231059690850.0003439776500750.00037759778550699990.000386196782373308230.000328258779891358470.000317477387959351860.00034349459649925290.000373085004221121060.000416213755800047130.00.000246660820065999970.00.00.00.000354712827893550240.000385269632304794660.00.00.00.00.00.00.00.00.00.000204260201850999970.001040580816450.00039114021364489090.000221640391906747180.00.0011596380911904530.00125953533638105950.00070256902178696373.53865437683e-050.00045385286791000010.00.00.000106159631305019880.000459437567557360360.00062376988087442950.000278351155298454250.000117605105910.0003115847440420.00.00035281531772910760.00.00036919090250145030.000267329948946184070.000298233380676915240.0002730879541320.000378788945713000060.00038741506875303470.000219529531031444830.000212319262610039760.00034457817566802030.00037426192852465770.00041752673294768130.000204260201850999970.000493541532660.00039114021364489090.000221640391906747180.00.00069578285471427170.00050381413455242380.000281027608714785550.000418239263416000050.0001262629819040.00038741506875303470.00054882382757861190.00031847889391505960.000114859391889340090.000124753976174885920.000139175577649227133.53865437683e-054.1584658724999996e-050.00.00.000106159631305019880.00.000124753976174885920.00.0001504074614590.000210941305084000030.000129138356251011540.000109764765515722410.000212319262610039760.000229718783778680180.000124753976174885920.000278351155298454250.0005050909953950.00079815470643499990.00052658358859635780.000447584480743722340.0005411049168459750.00093671737074801620.00089023468435476810.00056751206420073180.07.80597808957e-050.00.00.00.000234179342687004040.00.00.000469017334271999960.0003388513988560.00052658358859635780.000447584480743722340.000432883933476780.00035126901403050610.000381529150437757870.0002837560321003660.00278960883848440.00332491697657670040.0028213014135297710.00268297694486905940.0028645481570563970.0037849000550719620.00292809261465836740.0032617582599932120.00278960883848440.00332491697657670040.0028213014135297710.00268297694486905940.0028645481570563970.0037849000550719620.00292809261465836740.0032617582599932126.19455236781e-050.000108668959836000010.09.44696752389414e-059.1366895795304e-059.885439465885827e-050.00010737022539641820.000119782259452203690.0001913945683760.000108668959836000010.000111143667265214870.00018893935047788280.0002741006873859129.885439465885827e-050.00010737022539641820.000119782259452203690.0001294490446980.000108668959836000010.000111143667265214879.44696752389414e-050.0001827337915906089.885439465885827e-050.00010737022539641820.000119782259452203690.0001349352750110.000107785472357000020.000220480120428556349.370162909878742e-059.062407550428528e-059.805070039333912e-050.000106497296734658720.000118808419944462180.000421905545473.29514648863e-050.00033343100179564460.00056681805143364830.0003654675831812169.885439465885827e-050.00.00.00.00.00.00.00.00.00.00.00.00.00.00.00.00.00.00.000197986825533000023.29514648863e-050.000222287334530429750.00018893935047788280.0001827337915906089.885439465885827e-050.00.03.14898917463e-050.00.09.44696752389414e-050.00.00.00.00.0001078321103353.58935599654e-050.000121067208985323320.000102904467670989729.952465434845612e-050.000107680679896256320.00.03.70478890884e-050.0001444590349680.000111143667265214870.00.09.885439465885827e-050.00021474045079283640.000119782259452203696.75035210202e-050.00.000111143667265214870.09.1366895795304e-050.00.00.09.89934127665e-050.000181547844540.000111143667265214879.44696752389414e-059.1366895795304e-050.000197708789317716520.00010737022539641820.000239564518904407320.0002612082441890.0003179558778220.00032805308241184390.000185891941599207250.000269679708557106860.000389039875754216360.000211276895134887450.000353550862576665640.03.57900751321e-050.00.00.00.00.00010737022539641820.00.00.00.00.00.00.00.00.09.89934127665e-050.000464788693984000040.000111143667265214879.44696752389414e-059.1366895795304e-050.00059312636795314960.000322110676189254560.000479129037808814632.97246967654e-050.0001771906962710.00.08.91740902962167e-050.00019296377837409130.000104793339986904160.000233814970450701620.06.59029297726e-050.00.00.00.000197708789317716520.00.00.00.00.00.00.00.00.00.09.89934127665e-050.000108668959836000010.000111143667265214879.44696752389414e-059.1366895795304e-059.885439465885827e-050.00010737022539641820.000119782259452203690.03.6085860876999994e-050.00.00.00.00.000108257582631099360.00.000236807771715999980.0002599531980390.000265873086399141440.000225986281944134370.00021856394680445270.000236475218595700150.000256846421536529830.000286537953983702869.89934127665e-050.000141620424722000020.000111143667265214879.44696752389414e-059.1366895795304e-050.000197708789317716520.00010737022539641820.000119782259452203690.0004844042797920.000855364539010.00044096024085711270.0004685081454939370.00054374445302571150.00088245630354005170.00085197837387726970.00083165893961123530.0009302960795990.00137264849898690.00127173709335288730.00054356171194433550.00097558943350018520.00144959968200174950.00149122745702564680.00117711835793297510.0009302960795990.00137264849898690.00127173709335288730.00054356171194433550.00097558943350018520.00144959968200174950.00149122745702564680.00117711835793297510.09.20667576387e-050.00.00.08.375163991931047e-059.096644096085432e-050.000101482192035894770.0001129050260290.00.00018448336607287377.840340393980171e-057.582830807501421e-050.00.00.02.5105318214000002e-050.0001190810963930.00.07.531595464207491e-058.148808208365345e-050.00017701577700490579.87394300889787e-052.5105318214000002e-050.00.00.07.531595464207491e-050.00.00.00.08.95784668917e-050.00.00.08.148808208365345e-058.850788850245284e-059.87394300889787e-050.00.0002669318073820.00.00.00.000242823546611692080.00026374162748381920.000294230248050379370.000347603406746999949.11061361668e-050.000363407468295631870.000231537354373097650.000447865397572721470.00.000175241659150359869.807674935012648e-050.0002699133524880.00.000274855285263977270.00023362095363143620.000301263818568299640.00.00.00.06.24157728638e-050.00.00.00.08.850788850245284e-059.87394300889787e-050.02.71626940279e-050.00.00.08.148808208365345e-050.00.00.0001496636579070.0006243057676230.00044899097372040450.00.00.00087856024921978680.00060724617542080220.00038711087822963840.00.0001267866736620.00.00.00.000220614075885012930.000159745945101988030.00.00.0001267866736620.00.00.00.000220614075885012930.000159745945101988030.00.00.0001267866736620.00.00.00.000220614075885012930.000159745945101988030.03.68751716568e-050.00.000110625514970272120.00.00.00.00.03.68751716568e-050.00.000110625514970272120.00.00.00.00.03.68751716568e-050.00.000110625514970272120.00.00.00.00.03.99525494159e-050.000142554979570.00.00.000119857648247603070.000129679958584738820.00014085126342325830.000157133716700740343.99525494159e-050.000142554979570.00.00.000119857648247603070.000129679958584738820.00014085126342325830.000157133716700740343.99525494159e-050.000142554979570.00.00.000119857648247603070.000129679958584738820.00014085126342325830.000157133716700740346.75035210202e-050.000217337919672000020.000111143667265214870.09.1366895795304e-050.000197708789317716520.00021474045079283640.000239564518904407326.75035210202e-050.000217337919672000020.000111143667265214870.09.1366895795304e-050.000197708789317716520.00021474045079283640.000239564518904407326.75035210202e-050.000217337919672000020.000111143667265214870.09.1366895795304e-050.000197708789317716520.00021474045079283640.000239564518904407320.06.08987940675e-050.00.00.08.260435718068978e-050.00.000100092025021704420.06.08987940675e-050.00.00.08.260435718068978e-050.00.000100092025021704420.06.08987940675e-050.00.00.08.260435718068978e-050.00.000100092025021704426.63619782685e-053.46538822708e-050.000107615296875842959.147063792976864e-050.00.00.000103961646812404920.06.63619782685e-053.46538822708e-050.000107615296875842959.147063792976864e-050.00.00.000103961646812404920.06.63619782685e-053.46538822708e-050.000107615296875842959.147063792976864e-050.00.00.000103961646812404920.00.09.77057575148e-050.00.00.00.000132530067564623170.00.000160587204979877460.09.77057575148e-050.00.00.00.000132530067564623170.00.000160587204979877460.09.77057575148e-050.00.00.00.000132530067564623170.00.000160587204979877460.00.0001004364628790.00.00.09.136542536652052e-059.923611741184108e-050.000110707845857339770.00.0001004364628790.00.00.09.136542536652052e-059.923611741184108e-050.000110707845857339770.00.0001004364628790.00.00.09.136542536652052e-059.923611741184108e-050.000110707845857339770.00.0001170896713440.00.00.00.00035126901403050610.00.00.00.0001170896713440.00.00.00.00035126901403050610.00.00.00.0001170896713440.00.00.00.00035126901403050610.00.00.00.000106916234676999990.00.00.09.725996893855409e-050.000105638447567443730.00011785028752555520.00.000106916234676999990.00.00.09.725996893855409e-050.000105638447567443730.00011785028752555520.00.000106916234676999990.00.00.09.725996893855409e-050.000105638447567443730.00011785028752555520.000279181866510.0001029357152510.0003304477851452120.00028729491497447250.00021980289940988490.000179112418045258040.000129694727706564540.00.000279181866510.0001029357152510.0003304477851452120.00028729491497447250.00021980289940988490.000179112418045258040.000129694727706564540.00.00.0001029357152510.00.00.00.000179112418045258040.000129694727706564540.00.000279181866510.00.0003304477851452120.00028729491497447250.00021980289940988490.00.00.00.04.90605524283e-050.00.00.00.00.000147181657284977750.00.04.90605524283e-050.00.00.00.00.000147181657284977750.00.04.90605524283e-050.00.00.00.00.000147181657284977750.00.04.87864711298e-050.00.00.00.00.000146359413389530950.00.04.87864711298e-050.00.00.00.00.000146359413389530950.00.04.87864711298e-050.00.00.00.00.000146359413389530950.00.00.000186616856239000020.00.00.00.00.00026462964643157620.000295220922286239360.00.000186616856239000020.00.00.00.00.00026462964643157620.000295220922286239360.00.000186616856239000020.00.00.00.00.00026462964643157620.000295220922286239360.00.0002485802456240.00.00.00.000226129427782138340.00024560939059430660.00027400191849691580.00.0002485802456240.00.00.00.000226129427782138340.00024560939059430660.00027400191849691580.00.0002485802456240.00.00.00.000226129427782138340.00024560939059430660.00027400191849691585.86187979801e-050.0001335147278220.00.00013298423514404834.287215879625803e-050.000231927618238090570.00.000168616565228871325.86187979801e-050.0001335147278220.00.00013298423514404834.287215879625803e-050.000231927618238090570.00.000168616565228871325.86187979801e-050.0001335147278220.00.00013298423514404834.287215879625803e-050.000231927618238090570.00.000168616565228871320.00.00013742638787450.00.00.06.347492709674056e-050.000114904978055815970.000233899258471772450.00.00013742638787450.00.00.06.347492709674056e-050.000114904978055815970.000233899258471772450.02.66912066725e-050.00.00.00.00.08.007362001736355e-050.00.000110735181202000010.00.00.06.347492709674056e-050.000114904978055815970.00015382563845440892.60458765631e-050.000571619705390.07.81376296891583e-050.00.00061323234652783270.00075486727956668250.000346759490075192972.60458765631e-050.000571619705390.07.81376296891583e-050.00.00061323234652783270.00075486727956668250.000346759490075192970.00.000119484761125999990.00.00.08.176431287037769e-050.000177615830486278249.90741400214837e-052.60458765631e-050.000452134944264000030.07.81376296891583e-050.00.0005314680336574550.00057725144908040420.000247685350053709260.00.0002245243008530.00.00.00.000266034620920162730.000192634816152397360.00021490346548777720.00.0002245243008530.00.00.00.000266034620920162730.000192634816152397360.00021490346548777720.00.0002245243008530.00.00.00.000266034620920162730.000192634816152397360.00021490346548777720.0001056972571850.00027036088735830.00.00016119301229581610.000155898759259120060.000423493806808773160.000183205139837245030.00020438371542893490.0001056972571850.00027036088735830.00.00016119301229581610.000155898759259120060.000423493806808773160.000183205139837245030.00020438371542893490.02.87148479723e-050.00.00.08.614454391700507e-050.00.00.0001056972571850.000241646039385999980.00.00016119301229581610.000155898759259120060.000337349262891768060.000183205139837245030.00020438371542893490.00.00.00.00.00.00.00.04.76357319958e-050.0003119491232930.00.00.000142907195987526750.00030923682431745410.000251907067276211940.00037470347828638074.76357319958e-050.0003119491232930.00.00.000142907195987526750.00030923682431745410.000251907067276211940.00037470347828638074.76357319958e-050.0003119491232930.00.00.000142907195987526750.00030923682431745410.000251907067276211940.00037470347828638070.03.43038395614e-050.00.00.00.00.00.000102911518684287660.03.43038395614e-050.00.00.00.00.00.000102911518684287660.03.43038395614e-050.00.00.00.00.00.000102911518684287660.00.000174516918386100020.00.00.09.20628713616848e-050.000203955291838076840.000227532591958342060.00.000174516918386100020.00.00.09.20628713616848e-050.000203955291838076840.000227532591958342060.07.33137649511e-050.00.00.00.00.000103961646812404920.000115979648041022610.00.0001012031534350.00.00.09.20628713616848e-059.999364502567192e-050.000111552943917319430.00.000356040449820.00.00.00.000243641134310721380.00052925929286315240.000295220922286239360.00.000356040449820.00.00.00.000243641134310721380.00052925929286315240.000295220922286239360.00.000356040449820.00.00.00.000243641134310721380.00052925929286315240.000295220922286239360.00.000250143643396000030.00.00.00.000227551625441145450.00024715410374269850.000275725201003185770.00.000250143643396000030.00.00.00.000227551625441145450.00024715410374269850.000275725201003185770.00.000250143643396000030.00.00.00.000227551625441145450.00024715410374269850.000275725201003185770.00.0002651522619990.00.00.00.000241204722967614150.00026198334996726040.00029226871306337690.00.0002651522619990.00.00.00.000241204722967614150.00026198334996726040.00029226871306337690.00.0002651522619990.00.00.00.000241204722967614150.00026198334996726040.00029226871306337696.57626034633e-050.000270223937310.00.00.000197287810389859960.0003201832605764790.00023184367253739860.000258644878817147736.57626034633e-050.000270223937310.00.00.000197287810389859960.0003201832605764790.00023184367253739860.000258644878817147736.57626034633e-050.000270223937310.00.00.000197287810389859960.0003201832605764790.00023184367253739860.000258644878817147736.07709911769e-050.00.000182312973530839830.00.00.00.00.06.07709911769e-050.00.000182312973530839830.00.00.00.00.06.07709911769e-050.00.000182312973530839830.00.00.00.00.00.000127350990916100020.000302433769639599945.215202848598544e-050.00011974959303738640.000210151351224923870.000465475156450962240.000105478328662453260.000336347823804170950.000127350990916100020.000302433769639599945.215202848598544e-050.00011974959303738640.000210151351224923870.000465475156450962240.000105478328662453260.000336347823804170952.49279034377e-050.00.04.432807838134943e-053.0455631931767994e-050.00.00.00.01.53663886994e-050.00.00.00.00.04.609916609832444e-050.00.00.00.00.00.00.00.00.00.00.00.00.00.00.00.01.35112258025e-050.00.00.04.053367740737122e-050.00.00.04.64507552212e-051.87351739143e-055.215202848598544e-054.432807838134943e-054.287215879625803e-050.00.05.620552174295711e-051.23852903189e-050.00.00.03.715587095675696e-050.00.00.00.00.00.00.00.00.00.00.00.00.00.00.00.00.00.00.00.00.00.00.00.00.00.00.00.01.32728752527e-050.00.00.00.00.03.981862575795326e-050.00.00.00.00.00.00.00.00.01.19955746322e-050.00.00.00.03.5986723896601703e-050.00.00.00.00.00.00.00.00.00.00.00.00.00.00.00.00.00.00.00.00.00.00.00.00.00.09.458748865799999e-050.00.00.00.000128300384557241580.00.000155462081416689861.97113373776e-050.0001484762684830.00.05.9134012132769676e-050.000337174771893720636.949160476585156e-053.876242878824628e-051.03644787582e-050.00.03.109343627468754e-050.00.00.00.00.001137321642513760.000224583488958420020.00059248409260097820.00181310905035006050.001006371784590140.00020841357976036610.000114756401479168850.00035058048563450350.001137321642513760.000224583488958420020.00059248409260097820.00181310905035006050.001006371784590140.00020841357976036610.000114756401479168850.00035058048563450350.00.00.00.00.00.00.00.00.0001395384194020.06.336227759979539e-050.00026844019799612468.681278260924523e-050.00.00.00.00.00.00.00.00.00.00.00.000297902815610.00.000247302504362115030.000319604916719033550.00032680102574990640.00.00.09.5180779455e-060.00.01.4515491661399054e-051.4038742175097085e-050.00.00.05.41093914514e-050.00.00.000162328174354237340.00.00.00.00.00.00.00.00.00.00.00.00.00.00.00.00.00.00.00.00.00.00.00.00.00.00.00.00.06.48621200762e-060.00.00.00.00.01.945863602286132e-050.00.00.00.00.00.00.00.00.00.00.00.00.00.00.00.00.00.00.00.00.00.00.00.06.89384034143e-050.02.1253177752909425e-050.000150619207138937083.4942825351182085e-050.00.00.00.01.13484003819e-050.00.00.01.6319670024872404e-051.772553112092425e-050.00.0001133174923920.00.000107250499259314680.000182321135941369375.038084197526367e-050.00.00.06.45311929234e-050.000134287098650.08.537259540111742e-050.0001082209833691958.933508258059785e-059.70308703582446e-050.000216495343009908848.30262833633e-050.05.489687209051099e-050.000181500793372454371.2681184626879504e-050.00.00.04.37061068606e-060.00.01.3111832058192093e-050.00.00.00.00.01.62099948231e-050.00.00.04.862998446927705e-050.00.00.00.00.00.00.00.00.00.02.49368261455e-054.61824468174e-050.00.07.48104784364234e-054.047059110194869e-050.09.807674935012648e-050.0001442315315930.07.600631954235546e-050.000206731845365934520.000149956429870319540.00.00.00.00.00.00.00.00.00.00.03.79141540375e-050.00.00.00.000113742462112521290.00.00.00.00.00.00.00.00.00.00.00.00.00.00.00.00.00.00.00.01.00693362784e-050.00.00.01.3658251583670113e-050.01.6549757251606852e-055.56546424014e-050.00.00.00013297989889013073.398402831410697e-050.00.00.00.00.00.00.00.00.00.00.00.00.00.00.00.00.00.00.02.01708695122e-050.02.2412441993977214e-053.810016654264744e-050.00.00.00.01.91609316362e-050.00.05.748279490848304e-050.00.00.00.00.00109542202911120.00172835514285239980.00096819137421937580.00120438298246693260.00111369173064818860.00164230108139371740.00136882090854629790.0021739434386186120.00109542202911120.00172835514285239980.00096819137421937580.00120438298246693260.00111369173064818860.00164230108139371740.00136882090854629790.0021739434386186122.15309227599e-050.00.03.283561361581439e-053.175715466389483e-050.00.00.00.03.58313867567e-050.00.00.03.2595232833461376e-053.540315540098114e-053.9495772035591476e-050.0001789062563690.06.306756933188937e-050.00021442419310048090.000259227006675048540.00.00.04.25766416266e-050.00.06.493126974169493e-056.279865513818077e-050.00.00.04.43246562389e-050.00.06.759706967243901e-056.537689904414713e-050.00.00.00.00.00.00.00.00.00.00.02.6665737279e-050.07.999721183690982e-050.00.00.00.00.00.00.00.00.00.00.00.00.00.000298897474950.001024968637680.000434908100597414960.000369662330145924649.212199410766188e-050.0009424250716359270.00068954065825864120.00144294018314839180.05.02258483026e-050.00.00.07.011765202546924e-053.8078975285939015e-054.248091759642107e-050.07.73038664721e-050.00.00.07.032207666694289e-057.637998541319544e-058.52095373362615e-052.20791195003e-050.00.06.623735850086696e-050.00.00.00.00.000103267120797999990.00.04.4158239000577975e-050.000265643123391986550.00.00.07.63076383723e-050.0001590328863854.4312181066523576e-057.532876064804478e-050.000109281973402226350.000157650145730466820.000128423210768264920.000191025302655801920.00.00.00.00.00.00.00.00.000269533815810000040.0003809925172560.000345906311386638150.000235210211819405080.000227484924225042520.00036919090250145030.00040099492341927610.000372791725846144071.13326454072e-050.00.03.3997936221683925e-050.00.00.00.00.000253528764302270.00085506290090350.00.00039528382819650020.000365302464711272470.00062044582225938170.00103114426898446680.00091359861146600160.000253528764302270.00085506290090350.00.00039528382819650020.000365302464711272470.00062044582225938170.00103114426898446680.00091359861146600160.00.00.00.00.00.00.00.00.00.00.00.00.00.00.00.00.00.00.00.00.00.00.00.00.03.80558220135e-050.00.00.00.00.00.000114167466040381590.00.00.00.00.00.00.00.00.00.00.00.00.00.00.00.00.00.00.00.00.00.00.00.00.00.00.00.00.00.00.00.00.0001921458660520.0004555508624250.00.000235210211819405080.00034122738633756390.00036919090250145030.00040099492341927610.00059646676135383050.00.00.00.00.00.00.00.05.33578721257e-050.000361456216464999970.00.000160073616377095140.00.00025125491975793140.00063014934556519070.000202964384071789548.02502612457e-060.00.00.02.4075078373708608e-050.00.00.00.00067719498786980010.00027327054834050.00037883226423041670.00093598465816204280.00071676804121475040.000141471349562508340.00035965195135312870.00031868834410694210.00067719498786980010.00027327054834050.00037883226423041670.00093598465816204280.00071676804121475040.000141471349562508340.00035965195135312870.00031868834410694211.06159631305e-050.00.00.03.1847889391505954e-050.00.00.00.03.76803140432e-050.00.00.02.020140058355228e-054.388330820222118e-054.8956233343949235e-051.36961382996e-050.00.04.108841489893351e-050.00.00.00.00.00.00.00.00.00.00.00.00.000106415257060.09.10035396399746e-050.000116026513884068970.000112215717654635080.00.00.02.46569458033e-050.00.03.7602937615500326e-053.636789979454188e-050.00.00.00.00.00.00.00.00.00.00.01.28362177976e-056.17950116593e-050.00.03.850865339275235e-054.166442984773025e-056.690513665309384e-057.681546847693548e-050.00.00.00.00.00.00.00.00.0001535000381370.00.000109882718041784560.000224154950647990620.000126462445719918290.00.00.00.00.00.00.00.00.00.00.00.00.00.00.00.00.00.00.08.23571979674e-050.07.906429974551728e-050.000168007294156717940.00.00.00.09.08611946298e-050.0001484828216756.712637329879314e-059.509323745173969e-050.000110363973138882037.960551913122581e-050.000172926303608752750.000192916642286057365.49796651974e-050.03.1755333504347114e-058.097400734766406e-055.2209654740173714e-050.00.00.00.00.00.00.00.00.00.00.00.00.00.00.00.00.00.00.08.34656226662e-050.00.00.000106223966628118440.000144172901370589990.00.00.04.3810747181e-052.5312400963000006e-050.06.681333553130928e-056.461890601175123e-050.07.593720288906097e-050.00.003576616374638330.00168232659401380.0024215589049118740.0044377289913472450.00387056122765490120.0014573352404995860.00159312359547240760.0019965209460681670.003576616374638330.00168232659401380.0024215589049118740.0044377289913472450.00387056122765490120.0014573352404995860.00159312359547240760.0019965209460681670.0001705911770660.05.4675513735307313e-050.00023236492699900910.000224733090464255760.00.00.00.000379125915839999930.00.0002135358646670270.00046963330285122560.000454208580002875370.00.00.09.4304241007e-062.12854514778e-050.00.02.82912723020992e-053.060973641720992e-053.324661801614979e-050.00.0001609584945160.00.000167401572917977920.000177859573752327950.00013761433687687760.00.00.00.00.00.00.00.00.00.00.00.00.00.00.00.00.00.00.00.000145076453630.000148182375210000020.000113468848588754930.000185444486803674350.000136316025496833720.000100922478229127260.000160192400708563770.0001834322466924960.000463293057403000031.01517139296e-050.000273929846593054840.00055298158384814680.00056296774176904483.0455141788840172e-050.00.01.85779354784e-050.00.00.05.573380643513542e-050.00.00.09.604416982630001e-060.00.02.881325094787713e-050.00.00.00.08.787512250100001e-059.74824492645e-059.970240739967804e-050.00.00016392296010333958.867820697338756e-059.631740807619868e-050.000107451732743888584.55743258774e-053.48586203164e-055.116802794851402e-054.349169954396547e-054.2063250139724845e-050.04.94308207485397e-055.514504020063716e-050.0003352948054280.00.0002620198532629220.00047461912130472760.00026924544171563010.00.00.00.0003306523526660.00.00019097925924445370.0004869845230627120.000313993275690903850.00.00.00.0003950390377420.00.00030132283125236030.00051223557240670450.00037155870956756960.00.00.08.39705987636e-050.000102639270932000010.00.000128058893101676120.000123852903189189840.00.00014554630553736690.000162371507257431644.26862977006e-050.000147306812222000010.00.000128058893101676120.00.000134002623870896770.00014554630553736690.000162371507257431640.000199573823435000020.0005416849520440.00011589339663552320.00019701368169488640.00028581439197505350.00051539470719575690.000485154351791222950.00062450579714396770.0004611800745896.07715063931e-050.00044761724932415850.000487180571582463470.00044874240285938348.739301556797616e-059.492150361132625e-050.00.00.00.00.00.00.00.00.00.01.23320132094e-050.00.00.00.00.03.6996039628275565e-050.000100752931332999990.08.581979371111528e-050.000145889878217099377.054912206979167e-050.00.00.00.0001373591295860.00050563142901499994.4024439631026676e-050.000187099032129072250.000180953916997192910.000469879330456391230.000382767881445672640.00066424707514403840.0015415818198270.00156525818880280010.00172817506159441940.00158302271757896060.00131354768030002740.00189542741418577020.00193784119326724530.00086250595895853830.0015415818198270.00156525818880280010.00172817506159441940.00158302271757896060.00131354768030002740.00189542741418577020.00193784119326724530.00086250595895853830.001117832010390.000203853083143000010.00146230197519527790.00101693826874860430.00087425578721781080.000354712827893550240.000256846421536529830.00.00.000335566158207999960.00.00.00.000358224836090516130.00064847363853282290.00.0003121365323640.00.000265873086399141440.000451972563888268630.00021856394680445270.00.00.00.000111613277072999990.0005682855600590.00.000114111884942087650.000220727946277764120.00047763311478735480.00064847363853282290.00057874992685817230.08.30343354678e-050.00.00.00.00011940827869683870.000129694727706564540.00.00.0003745190519250.00.00.00.00058544835671751030.000254352766958505230.0002837560321003660.0048779588844120.00284644202267890020.0050076865965120670.0045362504895651710.0050899395671594660.0031219240738419130.00359725361962925360.00182014837455530860.0048779588844120.00284644202267890020.0050076865965120670.0045362504895651710.0050899395671594660.0031219240738419130.00359725361962925360.00182014837455530860.0001335683690225.70508019201e-059.224168303643685e-050.00015680680787960340.000151656616150028428.204242277810006e-058.910998298206135e-050.00.000174399947400999990.0002180772858450.000195805449911281070.000166430330384849840.000160964061906528340.000217693793292070520.00033102589346043730.000105512170781002516.19156502573e-050.00.000185746950772002940.00.00.00.00.00.0009012833102626.5169987554e-050.00101190503032509030.00086009704322021280.00083184785724082730.00.000195509962662134650.00.0005544903012593.79686014445e-050.00050522990141730460.00049382146875934180.00066441953360127450.00.000113905804333591490.00.000146813641683999970.0002578458729760.000125551179688483470.000160073616377095140.00015481612898648730.00033500655967724190.000303221469869514340.000135309589381192986.14815940495e-050.000111277452012000010.00.00.000184444782148572440.000201411201102692470.00.000132421154933970980.0004803806657290.00.00050427994486448730.000476252081783092970.00046060997053830930.00.00.00.0006318030657890.001389147541640.00058457095706969130.00066636218136421710.00064447605893260320.001406663196953370.00176204384887607280.00099873557908266968.64324209268e-050.0001544670917799.755055688026056e-054.145791503291673e-050.000120288790867198780.00021691072209317820.000141357922644205250.000105132630598337040.00.00.00.00.00.00.00.01.35605368455e-050.00.00.04.0681610536595204e-050.00.00.02.99644120619e-056.7632805502e-050.00.08.989323618570229e-059.725996893855409e-050.000105638447567443730.00.01.43574239862e-050.00.00.04.3072271958502536e-050.00.00.000523611656720.00.00052152028485985440.00062059309733889210.000428721587962580330.00.00.00.00077970757886100010.00.0007827044275213750.00066528156660139060.00089113674245880510.00.00.00.00.0001183203653870.00.00.09.241560266958396e-050.000150565143659345050.000111980349832711454.8308785429999995e-050.05.423810962542486e-054.61012015166034e-054.458704514810835e-050.00.00.08.22373519108e-053.39533112642e-050.000109793744181021984.666113513826255e-059.02571764131748e-054.882686699749275e-055.3033066794991994e-050.00.000109294038472999990.0002026456072420.000106349234559656589.039451277765373e-050.00013113836808267160.000236475218595700150.000256846421536529830.000114615181593481150.01.42692456409e-050.00.00.00.04.280773692275497e-050.03.33131843101e-050.0001042586284865.402202153926778e-054.591753139103925e-050.00.00014414624878542685.218791832017139e-050.0001164417183519432.53923734201e-050.07.617712026042816e-050.00.00.00.00.00.00090868191436470.00091783225012970010.00097277610696910010.00079982360465208390.00095344603147433720.00123007186987877610.00113425626048530950.000389168620024729650.00090868191436470.00091783225012970010.00097277610696910010.00079982360465208390.00095344603147433720.00123007186987877610.00113425626048530950.000389168620024729650.0001259558981450.00.00.000192088339652514160.00018577935478378480.00.00.00.02.29206780374e-050.00.00.00.06.876203411214184e-050.00.000141252390067999980.00.00020038710452743170.000113549757430057650.000109820308246572270.00.00.00.00.000107413190510.00.00.00.00.000152315901143756060.000169923670385684270.000121991882398999980.0001542187116970.000136964923296527420.00011641717554697830.000112593548353808960.000182730850733041040.00013231482321578810.000147610461143119680.08.639216797099999e-050.00.00.05.911880464892504e-050.000128423210768264927.163448849592572e-050.04.54246182613e-050.00.00.00.000136273854783962780.00.00.00.0002020835634280.00.00.00.000290608099960980930.000315642590322000470.05.56552827535e-050.06.248630141177978e-055.311198331405922e-055.136756353468703e-050.00.00.00.0003864183965060.00.00057293777773336110.00032465634870847470.00026166106307575320.00.00.00.00.00.00.00.00.00.00.07.74080644932e-050.0002993793202250.00.00.00023222419347973090.00056134025975186630.00033679770092335810.00.0085678356307361990.0066456737164688990.0111018411282645880.0067156953900921560.007885970373855190.0069132548181336990.0078606783748475930.0051630879564298050.0085678356307361990.0066456737164688990.0111018411282645880.0067156953900921560.007885970373855190.0069132548181336990.0078606783748475930.0051630879564298050.000251240101687000030.00.000255840139742570070.000217458497719827370.000280421667598165650.00.00.00.04.21873349384e-050.00.00.00.00.000126562004815101640.04.13840681631e-056.66211713566e-056.813832867515685e-050.05.601387581420646e-056.0604201750656835e-056.582496230333176e-057.343435001592385e-050.0002382039905650.0002008821396230.000274377764584418040.000223792240371861170.000216441966738390.000312444544058713330.00029020187481073520.00.0004245228457530.0001482296355150.000394937691447268360.0003916364206507570.000486994425161377650.000175634507015253060.00012717638347925260.000141878016050182980.00161409060434999980.00.00181452327890765580.00135336556971935330.00167438296442653350.00.00.00.0004057414421460.00.00039302977989438310.00050110001648481970.00032309453005875610.00.00.00.000425999192991999973.11884940437e-050.00087449126026500230.000164647148273583570.000238859170436294670.09.356548213116442e-050.02.19409828582e-050.06.582294857454473e-050.00.00.00.00.00.000102509132499999990.00.000197468845723634185.594806009296529e-055.41104916845975e-050.00.00.00.00.000100449345317000010.00.00.00.00017478603113595230.000126562004815101640.00.00.00.00.00.00.00.00.00.05.99839895055e-050.00.00.00.08.505952921014947e-059.489243930629124e-050.0004504607378680.000488039957263999950.00061857218132475890.000239429896919584430.00049338013536018080.00041672286757656310.00063321894493963460.00041417805927693164.34600237383e-050.00.00013038007121496360.00.00.00.00.00.0002794361940520.0006460254011740.00042573858628273240.000259874635776707270.000152695360096261460.00070908165013512470.000770165624524920.000458828928860556660.0003067547356310.0004095145460960.00038376020961385510.00032618774657974110.000210316250698624240.00045673639651942830.0004960820407658470.000275725201003185770.000375157056160.00.00061634215483437347.483961285162891e-050.000434289400793263140.00.00.00.000213364585332000030.0003789443452150.000259512486246051930.00022057991156269570.000160001358186991680.00040393135425198550.000313377212879498150.00041952446851202430.000139195316425999980.00.000197468845723634180.000111896120185930590.0001082209833691950.00.00.00.0001830772821430.0001026180821870.000329114742872723660.000111896120185930590.0001082209833691950.000117089671343502020.000190764575218878940.00.000107772979391999990.0003498799928670.00017609775852410677.483961285162891e-057.238156679887718e-050.000234939665228195620.000340238116840597940.000474462196531455970.00.0001221330274110.00.00.00.000175634507015253060.000190764575218878940.00.00.00.00.00.00.00.00.00.0001612774836430.09.41633847663626e-058.003680818854757e-050.00030963225797297460.00.00.07.60514745428e-050.0002101367085656.582294857454473e-050.00.000162331475053792540.000234179342687004040.000254352766958505230.000141878016050182980.00.0002101367085650.00.00.00.000234179342687004040.000254352766958505230.000141878016050182980.0002681668686890.000359811279198000030.00027957788466713840.000237635059363935060.000287287662036780540.000372996994279815770.000405128891701949140.00030130795161172884.34600237383e-050.000191215573557000020.00013038007121496360.00.00.000173945713678567930.000188930300457158960.000210770706536089170.00025315802050.0004836103183240.00026329179429817890.000279740300464826470.000216441966738390.00065101857266987130.000445117342177384170.00035469504012545740.0004428185616310.0001050683542830.0007240524343199920.000279740300464826470.00032466295010758510.000117089671343502020.00012717638347925267.093900802509149e-054.34600237383e-050.00.00013038007121496360.00.00.00.00.00.00088047713164699990.00.0011299606171963510.00076835335861005670.00074311741913513920.00.00.00.000234508667135999989.14777747487e-050.00026329179429817890.000223792240371861170.000216441966738395.854483567175101e-050.00014494948054933457.093900802509149e-050.00.000456319013330999940.00.00.00.00052435809340785680.00063281002407550820.000211788922509693441.79496961144e-050.0001475135503190.00.05.384908834312602e-050.000174786031135952260.000126562004815101640.000141192615006462315.8627166783999995e-050.0001568366745436.582294857454473e-055.594806009296529e-055.41104916845975e-050.000191535573494000260.000208035442111123187.093900802509149e-050.00.0001760073623080.00.00.00.000117089671343502020.00012717638347925260.0002837560321003669.42433359848e-050.0002802875149286.55049633157305e-055.56777796094244e-050.000161547265029378060.000273623370171163440.00049664286710926037.059630750323114e-050.0001364654955650.000224705306779000030.000153214998941878120.000130229382815263850.000125952104938159160.000204410782175944240.00022201978810784780.000247685350053709260.000232860409265999990.000405850114506999940.00026076014242992720.0002770504898834340.00016077059548596760.000347891427357135870.000377860600914317860.00049179831525087470.00301360359903680030.0047541828547914990.00338077686182055570.00309883183454579470.00256120210073974060.0056339968179146980.0050392636391669240.00358928810729252250.00301360359903680030.0047541828547914990.00338077686182055570.00309883183454579470.00256120210073974060.0056339968179146980.0050392636391669240.00358928810729252250.000410199342479000060.0001352817663260.00041508757366396580.00058802552954851280.000227484924225042520.000123063634167150080.000133664974473092040.000149116690338457628.31861572168e-050.000303338877419000030.000136964923296527420.00.000112593548353808960.00036546170146608210.000396944469647364330.000147610461143119680.00.00.00.00.00.00.00.00.00.0001061578212940.00.00.00.000152661217068110240.000165812246814721780.00.0001693575390440.0001352817663260.00027672504910931050.000117605105909702570.000113742462112521290.000123063634167150080.000133664974473092040.000149116690338457620.000337008104380.000103536640522000020.000334803145835955830.0004009924935507030.00027522867375375520.000148891804300996420.0001617181172637410.00.00.00.00.00.00.00.00.00.08.98103427662e-050.00.00.00.000121820567155360690.00.000147610461143119680.00.00.00.00.00.00.00.08.44606996145e-058.47117967904e-050.000136964923296527420.00011641717554697830.00.000121820567155360690.00013231482321578810.00.00.00.00.00.00.00.00.03.79141540375e-058.91099829821e-050.00.00.000113742462112521290.00.000267329948946184070.07.82228862248e-050.00.00.00.000234668658674254430.00.00.00.00.0002343244529560.00.00.00.00012562745987896570.000272899322882562870.000304446576107684340.00.000127902542250999980.00.00.00.00024866466285321050.000135042963900649690.00.08.64584317758e-050.00.00.00.000124332331426605240.000135042963900649690.00.000121991882398999980.00.000136964923296527420.00011641717554697830.000112593548353808960.00.00.04.56549744322e-050.00.000136964923296527420.00.00.00.00.00.00.0005762679910710.00.00.00.00060910283577680350.00052925929286315240.00059044184457247870.000206452582014000050.00053056258937699990.000273929846593054840.00023283435109395660.000112593548353808960.000487282268621442760.00066157411607894060.00044283138342935910.000241543927150000020.000478129967321999940.00027119054812712430.000230506007583016980.00022293522574054170.00060301180741903560.000392975024950890630.000438403069595065360.00.000129679958584999980.00.00.00.000389039875754216360.00.00.00047519292986800010.00.00040476201213003620.00068807763457617020.000332739142896330860.00.00.00.000152673815933999980.0002678305676760.00.00023283435109395660.000225187096707617920.000243641134310721380.00026462964643157620.000295220922286239360.04.06068557185e-050.00.00.00.000121820567155360690.00.00.0001491011895990.0002672108763240.000167401572917977920.000142287659001862360.00013761433687687760.000297783608601992840.0003234362345274820.00018041278584159070.04.06068557185e-050.00.00.00.000121820567155360690.00.00.000214190832630.00070874569096300010.00041508757366396580.00.000227484924225042520.00086144543917005040.00066832487236546020.00059646676135383050.000206452582014000050.0002186270806280.000273929846593054840.00023283435109395660.000112593548353808960.000243641134310721380.00026462964643157620.000147610461143119680.000201025293902070020.00053165846988280.00.00035154525342586970.000251530628279603770.00056663813278430370.00055251852522671150.000475818751638990340.000201025293902070020.00053165846988280.00.00035154525342586970.000251530628279603770.00056663813278430370.00055251852522671150.000475818751638990340.000140479150180000020.00.00.00029404589297310420.000127391557566023810.00.00.08.97609063797e-060.00.02.6928271913903856e-050.00.00.00.01.71225211782e-059.97423086738e-050.00.05.136756353468703e-050.00011115425021549040.00012072965436279286.734302144317442e-050.07.20172285756e-050.00.00.08.533185954042954e-056.178852593567462e-056.893130025079644e-050.01.89626354534e-050.00.00.05.688790636028636e-050.00.00.00.000148961944942999990.00.00.00.000135508271330120320.000147181657284977750.000164195906215380330.04.27663628882e-050.00.00.00.06.064429397390287e-056.765479469059649e-052.00460315351e-055.74113553905e-050.03.057108853886167e-052.9567006066384838e-056.398003261740428e-056.949160476585156e-053.876242878824628e-051.44015003708e-050.00.00.04.3204501112508085e-050.00.00.00.03.12679554245e-050.00.00.02.844395318014318e-053.089426296783731e-053.446565012539822e-050.06.05286785338e-050.00.00.08.533185954042954e-056.178852593567462e-053.446565012539822e-050.00137872020690924010.00063880136007460020.00095618290277584350.00163174494219134740.00154823277575811930.00011940827869683870.00083877691981148420.00095821888171417780.00137872020690924010.00063880136007460020.00095618290277584350.00163174494219134740.00154823277575811930.00011940827869683870.00083877691981148420.00095821888171417780.000102410034992000020.08.474704628972636e-050.000108049691054539220.000114433367630373460.00.00.00.000300028902885999940.00.00020623153526900790.00043923045048926740.000254624722899358060.00.00.05.51532310288e-050.03.808856013021408e-056.474887853455534e-056.262225442150048e-050.00.00.06.21335634728e-060.00.00.01.8640069041851315e-050.00.00.01.29628711642e-050.00.01.9768954338166124e-051.911965915442039e-050.00.00.00.00.00.00.00.00.00.00.01.29628711642e-050.00.01.9768954338166124e-051.911965915442039e-050.00.00.02.04411659211e-050.02.2712776225052285e-053.861072153819381e-050.00.00.00.00.0001563641926060.00059073095883800010.000134252746597586310.000114111884942087650.000220727946277764120.00011940827869683870.00074620329791492580.00090658129990086047.891199004800001e-050.04.5578243382709965e-050.000116221516428411977.493621033295521e-050.00.00.00.0001343205172660.02.2636940578224066e-057.696360854190887e-050.00030336100267714750.00.00.02.60778559331e-050.00.05.956227586124471e-051.867129193806882e-050.00.00.00.00.00.00.00.00.00.00.03.26155319705e-050.02.2524131904246203e-053.829003448222874e-053.7032429525006925e-050.00.00.01.35751476786e-054.80704012366e-050.04.0725443035868734e-050.00.09.257362189655843e-055.163758181331748e-050.000401502469416000030.00.00037941092239907640.00045737063885452810.00036772584699509210.00.00.00.00.00.00.00.00.00.00.00.00.00.00.00.00.00.00.06.2237639793599995e-060.00.00.01.867129193806882e-050.00.00.01.25746320944e-050.00.01.917687251106631e-051.8547023772091656e-050.00.00.06.3816724137e-060.00.01.914501724111437e-050.00.00.00.00.00031484032385250.00113211159244490.00.00054138705616168540.00040313391539670230.0012088974721864120.00131333723482299080.00087410007032420180.00031484032385250.00113211159244490.00.00054138705616168540.00040313391539670230.0012088974721864120.00131333723482299080.00087410007032420180.00.00.00.00.00.00.00.02.08191567183e-050.00.03.175013878553954e-053.0707331369220626e-050.00.00.00.00.00.00.00.00.00.00.06.60789542219e-050.00.00.00019823686266567410.00.00.00.01.01796906731e-050.00.00.03.053907201925229e-050.00.00.00.00.000101947645174999990.00.00.09.401205811736238e-050.000138396527391215557.343435001592385e-055.36108501542e-050.00.09.785649799362249e-056.297605246907958e-050.00.00.00.00.0006184023137780.00.00.00.00068915635133604060.00074852385704931540.00041752673294768130.07.11976348099e-050.00.00.07.934365887092572e-058.617873354186196e-054.8070512017002776e-052.06485078927e-050.00.03.14898917463138e-053.0455631931767994e-050.00.00.00.02.7841224772e-050.00.00.03.3132516891155793e-050.05.039115742472016e-052.12285221593e-050.00.03.237443926727767e-053.131112721075024e-050.00.00.00.00.00.00.00.00.00.00.00.0001222746420330.000312722773910000030.00.000149679225703257820.000217144700396631570.00031325288697092750.000340238116840597940.00028467731791887360.0010396042053540.0022218027032760.00135628394550631890.0010233137977091680.00073921487284510350.00231298601063481240.00221787531839776840.0021345467807999440.0010396042053540.0022218027032760.00135628394550631890.0010233137977091680.00073921487284510350.00231298601063481240.00221787531839776840.0021345467807999440.000186483582317999980.000148961944942999990.000304708481041712650.000129497757069110670.000125244508843000960.000135508271330120320.000147181657284977750.000164195906215380330.00.000297923889887000040.00.00.00.000271016542660240650.00029436331456995550.000328391812430760670.00.0001450708162920.00.00.00.000271016542660240650.00.000164195906215380330.000393821620354000030.00.00044215850238118090.00037582501236361470.000363481346316100660.00.00.00.00.000446885834829999940.00.00.00.00040652481399036090.00044154497185493330.00049258771864614090.00.000446885834829999940.00.00.00.00040652481399036090.00044154497185493330.00049258771864614090.00.000297923889887000040.00.00.00.000271016542660240650.00029436331456995550.000328391812430760670.0004592990026820.000148961944942999990.00060941696208342530.00051799102827644270.00025048901768600190.000135508271330120320.000147181657284977750.000164195906215380330.00.000289188547664000040.00.00.00.00041587021201312790.000451695430978035170.00.00079177156364920.00121626041773580.000291602739921639060.00136057080659845310.00072314114442720520.00119335373382691840.00229658582749817830.000158841691882270070.00079177156364920.00121626041773580.000291602739921639060.00136057080659845310.00072314114442720520.00119335373382691840.00229658582749817830.000158841691882270070.00.00.00.00.00.00.00.00.00.00.00.00.00.00.00.08.17397190011e-059.29018971515e-050.00.000245219157003209560.00.00.00027870569145453230.00.09.01770740027e-050.00.00.00.000129679958584738820.00014085126342325830.04.12843010631e-050.0002795489294080.00.00.000123852903189189840.000402007871612690350.00043663891661210070.00.04.79822985288e-050.00.00.00.00.000143946895586406810.00.0001638805104824.69504211411e-050.00.00037178388319841450.000119857648247603070.00.00014085126342325830.00.04.46675412903e-050.00.00.00.000134002623870896770.00.00.0005048670331030.0002705312220080.000291602739921639060.0007435677663968290.00047943059299041230.000389039875754216360.00042255379026977490.00.00.000147868734238999980.00.00.00.00.00028476451083397870.000158841691882270070.09.92361174118e-050.00.00.00.00.00029770835223552320.00.09.63961825546e-050.00.00.00.000138623404004375950.000150565143659345050.00.00161438001780189980.00419440090018839960.00111110217734710980.0020294042478190480.0017026336282380.0049171136567839860.0045896415380178530.00307644750576449920.00161438001780189980.00419440090018839960.00111110217734710980.0020294042478190480.0017026336282380.0049171136567839860.0045896415380178530.00307644750576449920.00.00.00.00.00.00.00.00.0004659692542510.0003550616850710.000473006769989170230.00053606048275120240.00038884051001257280.000420705912152815360.00030463180228751210.00033984734077136854.36564408301e-050.0001906015427780.00.000130969322490350570.00.000274096276099561550.00029770835223552320.04.42331797104e-059.98389033601e-050.00.00.000132699539131274820.00014357423986167510.000155942470218607380.04.42331797104e-050.00.00.00.000132699539131274820.00.00.00.000182941275860.0009828124430090.00.00054882382757861190.00.00100501967903172560.0012475397617488590.00069587788824613554.42331797104e-050.00.00.00.000132699539131274820.00.00.00.00.00.00.00.00.00.00.00.0001778200914990.0004146824062610.00.00027118353833296120.00026227673616534320.00028377026231484020.00061643141168767160.00034384554478044350.000142084663030.000155971918822999970.00015952385183948490.00013559176916648060.00013113836808267160.00014188513115742010.00015410785292191790.000171922772390221740.00.000157828727381000020.00.00.00.00014357423986167510.000155942470218607380.000173969472061533884.29547641119e-050.000455798021385999950.00.00.000128864292335573250.00055769878142800970.000302870924817642070.00050682435791337040.09.34902027006e-050.00.00.00.000280470608101876960.00.09.83718736687e-050.0002505620062610.00015952385183948490.00013559176916648060.00.00042565539347226030.00015410785292191790.000171922772390221740.09.11572595897e-050.00.00.00.000131089523351964230.000142382255416989350.00.00.0004373232898690.00.00.00.00054203308532048140.00044154497185493330.000328391812430760670.000327882115420000040.00050927249369899990.000319047703678969730.00027118353833296120.00039341510424801480.00056754052462968040.00061643141168767160.00034384554478044358.14020923909e-050.000194038427352999970.00.00.000244206277172788370.00039486796346821788.850788850245284e-059.87394300889787e-058.14020923909e-050.000194038427352999970.00.00.000244206277172788370.00039486796346821788.850788850245284e-059.87394300889787e-055.62967741769e-050.00.00.00.000168890322530713440.00.00.00.00.00.00.00.00.00.00.00.00.00.00.00.00.00.00.00.00.00.00.00.00.00.00.00.00.00.00.00.00.00.00.02.5105318214000002e-050.000194038427352999970.00.07.531595464207491e-050.00039486796346821788.850788850245284e-059.87394300889787e-050.00.00.00.00.00.00.00.00.00.00.00.00.00.00.00.00.00.00.00.00.00.00.00.00.00074903471419180.00113310173510390010.00068976726371464220.00072687559208126420.00083046128677833730.00140951726760442480.0010487785074495810.00094100943025651210.00074903471419180.00113310173510390010.00068976726371464220.00072687559208126420.00083046128677833730.00140951726760442480.0010487785074495810.00094100943025651218.738713941399999e-050.00.08.934341379186706e-050.000172818004450032340.00.00.00.000361160754501999960.0004913353622570.00054238109625424860.00018440480606641360.00035669636118486680.00048240944593522840.00052396669993452080.000467629940901403130.05.1211193836e-050.00.00.00.000153633581508034490.00.03.14898917463e-053.99274198174e-050.09.44696752389414e-050.00.00.00.000119782259452203690.00.00.00.00.00.00.00.00.06.38107732719e-050.00.00.00.000191432319815566830.00.05.69150636007e-050.00.00.000170745190802234830.00.00.00.05.99288241238e-056.7632805502e-050.00.00.000179786472371404589.725996893855409e-050.000105638447567443730.00.00.000276443069057999960.00.00.00.000385927556748182650.000209586679973808320.000233814970450701620.06.98622266579e-050.00.00.00.00.000209586679973808320.00.0001521530408050.00.000147386167460393620.00018791250618180730.000121160448772033540.00.00.00.07.28788847037e-050.00.00.09.885439465885827e-050.00.000119782259452203690.00.00025530451833270.00.00.00.00035229261243670730.000195509962662134650.00021811097989804250.00.00025530451833270.00.00.00.00035229261243670730.000195509962662134650.00021811097989804250.00.0001978748223880.00.00.00.000180003524602697140.000195509962662134650.00021811097989804250.05.74296959447e-050.00.00.00.000172289087834010140.00.00.00142958050216190.000176826505925699980.0021680743253394660.00114470116271060510.00097596601843723640.00.00053047951777652970.00.00142958050216190.000176826505925699980.0021680743253394660.00114470116271060510.00097596601843723640.00.00053047951777652970.00.0008794089949060.00.00143571466655536410.0006779588458324030.00052455347233068640.00.00.06.10789522809e-050.00.000183236856842651550.00.00.00.00.00.000280865031569999970.0001015439340960.00031533784665944680.00026803024137560120.000259227006675048540.00.00030463180228751210.00.0002082275234057.52825718297e-050.000233784955282003660.000198712075502600870.00019218553943150150.00.000225847715489017580.00.00639912351137260.0075369400631120.0072297621303797980.0061335221540177890.0058340862497179550.0079792128392375890.006951360368604530.0076802469815036470.00639912351137260.0075369400631120.0072297621303797980.0061335221540177890.0058340862497179550.0079792128392375890.006951360368604530.0076802469815036478.62021573385e-050.00.00013978894233356920.000118817529681967530.00.00.00.00.000294565764817000040.00.00033072018064283450.00028110488729636220.000271872226512855770.00.00.00.03.29514648863e-050.00.00.09.885439465885827e-050.00.00.00066909051459000010.000286079459690.0008891493381217190.00066128772667258970.000456834478976519940.00029656318397657480.000322110676189254560.000239564518904407320.000227611052910999980.00.000220480120428556340.00028110488729636220.00018124815100857050.00.00.00.0001963771765450.0002510700436270.000220480120428556340.000187403258197574830.00018124815100857050.000196101400786678240.00031949189020397610.000237616839888924429.24011556098e-050.00.09.44696752389414e-050.0001827337915906080.00.00.03.79818694856e-050.000365097542571999960.000113945608456774920.00.00.00050673261127650030.000220154075602739850.000368405940836189376.85377808347e-050.00.000111143667265214879.44696752389414e-050.00.00.00.03.04556319318e-050.00.00.09.1366895795304e-050.00.00.00.0001059403189260.0001162948517540.00011894322286277380.00010109912613290229.777860778093934e-050.000105791545161234270.000114904978055815970.000128188032045340770.03.52638483871e-050.00.00.00.000105791545161234270.00.00.00.000186124680955999970.00.00.00.00020269304451060010.000110077037801369930.00024560396055745960.00.0001228370950330.00.00.00.00023881655739367740.000129694727706564540.09.34354154244e-050.00.00.00018893935047788289.1366895795304e-050.00.00.00.07.47161675113e-050.00.00.00.000101346522255300080.00.00012280198027872980.0008643733241410.00116264384113000020.00107996235979828260.00101993808665051770.00049321952597464980.0012807330423059160.00104329652641829350.00116390195467716480.0001059403189260.000151558700141000010.00011894322286277380.00010109912613290229.777860778093934e-050.000211583090322468550.000114904978055815970.000128188032045340770.00.000177481421101999980.00.00.00.000301505903709517740.000109159729153025150.000121778630443073739.89934127665e-050.00.000111143667265214879.44696752389414e-059.1366895795304e-050.00.00.00.0001563641926060.000259098175742000030.000134252746597586310.000114111884942087650.000220727946277764120.000358224836090516130.000129694727706564540.000289374963429086140.00.00.00.00.00.00.00.00.0003834179353090.00098041121120.00032673560015316180.000372187756664263150.0004513304491093330.00092422903922016870.00095339585683325570.0010636087375454710.0008528585659950.000354820999709000040.0008891493381217190.00075575740191153120.00091366895795303990.00039541757863543310.00042948090158567280.000239564518904407320.00.00.00.00.00.00.00.06.85377808347e-050.0005145306789780.000111143667265214879.44696752389414e-050.00.00039541757863543310.00042948090158567280.00071869355671322197.22406102146e-050.0002325897035080.00011894322286277380.09.777860778093934e-050.000211583090322468550.0002298099561116320.00025637606409068150.00.000102533442962000010.00.00.00.000199342746254226580.000108257582631099360.09.89934127665e-050.0002572653394890.000111143667265214879.44696752389414e-059.1366895795304e-050.000197708789317716520.00021474045079283640.000359346778356610960.000267558866182000030.000434675839343000030.00033343100179564460.00037787870095576569.1366895795304e-050.00039541757863543310.00042948090158567280.000479129037808814639.89934127665e-050.000365934299324999950.000111143667265214879.44696752389414e-059.1366895795304e-050.00029656318397657480.000322110676189254560.000479129037808814630.0001632053561830.0002381621927850.000183236856842651550.000155747302420957440.000150631909284149820.00016297616416730690.00035403155400981140.00019747886017795740.000338093140095000030.000108668959836000010.000344445239092197359.44696752389414e-050.00057536450595421179.885439465885827e-050.00010737022539641820.000119782259452203690.0005558783276963.99274198174e-050.00055571833632607430.000472348376194707040.00063956827056712780.00.00.000119782259452203690.00.0001481008593790.00.00.00.000101346522255300080.000220154075602739850.00012280198027872980.000104551410108999990.000217337919672000020.000222287334530429750.09.1366895795304e-050.000197708789317716520.00021474045079283640.000239564518904407320.00.00.00.00.00.00.00.00.0002665246063680.0002502893845580.00033343100179564460.000283409025716824160.0001827337915906080.00029656318397657480.00021474045079283640.000239564518904407320.07.04745200189e-050.00.00.00.000101346522255300080.000110077037801369930.00.00166534006170990.00122849045301549970.00122827798725876160.00159084604059541130.00217689615727526460.00104304126243974740.00136528304264244350.00127714705396507070.00166534006170990.00122849045301549970.00122827798725876160.00159084604059541130.00217689615727526460.00104304126243974740.00136528304264244350.00127714705396507070.03.57900751321e-050.00.00.00.00.00010737022539641820.00.0004420129201040.00.000252498783013542550.00057199207308054020.00050154790421911560.00.00.00.000119447235533999990.00.00013410795153838010.00011398881232305330.000110244942739654720.00.00.03.11002217643e-050.00.04.7429219667287455e-054.587144562562587e-050.00.00.03.65089559842e-050.00.05.56777796094244e-055.384908834312602e-050.00.00.07.36414412043e-058.08391042681e-058.268004516070863e-057.027622182409055e-056.796805662821394e-057.353802529500433e-057.987297255099403e-058.910631495834663e-050.07.98711226881e-050.00.00.00.000114859391889340090.000124753976174885920.02.34254072747e-050.00.07.027622182409055e-050.00.00.00.00.00.000139045963807999980.00.00.00.00013635175523448720.000204674492161922187.611164402692107e-057.24304040008e-052.97021049861e-058.217895397791645e-050.00.000135112258024570730.00.08.910631495834663e-053.93183819648e-050.00.00.00.000117955145894466530.00.00.00.0003123177875160.00.000248040135482125830.00028110488729636220.00040780833976928360.00.00.00.08.08391042681e-050.00.00.07.353802529500433e-057.987297255099403e-058.910631495834663e-052.26560188761e-050.000105351779365999990.00.06.796805662821394e-050.000147076050590008667.987297255099403e-058.910631495834663e-050.00.000242691784825999960.00.00.00.00012562745987896570.00028476451083397870.000317683383764540140.0002160203274360.00.000165360090321417260.000210828665472271670.000271872226512855770.00.00.09.52714639917e-050.0003399387974350.00.00.00028581439197505350.00030923682431745410.000335876089701615850.00037470347828638076.2902064362e-059.442061623799998e-057.062253857477194e-056.0027606141410684e-055.805604836993274e-056.281372993948285e-056.822483072064073e-050.000152223288053842140.000118287431697000010.00.00019278948918989880.000109244553356880095.282825254515207e-050.00.00.00.000225992123439000020.0001455463055370.00067797637031781080.00.00.00.00043663891661210070.00.000225992123439000020.0001455463055370.00067797637031781080.00.00.00.00043663891661210070.00.000225992123439000020.0001455463055370.00067797637031781080.00.00.00.00043663891661210070.00.00397195652509960050.00214303260253910.004408952800461090.00352127087006762080.003985645904766560.00284443676480117730.00221812118044999870.00136653986236529240.00397195652509960050.00214303260253910.004408952800461090.00352127087006762080.003985645904766560.00284443676480117730.00221812118044999870.00136653986236529246.979177597e-050.0001723538282570.00011491124920640860.09.446407870361935e-050.000282208915690669330.00011100989405392390.000123842675026854630.0005973798996630.00.00084747046289726340.000480220849131285370.000464448386959461930.00.00.00.000351127545940.00.0004270717293340540.00036300158674490870.00026330932174079730.00.00.00.0002852881029330.00.000320303797000540460.00027225119005868150.00026330932174079730.00.00.00.0002550379707053.43810170561e-050.000320303797000540460.000181500793372454370.00026330932174079730.00.000103143051168212760.00.0001574448726820.00.00.000240110424565642680.00023222419347973090.00.00.06.51146914076e-050.000105140225410.00.000195344074222895750.00.000204410782175944240.00011100989405392390.03.14880262345e-050.00.00.09.446407870361935e-050.00.00.00.0002206846139870.00.00.000193702527380686540.00046835131458097010.00.00.00.00.000140949040037999980.00.00.00.00020269304451060010.000220154075602739850.03.83037497355e-050.00.00011491124920640860.00.00.00.00.00.00.00.00.00.00.00.00.00.0002046982433480.0006639686580360.00022982249841281720.000195344074222895750.000188928157407238760.00081764312870377690.00055504947026961950.00061921337513427320.0001770801958770.0003269106978670.00022982249841281729.767203711144788e-050.00020374605210584570.00051102695543986050.00022201978810784780.000247685350053709260.00045862770977900010.00.00051491876226669170.000437669634651298160.000423294732418750160.00.00.00.00.00.00.00.00.00.00.00.0001973540307950.00.00038017366559877240.000107713087655615420.000104175339131094260.00.00.00.000480402034964999970.00.00044365440899536220.000458672477695907330.00053887921820296550.00.00.00.00.000112352653390.00.00.00.000102205391087972120.00011100989405392390.000123842675026854636.979177597e-050.0001791468214460.00011491124920640860.09.446407870361935e-050.000204410782175944240.000333029682161771660.00.0003123412851080.00040782966103900010.00035067743292300550.000298068113253901340.000288278309147252170.00051983776501640990.000451695430978035170.000251955787123600830.00.00.00.00.00.00.00.00.00.00.00.00.00.00.00.00.00.00.00.00.00.00.00.00.00031831051087480.000372534674234900066.059927618025153e-050.00053036944996090030.000363962806483246570.00031893174717263480.00024003297669863240.0005586392988321960.00031831051087480.000372534674234900066.059927618025153e-050.00053036944996090030.000363962806483246570.00031893174717263480.00024003297669863240.0005586392988321960.04.86506924899e-050.00.00.03.239834816633327e-053.511841152376145e-057.843531777948487e-053.38360421223e-050.00.07.582434459967665e-052.5683781767343523e-050.00.00.00.00.00.00.00.00.00.00.00.02.48418215732e-050.00.00.02.1769379329207054e-050.05.275608539050126e-051.8568437073e-050.00.02.831769134926499e-052.738761986984542e-050.00.00.00.00.00.00.00.00.00.00.05.76348493122e-050.03.6255420872610205e-057.704077793550034e-055.960834912848708e-050.00.00.04.27990908291e-050.00.04.1758334707068304e-058.663893778027847e-050.00.00.00.00.00.00.00.00.00.00.00.02.47970615344e-050.00.00.02.1730155222307583e-050.05.2661029380788644e-050.03.47387368486e-050.00.00.05.826201037865077e-050.04.595420016719764e-050.01.30945860587e-050.00.00.01.776176163237218e-050.02.1521996543694915e-050.00.00.00.00.00.00.00.00.00.00.00.00.00.00.00.00.000100365641264000010.00.00.000176477083366271480.00012461984042552510.00.00.03.52505390417e-050.000138027688397000032.4343855307641322e-054.138348430574812e-054.002427751176692e-058.660851812122592e-050.000117586781852450830.00020988776521606960.08.83840873331e-050.00.00.08.040157432253806e-058.732778332242014e-059.742290435445898e-052.98559112325e-050.00.08.956773369737032e-050.00.00.00.00.00.00.00.00.00.00.00.00.00.00.00.00.00.00.00.00.00.00.00.00.00.00.00.00.00.00.00.00.00.00.00.00.00.00.00.00.00.00.00.00.00.00.00.00.00.00.00.00.00.00.00.00.00.00.00.00.000155173874000650.00060330694293123.6062572889245254e-050.000235048467241676740.000194410581871283640.00049841260095836560.00053130357833744280.00078020464949700150.000155173874000650.00060330694293123.6062572889245254e-050.000235048467241676740.000194410581871283640.00049841260095836560.00053130357833744280.00078020464949700159.93472485475e-060.00.00.02.980417456424354e-050.00.00.03.36382239679e-050.03.6062572889245254e-056.485209901457286e-050.00.00.00.00.00.00.00.00.00.00.00.00.00.00.00.00.00.00.00.00.00.00.00.00.00.00.00.08.35066728036e-050.0002197394436460.00.000127351385404981770.000123168633005824160.000199893416823989640.000217113273453530690.000242211640660257120.00.00.00.00.00.00.00.00.00.00.00.00.00.00.00.02.80942523744e-053.43402765199e-0
[truncated: 569,479 more chars]
